# A global map for introgressed structural variation and selection in humans

**DOI:** 10.1126/science.adz7518

**Published:** 2026-06-11

**Authors:** PingHsun Hsieh, Natthapon Soisangwan, David S. Gordon, Athef Javidh, William T. Harvey, David Porubsky, Kendra Hoekzema, Carl Baker, Katherine M. Munson, Christopher Kinipi, Matthew Leavesley, Nicolas Brucato, Murray P. Cox, François-X Ricaut, Irene Gallego Romero, Evan E. Eichler

**Affiliations:** 1. Department of Genetics, Cell Biology, and Development, University of Minnesota, Twin Cities, MN, USA; 2. Institute for Health Informatics, University of Minnesota, Twin Cities, MN, USA; 3. Bioinformatics and Computational Biology Graduate Program, University of Minnesota, Twin Cities, MN, USA; 4. Department of Genome Sciences, University of Washington School of Medicine, Seattle, WA, USA; 5. The University Clinic, University of Papua New Guinea, Port Moresby, Papua New Guinea; 6. Strand of Anthropology, Sociology and Archaeology in the School of Humanities and Social Sciences, University of Papua New Guinea, National Capital District, Papua New Guinea; 7. CIEHF and College of Arts, Society and Education, James Cook University, Cairns, Australia; 8. Centre de Recherche sur la Biodiversité et l'Environnement (CRBE), Université de Toulouse, CNRS, IRD, Toulouse INP, Université Toulouse 3 – Paul Sabatier (UT3), Toulouse, France; 9. College of Sciences, Massey University, Palmerston North, New Zealand; 10. Department of Statistics, University of Auckland, Auckland, New Zealand; 11. Human Genomics and Evolution, St Vincent's Institute of Medical Research, Fitzroy, Australia; 12. Center for Genomics, Evolution and Medicine, Institute of Genomics, University of Tartu, Tartu, Estonia; 13. School of Medicine, University of Melbourne, Melbourne, Australia; 14. Howard Hughes Medical Institute, University of Washington, Seattle, WA, USA

**Keywords:** Human evolution, adaptive introgression, structural variation

## Abstract

Genetic introgression from Neanderthals and Denisovan shaped modern human genomes; however, introgressed structural variants (SVs ≥50 base pairs) remain challenging to discover. We integrated high-quality phased assemblies from four new Papua New Guinea (PNG) haploid genomes with 94 published assemblies of diverse ancestry to infer an introgressed SV map. Introgressed SVs are enriched in genes (47%), including critical genomic disorder regions, and most abundant in PNG. We identify 11 centromeres likely derived from archaic hominins, adding unexplored diversity to centromere genomics. Pangenome genotyping of these 98 assemblies across 1,363 samples reveals 16 adaptive SVs, many associated with immune-related genes and expression, in the PNG. We hypothesize that archaic SVs contributed to reproductive success, underscoring introgression as a significant force in human adaptive evolution.

## INTRODUCTION

Over a decade of genomic research has shown that interbreeding occurred between archaic hominins, such as Neanderthals and Denisovans, and the ancestors of modern humans through multiple contacts over the past 100,000 years (1–6). Analyses based on single-nucleotide variants (SNVs) reveal that present-day Eurasians derive 2–5% of their ancestry from archaic sources, with the highest levels in Papua New Guinea (PNG) (5, 7). Despite selection against deleterious alleles, introgressed sequences have contributed to human phenotypic diversity, with many loci showing signals of positive selection in genes related to gene regulation, immunity, metabolism, and disease (8–16). However, our understanding of archaic introgression remains incomplete because most studies focus on SNVs, leaving other classes of variation, especially structural variants (SVs), underexplored. SVs, including insertions, deletions, and inversions, affect more bases than SNVs and often have larger effects on gene expression and phenotypes (17, 18). Many SVs are implicated in disease (e.g., 22q11.2 deletion syndrome, *LPA*-associated coronary disease) (17, 19) or adaptive evolution, including diet and brain expansion (20, 21). Although recent studies suggest adaptive SV introgression (12, 14, 16), most relied on short-read sequencing, which fails to capture the full spectrum of SVs in repetitive, complex regions (18, 19, 22, 23).

Advances in highly accurate long-read sequencing now enable near-complete resolution of complex genomic regions and SVs (18, 19, 22–24). Large-scale efforts such as the Human Genome Structural Variation Consortium (HGSVC) (18) and the Human Pangenome Reference Consortium (HPRC) (19) reveal that over 70% of SVs are inaccessible to short reads, with many located in disease- and trait-associated genes. Here, we construct a genome-wide map of introgressed SVs by projecting variants onto archaic segments identified in the HPRC Release I (HPRCr1, *n*=47) and two new long-read phased haplotype assemblies from Papuan individuals (PNG15 and PNG16). The inclusion of Papuan genomes, absent from the 1000 Genomes Project yet harboring the highest known levels of archaic ancestry (4, 5, 25), fills a major gap in the human pangenome and provides a powerful resource for discovering introgressed SVs. Using population genetic analyses and simulations, we first constructed a comprehensive map of SV introgression and delineated introgressed segments and SVs across this diverse population panel. We identified candidate archaic introgressed centromeres in the Papuan assemblies, offering the first insights into centromere evolution in archaic hominins. We further uncovered multiple high-confidence adaptive SV introgression signals in Papuans by pangenome genotyping of these 98 assemblies in a large short-read cohort. Our results provide new insights into the role of SV introgression in human evolution and biology.

## RESULTS

### High-quality, haplotype-phased Papuan assemblies.

We generated high-coverage sequencing data for a female from Western Lowland Papuan New Guinea (PNG15) and a male from the Solomon Islands (PNG16) using PacBio high-fidelity (>31-fold coverage), Oxford Nanopore Technologies (>44-fold coverage), Arima Hi-C (>40-fold coverage), and Illumina short-read platforms (>18-fold coverage) (Methods). A principal component analysis using SNVs from short-read genomes places the two PNG individuals, along with published short-read PNG samples (26), separately from the clusters of 1KG cohort (27) (Fig. 1A). Using these data, we constructed four nearly complete haplotype-phased assemblies (Table S1) ranging from 2.89 to 3.01 billion base pairs using Verkko v1.4.1 (28), with N50 values of 111–136 million base pairs (scaffolded) or 62.4–74.9 million base pairs (unscaffolded), surpassing both the human reference genome GRCh38 and most of the HPRCr1 assemblies (Fig. 1B). The PNG assemblies have an average QV accuracy of 44.5 (an error rate of 3.5×10^−5^ per base pair, Table S1), with limited collapsed and misassembled sequences (0.18% and 0.03%, respectively) (Tables S2–S4; Methods). We estimated that >99.3% of each PNG haploid assembly was completely and accurately assembled, which was highly compatible with the T2T-CHM13v1.1 assembly. Alignment to the T2T-CHM13v1.1 reference revealed 91.3‒95.4% unique alignments, with gaps primarily in acrocentric chromosomes and centromere regions (Figs. S2–S3). Overall, these high-quality PNG assemblies provide an important resource for comparative genomics research in humans.

### Genomic variation in the Papuan assemblies.

We assessed genomic variation in the four PNG haplotype assemblies relative to GRCh38 using both assembly-based callers (PAV v1.1.2, SVIM-ASM v1.0.3) and read-based callers (Sniffles2 v2.2, PBSV v2.9.0) (Methods). Because merging SVs across call sets is challenging, we used PAV as the primary call set given its high precision (18) and employed others for support (Methods). For comparison, we included the HPRCr1 PAV call set. PAV identified ~4.4 million SNVs and ~1 million indels (<50 bp) per PNG genome, consistent with non-African HPRCr1 samples (Fig. S3) (29). After quality control (29), ~12% of small variants were unique to PNG (7, 26). For large SVs (≥50 bp), the callers collectively detected ~30,000 insertions, ~23,000 deletions, and ~175 inversions per genome (29). Over 89% of PAV SVs had support from other callers (Fig. S4) (29). PAV-specific SVs were significantly longer than those identified by multiple callers (Fig. S4, Table S5, Mann-Whitney U [MWU] test, p<1.0×10^−7^ regardless of SV type), reflecting improved assembly sensitivity. Joint analysis with HPRCr1 showed 5.6% of SVs were PNG-specific (Fig. 2A,B), which was slightly higher than East Asian pairs (4.7–4.9%) and comparable to admixed Americans (5.2–6.6%). PNG-specific insertions and deletions constitute 4% and 6% of the total, respectively (Fig. S5). Because inversions had low concordance (<15%) and high population specificity (29), we focused on insertions and deletions for most of downstream analyses unless noted otherwise. Similar to previous studies (18, 19), we found an inverse relationship between SV length and abundance in the PNG genomes; e.g., 97.6% of SVs are shorter than 10 thousand base pairs (kbp) (Fig. S6). Overall, the PNG-specific insertions and deletions account for 6.8 and 8.6 Mbp, respectively. Roughly one-third of PNG-specific SVs overlapped with genes but were significantly depleted in genic regions (p<0.001) (29), consistent with purifying selection against large SVs (18).

The phased assemblies also enabled analysis of segmental duplications (SDs), highly identical sequences (>90% and >1 kbp in length) in humans known to be linked to genomic rearrangements and associated with neurodevelopmental delay and psychiatric disorders (21, 30). We first quantified nonredundant SD bases, defined as genomic loci covered by any SDs detected in the genome. Across PNG, HPRCr1, and T2T-CHM13v1.1 assemblies, we identified on average 157 Mbp of nonredundant SD bases (range: 125–173 Mbp) in individual PNG and HPRCr1 assemblies, compared to 183 Mbp in T2T-CHM13v1.1, with differences mainly in centromeric and acrocentric regions (Figs. 2C and S7) (29). To identify sample-specific SDs, we conservatively analyzed 172,624,644 nonredundant SD bases (5.75%) located within assembly sequences exhibiting ≥1 Mbp of synteny with T2T-CHM13v1.1 and excluding acrocentric and centromeric regions (Fig. S8). Among these SD sequences, 0.3%, 0.8%, and 20.4% are private to PNG, T2T, and HPRCr1 haplotypes, respectively, while SD bases found in common are at higher frequencies than those private to PNG, T2T, or HPRCr1 haplotypes (Figs. S9–S13). PNG-specific SD pairs are significantly higher in identity (one-sided MWU tests, uncorrected p<0.02, except for CHS and YRI) and longer in length (one-sided MWU tests, uncorrected p<0.01, except for YRI) compared with other population-specific SD pairs (Figs. S14–S15).

Across all assemblies, 8.2% (n=1,039) of the PNG-specific SVs overlap with SD sequences; 76 of these SVs also affect exonic sequences (Table S6). Several examples are located at *TPPP*/*CEP72*, *HLA-H/HCG4B*, *TCAF*2, *HBA2*, 15q11.3, and 22q11.2 loci (Figs. 2 and S16–S26). Among these, in three of the four PNG haplotypes, we observed a cluster of insertions along the *TPPP*/*CEP72* locus (Figs. 2D and S16); of which, a 94 bp sequence is inserted in the fourth exon of *TPPP*, which has known associations with diseases such as cystic fibrosis, chronic kidney disease, and multiple sclerosis (31, 32). In addition, three of the four PNG haplotypes carry a large, 54,848 bp deletion that removes both *HLA-H* and *HCG4B* (Figs. 2E and S17), which play a role in chronic obstructive pulmonary disease and pulmonary function (33). Several of these PNG-specific SVs also locate at known large copy number variable loci targeted by natural selection (20, 34). We detected a known 4.2-kbp deletion removing *HBA2* (Fig. S18), an α-globin gene that forms part of the hemoglobin molecule. This deletion causes α-thalassemia, a common blood disorder associated with mild to severe anemia (35). Its high frequency in malaria-endemic coastal Papua New Guinea and nearby islands suggests malaria-driven selection (36). At the *TCAF* locus, for example, while structurally similar to a previously published haplotype (e.g., VMRC53_hapB, Fig. S19), PNG15 haplotype 1 carries an extra copy of *TCAF2* compared to other known Melanesian *TCAF* haplotypes (e.g., VMRC73_hapA, Fig. S19), showing previously uncharacterized haplotype diversity at this locus in Oceania (20).

One of the most diverged SD loci is located within a 1.8 Mbp inversion on PNG15 haplotype 2 at chromosome 15q13.3 (Fig. 2F), a region known for recurrent inversions (37) and microdeletions associated with idiopathic epilepsies (38). While no large deletions or duplications are observed, this inversion effectively relocates and reorients a large number of genes, including *ARHGAP11B*, a human-specific gene playing a crucial role in human neocortex expansion (39). Another diverged SD locus found in the PNG15 haplotype 1 overlaps with a 0.68 Mbp deletion at chromosome 22q11.2 (Figs. 2G and S20), within the low-copy repeat 22A (LCR22A) region. While we found three HPRCr1 haplotypes with similar deletions at this locus with breakpoints off by 10 kbp from each other, further sequence analysis supports that these deletions share an ancestral origin (Fig. S20). Our analysis also shows the breakpoints of this deletion locate within homologous exonic sequences of the paralogous genes *FAM230D* (the eighth exon; chr22:18,185,813–18,186,274, GRCh38) and *FAM230F* (the eighth exon; chr22:18873303–18873780), mapping to pair of SDs that share >98.8% sequence identity. We infer this deletion is likely an ancestral state as it presents the same sequence structure as a high-quality chimpanzee assembly (Fig. S21) (40). This region corresponds to the proximal breakpoint of the 22q11.2 deletion syndrome (22q11.2DS or DiGeorge syndrome) locus, the most frequent microdeletion disorder (~1 in 4,000 births), driven by flanking LCR22A–D repeats (17). This 0.68 Mbp deletion creates a much shorter SD at LCR22A, potentially reducing the risk of genomic rearrangement at the 22q11.2DS locus. These findings highlight the importance of expanding genomics into understudied populations for a better understanding of human genomic variation and show that the PNG haplotype-phased assemblies carry substantial and complex genomic diversity that has not yet been fully characterized.

### A global map of genomic introgression from archaic hominins.

Modern non-African genomes carry 1–4% archaic hominin DNA, e.g., from Neanderthals (NDL) and/or Denisovan (DNS), with higher levels in Papuans (5, 7, 26). To detect SV introgression, we used three approaches to identify archaic sequences in modern human autosomes and projected SVs onto inferred introgressed haplotypes (Methods). Using archaic reference-free methods, Sprime (5) and hmmix (41), we first identified genomic segments showing signatures of archaic introgression. Because >24% of PNG segments showed <10% derived-allele matching to archaic-specific alleles (Figs. 3A, S27–S28), we excluded these low-affinity regions to reduce false positives. We then applied an HMM-based method (42) leveraging high-coverage archaic genomes (1–4) to refine candidate introgressed haplotypes. To ensure robust inference, we retained segments detected by at least two of the three methods, given their substantial call heterogeneity (Fig. S29) (43).

In total, our analysis identified 479 Mbp of introgressed sequences from the four PNG haplotypes, which span over 338 Mbp of the genome. This corresponds to 4.12% and 4.69% of the PNG15 and PNG16 genomes, respectively, predicted to have archaic origins (Figs. 3B and S30). Specifically, 2.12% and 2.37% of the PNG15 and PNG16 diploid genomes, respectively, likely originate from Neanderthals, with 1.34% and 1.64% derived from Denisovans, with additional 0.66 and 0.68% sequences showing unresolved archaic ancestry (Fig. S30). Segments with unresolved archaic ancestry are likely due to the actual source populations of archaic admixture and complex demographic dynamics during hominin evolution (44, 45). In all cases, the length distribution of introgressed segments is wide with a long tail (median NDL track: 36.0 kbp, s.d.: 136 kbp; median DNS track: 29.8 kbp, s.d.: 107 kbp) (Fig. S30). Taking advantage of our haplotype-phased assemblies, we constructed a map of introgressed SVs in the PNG samples by physically projecting all SVs identified onto the introgressed haplotypes. Our analysis identified 1,521 deletions and 2,271 insertions mapping to 1,957 putatively introgressed haplotype segments (or 241.5 Mbp of nonredundant DNA) potentially derived from archaic hominins (Fig. 3C, Table S6). In line with its higher introgression level, PNG16 contains 22% more introgressed insertion bases and 14% more deletion bases than PNG15. (Fig. S31). In addition, Neanderthal-derived deletions were twice as abundant as Denisovan ones, while Denisovan insertions contained 29% more bases than Neanderthal insertions, despite comparable length distributions. (Fig. S31).

To build a comprehensive view for the extent of introgressed SVs in modern-day humans, we applied the same analysis to the non-African genome assemblies from the HPRCr1 cohort. Overall, our analysis showed that the PNG samples carry twofold more DNA derived from archaic hominin compared to other non-African HPRCr1 samples (mean fraction: 4.40% in PNG, 1.34% in AMR [admixed American, range: 1.50–2.66%, n=16], 2.56% in EAS [East Asian, range: 2.49–2.61%, n=5], 1.40% in EUR [European, n=1], and 1.92% in SAS [South Asian, n=1]) (Figs. S32–S35, Table S7). Collectively, 365.7 Mbp of nonredundant introgressed DNA, comprising 12.1% of the genome, were found in these 25 individuals. Stratified by archaic origins, we saw that, on average, while the HPRCr1 individuals carry much less (<0.15%), and on average shorter, putative Denisovan DNA than the PNG (>1.34%), the EUR and SAS individuals carry longer Neanderthal segments than the others (Figs. 3B and S36). We also identified genome-wide introgressed SVs across population groups (Figs. S37–S40). As expected, there is a strong linear relationship between the amount of introgressed SV allele bases and the introgressed fraction among the samples (p=5.3×10^−8^, generalized linear model, chi-squared test, d.f.=1; Fig. S41). Across continental groups, on average, introgressed SVs affect 0.6 to 2.4 Mbp per individual, with the highest in PNG (mean: 1,909 SV alleles) and lowest in admixed Americans (mean: 769 SV alleles) (Figs. S42–S45). Across samples, we found that the amount of introgressed insertion bases is notably greater, more than threefold that of deletion bases in some cases (Figs. S41). This aligns with the general trend of more insertions than deletions observed in assemblies relative to GRCh38, likely due to reference bias, which favors the discovery of insertion variants over deletions (46). Regardless, compared to others, the PNG samples show higher levels of both total introgressed SV bases and the ratio of introgressed insertion bases to deletion bases (Fig. S41).

Across all assemblies, 47% of the introgressed SVs located within less than 1 kbp of genes (Fig. 3D). For example, in the PNG samples, 1,679 introgressed SVs are located within less than 1 kbp of genes (RefSeq release 109, Table S6), with an odds ratio of 1.09 for significant genic enrichment of introgressed SVs (p=0.0056, one-sided Fisher’s exact test; 95% C.I.: 1.04–1.15). It is worth noting that we observed enrichment of introgressed SVs in genes in each continental group, suggesting a potentially functional role of these introgressed SVs. We also found overrepresentations in multiple gene ontology (GO) biological processes, such as negative chemotaxis (GO:0050919, 4.53-fold enrichment, Bonferroni’s p=4.5×10^−5^, Fisher’s exact test), calcium ion transmembrane transport (GO:0070588, 2.15-fold enrichment, Bonferroni’s p=6.7×10^−3^, Fisher’s exact test) and neuron projection guidance (GO:0097485, 2.4-fold enrichment, Bonferroni’s p=6.1×10^−6^, Fisher’s exact test), suggesting broad functional implications of introgressed SVs (Table S8).

We observed several previously reported introgressed SVs, such as a Neanderthal-introgressed compound SV with a 4 kbp deletion and a 31 kbp duplication at the *TNFRSF10D* locus (14) and an introgressed variable number tandem repeat (VNTR) haplotype at the *MUC19* locus (47) (Fig. S46) with an uncertain archaic origin. Of note, we identified multiple novel introgressed SVs; for example, compared to the non-introgressed haplotype, all three Neanderthal-like PNG haplotypes at the *TPPP*/*CEP72* locus encompass the cluster of PNG-specific insertions among the assemblies discussed earlier (Figs. 2D and S16). Our sequence analysis suggests that this cluster of insertions represents differences in VNTRs at this locus between modern humans and Neanderthals. We also observed a 52 kbp deletion associated with a Neanderthal-like haplotype, which effectively removes both *GOLGA8K* and *ULK4P1* from the distal site of the chromosome 15q13.3 deletion region (Fig. S22). While the syndrome is rarely involved with this distal site (48), because *GOLGA* paralogs are known to mediate pathogenic microduplications and deletions at 15q11–13 (30), this 52 kbp deletion could affect the susceptibility to genomic instability at this locus.

### Adaptive introgression in PNG genomes.

The high-quality comprehensive SV introgression map that we generated above makes it possible to further study the evolutionary significance of introgression. We hypothesized that introgressed SVs that rose to high frequencies in the PNG could be the targets of positive selection for local adaptations (49). Using the haplotype-resolved PNG and HPRCr1 assemblies, we built an augmented pangenome reference that facilitates comprehensive genotyping of genetic variants, including SVs, across large short-read sequencing cohorts worldwide (Methods). To maximize the genotyping accuracy and sensitivity, we opted to genotype using a pangenome graph of unmerged variants from the PNG and HPRCr1 assembly call sets, including 28,092,023 SNVs, 8,841,949 short indels, and 613,375 SVs (Methods). We genotyped these variants in 71 published high-coverage PNG short-read genomes (>20×) (26, 50), along with 703 African and 585 East Asian samples from the high-coverage 1KG data set (27) and the four high-coverage archaic genomes. Our QC analysis showed an overall 99.1% genome-wide concordance rate for SNVs across samples (range: 98.5–99.3%, Fig. S47). Among SVs, the per-sample concordance rates for insertions are highly compatible regardless of size (range: 96.9–98.7%); in contrast, we observed a negative relationship between concordance rate and size for deletions (Fig. S47). Across the genome, discordant genotypes tend to occur in subtelomeric and centromeric regions, indicating a limited ability to accurately genotype highly repetitive variants, such as VNTRs (Fig. S48) (18). We conservatively excluded discordant variants, those in challenging regions, and those with missing genotypes, leaving 19,222,414 SNVs, 5,345,942 indels, and 199,917 SVs for subsequent analyses.

To detect signals of selection and introgression, we applied population genetic summary statistics and assigned empirical p-values by comparison to 10,000 whole-genome coalescent simulations under the inferred Papuan demographic models and accounting for linkage disequilibrium (Methods). Windows with p < 0.05 were prioritized as candidates for downstream analyses of selection and introgression. Overall, we identified 54 regions across the genome that show significant signals of positive selection (Fig. S49, Table S9; length range: 5,134–95,149 bp), of which 18 overlap with exonic sequences and also show evidence of archaic origins (Table S9). Variations in several of these genes have been shown to associate with immunity (*CD53*), digestion of lipids (*MALRD1*), neurodevelopment (*NECAB1*), psychiatric disorders (*AKAP11*, *DENND1A*), and stroke (*LRCH1*) (51–55). One of the strongest adaptive introgression signals is found at the GBP2/GBP7 (interferon-induced guanylate-binding proteins 2 and 7) locus in the chromosome 1p22.2 region in the PNG (population branch statistic [*PBS*] > 0.75, p < 0.004; introgression statistic *f*_*D*_ > 0.41, p < 0.008). This locus was recently identified as the strongest selection signal in a study using the same PNG short-read genomes (50) and likely associates with immunity against diverse pathogens (56).

To identify adaptive introgressed SV candidates, we searched for SVs that showed significant differentiation in frequency in the PNG cohort compared to the AFR and EAS groups (simulation-based p < 0.05, Methods) and located in genomic regions showing evidence for both selection and introgression based on SNV analysis. Across the genome, 13 regions encompassing 16 SVs showed significant selection signals in the PNG cohort (median *PBS*_*SV*_ = 0.79, p < 0.002; genome-wide top 1% *PBS*_*SV*_ cutoff=0.39, Fig. 4). All but one of these highly differentiated SVs are shorter than 1 kbp in length (median: 319 bp, range: 52–395,265 bp); 14 of these SVs are insertions and 2 are deletions (Table S10). By projecting these selected SVs onto the putatively introgressed loci, we find that two of the selected regions are significantly associated with a Denisovan background, while three are linked to a Neanderthal background (p-value of *f*_*D*_ < 0.034).

Our analysis revealed a putative Denisovan insertion allele (chr19:51484682) at an introgressed locus (chr19:51343160–51528394) on the chromosome 19q13.41 region (Fig. 5A). This 61 bp insertion shows an exceptionally higher frequency in the PNG cohort than other groups (68% in the PNG vs. 13% in other groups) and is located in the 4th intron of the gene *CEACAM18* and 6,545 bp away from the transcription stop site of *SIGLEC12*. *CEACAM18* is a member of the *CEACAM* gene family that serves as receptors for bacterial pathogens, such as *Haemophilus* and *Neisseria* (50). The absence of other highly differentiated nonsynonymous SNVs around this locus suggests that the insertion allele is a likely target for selection. Our inference using pairwise coalescent decoding (Methods) and SNVs in strong linkage to the insertion allele (e.g., chr19:51484850, *r*^*2*^ > 0.96, *D′* = 1) suggests a rather deep divergence (mean: 1.4 million years ago [mya], range:0.99–2.77 mya) from other haplotypes. Pairwise coalescent decoding also showed an enrichment of time to most recent common ancestor (TMRCA) consistent with selective sweeps (Fig. 5B). We found significant evidence for strong selection associated with the insertion-carrying haplotype (s = 0.008, log-likelihood ratio = 6.8468, p = 0.0088, chi-squared test with d.f. = 1). The allele frequency trajectory suggests that the selected variant may have been present at an intermediate frequency (~0.2) in the population prior to its recent increase (Fig. 5B). Consistent with this observation, our analysis of multiple selection episodes indicates stronger support for a selective sweep occurring within the last 100 generations (s = 0.0125, log-likelihood ratio = 10.84, p = 0.0126, chi-squared test with d.f. = 3). *CEACAM18* is exclusively expressed in the small intestine (terminal ileum), and the insertion linked SNVs are also eQTL associated with increasing the expression of this gene (p<9.4×10^−21^, normalized effect size=0.52, GTEx portal) but not associated with the expression of *SIGLEC12*. Given that this gene belongs to the immunoglobulin superfamily, which exhibits evidence of recurrent natural selection on protein surfaces targeted by bacteria in primates (57), we hypothesize that the intron insertion allele is likely involved in a pathogen-driven evolutionary process in the PNG population. Of note, while all three Neanderthal SV candidates are intergenic, a 188 bp insertion is located within 10 kbp upstream of the transcription start site of *PTPRH* (receptor-type tyrosine-protein phosphatase H), a gene recently implicated in regulating intestinal immunity through the dephosphorylation of *CEACAM20*, another member of the *CEACAM* gene family (58). Given that the insertion site is within regions displaying strong H3K27ac signals (UCSC Genome Browser, chr19:55218485–55218485), it suggests a potential regulatory role in gene expression. Taken together, our findings support the notion that archaic introgression has contributed to immune adaptations in modern human populations.

The largest introgressed SV found in these PNG assemblies remains a 395 kbp PNG-specific insertion allele at the chromosome 16p11.2 locus (Fig. 5C) and was previously reported (14). Found in three of the four PNG assemblies (Fig. S50), this insertion allele has a frequency of 62.6% in the PNG cohort and is absent in all other samples. We estimated that the insertion haplotype (chr16:29425843–29825843) diverged from the others about 596 thousand years ago (kya; range: 322–870 kya), consistent with its Denisovan origin (14). The fully phased haplotypes allow us to accurately decode pairs of first coalescence among haplotypes to estimate the local states of TMRCA (Methods). Between the insertion haplotypes, we observed low TMRCA estimates (<120 kya, Fig. 5D) around the insertion site (chr16:29628407) compared with the flanking sequences but did not find such signals in other populations (Fig. S51). The estimated low TMRCA among insertion-carrying haplotypes aligns with the timing of the inferred introgression event (60–170 kya) (14) and is indicative of a clear pattern of a selective sweep—a recent burst of coalescence among the derived lineages (59). This insertion encompasses the gene *NPIPB16*, where multiple amino acid substitutions are likely targets of selection (14).

To infer the time and strength of selection (Methods), we used SNVs in strong linkage to the insertion allele (e.g., chr16:29625843 and chr16:29626149, *r*^*2*^ > 0.41, *D′* > 0.86) and found significant evidence for strong selection (s = 0.005, log-likelihood ratio = 5.16, p = 0.023, chi-squared test with d.f. = 1, Fig. 5D). The inferred allele trajectory suggests that the selected introgressed variant segregated at low frequencies (<0.05) for most of its time in the population and only raised to high frequencies starting ~200 generations ago. In addition, we explicitly tested a model of multiple selection events and found stronger evidence of selection with a selection coefficient of 0.016 starting at 200 generations ago and changed to 0.005 <100 generations ago (log-likelihood ratio = 18.47, p = 0.0003, chi-squared test with d.f. = 3). Our inference suggests that this insertion allele has remained nearly neutral (s < 0.0028) and at low frequencies for an extended period since its introgression into the PNG population until strong selection began approximately 5,800 years ago (Fig. 5D), coinciding with the emergence of agriculture in the region during the mid-Holocene (60). While the phenotypic outcome of this selection is unclear, the *NPIP* gene family has been hypothesized in the involvement in innate antiviral responses (61) and/or related to other immune- or autoimmune-related functions with evidence of ancient and ongoing positive selection in the human lineage (62).

### Potential centromere introgression from archaic hominins in the PNG genomes.

The high-quality PNG haplotype assemblies provide a unique opportunity to test the hypothesis of centromere introgression from archaic hominins (63). We aligned the haplotype-resolved PNG assemblies to the complete T2T-CHM13v1.1 reference to identify centromere sequences. To ensure quality, we excluded contigs with sequence gaps (Ns), lacking unique sequence anchors, or showing large-scale misassemblies (Methods). After quality control, 59.8% of centromeres (55/92) from PNG15 and PNG16 were considered completely assembled (Table S11). Based on active α-satellite higher-order repeat (HOR) array lengths (Methods), we observed substantial variation in centromere size across chromosomes, ranging from 0.98 to 6.20 Mbp (s.d. 1.34 Mbp; Fig. S52, Table S11), consistent with previous studies (64). No significant correlation was found between centromere and chromosome sizes (Fig. S52). In addition, chromosomes 8, 9, 11, 14, and X showed similar centromere sizes, whereas chromosomes 7, 10, 19, 21, and 22 exhibited greater differences among PNG assemblies (Fig. S52).

Because extensive centromeric repeat variation limits the accuracy of sequence alignments and variant calling, we developed an alignment-free, k-mer approach to search for evidence of introgressed centromeres (Methods). Briefly, leveraging the limited presence of archaic DNA in modern-day Africans, we used the high-coverage Neanderthal and Denisovan genomes and identified archaic hominin (ARC)-specific k-mers that are not present in modern-day African samples. Low-frequency k-mers were removed to minimize the impact of sequencing errors (Fig. S53). Our method subsequently recorded the count of ARC-specific k-mers within 2 kbp windows across each individual haplotype assembly. Comparing the top 2% of loci per haplotype from the k-mer analysis with those identified by the three SNV-based methods revealed significant overlap (chi-squared test, *p* < 2.2×10^−^^1^^6^). Overlapping loci also contained significantly more putatively archaic k-mers than nonoverlapping loci (Mann–Whitney U test, *p* < 2.2×10^−^^1^^6^). These results demonstrate that our k-mer–based loci are significantly enriched for putatively introgressed sequences. Among the 55 complete centromeres (Figs. S54–S76, Table S11), 5 and 6 centromeres (mapping to chromosomes 4 [n=1], 5 [n=1], 9 [n=1], 11 [n=3], 17 [n=2], and 22 [n=3]) show evidence for archaic origins in PNG15 and PNG16, respectively (Fig. 6). The strongest signal for centromere introgression occurs on chromosome 4 of PNG16 haplotype 2 (Fig. 6A) and chromosome 22 of PNG15 haplotype 2 (Fig. 6B) as well as PNG16 haplotypes 1 and 2 (Fig. S75). Not only do the ARC-specific k-mer counts exceed that of the genome-wide introgression proportion in PNG16 (4.37%, ARC-specific k-mer threshold = 42), large putatively introgressed segments based on the SNV-based inference are found at both sides of the centromere despite limited availability of unique sequence in the region. It is worth noting that these long archaic haplotypes flanking the putative introgressed centromeres are incompatible with a simple model of incomplete lineage sorting (Table S12) with reasonable demographic parameters (Methods) (15, 29).

To investigate structural differences between putative archaic and modern human centromere sequences, we performed window-based alignments between archaic and modern human centromeres, archaic centromeres, and modern centromeres (Figs. 6C,D and S77–S78). While centromeres with the same ancestry origin show relatively higher sequence identity as expected under identity by descent (Figs. S77–S78, left panels), centromeres with different ancestry origins substantially diverged in both sequence identity and structure (Figs. 6C,D and Figs. S77–S78, right panels). Considering both nucleotide and indel differences, in the α-satellite HORs of chromosome 4, the sequence identity between two modern human sequences (median: 99.8%) is significantly higher than that between putative archaic and modern human sequences (median: 98.8%) (one-sided Mann-Whitney U test, p < 2.2×10^−16^). Similarly, in the α-satellite HORs of chromosome 22, the median sequence identity between two putative archaic centromeric sequences is 99.5%, compared to 98.5% between putative archaic and modern human sequences. In addition, the putative archaic α-satellite HORs on chromosome 4 seem to be expanded from two short α-satellite HOR regions in the modern human counterpart (PNG15 haplotype 1–0000042:138,987,977–139,068,718 and 141,985,570–142,102,299, Fig. 6C). The putative archaic α-satellite HORs on chromosome 22 also primarily share homology with a narrow region in the modern human haplotype (PNG15 haplotype 2–0000147:7,206,683–7,344,983, Fig. 6D). Unlike modern human chromosome 4 centromeric sequences, the archaic haplotype lacks the unique, long stretch of human satellite sequences (Figs. 6A and S57).

To further support the hypothesis of archaic introgressed centromeric sequences in the PNG samples, we constructed phylogenetic trees using sequences orthologous to the flanking introgressed region from all haplotype-resolved assemblies. Our phylogenetic reconstruction shows that the PNG16 haplotype 2 chromosome 4 centromere lineage diverged from the rest of the modern human lineages 489,447–507,131 years ago (95% highest posterior density [HPD] intervals: 397–583 kya for left flanking and 386–633 kya for right flanking, Figs. 6E and S79). In addition, we also observed evidence for archaic origins for the chromosome 22 centromeres of PNG15 haplotype 2, PNG16 haplotype 1, and PNG16 haplotype 2 (Figs. 6F and S75). We noted that in each of these chromosome 22 centromeres only one of the flanking sequences shows introgression signals based on the SNV-based analysis (Fig. S75). The lineage that gave rise to the three PNG haplotypes diverged from other modern human lineages approximately 580,661 years ago (95% HPD interval: 469–694 kya, Figs. 6F and S79). These divergence time estimates are all consistent with the 400,000–700,000 years of modern human–Neanderthal/Denisovan divergence (2, 65), suggesting that these centromeres are likely introgressed from archaic hominins into the early ancestors of the PNG.

Estimating the mutation rate within α-satellite HORs is challenging (64), but the putatively introgressed archaic centromeric sequences offer a valuable recent time point for such estimates. We attempted to use the expansion of modern human α-satellite HORs in the putative archaic centromeric sequences (Fig. 6C,D) and assumed orthologous sequences between archaic and modern humans within these expanded loci. To estimate mutation rates, we applied an evolutionary model (66), assuming a human-archaic divergence of 400–700 kya based on our phylogenetic inferences (Methods). Using this approach, the mean mutation rates of α-satellite HORs for chromosomes 4 and 22 are 2.94×10^−8^ (s.d.: 0.91×10^−8^) and 7.46×10^−8^ (s.d.: 1.89×10^−8^) per allele per generation, respectively (Fig. S80). Compared to the mutation rate for the euchromatic portion of the human genome (67, 68), our estimated mutation rate for α-satellite HORs is up to 5.96-fold higher, reflecting a 35.4% increase from recent estimates (64, 69). While sequence alignments for α-satellite HORs are expected to be biologically meaningful between closely related hominins, we note that these estimates are only approximations, as our approach does not account for the observations of rapid turnover and structural changes in human α-satellite HORs (64). Nevertheless, our discovery of putatively archaic introgressed centromeres in the modern human gene pool, along with direct measurements of α-satellite HORs mutation rates, highlights the complexity of centromere and genome biology and opens new avenues for research in human evolution and genome function.

## DISCUSSION

DNA introgression from closely related species is a widespread evolutionary process that introduces genetic novelty and facilitates local adaptation. Genomic studies have established interbreeding between modern humans and archaic hominins such as Neanderthals and Denisovans, with evidence of adaptive introgression at both SNV and SV levels (10–16, 42, 44, 49, 50). Although SVs have long been recognized as important drivers of phenotypic diversity and human evolution, their complexity and the limitations of short-read sequencing have limited systematic study (12, 14, 16, 18–23, 34), leaving gaps in our understanding of introgressed variation. Here, by integrating long-read haplotype assemblies from two PNG individuals with the 47 high-quality HPRCr1 assemblies, we generated a global map of Neanderthal and Denisovan introgressed sequences, uncovering extensive previously inaccessible variation, particularly SVs. The four newly assembled PNG assemblies are more complete than the HPRCr1 ones, revealing 12% of small variants and 5% of SVs exclusive to these individuals. These PNG-specific variants affect over 12 Mbp of the genome, three times the number of SNVs found in a typical non-African genome. Although SVs are generally depleted in genic regions (14), we identified rare, biomedically relevant variants, including a 0.68 Mbp deletion at the 22q11.2 deletion syndrome locus (17). The 22q11.2 microdeletion syndrome is associated with learning disabilities and congenital heart defects. About 90% of affected individuals carry a ~3 Mbp de novo deletion resulting from recombination between low-copy repeats (LCR22A-D) (17). The PNG deletion removes LCR22A and potentially reduces genomic instability by lowering the likelihood of LCR-mediated unequal crossing-over. Our findings underscore the value of including diverse populations in genomic studies to uncover variants with potential medical and evolutionary significance.

Our introgression map, derived from only 49 globally diverse samples, shows complex forms of archaic variation that short-read analyses cannot resolve. On average, introgressed SV alleles affect 0.7–2.1 Mbp per individual, with the highest levels in Papuans and the lowest in admixed American groups. Unexpectedly, PNG individuals carry more Neanderthal than Denisovan sequences, despite genome-wide estimates suggesting the opposite (5, 7, 26). This discrepancy may reflect differences between the sequenced Denisovan genome and the ancestral population that admixed with Papuans or the fact that our PNG samples are lowlanders, who carry less Denisovan DNA than highlanders (44). A larger cohort including highlanders and other regional groups in the future would provide a more complete view of SV introgression and the genomic legacy of extinct hominins.

Our analysis of adaptive SV introgression highlights the continued role of archaic variation in human immunity (11, 13, 44, 49, 50). Our top Denisovan-introgressed SV, a 61 bp insertion, is involved in an immune-related gene *CEACAM18*, while the 188 bp Neanderthal-introgressed insertion distal to *PTPRH* could impact the function of the gene in intestinal immunity (58). In addition, one of the non-SV adaptive introgressed signals lies within the guanylate-binding protein (GBP) gene cluster, which plays a key role in immune response and has been previously identified as a target of positive selection in PNG (44, 50). These further support the contribution of archaic alleles, particularly Denisovan, to immune adaptation in the region. The estimated onset of selection in the mid-Holocene coincides with the rise of agriculture (~5,000 years ago) and associated cultural shifts, including animal domestication and regional population movements across Papua New Guinea and the Wallacean Islands (60, 70). These activities likely increased pathogen transmission, driving immune-related adaptation beyond the effects of agriculture alone.

Consistent with previous reports (63, 64), we observed substantial centromeric variation in α-satellite HOR structure and putative kinetochore attachment sites. Given the importance of centromere integrity for chromosome stability (71, 72), our discovery of introgressed centromeres in Papuans raises intriguing questions about their persistence in modern humans. Suppressed recombination and reduced selection due to a reduction of local effective population size in these regions may allow archaic centromeric variants to persist, providing unique glimpses into the structure and organization of ancient genomes. Although ILS or convergent evolution represent potential alternatives to centromere introgression, our analyses indicate that the substantial physical length of the identified archaic-like centromeres provides a decisive diagnostic feature, strongly favoring recent introgression (~50 kya) over substantially older ILS (~550 kya) or independent convergent mutation. These results demonstrate that phased long-read assemblies can recover introgressed centromeres, offering a new avenue to explore their structure, function, and role in human evolution.

## Materials and Methods

### Ethics approval and PNG sample collection

The two samples, PNG15 and PNG16, were collected by Dr. Christopher Kinipi, Director of Health Services, at the University of Papua New Guinea (Port Moresby, Papua New Guinea) with ethics approvals obtained from the Medical Research Advisory Committee of the National Department of Health of the Government of Papua New Guinea (permit number MRAC 16.21), the University of Melbourne’s Human Research Ethics Committee (approvals 1851585.1 and 26981), and by the French Ethics Committees (Committees of Protection of Persons 25/21_3, n◦SI:21.01.21.42754). Research visas in PNG were granted by the National Research Institute (visa n°99902292358) with full support from the School of Humanities and Social Sciences, University of Papua New Guinea. After a full presentation of the project to a wide audience, a discussion with each individual willing to participate ensured that the project was fully understood. Both individuals gave their full informed written consent to participate in the study. A material transfer agreement between the University of Melbourne and University of Washington was implemented to share the two LCLs used in the plasmid reporter experiments from the two donors sampled.

### Genomic sequencing production for the PNG samples

All PNG data generated in the study are available on the European Genome-Phenome Archive (EGAS50000001105). Briefly, we generated PacBio HiFi, UL-ONT, Illumina, and Arima Hi-C sequencing data for both PNG15 and PNG16. PacBio HiFi data were generated using HMW DNA extracted from the two LCLs on the Sequel II platform on four SMRT Cells 8M, each with 2-hour pre-extension and 30-hour movies, aiming for a minimum estimated coverage of 30× in PacBio HiFi reads, assuming a genome size of 3.1 Gbp. Circular consensus sequencing (CCS) was calculated using SMRT Link v8.0. UL-ONT libraries were generated using a modified fragmentase protocol and sequenced on R9.4.1 flow cells on a PromethION instrument, with two nuclease washes and reloads after 24 and 48 hours of sequencing. Hi-C data for these LCLs were generated using Arima Hi-C kits, followed by sequencing on an Illumina NovaSeq 6000. Illumina whole-genome sequencing data was generated by the Northwest Genomics Center using the PCR-free TruSeq library prep kit and sequenced to approximately 30× on the NovaSeq 6000 with paired-end 150 bp reads. For downstream analysis, we also included BAM and/or Fastq files for the high-coverage genomes from the 1000 Genome Project (27) and the three published Neanderthal and Denisovan genomes (1–4).

### PNG assembly production and QC

We produced fully phased assemblies using Verkko (v1.4.1) as our primary assembler, which uses rukki (v0.3.0) to leverage information from Hi-C data for extended phasing and supplies with UL-ONT reads for resolving loops and tangles in assembly graphs to achieve high assembly contig contiguity (28). All assemblies were annotated for potential assembly errors using Flagger (19) (v0.4.0) and NucFreq (73) (commit #bd080aa). Briefly, Flagger utilizes read coverage to fit a mixture model per window and infer regions with assembly errors. PacBio HiFi reads were aligned to its diploid assembly using default parameters on Meryl (74) (v1.4.1, k-mer size of 15) and Winnowmap2 (75) (v2.03, --eqx -map-pb -Y -L -y -I8g -p0.5), followed by secphase relocalizing misaligned reads to the correct haplotype. The main Flagger workflow used the author-provided T2T-CHM13v1.1 reference files for coverage biases, such as centromere satellite repeats and SDs, and annotated sequences as haploid (correctly assembled), duplicated, collapsed, erroneous, or unknown across the assemblies. NucFreq was performed for each assembly with default settings. Assembly quality was assessed by computing QV estimates with Merqury (74) (v1.6) as described previously. Gene completeness of each assembly was evaluated using compleasm (76) (v0.2.2) and the primate set of known single-copy genes of OrthoDB (v10, n=13,780 genes). OrthoDB IDs were mapped to human NCBI Protein Accession IDs (https://busco-data.ezlab.org/v5/data/info_mappings_all_busco_datasets_odb10.txt). NCBI IDs were uploaded to UniProtKB (https://www.uniprot.org/id-mapping) to obtain curated proteins and chromosomal locations. Note that because PNG16 is a male individual, the relatively high fraction of missing genes (3.13%) in PNG16 haplotype 1 assembly can be mainly explained by the lack of chromosome X, which is placed in PNG16 haplotype 2 assembly.

### Variant calling

For assembly-based call sets, we used PAV (18) (v1.1.2) with minimap2 (77) (v2.24) and LRA (v1.3.1, https://github.com/ChaissonLab/LRA) alignments against GRCh38-noALT reference genome. We also ran SVIM-asm (78) (v1.0.3) using the same alignments before any alignment trimming applied in PAV. For read-based variant calling, we ran PBSV (v2.9.0) for PacBio HiFi data and Sniffles2 (79) (v2.2) for both PacBio HiFi and ONT reads against the same reference genome. We used default settings provided by the authors for all variant calling tools. For HPRCr1 cohort (19), we also generated variant calls for the 94 assemblies using PAV with alignments against the same reference genome. While we opted to not merge variants to retain all information for our downstream inferences, for comparisons between different call sets, SV-Pop (18) was used to merge variant calls to generate per-sample support information from all other callers and to compare between call sets from different callers (e.g., PAV vs. PBSV) and cohorts (e.g., HPRC vs. PNG).

### Segmental duplication identification

We used the WGAC (80) approach that performs an all-by-all comparison of assembled genomic sequence for the PNG, HPRC, and T2T-CHM13v1.1 assemblies. Briefly, WGAC breaks each assembly into 400 kbp chunks and masks common repeats (“fuguized”) using RepeatMasker (v4.1.5, https://www.repeatmasker.org/) and Tandem Repeats Finder (v4.10.0) before running alignments with blastall (v2.2.11) and lastz (v1.02) to yield a set of putative alignments. The repeats are then added back in, and the putative alignments are heuristically trimmed and then re-aligned to identify SDs. To classify diverged and syntenic SDs, we aligned each assembly to T2T-CHM13v1.1 and retained sequences that are syntenic on the basis of an unambiguous one-to-one correspondence in alignments with ≥1 Mbp of aligned sequence. We determined an SD sequence to be diverged from the T2T-CHM13v1.1 reference assembly if less than 50% of it overlaps with a reference SD. Similarly, assembly- or cohort-specific SD loci were defined using these syntenic and diverged SDs in the coordinate system of T2T-CHM13v1.1 reference genome.

### Identification of archaic introgressed sequences

We took three approaches to identify putative introgressed sequences from archaic hominins in the PNG and HPRC samples using the assembly-based variant call set. First, we adopted a hidden-Markov model (HMM) from Seguin-Orlando et al. (2014) (81) to identify introgressed segments on individual haplotypes. The HMM has two states representing segments of the haploid genome for the archaic and human origin, respectively, and uses subsequent posterior decoding to infer introgressed segments; however, rather than using predefined parameters, we used the “train” function of hmmix (41) (https://github.com/LauritsSkov/Introgression-detection) to train our model to estimate emission probabilities from the data. We also performed hmmix using the precomputed files provided by hmmix and annotated the archaic segments (41). For the third approach, we used Sprime (5) (https://github.com/browning-lab/sprime), an archaic-reference free method, following the published protocol (82) to detect putative introgressed segments. We used all HPRC African samples as the outgroup to account for ancestral polymorphism within the hominin lineages when applicable. Because we observed a large number of putative introgressed segments with low fractions of allele matching to archaic alleles, we heuristically filter any segments with less than 10% of variants matching either Neanderthal or Denisovan alleles. It is known that there is a substantial heterogeneity across different methods for detecting archaic introgressed segments (43). To balance sensitivity and accuracy (43), we required that an archaic sequence be identified by at least two out of the three methods. To generate consensus calls, we intersected the introgressed segment calls from both approaches with reciprocal segment overlap ≥50% and removed any segment less than 1 kbp in length. Finally, we assigned the origin of each QC-passed segment as Neanderthal or Denisovan if the ratio of Neanderthal alleles to Denisovan alleles is at least 1.5 or vice versa. We assign an unresolved origin to an introgressed segment if neither of the ratios is greater than 1.5. We note that for our population genetics inferences, if applicable, the ancestral state of each allele used was determined using a high-quality chimpanzee assembly (40) as an outgroup, and archaic hominin variants of the Neanderthal and Denisovan genomes in GRCh38 space were generated using snpAD (83) (v0.3.11, https://bioinf.eva.mpg.de/snpAD). We also used a HapMap genetic map downloaded from https://bochet.gcc.biostat.washington.edu/beagle/genetic_maps/ if needed. Regions in the human genome identified as uncallable as part of the PAV variant calling were excluded from downstream analysis. In all analyses, we focused on segments outside known complex regions, such as acrocentric and high-identity SD sequences (>99% sequence identity), to avoid false positives due to inaccurate mapping.

### Pangenome-based genotyping in short-read genomes

To study the evolutionary history and population frequencies of the variants identified in the 98 assemblies, we used PanGenie (84) (v2.3.1, https://github.com/eblerjana/pangenie), a pangenome-based genotyping method, to infer genotypes in a large cohort of short-read samples. First, a pangenome graph of 98 assemblies was built following the procedure provided by the authors and removed variants that are more over 20% genotypes missing. We genotyped variants in 71 published high-coverage PNG short-read genomes (>20×) (26, 50), along with 703 African and 585 East Asian samples from the high-coverage 1000 Genomes Project (1KG) data set (27). Genotyping concordance rate was determined by comparing assembly-based genotypes (ground truth) with the PanGenie-inferred genotypes (imputed) using the same sample. Variants with discordant genotypes in each individual were collected and conservatively removed from downstream analysis.

### Selection scan and coalescent simulation

We computed a variety of summary statistics to detect signals of natural selection and introgression, including population branch statistics (85) (*PBS*), nucleotide diversity (86), *f*_*D*_ (87), *r*^*2*^, and Lewontin’s *D´* (88), for the PNG. Using the PanGenie-inferred genotypes, we first scanned the genome to compute these statistics in windows of 100 SNVs with a sliding size of 50 SNVs. For individual SVs, we evaluated the strength of selection by computing these statistics, except for *f*_*D*_. Variants with any missing genotypes were excluded from downstream analysis. Because these statistics are sensitive to demographic history, we performed a large scale of coalescent simulation using msprime (89) (v0.7.4) based on published population genetics models as published in Stdpopsim (90) (v0.1.2) as well as Hsieh et al. (2019) (14). Model parameters were drawn from estimated confidence intervals when possible to account for model uncertainty as described in Hsieh et al. (2019). We generate 10,000 whole-genome simulations to account for past histories between modern and archaic hominins and among multiple African and non-African populations, using chimpanzee as the outgroup. Note that our simulations carefully match the local mutation rate and recombination rate variation at these regions to avoid possible biases to our selection inferences as described previously (14). Significance for each window is computed as the fraction of simulations that have statistic values greater or equal to the observed ones from the data. We also implemented a procedure to directly search for evidence of recent positive selection in assemblies using pairwise sequentially Markovian coalescent (91, 92) (https://github.com/hsiehphLab/pairwiseCoalDecoding). Briefly, we inferred local genealogical trees that change due to ancestral recombination events and can be probabilistically inferred from the patterns of mutations. Because this model describes the entire distribution of pairwise coalescence times between haplotypes, we can easily estimate the TMRCA across local genealogical trees of a sample. Our procedure leveraged the software package of msmc-tools (https://github.com/stschiff/msmc-tools) for decoding the inferred PSMC model to obtain the posterior probabilities for each time state. To estimate selection coefficients of a mutation, we used CLUES (93) (https://github.com/standard-aaron/clues) and followed the instructions provided by the authors. For each variant, we tested CLUES with three different time spans (in generations): (a) [0, 1000], (b) [0, 500, 1000], and (c) [0, 200, 500, 1000]. We determined the best-fit model using a likelihood ratio test with a specified degree of freedom and reported the inferred selection coefficients. We used the R package irlba (v2.3.3) to calculate principal components with only SNVs with minor allele frequencies >10% and passing linkage disequilibrium pruning (r^2^ > 0.4).

### Identification of archaic introgressed sequences in an assembly

We developed a k-mer-based approach to directly identify archaic introgressed sequences in an assembly (https://github.com/hsiehphLab/ARCkmerFinder) (94). Our approach begins with the generation of k-mer databases for each of the four archaic genomes and also creating a joint k-mer database of African genomes from the 1KG short-read collection using the software package Meryl (74) (v1.4.1, k-mer size =15 bp). Notably, k-mers that have low frequencies are far more likely to be errors. To determine a frequency cutoff for the observed k-mers, we applied a mixture model of negative binomials (95) to the observed k-mer distribution of each short-read genome.

$$y\sim \left(\left(2\left(1-d\right)\left(1-{\left(1-r\right)}^{k}\right)+2d{\left(1-{\left(1-r\right)}^{k}\right)}^{2}+2d{\left(1-r\right)}^{k}\left(1-{\left(1-r\right)}^{k}\right)\right)\cdot \text{dnbinom}\left(x,\frac{\text{kmercoverage}}{\text{bias}},\text{kmercoverage}\right)\cdot \text{length}+\left(\left(1-d\right){\left(1-r\right)}^{k}+d{\left(1-{\left(1-r\right)}^{k}\right)}^{2}\right)\cdot \text{dnbinom}\left(x,\frac{2\cdot \text{kmercoverage}}{\text{bias}},2\cdot \text{kmercoverage}\right)\cdot \text{length}+\left(2d{\left(1-r\right)}^{k}\left(1-{\left(1-r\right)}^{k}\right)\right)\cdot \text{dnbinom}\left(x,\frac{3\cdot \text{kmercoverage}}{\text{bias}},3\cdot \text{kmercoverage}\right)\cdot \text{length}+\left(d({1-r)}^{2k}\right)\cdot \text{dnbinom}\left(x,\frac{4\cdot \text{kmercoverage}}{\text{bias}},4\cdot \text{kmercoverage}\right)\cdot \text{length}\right),$$

where k is the k-mer length (k=21) used for constructing the k-mer database, r is the rate of heterozygosity between chromosomes, d represents the percentage of the genome that is a two-copy repeat, and length indicates the genome size (3 × 10^9^ base pairs). The R function dnbinom is the density of negative binomial with kmercoverage estimated by using the mode of the observed k-mer distribution x and bias = 0.5. This model accounts for differences in heterozygous, homozygous, unique, and duplicated sequences (95). We set the cutoff for each genome by identifying the highest k-mer frequency bin, starting from the lowest, where most k-mers remain unexplained by the model (Fig. S53). Archaic-specific k-mers were identified by subtracting the joint African k-mers from each of the four archaic k-mer databases, leveraging the limited archaic introgression in Africans to minimize the impact of ancestral polymorphisms. It is worth noting that common simple repeat sequences in the lineage of humans are likely removed from the processed archaic k-mer databases through this procedure, making our downstream introgression calling more conservative in repetitive sequences. Given an assembly, we scan contig sequences for the occurrences of archaic-specific k-mers. We smooth out the k-mer signals by applying a sliding window of 2 kbp spanning the assembly to aggregate neighboring information for evidence of archaic sequences. Genome-wide cutoff of k-mer occurrences for archaic introgression were determined by the whole-genome estimation of archaic introgression using the SNV-based inferences as described above. To evaluate the performance of our approach in detecting introgressed sequences, we compared the top 2% of loci per PNG haplotype assembly from the k-mer analysis with those identified by three reference-based SNV methods in the same haplotype. The top 2% threshold was chosen assuming equal amounts of introgressed sequence between the two haploid assemblies of an individual. Loci were considered shared if they overlapped by at least 50% reciprocally. In each assembly, we observed significant overlap between the two call sets (chi-squared test, *p* < 2.2×10^−^^1^^6^). In addition, overlapping loci contained significantly higher counts of putatively archaic k-mers than nonoverlapping loci (Mann–Whitney U test, *p* < 2.2×10^−^^1^^6^), supporting that loci identified by our k-mer–based approach are enriched for introgressed sequences.

### Centromere analysis

We identified centromere sequences by aligning the complete centromeres from T2T-CHM13v1.1 to assemblies using minimap2 (v2.28-r1209) with the parameters “-t 20 -I 15G -x asm20 -s 5000 -K 8G”. We used StainedGlass (96) (v0.6) to visualize the centromere sequence structure in a heatmap using default parameters and applied RepeatMasker (v4.1.5) to annotated repeats within each centromeric sequence. Individual centromeres were determined using the minimum and maximum locations of annotated active α-satellite HOR arrays. To compare centromere sequences, we used the function “slide” in StainedGlass with a sliding size of 1,000 bp. The Methylation analysis (modified-bases 5mCG_5hmCG) was based on ONT sequencing reads basecalled and methylation-called by ONT basecaller Dorado (v0.8.2) with models dna_r9.4.1_e8_hac@v3.3 and dna_r9.4.1_e8_sup@v3.3. The basecalled reads were aligned to the assembly using Winnowmap2 (v2.03) using these parameters “-s 4000 -t 16 -I 10g”. The Winnowmap2 results were run through the CDR-Finder pipeline(97) (https://github.com/EichlerLab/CDR-Finder.git, commit 0248ce66) to identify putative centromere dip regions. To infer phylogeny, we used BEAST (98) setting GAMMA Category Count = 5, shape parameter = 0.1 and Proportion Invariant = 0.38 and used the GTR substitution model (all rates = 1.0) for the Site Model. In addition, we used the Calibrated Yule Model and kept most of the parameters of the priors as default, but set birthRate and clockRate using Gamma(0.001, 1000) with calibrations based on human–chimpanzee and human–gorilla divergence following the distributions of a log-normal(M = 6500000, S = 0.09) and log-normal(M = 10500000, S = 0.09), respectively. Mutation rates were estimated using a customized pipeline (99), which follows the same procedure as Logsdon et al. (2022) (64). We took the best alignment of one-to-one correspondence for individual 20 kbp windows as input from the pairwise centromere sequences analysis of StainedGlass to estimate sequence divergence between archaic and modern human centromeres. Mutation rates were estimated for each 20-kbp sequence using Kimura’s model of neutral evolution (100). To account for parameter uncertainty for mutation rate estimates, we uniformly drew an archaic-modern human divergence time from [400,000, 700,000] years, according to our phylogenetic analysis, and an ancestral population size from [1,000, 30,000] years (1–3). We assumed a generation time of 29 years for our inferences.

## Supplementary Material

Supplementary Text & Figs. S1-S80

Supplementary Tables S1-S12

Supplementary Text

Figs. S1 to S80

Tables S1 to S12

## Figures and Tables

**Fig. 1:**
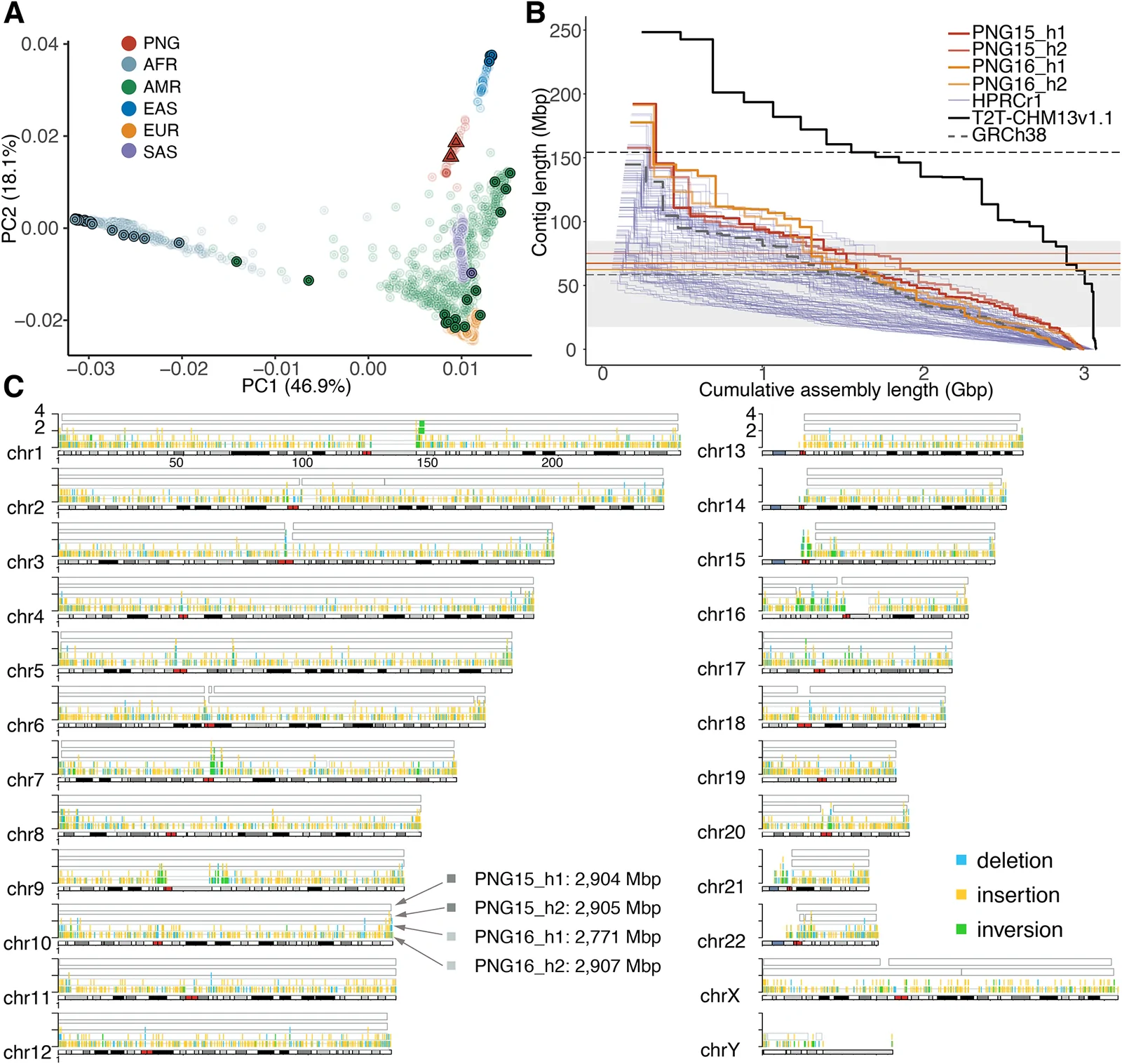
High-quality haplotype-phased diploid assemblies from two PNG individuals. (A) The first two principal components of 71 PNG samples (red), including PNG15 and PNG16 (triangles), along with the 1KG samples. Each point represents a sample, with colors indicating the continent of sampling origin. Bordered symbols indicate HPRC and PNG assemblies. PNG: Papua New Guinea, AFR: African, AMR: admixed American, EAS: East Asian, EUR: European, and SAS: South Asian. (B) Assembly contiguity of the PNG, HPRCr1, GRCh38, and T2T-CHM13v1.1 assemblies. All assemblies are gap (N)-stripped before computation. Red and orange horizontal lines indicate the N50 values for PNG15 and PNG16, respectively. For comparison, black solid and gray dashed lines represent the N50 for T2T-CHM13v1.1 and GRCh38, respectively. The shaded area highlights the range of N50 values for the HPRCr1 assemblies. (C) Completeness of the PNG assemblies is compared with T2T-CHM13v1.1 using contig alignments ≥1 Mbp. The alignment blocks from the top to the bottom are for PNG15_h1, PNG15_h2, PNG16_h1, and PNG16_h2. The histogram within alignment blocks highlights PNG-specific SVs in the assembly discovery set, where the y-axis refers to allele count in the four PNG assemblies and colors indicate SV types.

**Fig. 2:**
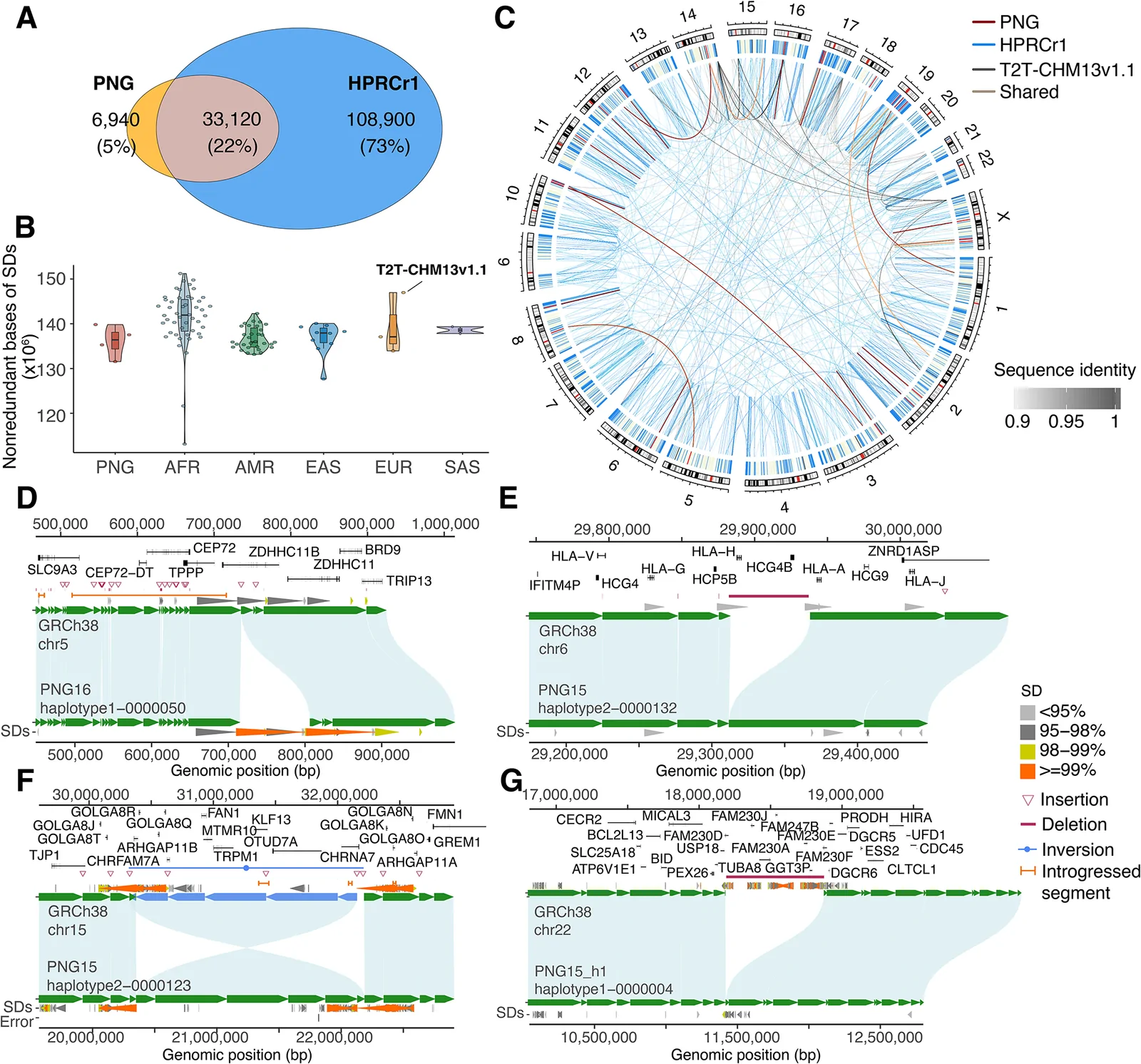
Characterization of genomic variation in the PNG assemblies. (A) Venn diagram showing the number of SVs shared between PNG and HPRCr1 samples, as well as cohort-specific SV calls. SVs overlapping assembly errors were excluded. (B) Distribution of nonredundant bases of SDs by haplotype across continental groups. SDs are collapsed to compute the total nonredundant bases for each haplotype. The number of such bases is highlighted for T2T-CHM13v1.1 for comparison. (C) Circos plot highlighting intrachromosomal and interchromosomal SDs shared between PNG and HPRCr1 (gray), as well as cohort-specific SDs (red: PNG, blue: HPRCr1). SDs unique to T2T-CHM13v1.1 are black. Color intensity indicates sequence identity between SD pairs. (D-G) Alignment plots between the GRCh38 reference genome (top) and PNG haplotypes showing the cluster of insertions at *TPPP*/*CEP72* (D), the 54 kbp deletion at *HLA-H*/*HCG4B* (E), 1.8 Mbp inversion at chr15q11.3 (F), and 0.68 Mbp deletion at chr22q11.2 (G) loci. The gene annotation of the reference genome is shown at the top, followed by SDs predicted by WGAC (Methods).

**Fig. 3:**
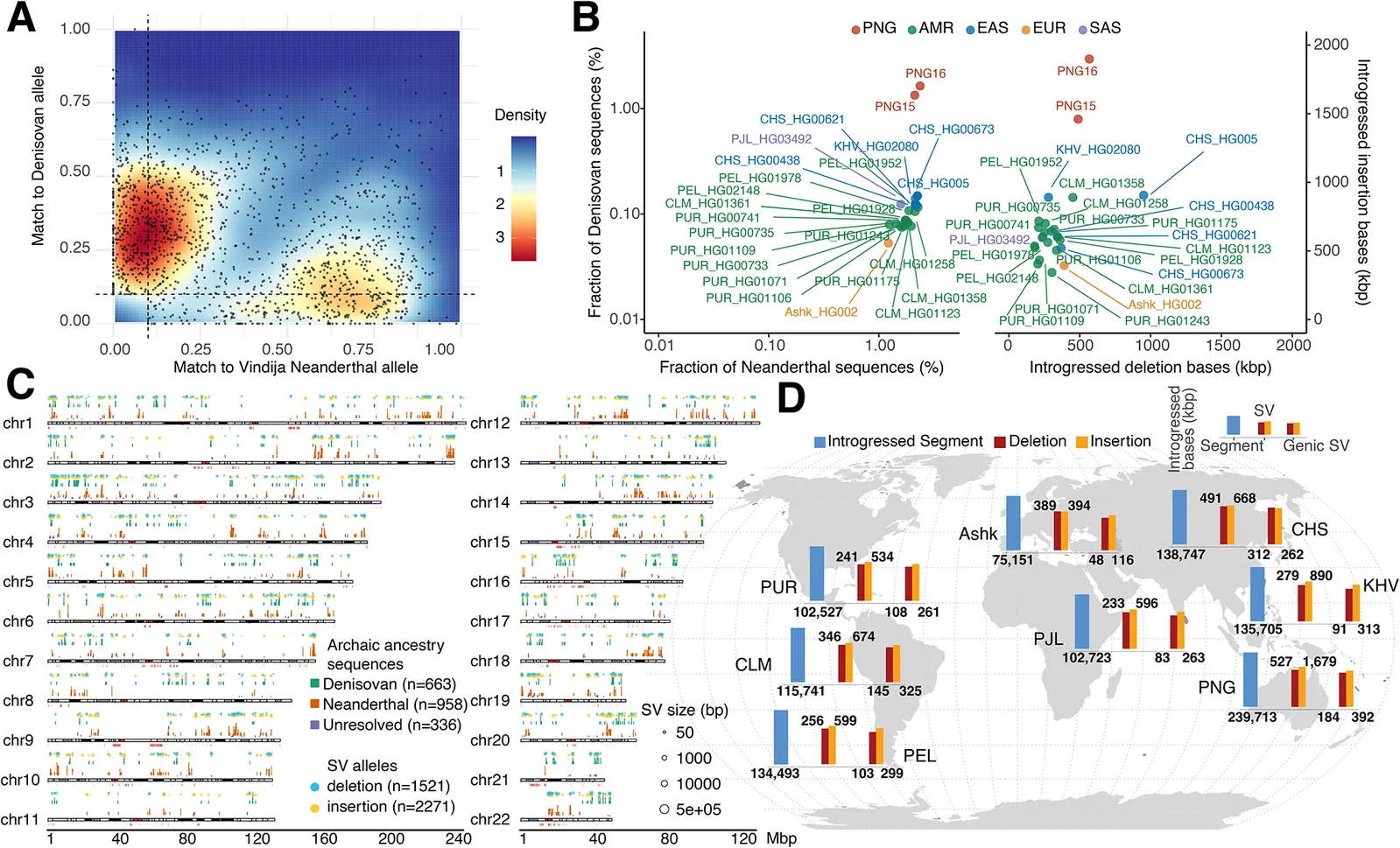
Archaic introgressed sequences and SVs from a diverse population panel of non-African samples in HPRCr1 and PNG cohorts. (A) Density plots of matched proportion to the Vindija Neanderthal and Denisovan genomes in individual introgressed segments (dots). The proportion of putative archaic-specific alleles in each segment was computed for a given archaic genome. Segments with less than 10% match rate on both axes were excluded due to low confidence. Similar plots for other Neanderthal genomes are in Figs. S27–S28. (B) Summary of introgressed sequences and SVs in the PNG and HPRCr1 non-African samples. Left: Fractions of Denisovan (y-axis) and Neanderthal (x-axis) sequences in individual diploid genomes. Right: The amounts of introgressed bases for insertion (y-axis) and deletion (x-axis) alleles in individual diploid genomes. (C) Genome-wide distribution of introgressed segments (vertical bars; green: putative DNS sequences, red: NDL sequences, dark blue: unresolved origin) and SV alleles (circles; blue: deletion, yellow: insertion) in PNG samples. Similar plots for other non-African HPRCr1 samples are in Figs. S37–S40. (D) The mean number of bases for introgressed sequences, introgressed SVs, and introgressed SVs that overlap with genes in individual populations. The numbers are expressed in thousands of base pairs. The height of each bar chart is on a base-10 logarithm scale (kilobase pair, kbp). Ashk: Ashkenazi, CHS: Han Chinese South, CLM: Colombian in Medellin, KHV: Kinh in Ho Chi Minh City, PEL: Peruvian in Lima, PJL: Punjabi in Lahore, PUR: Puerto Rican.

**Fig. 4:**
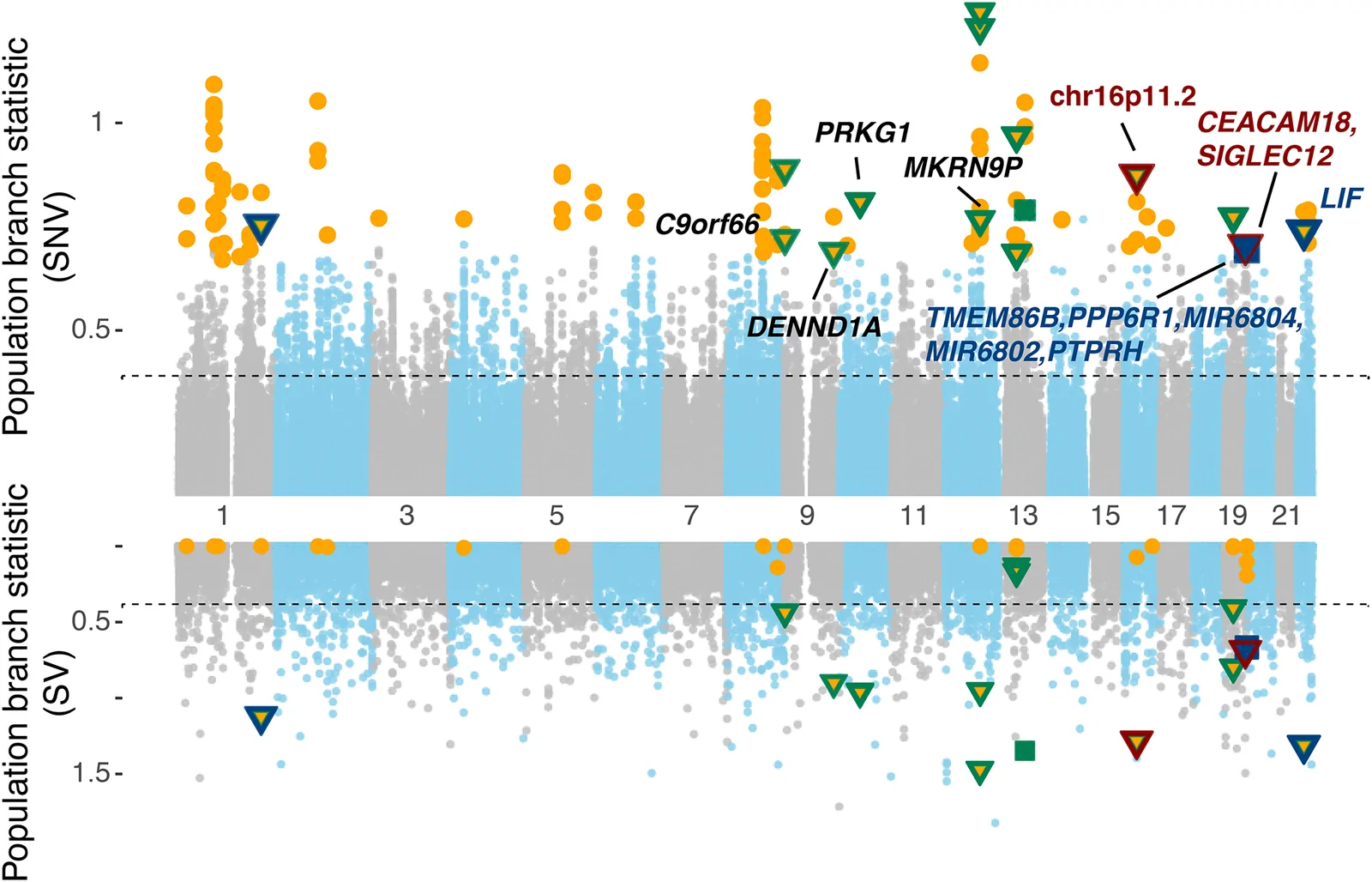
Candidate loci with significant adaptive introgression signals. Top: Genome-wide Manhattan plot (SNVs). Bottom: Hudson (SVs) plot for the adaptive introgression scans in the PNG. The Manhattan plot illustrates the distribution of population branch statistics (PBS) from segments of 100 SNVs, while the Hudson plot depicts the PBS values of individual SVs. PBS values were computed using East Asian and African groups as the sister- and out-groups, respectively. Orange circles represent segments showing significant PBS values (p-value<0.05, simulations based on 10,000 demographic models). Green symbols indicate significant test statistics for SVs (p < 0.05). Red and blue symbols represent putatively introgressed SVs using the Denisovan and Neanderthal as the archaic reference, respectively. Triangles and squares denote insertion and deletion loci, respectively.

**Fig. 5:**
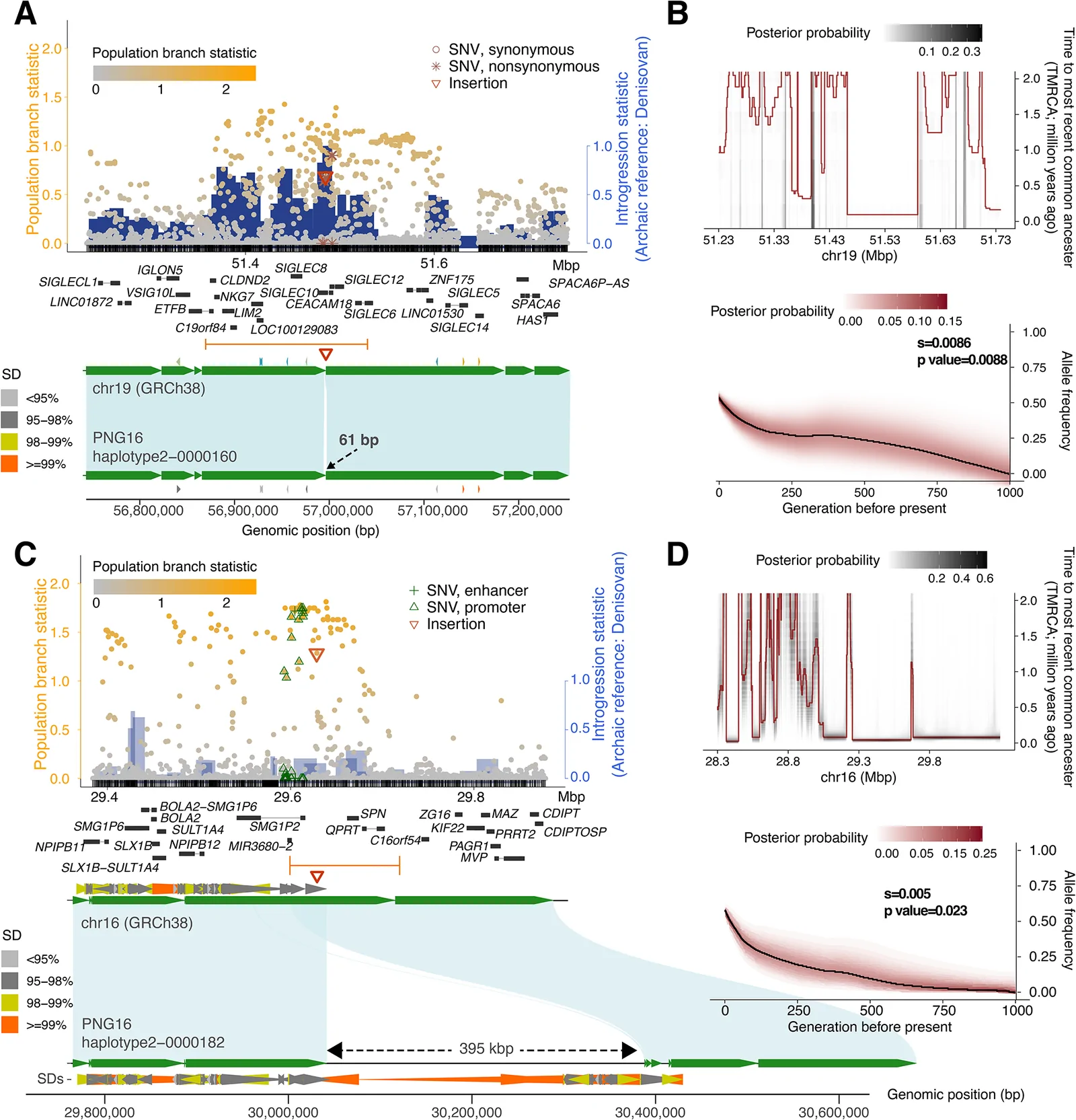
Candidates of adaptive introgressed SVs in the PNG. (A) Distributions of population branch statistics (PBS) of SNVs (circles) across the 61 bp insertion locus at chr19:51231472–51741792. Orange gradient of the circles and the blue histogram indicate the strength of the selection (PBS) and introgression (*f*_*D*_, archaic reference: Denisovan). Nonsynonymous, synonymous, promoter, and enhancer SNVs as well as SVs are annotated as symbols (RefSeq and ENCODE elements). The middle panel shows gene annotations in the GRCh38 coordinates. Introgressed segments and SVs are denoted as orange segments and red triangles, respectively. Bottom: green ribbon depicts syntenic alignments between GRCh38 and PNG16 haplotype 2 contig, while green arrows indicate sequence orientations. (B) Top: evidence for positive selection using pairwise coalescent decoding between homologous introgressed sequences (Methods). The heatmap shows the posterior probability distribution of piecewise time to most recent common ancestor (TMRCA). An enrichment for recent coalescence times in the distribution of TMRCAs along the sequence is consistent with positively selected loci. Bottom: the likelihood-based ancestral recombination graph approach (CLUES, Methods) to infer selection coefficient and allele trajectory using SNVs (e.g., chr19:51484850) that are in linkage with the SV candidate. The heatmap shows the posterior probability of the allele trajectory over the past 1,000 generations. The significance of the selection signal is computed using a chi-squared test (log-likelihood: 5.1641, degree of freedom=1). (C) and (D) are similar to (A) and (B), respectively, but for the putative candidate of the 395 kbp insertion sequence at chr16:29362357–29885651.

**Fig. 6:**
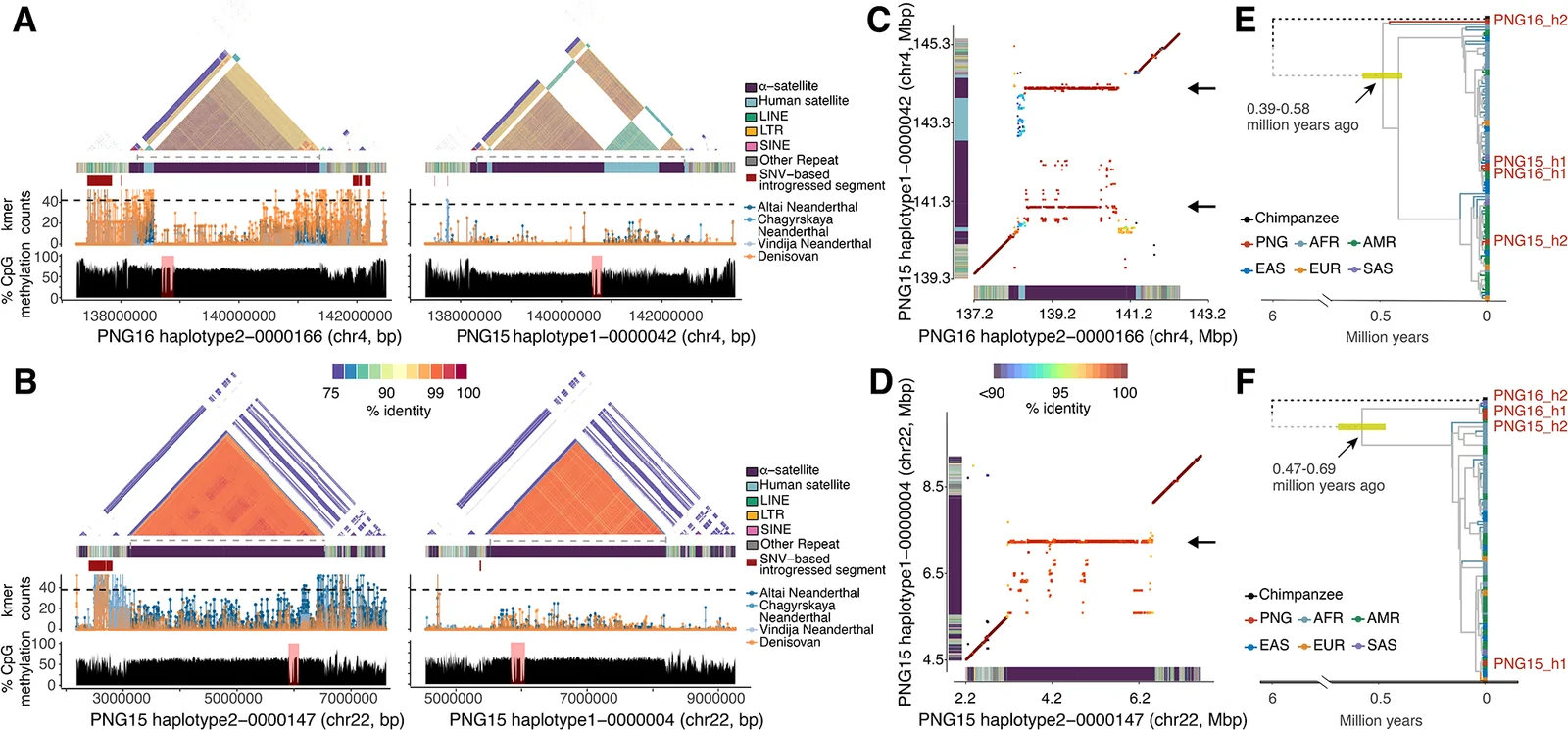
Evidence for archaic introgressed centromeres on chromosomes 4 and 22. (A&B) Comparison between a putative introgressed (left) and non-introgressed (right) centromeres. Top panels: heatmaps of pairwise sequence identity in 5 kbp windows (StainedGlass) across the centromere and flanking regions. Colors of the heatmap indicate sequence identity. The colored horizontal bars show underlying repeat content across the centromere regions. The gray dashed lines indicate the inferred centromere sequence based on annotated active α-satellite arrays. Red rectangles represent SNV-based introgression signals. Middle panels: Distribution of archaic-specific k-mers across 20 kbp windows using four high-quality archaic short-read genomes. The black dashed lines indicate the genome-wide k-mer count cutoffs, 38 and 42, that correspond to the genome-wide archaic introgression fraction for PNG15 and PNG16, respectively. Bottom panels: The percentage of average methylated CpG frequency across 5 kbp bins across the region. The pink rectangles indicate the putative centromeric hypomethylated loci associated with kinetochore attachment. (C&D) Allelic variation between the putative archaic and modern human centromeric haplotypes. 20 kbp windows with a 1 kbp sliding step from the query sequence (horizontal axis) were aligned to the target sequence (vertical axis). Only the best alignments, based on sequence identity, were retained. Colors of dots indicate percent sequence identity. The α-satellite and other human satellite array structures are shown on the axes as in (A&B). The black arrows indicate the modern human α-satellite HORs expanded in the putative archaic centromeric sequences. (E&F) The phylogeny of the putative introgressed sequence from the left flanking of centromere in (A). The ancestral branches leading to the chimpanzee and ancestral lineages of humans are truncated (dashed lines) for illustrative purposes. 95% high posterior density intervals for node ages between 0.2 and 1.0 million years are displayed as horizontal green line.

## Data Availability

The materials of PNG15 and PNG16 are available from Dr. Irene Gallego Romero under a material transfer agreement with the St Vincent’s Institute. All data are available and described in the manuscript or supplementary material. Briefly, all PNG data generated in the study are available on the European Genome-Phenome Archive (EGAS50000001105). BAM and/or Fastq files for the high-coverage short-read genomes from the 1000 Genome Project (27), PNG (26, 44), and the three published Neanderthal and Denisovan genomes (1–4).
