# Supplementary material for: A global map for introgressed structural variation and selection in humans
Source: Science. Author manuscript; Available in PMC 2026 Jul 2. (PMC13322399; doi:10.1126/science.adz7518)
Supplement: Supplementary file 1 [file NIHMS2190982-supplement-Supplementary_Text___Figs__S1-S80.pdf]

Science
AAAS

Supplementary Materials for

A global map for introgressed structural variation and selection in humans

PingHsun Hsieh, Natthapon Soisangwan, David S. Gordon, Athef Javidh, William T. Harvey, David Porubsky, Kendra Hoekzema, Carl Baker, Katherine M. Munson, Christopher Kinipi, Matthew Leavesley, Nicolas Brucato, Murray P. Cox, François-X Ricaut, Irene Gallego Romero, Evan E. Eichler

Correspondence: hsiehph@umn.edu, ee3@uw.edu

The PDF file includes:

Supplementary Text
Figs. S1 to S80

Other Supplementary Materials for this manuscript include the following:

Tables S1 to S12

Table of Contents

Supplementary Text 3

Sequencing and assembly 3

Assembly QC analysis 3

Comparisons of genomic variation between the Papuan and HPRCr1 assemblies 4

Segmental duplication analysis 4

Archaic centromere introgression vs. incomplete lineage sorting 5

Supplementary Figures 6

Supplementary Tables 74

Supplementary Text

Sequencing and assembly

We extracted high-molecular-weight (HMW) DNA from established, quality control (QC)-passed lymphoblastoid cell lines (LCLs) of a female individual from a Western Lowland area in Papua New Guinea (PNG15) and a male individual from the Solomon Islands (PNG16) (**Methods**). High-coverage sequencing data of these two Papua New Guinea (PNG) samples were generated using Illumina short-read sequencing (PNG15: 18×, PNG16: 39×), PacBio high-fidelity (HiFi; PNG15: 31.1×, PNG16: 38.4×), Oxford Nanopore Technologies (ONT; PNG15: 44×, PNG16: 51×), and Arima Hi-C (PNG15: 427 million read pairs, PNG16: 842 million read pairs) (**Methods**).
After standard QC for sequencing reads (**Methods**), we generated four haplotype-phased assemblies (**Table S1**) using Verkko (v1.4.1) (*28*). The four haploid assemblies range from 2.89 to 3.01 billion base pairs (Gbp) in size, mainly due to the size difference in the sex chromosomes (**Table S1**). The N50 values, a measure of assembly contiguity, for the four PNG assemblies are 111–136 million base pairs (Mbp, with scaffolding) or 62.4–74.9 Mbp (without scaffolding), which are higher than both the human reference genome GRCh38 (58.3 Mbp) and Year-1 releases of the Human Pangenome Reference Consortium (HPRCr1; n=47, range of N50: 17.4–84.9 Mbp, median: 40.9 Mbp) (**Fig. 1B**). The average quality value (QV) of these PNG assemblies estimated using k-mers derived from Illumina data is 44.57 (**Table S1**), which corresponds to an error rate of 3.5×10^{-5} per base pair.

Assembly QC analysis

To systematically assess the quality of these PNG assemblies, we took three approaches. First, we adopted the read-based pipeline, Flagger(*19*), to detect possible misassemblies within a phased diploid assembly. Only ≤0.67% (or 19.6 Mbp) of each haploid assembly are marked as errors by Flagger (**Table S2**), which is lower than 58% of the HPRC assemblies. Of these assemblies, the most and least prominent error types are erroneous bases associated with low read coverage (range: 0.15–0.47%) and false duplicated bases (range: 0.002–0.06%), respectively. In addition, we used NucFreq(*73*), a read-depth-based method, to detect misassembled and/or collapsed regions in an assembly (**Methods**; **Table S3**). On average, 0.18% and 0.032% of these PNG assemblies are considered as collapsed (range: 0.126–0.273%) and misassembled (range: 0.024–0.051%) sequences, respectively. Of note, while Flagger and NucFreq together identified 0.65–0.84% of the assemblies as errors, only 0.026–0.034% of the assembled sequences were flagged as errors by both methods (**Figs. S1-S2**). In our third approach, we compared the presence of a set of single-copy orthologs conserved across a wide range of taxa in primates (primates_odb10, 13,870 genes) to assess the completeness of the PNG assemblies using compleasm(*76*). Compatible with the estimate of 99.5% for the telomere-to-telomere (T2T) T2T-CHM13 reference assembly(*76*), the overall completeness of the four PNG assemblies reported by compleasm is >99.3%, except for PNG16 haplotype 1 (95.8%; **Table S4**). Further investigation revealed that ~65% of these missing single-copy genes (336 of 520 genes) are located on the X chromosome, which is not present in this male haploid assembly. The completeness of this assembly increases to 98.3% after accounting for the sex chromosome difference. Considering alignments of phased assemblies to the T2T-CHM13 reference using minimap2 (*24, 77*), 91.3–95.4% (or 2.84–2.97 Gbp) of the phased assemblies have unique alignments with at least ≥1 Mbp (**Fig. 1C**). As expected, alignment gaps are mostly found in acrocentric chromosomes (13, 14, 15, 21 and 22) and some centromere regions, due to the complexity and the repetitive nature of these regions (*24*).

Comparisons of genomic variation between the Papuan and HPRCr1 assemblies

We assessed genomic variation in the four PNG haplotype assemblies relative to the human reference genome GRCh38 using both assembly-based callers PAV (v1.1.2) (*18*) and SVIM-ASM (v1.0.3) (*78*) as well as read-based callers Sniffles2 (v2.2) (*79*) and PBSV (v.2.9.0, **Methods**). Because merging variants, especially for SVs, across call sets remains challenging, we chose PAV calls as the primary call set given its high precision and call rates(*18*) and used other call sets as a secondary support. In addition, we included HPRCr1 PAV call set for comparison in our downstream analysis for comparison. For SV comparisons between PNG and HPRCr1 call sets, SVs of the same type that overlap by at least 50% reciprocally are merged(*18*) and re-genotyped based on supporting evidence (**Methods**). PAV identified 4.59 and 4.25 million (or 4.53 and 4.20 million after QC, **Methods**) SNVs and 1.07 and 0.99 million small insertions and deletions <50 bp (indels) in PNG15 and PNG16, respectively, compatible to the number of small variants found in the HPRCr1 non-African samples (mean SNVs: 4.26–4.40 million, mean indels: 0.97–1.03 million, **Fig. S3**). Removing variants within the erroneous assembled regions in the PNG assemblies results in ~1.3% and ~1.8% decreases in SNVs and small indels, respectively. It is worth noting that compared with the HPRCr1 call set, 12% of the small variants are exclusively found in the two PNG genomes, confirming the presence of unique diversity in the Papuan(*7, 26*).

For large SVs (length ≥50 bp), the suite of callers collectively identified 30,789 and 30,574 insertion (INS), 23,312 and 22,581 deletion (DEL), as well as 175 and 175 inversion (INV) calls in PNG15 and PNG16, respectively. Of the 42,261 insertion and deletion SVs that PAV identified, >89.7% of these SVs (PNG15: 89.7%, PNG16: 90.7%) have at least one secondary support from other callers (**Fig. S4**). Of note, PAV-specific SV calls are significantly longer than those identified by multiple callers (**Fig. S4**, **Table S5**, Mann-Whitney U [MWU] test, $p<1.0\times10^{-7}$ regardless of SV type)—a likely benefit of assembly versus read-based callers. To assess the SV diversity in the PNG samples, we analyzed PAV SV calls from the PNG and HPRCr1 cohorts together and found that 5.6% of the SVs are PNG-specific (**Fig. 2A**). While the fraction of PNG-specific SVs in the two samples is significantly higher than in any pair of East Asian samples (range: 4.7–4.9%), it is comparable to those observed among admixed American samples (range: 5.2–6.6%). Between SV types, 4% and 6% of the insertions and deletions, respectively, are PNG-specific (**Fig. S5**). We noticed a much lower support rate for inversions (<15%) by different callers and that over 90% of inversions are sample- and/or cohort-specific (PNG vs. HPRCr1), highlighting the difficulty in calling inversions. Because of the unusually high population specificity and the overall lower support for inversions, unless mentioned otherwise, we focus on insertions and deletions for most of the downstream analysis. Similar to previous studies(*18, 19*), we found an inverse relationship between SV length and abundance in the PNG genomes; e.g., 97.6% of SVs are shorter than 10 thousand base pairs (kbp) regardless of SV type (**Fig. S6**). Overall, the PNG-specific insertions and deletions account for 6.8 and 8.6 Mbp, respectively, in the human genome. Among PNG-specific SVs, while 31% (n=1,094 out of 3,568**;** 12 exon-overlapped SVs) and 32% (n=1,082 out of 3,372; 57 exon-overlapped SVs) of the insertions and deletions overlap with genes (i.e., introns and/or exons), respectively, our analysis shows a significant depletion of these SVs in genes (p<0.001; 1,000 permutations). These results are consistent with the expectation of selection against large SVs in functionally constrained sequences(*18*).

Segmental duplication analysis

The high-quality haplotype-phased assemblies also provide a powerful means to assess the diversity and variation of segmental duplications (SDs)**—** highly-identical (>90% sequence identity) DNA duplications that are ≥1 kbp in length and dispersed both interchromosomally and intrachromosomally, in the human genome. We applied whole-genome assembly comparison (WGAC) (*80*) to the PNG and HPRC haplotype assemblies, along with the complete human reference genome, T2T-CHM13, to detect SDs (**Methods**). We limited our SD analysis to contigs with length ≥1 Mbp to reduce potential false discoveries(*23*) and removed SDs overlapping with assembly errors. On average, we identified 157 Mbp

of nonredundant SD sequences (range: 125–173 Mbp, s.d.: 8.34 Mbp) in the PNG and HPRC assemblies, compared with the 182.9 Mbp of nonredundant SDs found in T2T-CHM13. We noted that the difference in nonredundant SD sequences between T2T-CHM13 and the PNG/HPRC cohorts is diminished after excluding SDs present within acrocentric/centromeric sequences (**Figs. 2C** and **S6**). Considering SD pairs, on average we observed a ratio of 1.9:1 between interchromosomal SD pairs (mean: 10,734, s.d.: 682 bp) and intrachromosomal SD pairs (mean: 5,636, s.d.: 322 bp; **Figs. 2C** and **S11**). Consistent with previous studies(*22*), intrachromosomal SDs show significantly higher sequence identity (mean: 94.1%, s.d.: 0.07%) and greater length (mean: 14,083 bp, s.d.: 719) than interchromosomal SDs (identity: 93.2%, s.d.: 0.06%; length: 8,428 bp, s.d.: 576; $p < 2.2\times10^{-16}$ for both comparisons, one-sided MWU tests; **Figs. S12-S13**). In PNG and HPRCr1 assemblies, 85% and 91% of private SD pairs are intrachromosomal, respectively (**Fig. 2C**), and show significantly higher sequence identity than shared pairs (MWU test, $p < 5\times10^{-5}$; **Fig. S14**).

Archaic centromere introgression vs. incomplete lineage sorting

We explicitly evaluated whether the archaic centromere signals observed in the PNG assemblies can be explained by incomplete lineage sorting (ILS). Following a simple model of ILS (*15*), the expected length L_{exp} of a shared sequence between two populations separated by t generations is $1/(r \times t)$, where r is the locus recombination rate per base per generation. Because the length distribution of the shared sequence evolved in the two lineages is a sum of two exponential distributions, it follows a Gamma distribution with shape parameter 2 and rate parameter $1/L_{exp}$. Thus, the probability of sharing a sequence of L_{obv} due to ILS is

$$1 - CDF\{Gamma\left(L_{obv}, shape = 2,\ rate = \frac{1}{L_{exp}}\right)\}$$

Recombination rates were interpolated from the HapMap genetic map (https://bochet.gcc.biostat.washington.edu/beagle/genetic_maps/). Due to the lack of reliable recombination measurements within centromeric cores, we tested our hypothesis using unique flanking sequences that show archaic introgression signals based on SNV-based inference (**Figs. 6A&B**). The estimated recombination rates of these five flanking sequences (448-663 kbp) are between 0.08×10^{-8} and 0.17×10^{-8} per base pair per generation, which are nearly two orders of magnitude lower than the genome-wide average (1.00×10^{-8}). Assuming a human–Neanderthal divergence of 550,000 years ago (*2*) and the estimated time of archaic introgression at 50,000 years ago, the expected sequence length due to ancestral polymorphism ranges from 16,246 and 34,523 base pairs. Our results suggest that these introgressed sequences are significantly longer than those persisting from the human-archaic ancestor ($p < 6.5\times10^{-6}$, **Table S12**). Similarly, while convergent evolution can produce identical mutations under shared selective pressures, it is highly unlikely to generate the contiguous, high-identity haplotypes spanning hundreds of kilobases observed in our data.

Supplementary Figures

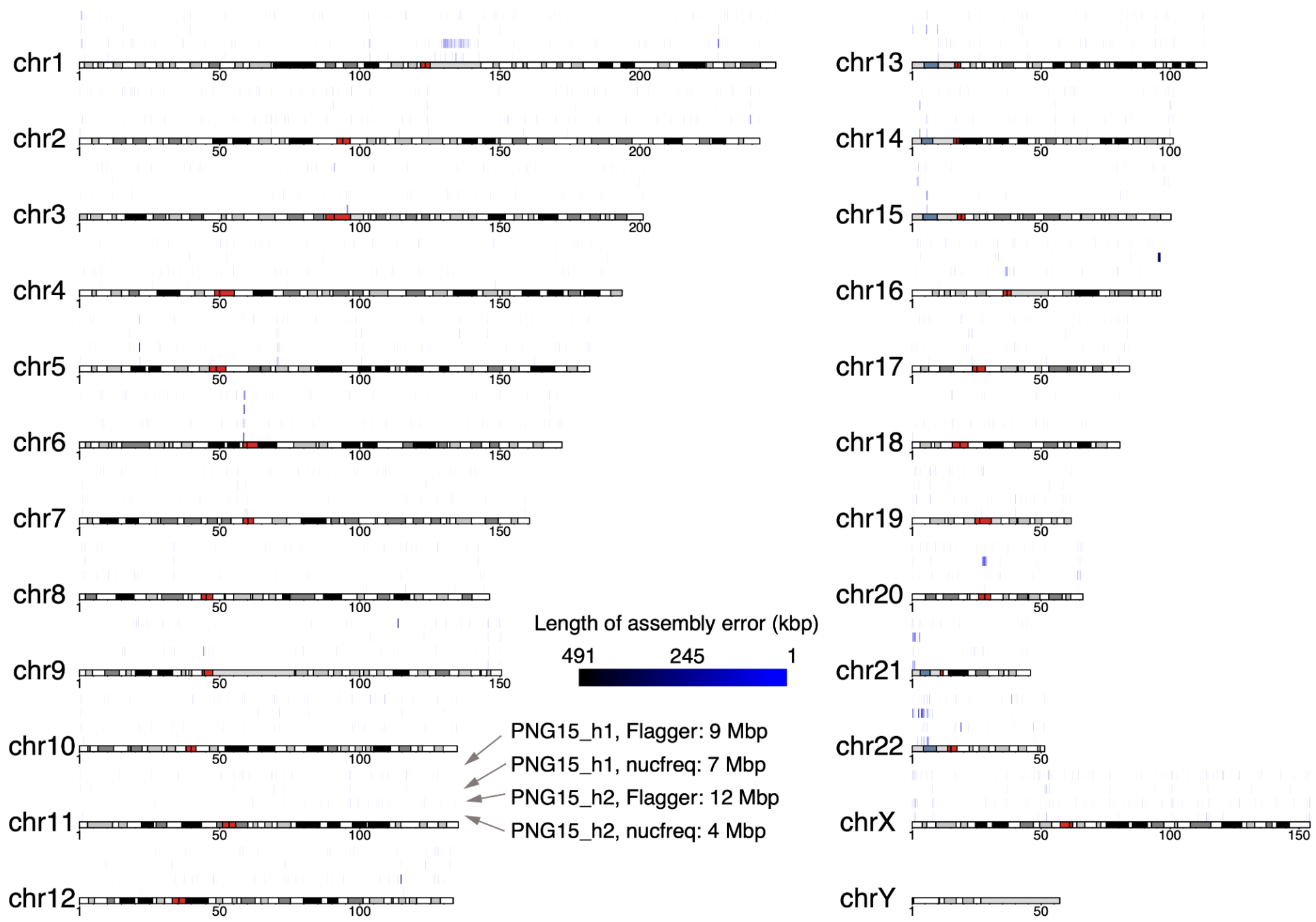

Fig. S1. Genome-wide distribution of assembly errors in PNG15 haplotype assemblies detected using Flagger as well as NucFreq. Vertical bars in each track show erroneous assembly loci, with color intensity indicating the size of errors.

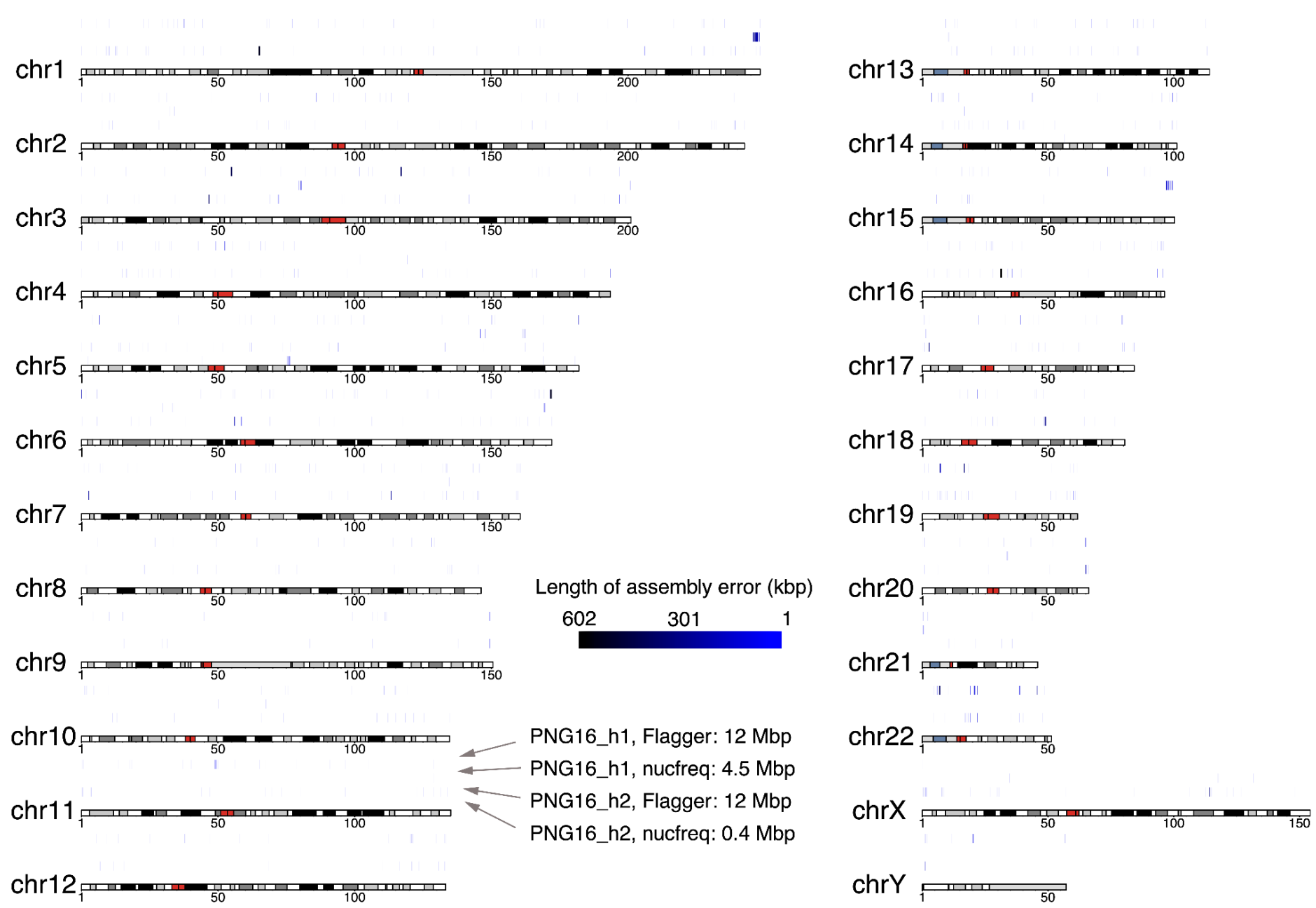

Fig. S2. Genome-wide distribution of assembly errors in PNG16 haplotype assemblies detected using Flagger as well as NucFreq. Vertical bars in each track show erroneous assembly loci, with color intensity indicating the size of errors.

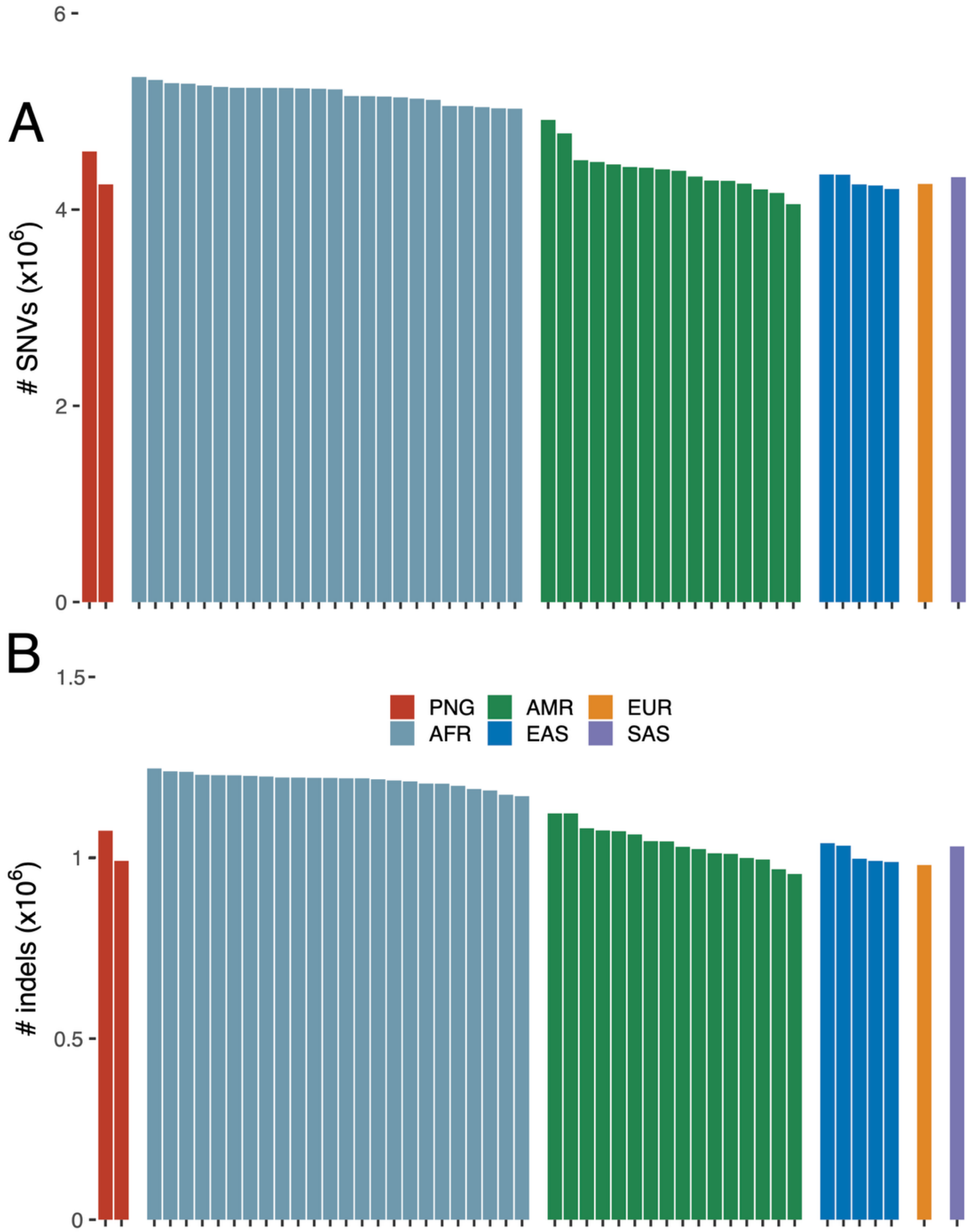

Fig. S3. Variation of (A) SNVs and (B) indels (<50 bp) across PNG and HPRCr1 cohorts. Each bar chart shows the number of variants in a given diploid genome. Variants within assembly errors and uncallable regions (Methods) were excluded. PNG: Papua New Guinea, AFR: African, AMR: admixed American, EAS: East Asian, EUR: European, and SAS: South Asian.

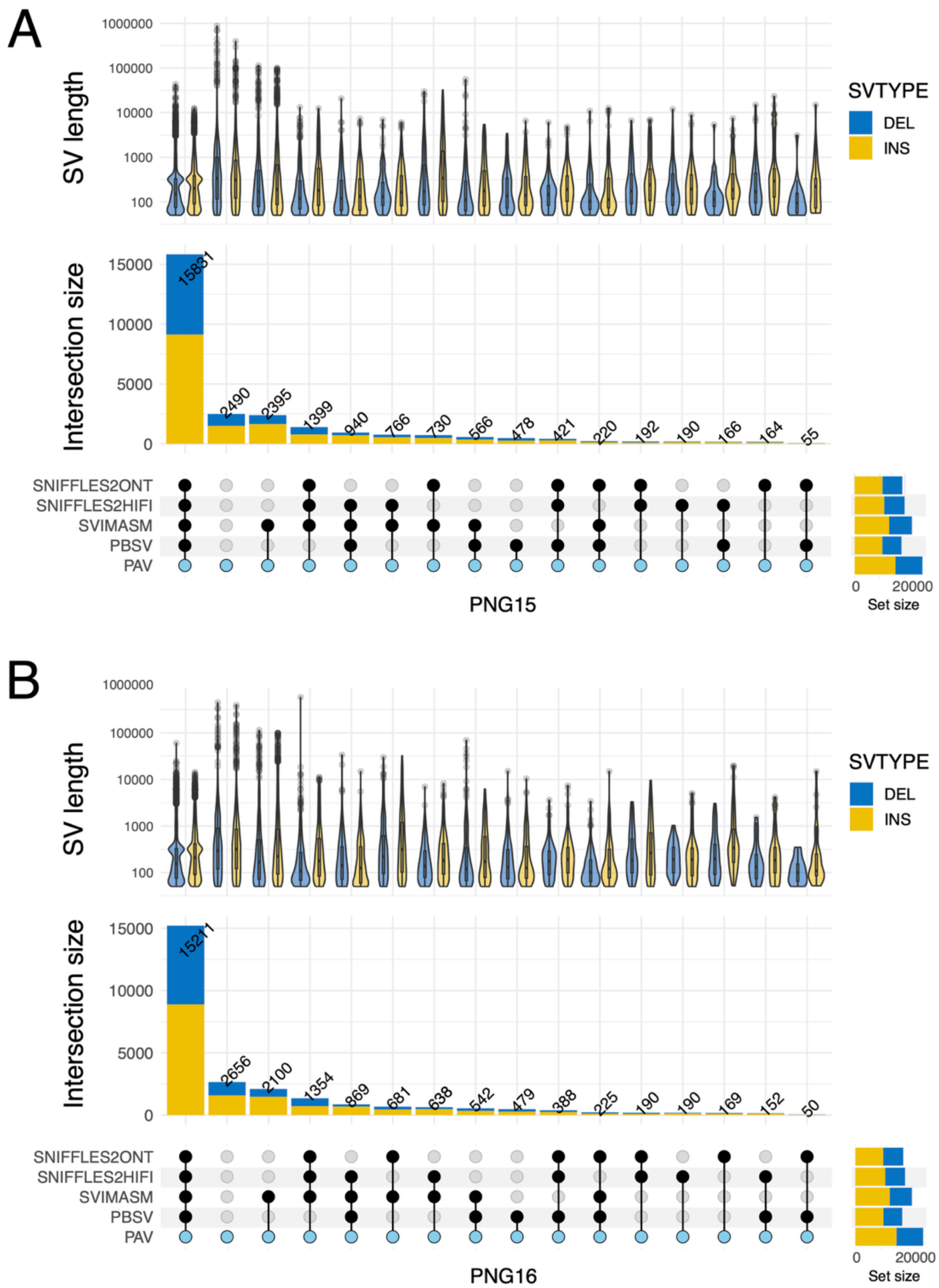

Fig. S4. UpSet plot comparing SV calls across five callers for (A) PNG15 and (B) PNG16. The analysis examines PAV and its intersection with the other four callers. The top panel shows the length distribution of SVs on a base-10 logarithmic scale, while the bottom panel displays the number of shared SVs across different combinations of callers, including PAV-unique calls. Deletion and insertion calls are color-coded for comparison.

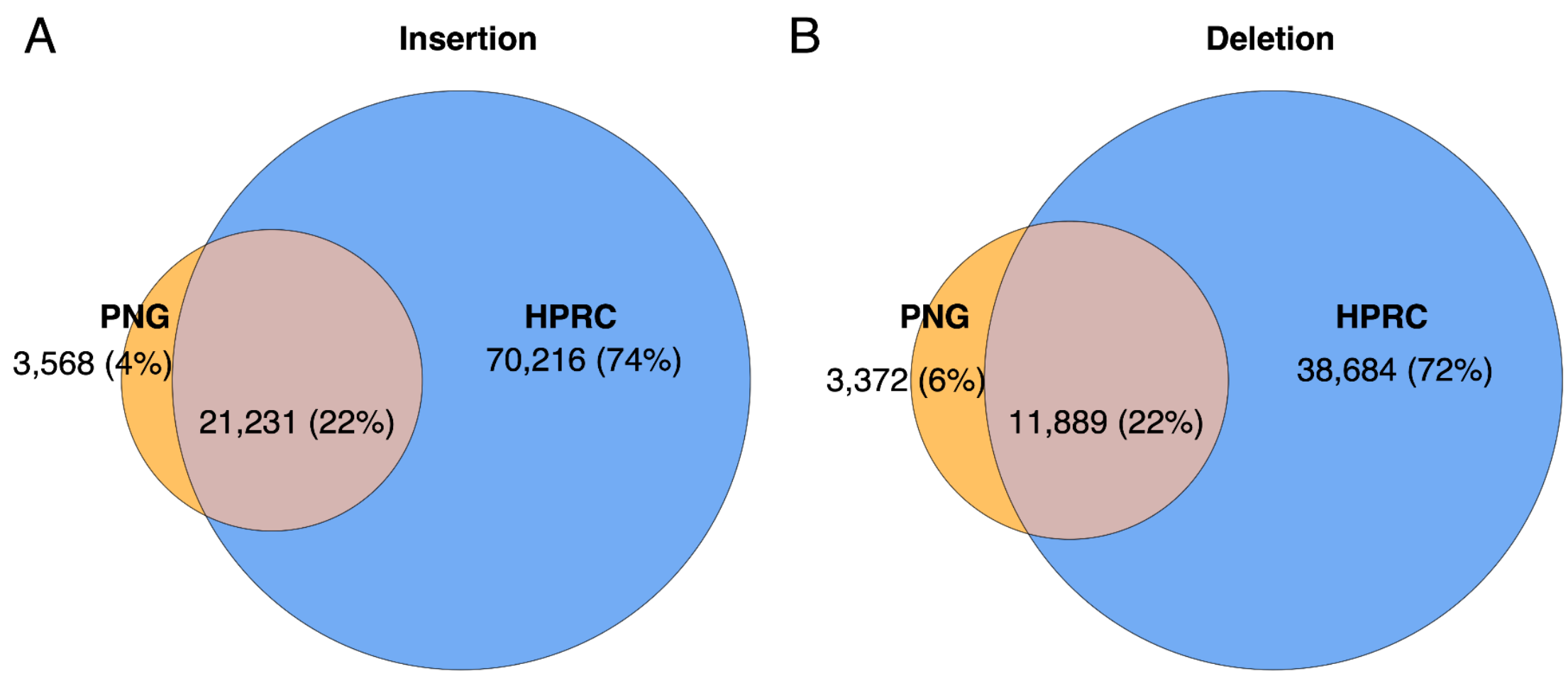

Fig. S5. Venn diagrams of shared and unique PAV (A) insertion and (B) deletion calls between PNG and HPRCr1 samples. SVs in erroneous assembly regions and uncallable sequences were excluded.

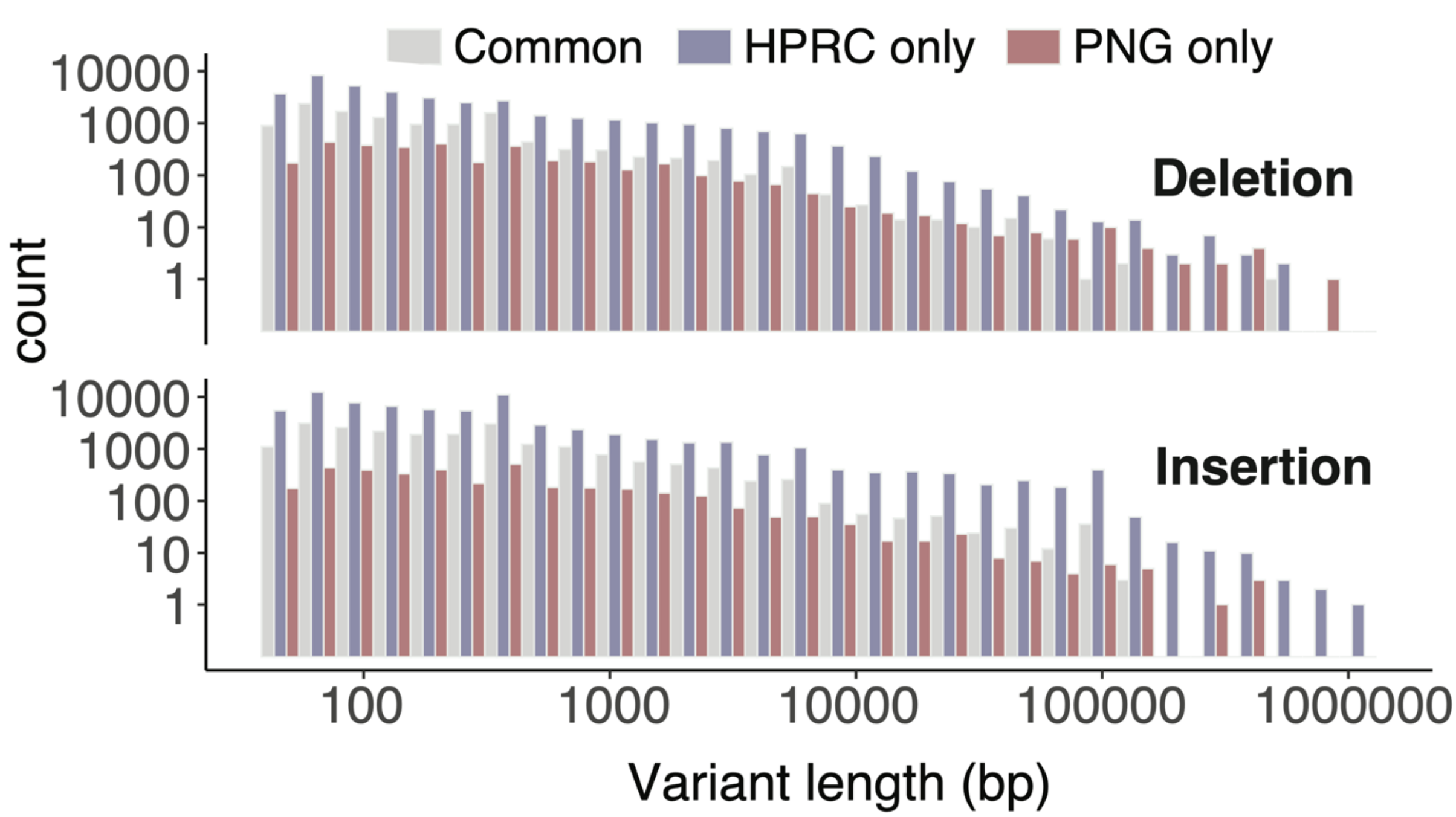

Fig. S6. Length comparison between shared and cohort-specific SVs (gray: shared, blue: HPRCr1, red: PNG). Top: Length distribution of deletion calls. Bottom: Length distribution of insertion calls. Both x- and y-axes use a base-10 logarithmic scale.

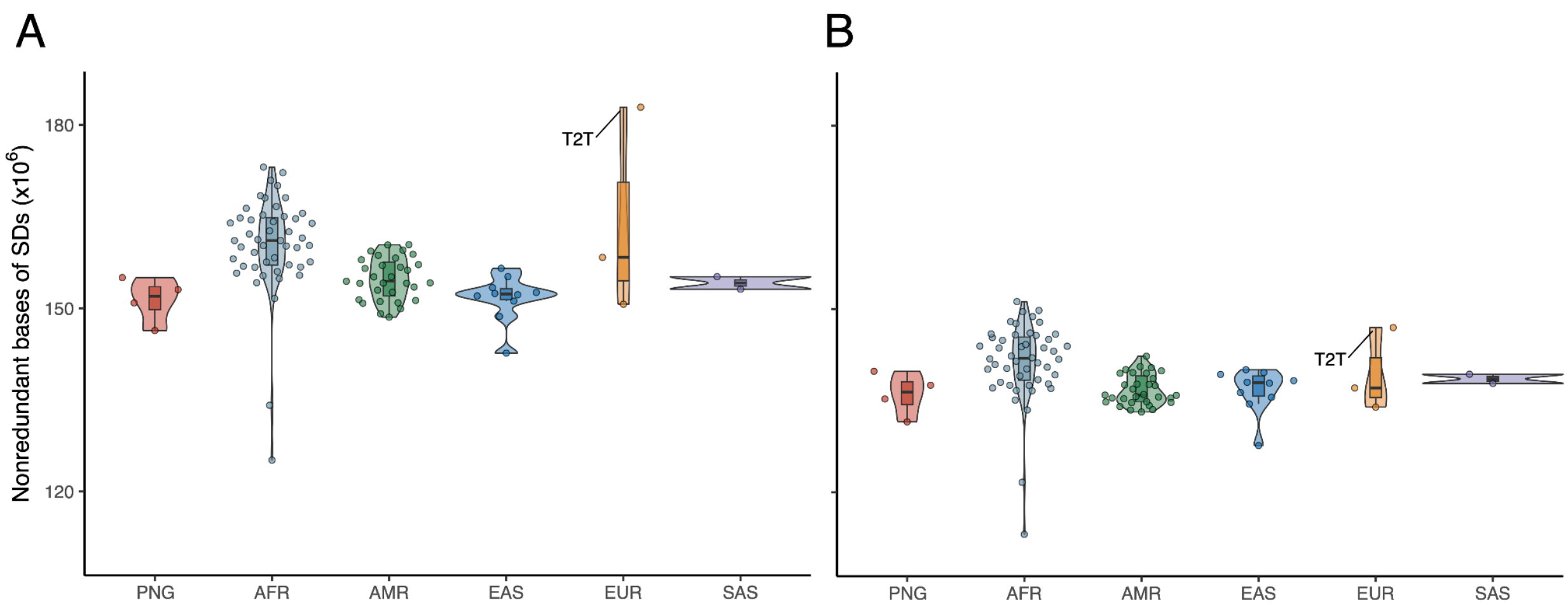

Fig. S7. Nonredundant SD bases across PNG and HPRCr1 haplotype assemblies. (A) All SDs and (B) SDs not in acrocentric/centromeric sequences are shown. Each point shows the measurement for a given haplotype assembly. In both analyses, SDs overlapping assembly errors are excluded.

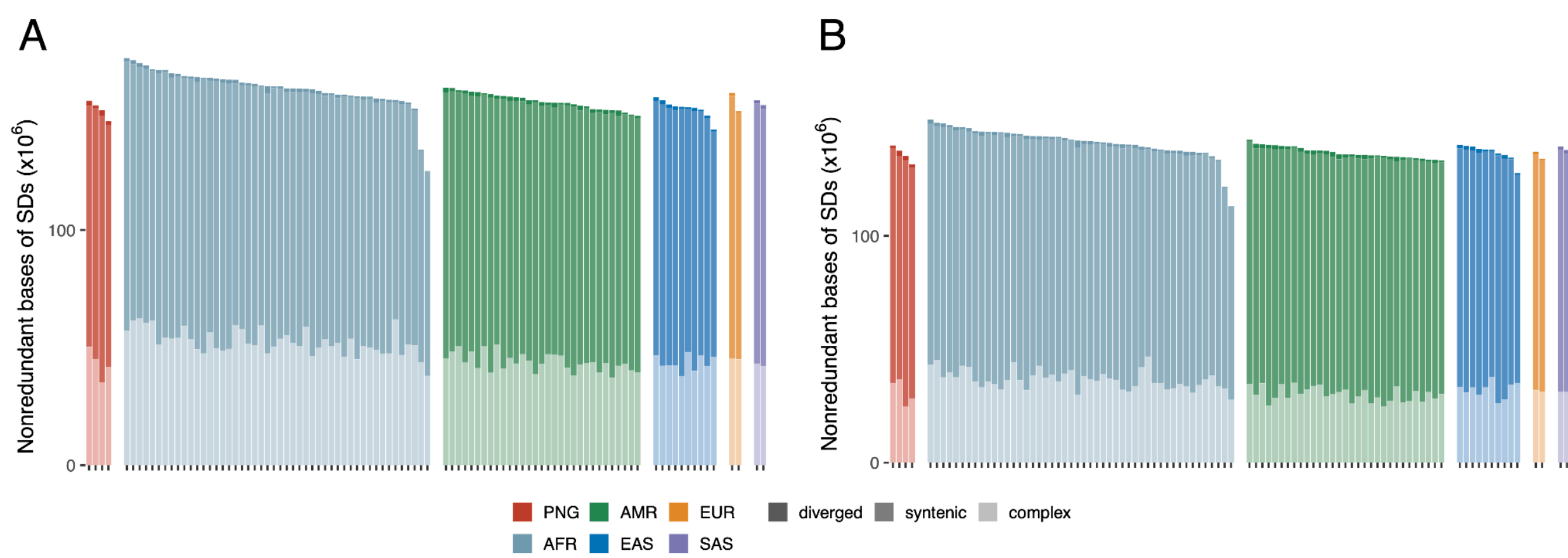

Fig. S8. The number of nonredundant SD bases in PNG and HPRCr1 assemblies. Each bar chart represents SDs from a haplotype. (A) All SDs and (B) SDs not in acrocentric/centromeric sequences are shown. In both analyses, SDs overlapping assembly errors are excluded. Each bar chart refers to data from an individual haplotype assembly. Complex SDs are shown in light color (bottom) and are those not aligned with T2T-CHM13 for at least 1 Mbp. Diverged SDs (dark color, top) and syntenic SDs (intermediate color, middle) are those that align at least 1 Mbp with T2T-CHM13. SDs lacking at least 50% reciprocal overlap with any SDs in T2T-CHM13 are classified as diverged.

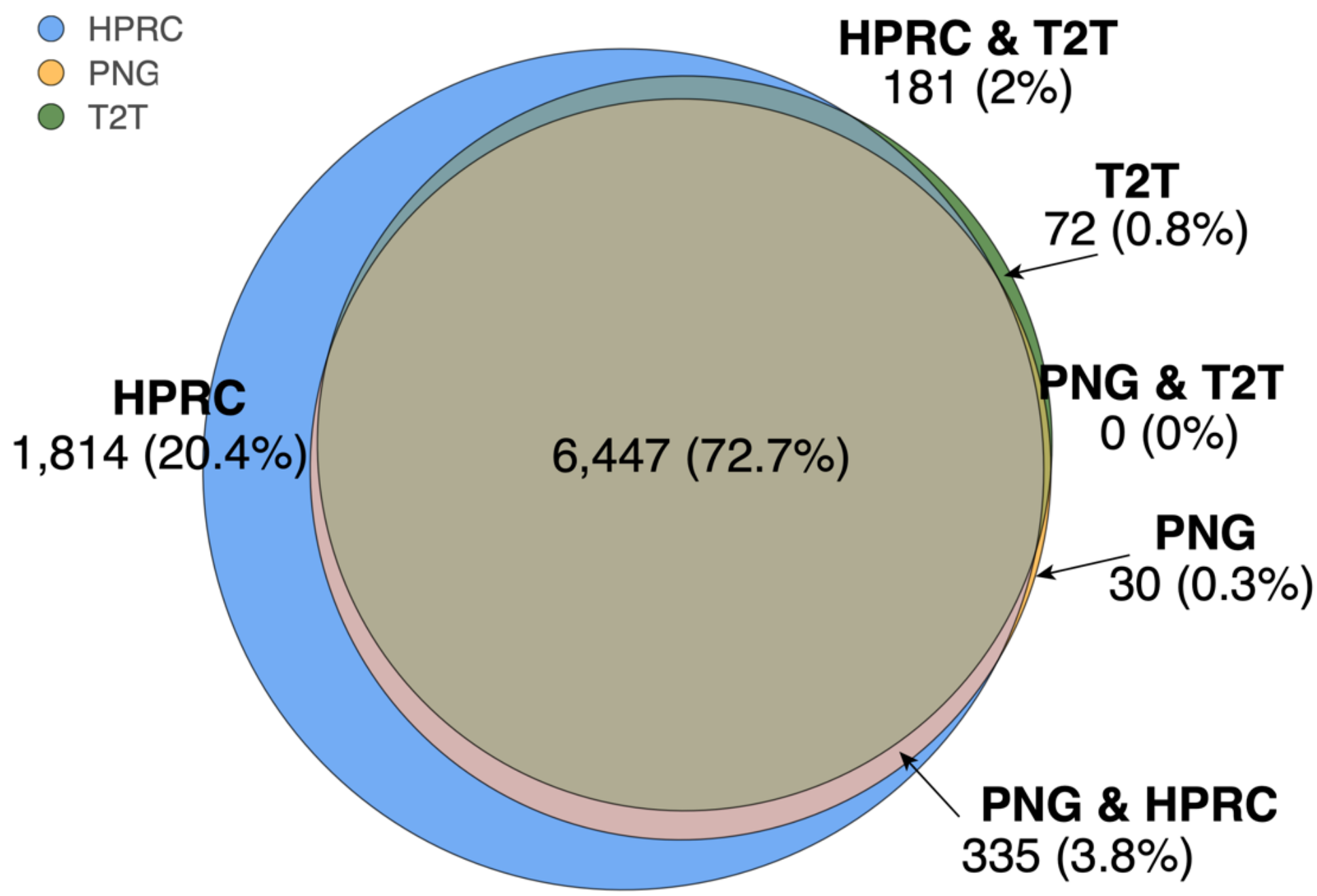

Fig. S9. Shared and unique nonredundant SD loci between assemblies: T2T-CHM13, PNG, and HPRCr1 haplotypes. SDs overlapping assembly errors were excluded.

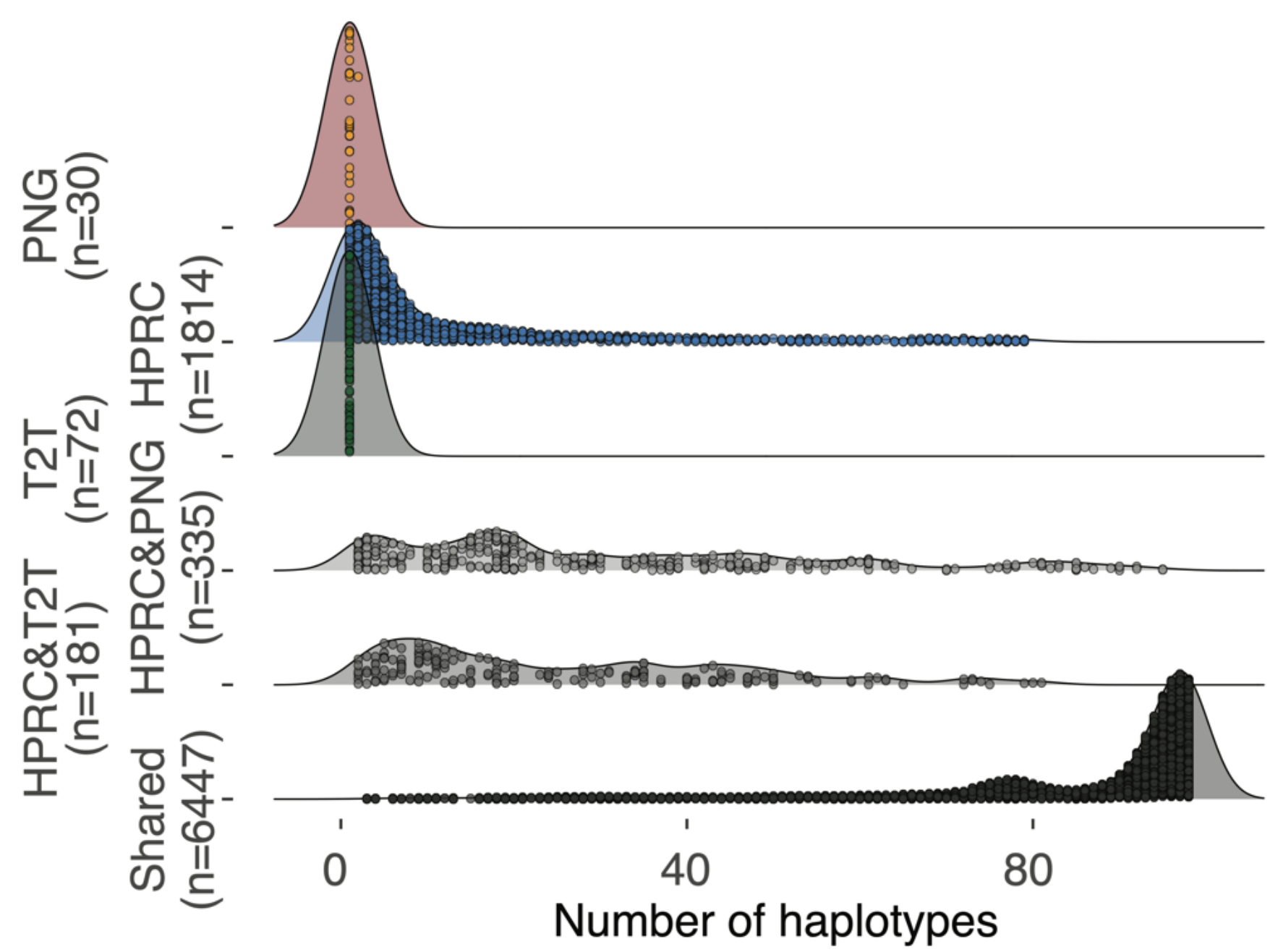

Fig. S10. Haplotype frequency of shared and cohort-specific nonredundant SD regions. Note that SDs in sequence alignments <1 Mbp of synteny with T2T-CHM13 and/or overlapping with assembly errors and acrocentric/centromeric regions were excluded from the SD analysis.

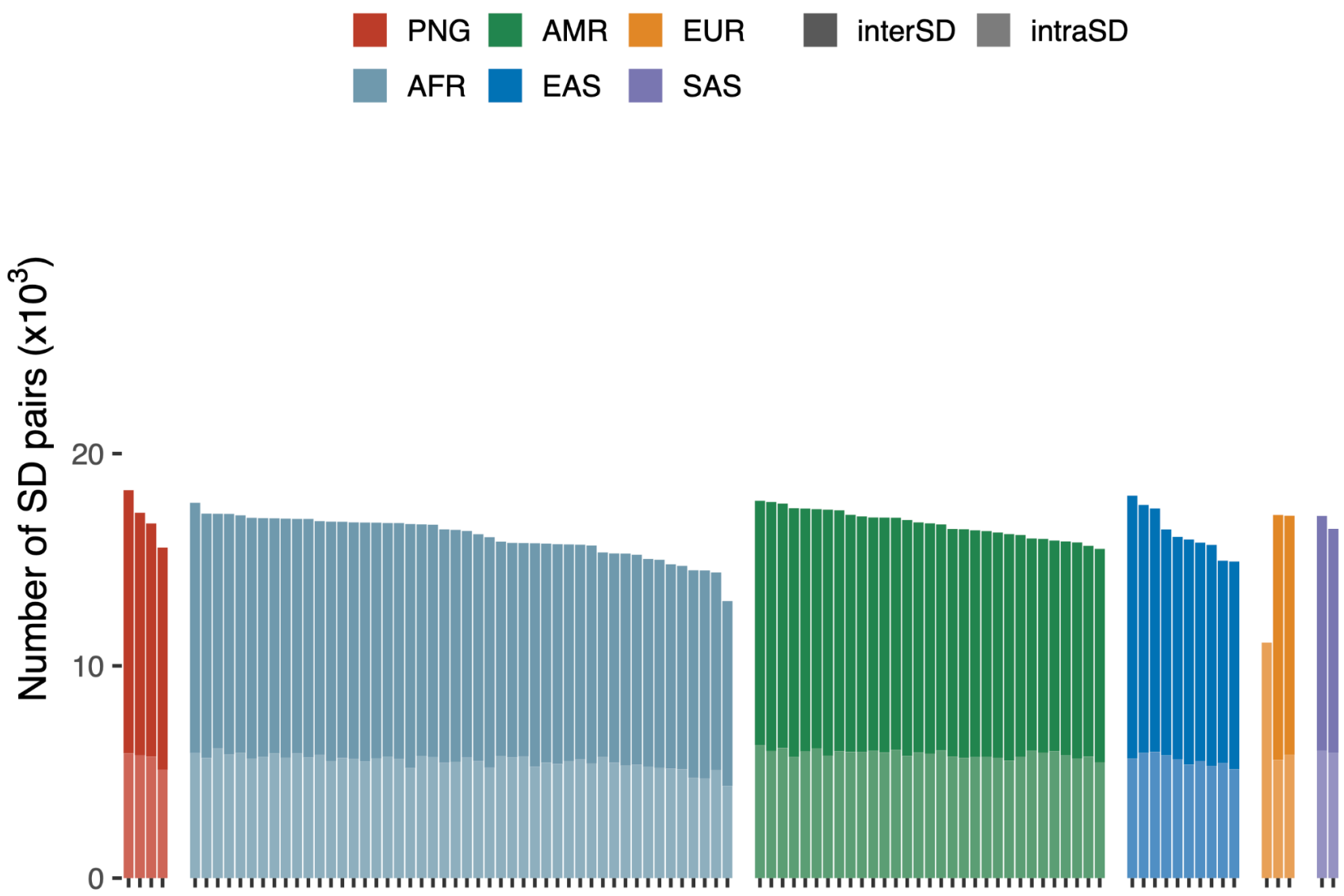

Fig. S11. Interchromosomal and intrachromosomal SDs in PNG and HPRCr1 assemblies. InterSD and intraSD are interchromosomal and intrachromosomal SDs, respectively. SDs overlapping assembly errors were excluded.

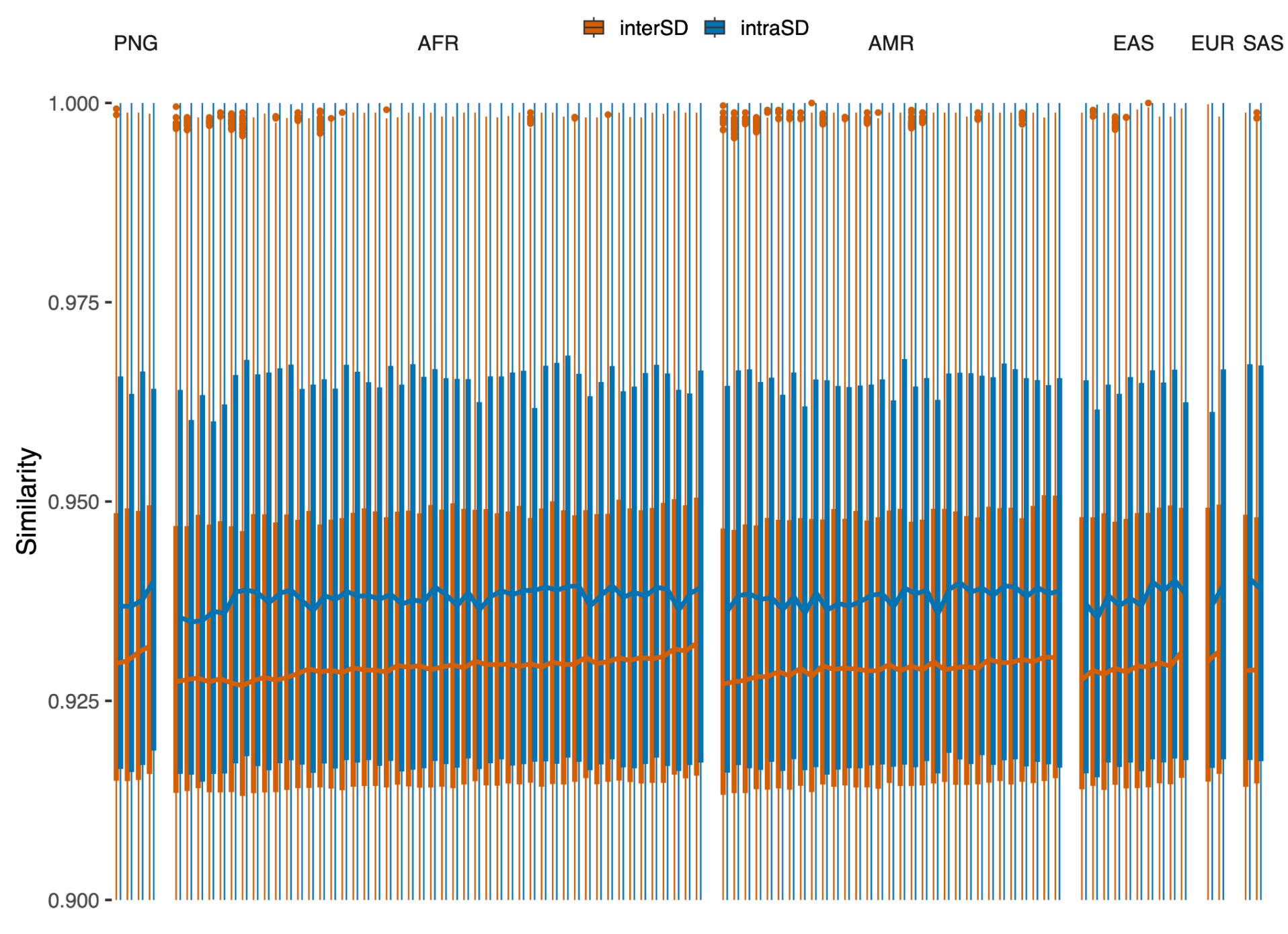

Fig. S12. Sequence similarity of interchromosomal and intrachromosomal SDs in assemblies. Each pair of boxplots—red for interSD and blue for intraSD—represents SDs from a specific haplotype assembly. Each boxplot shows the median (thick line), upper and lower quartiles (box), and 1.5× interquartile range (whiskers). The blue and red lines indicate median values across haplotypes.

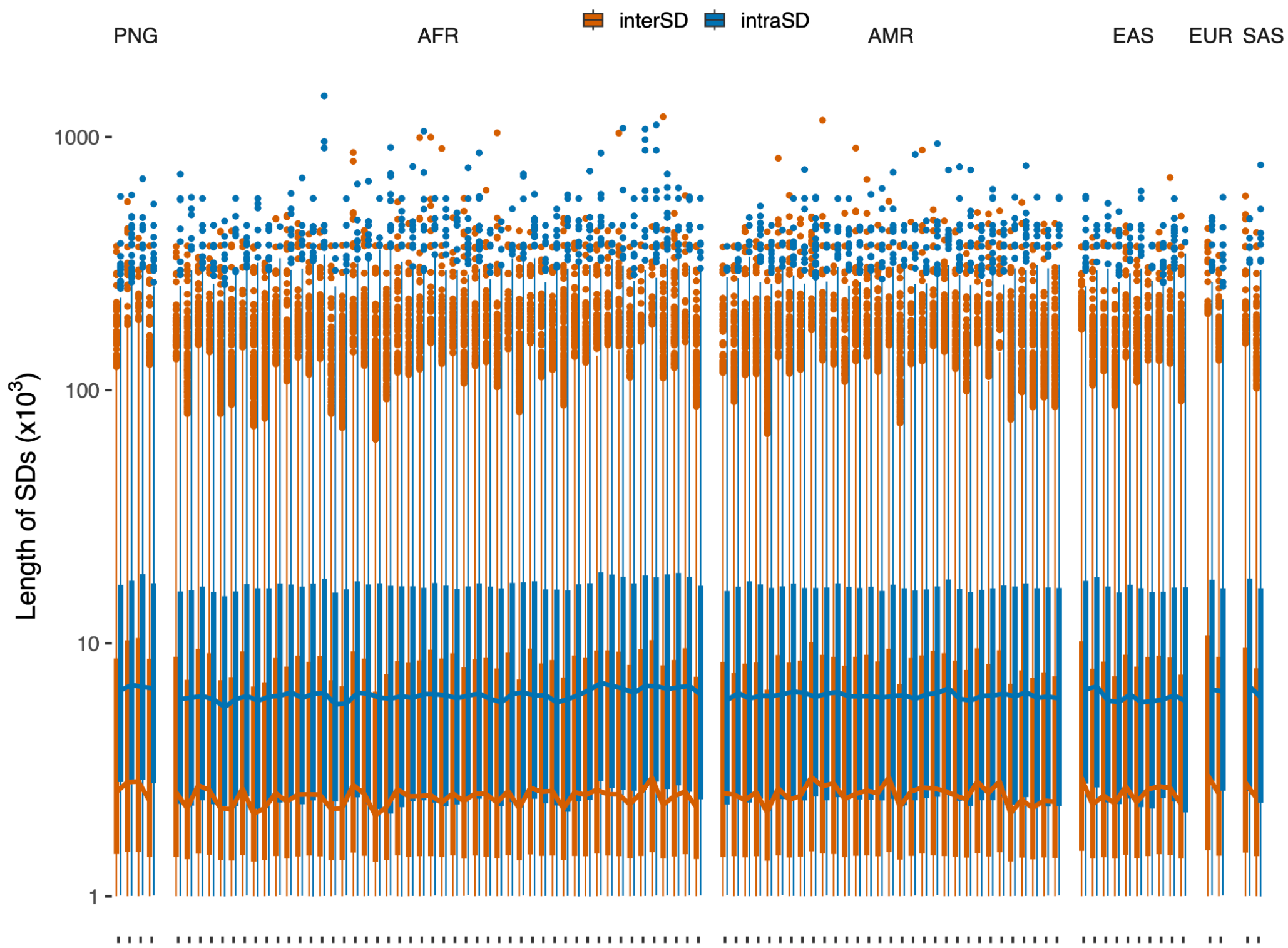

Fig. S13. Length distribution of interchromosomal and intrachromosomal SDs in assemblies. Each pair of boxplots—red for interSD and blue for intraSD—represents SDs from a specific haplotype assembly. Each boxplot shows the median (thick line), upper and lower quartiles (box), and 1.5× interquartile range (whiskers). The blue and red lines indicate median values across haplotypes.

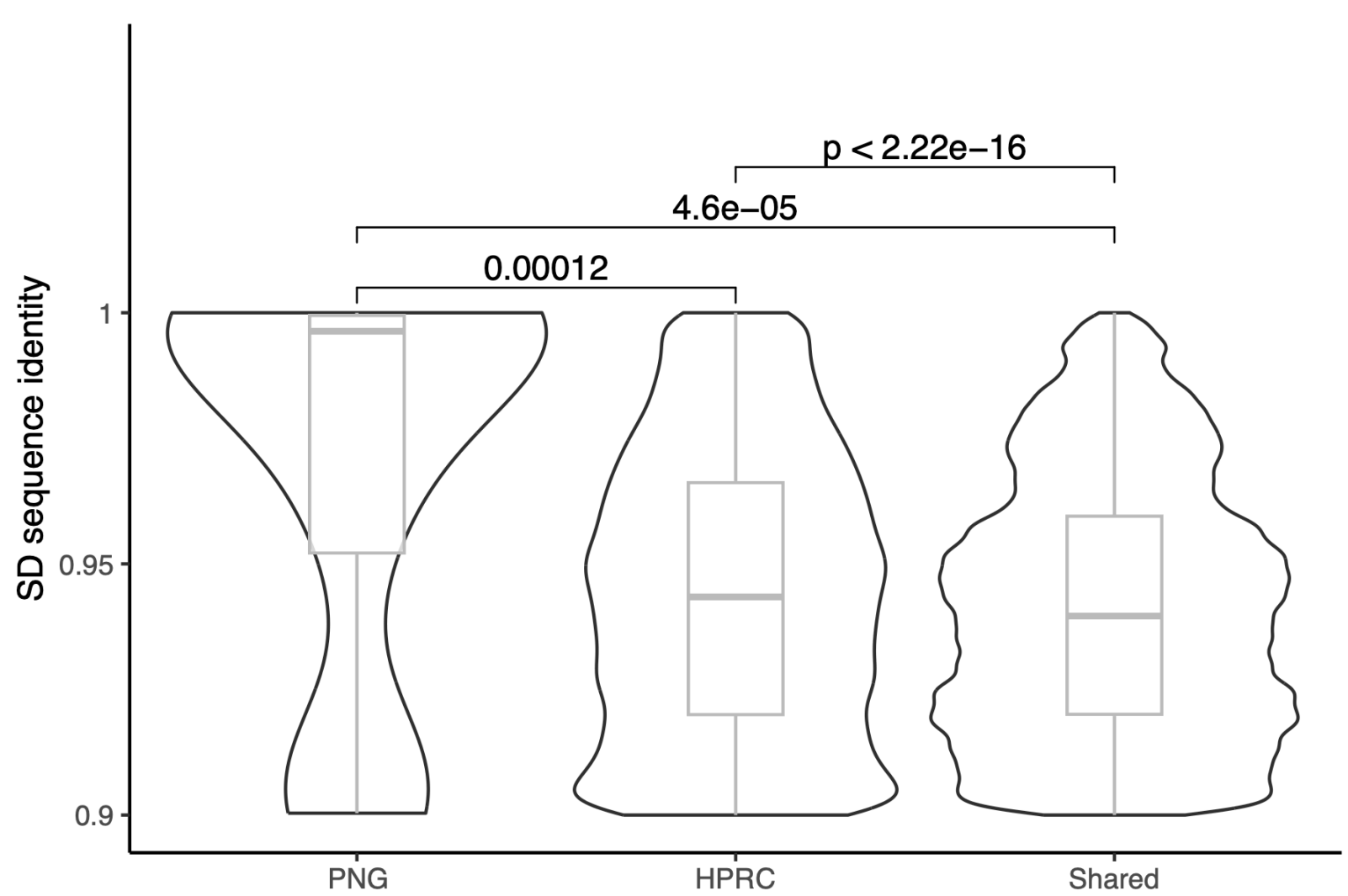

Fig. S14. Significant difference in sequence identity between cohort-specific and shared SDs. Note that PNG-specific SDs overall also have higher sequence identity than HPRCr1-specific SDs. Statistical significance was tested using Mann-Whitney U test without corrections for multiple testing. SDs overlapping assembly errors were excluded.

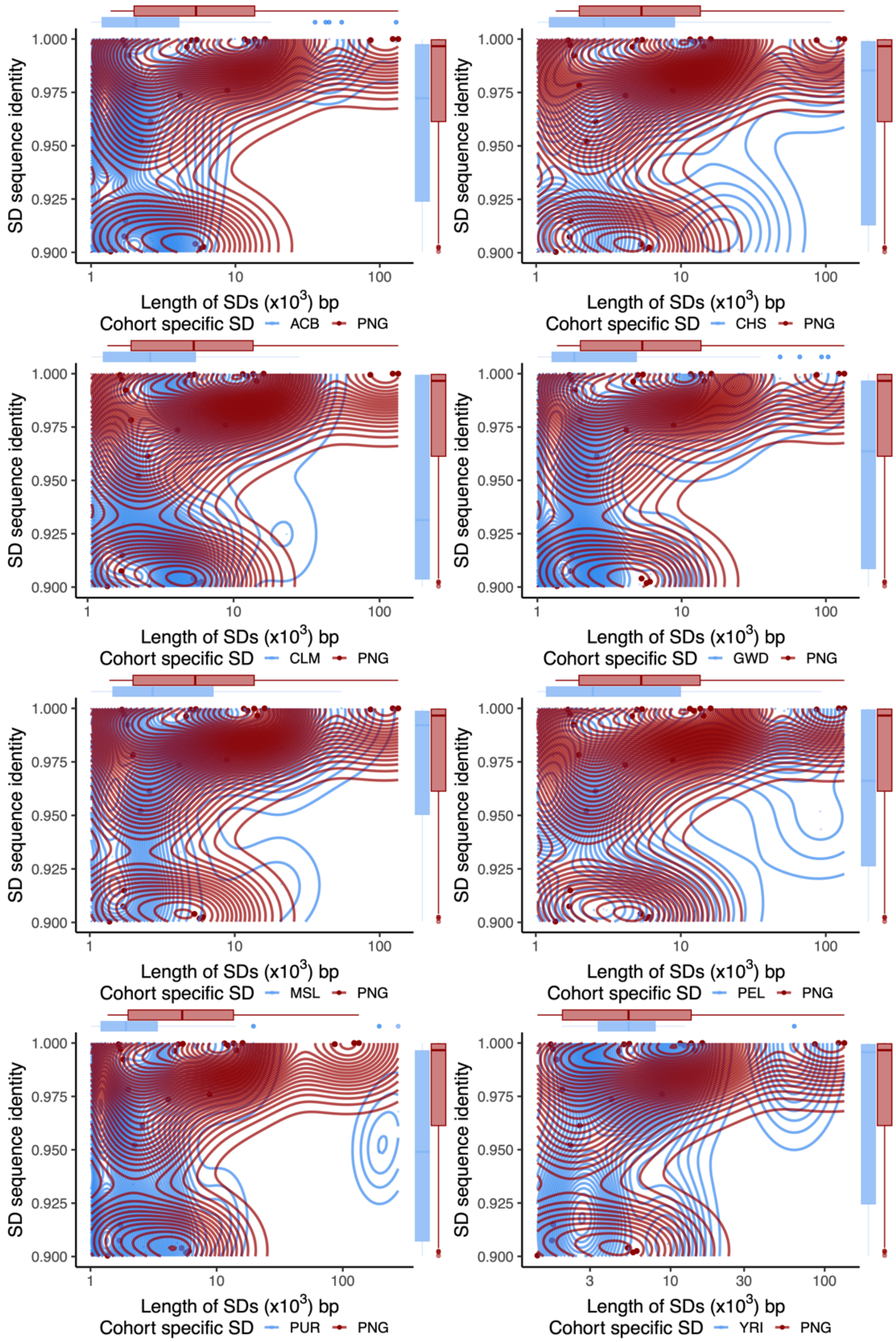

Fig. S15. Comparisons of length and sequence identity for population-specific SD pairs. Each boxplot represents the median (thick line), upper and lower quartiles (box), and 1.5× interquartile range (whiskers). Only populations with more than one sample are included in the analysis. SDs in sequences lacking at least 1 Mbp of synteny with T2T-CHM13 and/or overlapping with assembly errors and acrocentric/centromeric regions were excluded from the SD analysis. PNG: red; others: blue.

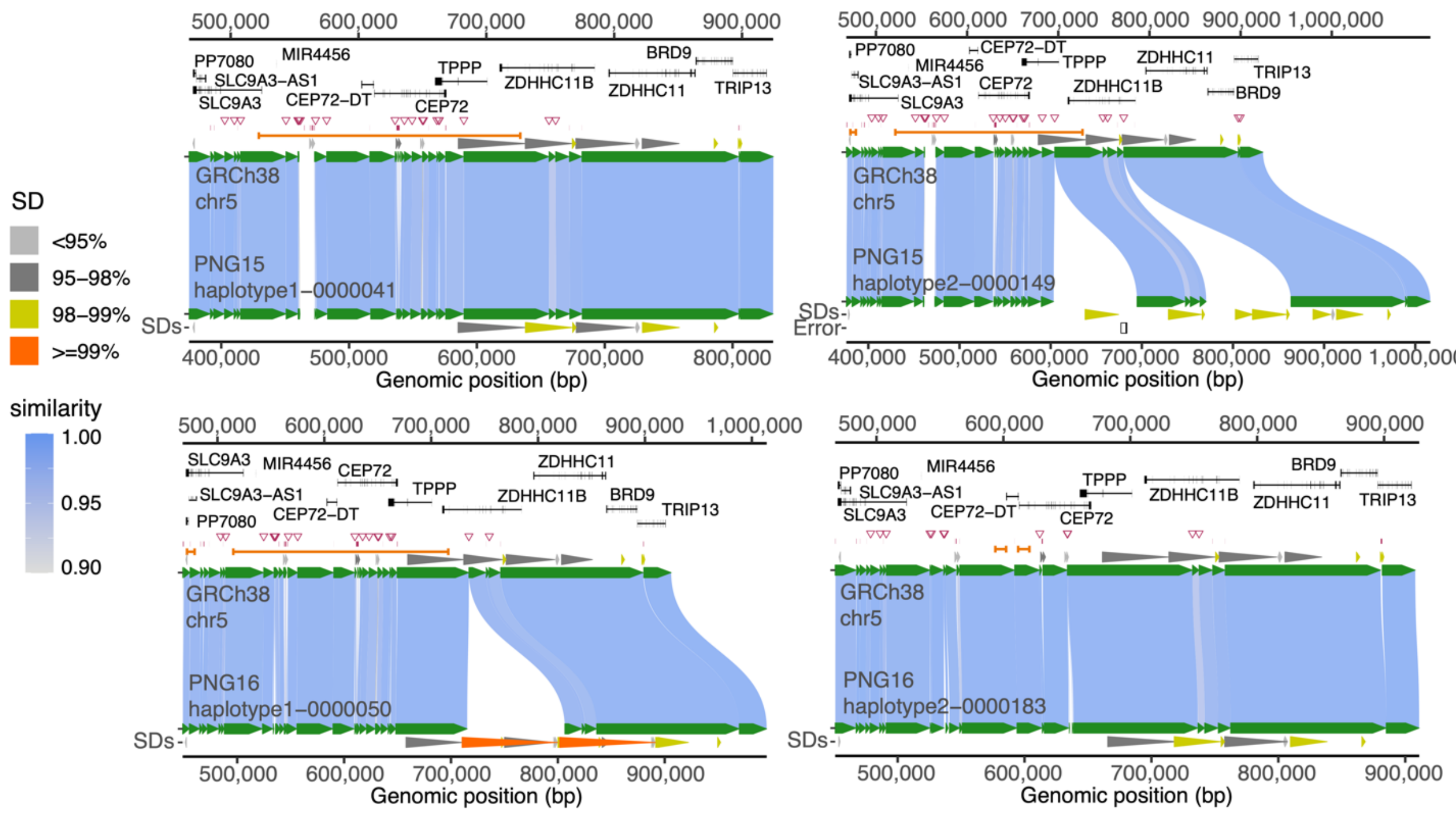

Fig. S16. Diverged SDs and SVs at the *TPPP*/*CEP72* locus in the newly assembled PNG haplotypes. The top represents the GRCh38 reference genome aligned to the bottom, new genome assemblies. The gene annotation of the reference genome is shown at the top. The red triangles and vertical rectangles indicate insertion and deletion locations in the GRCh38 coordinates, respectively.

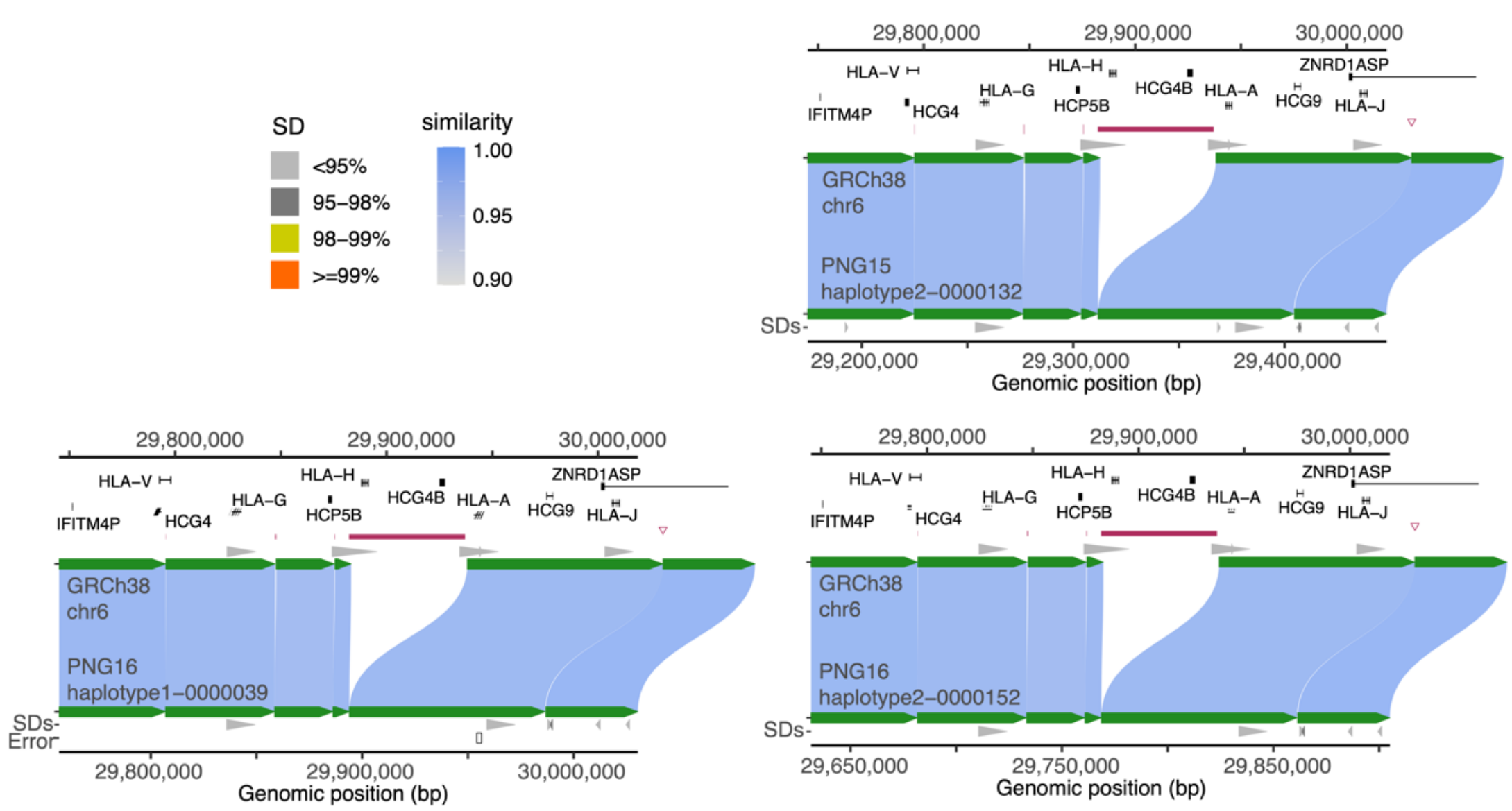

Fig. S17. Diverged SDs and SVs at the *HLA-H*/*HCG4B* locus in the newly assembled PNG haplotypes. The top represents the GRCh38 reference genome aligned to the bottom, new genome assemblies. The gene annotation of the reference genome is shown at the top. The red rectangles indicate the 54-kbp PNG deletion locations with respect to the GRCh38 coordinates.

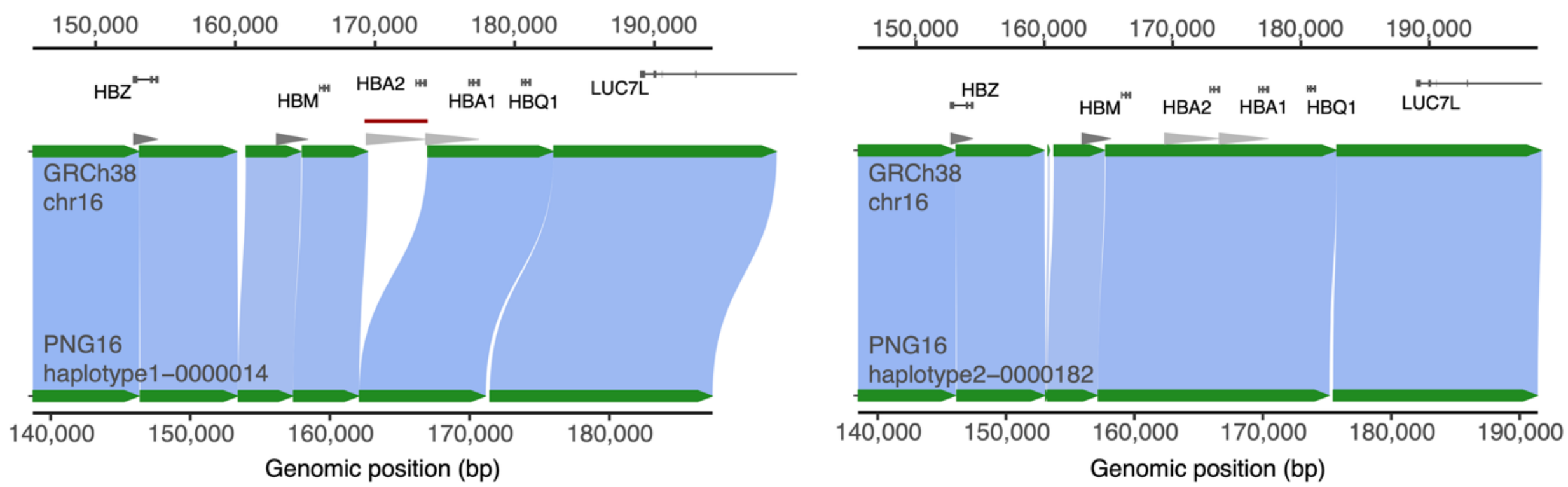

Fig. S18. Diverged SDs and SVs at the *HBA2* locus in the newly assembled PNG haplotypes. The top represents the GRCh38 reference genome aligned to the bottom, new PNG16 genome assemblies (Left: haplotype1; Right: haplotype 2). The gene annotation of the reference genome is shown at the top. The red rectangles indicate the 4.2-kbp PNG deletion locations with respect to the GRCh38 coordinates

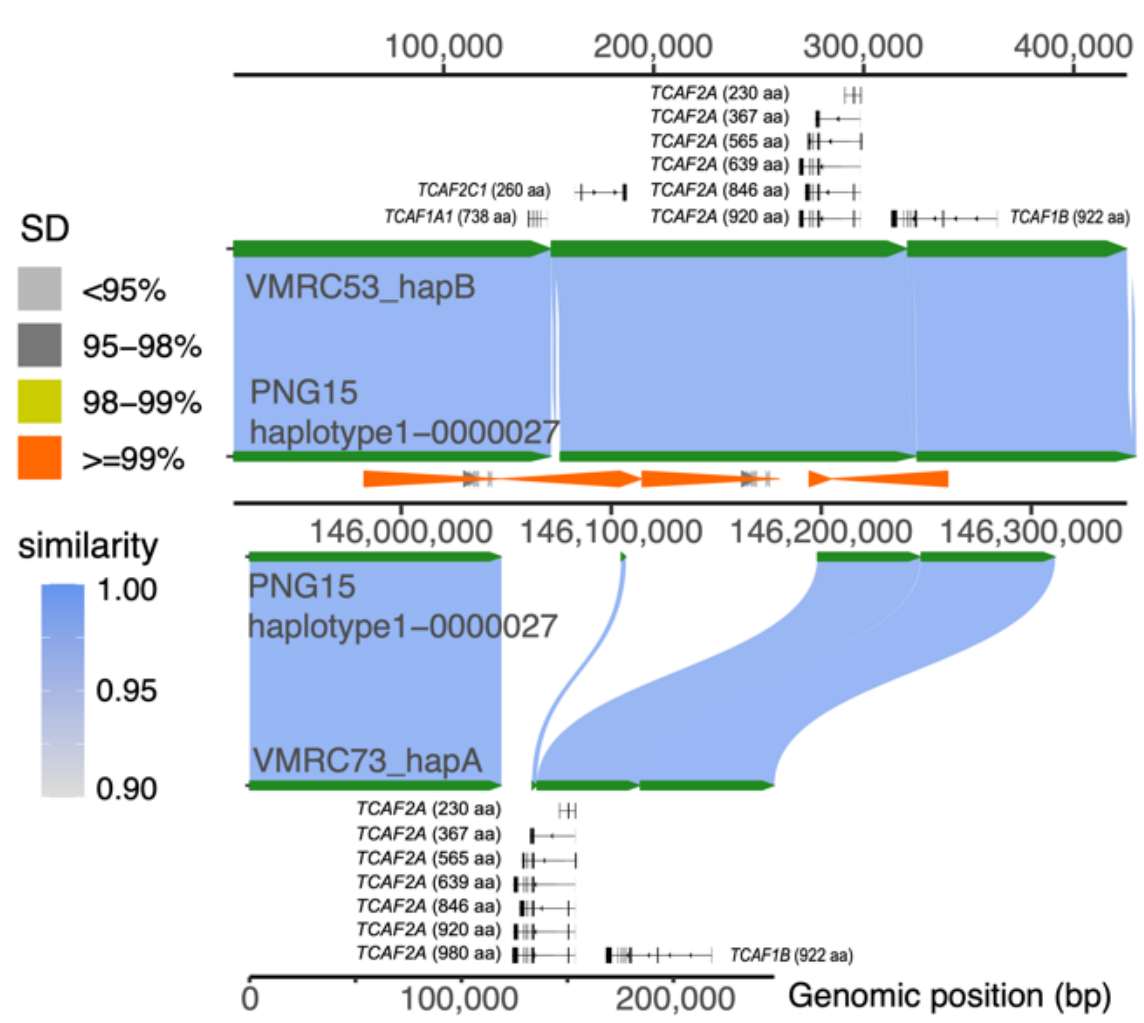

Fig. S19. Diverged SDs and SVs at the *TCAF* locus in the newly assembled PNG haplotypes. The top represents the published, gap-free VMRC53_hapB sequence aligned to the middle, new PNG assemblies, followed by alignments against another published, gap-free contig, VMRC73_hapA, which was sequenced from a Melanesian individual (Hsieh et al., 2021). Gene annotations are based on full-length Iso-Seq data from Hsieh et al., 2021. SDs predicted by WGAC (**Methods**).

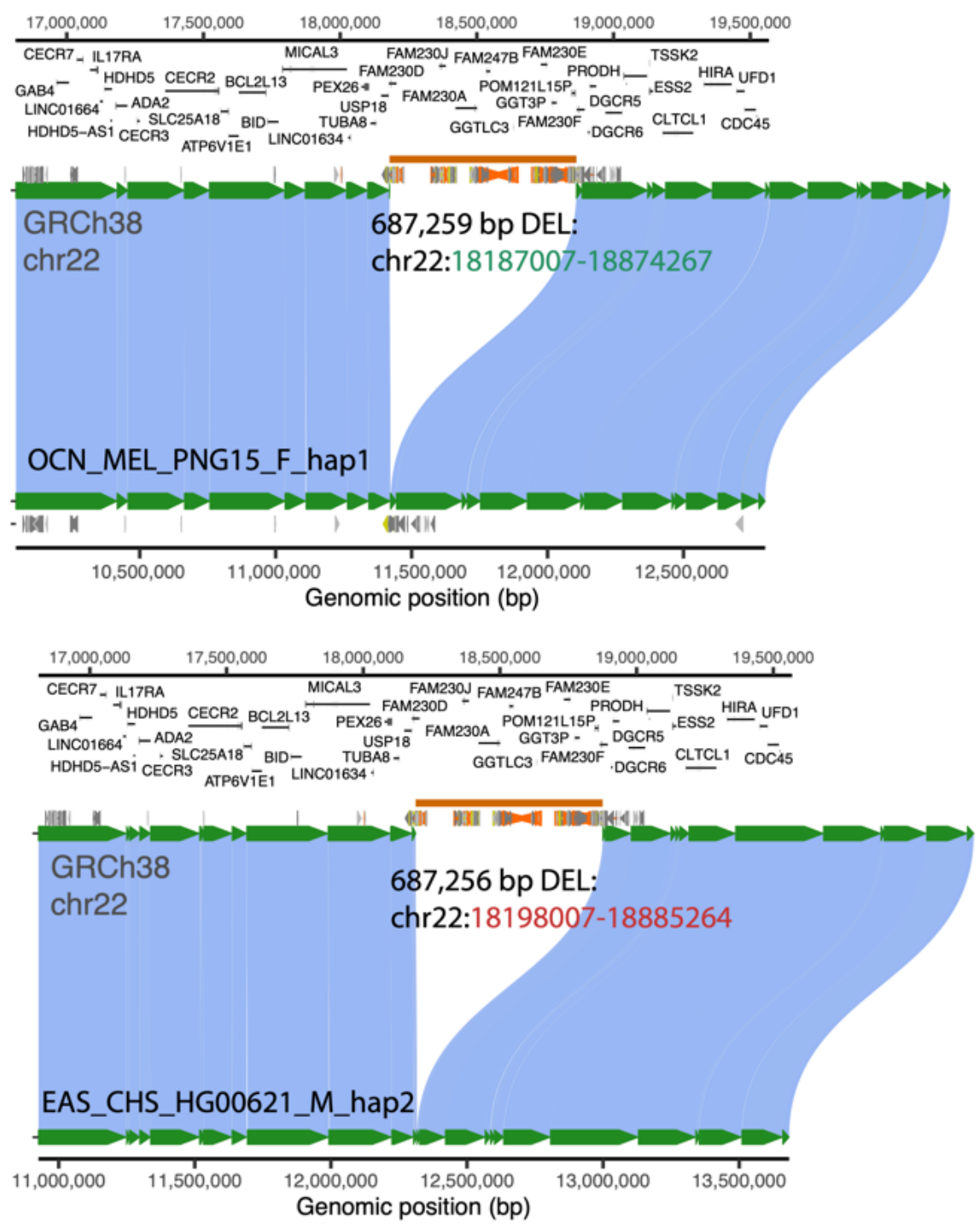

Fig. S20. Diverged SDs and SVs at the chr22q11.2 DiGeorge syndrome locus in the assembled PNG and HPRCr1 haplotypes. The top represents the GRCh38 reference genome aligned to the bottom, PNG/HPRCr1 assemblies. The gene annotation of the reference genome is shown at the top. The red rectangles indicate the 0.68 Mbp deletion locations in with respective to the GRCh38 coordinates.

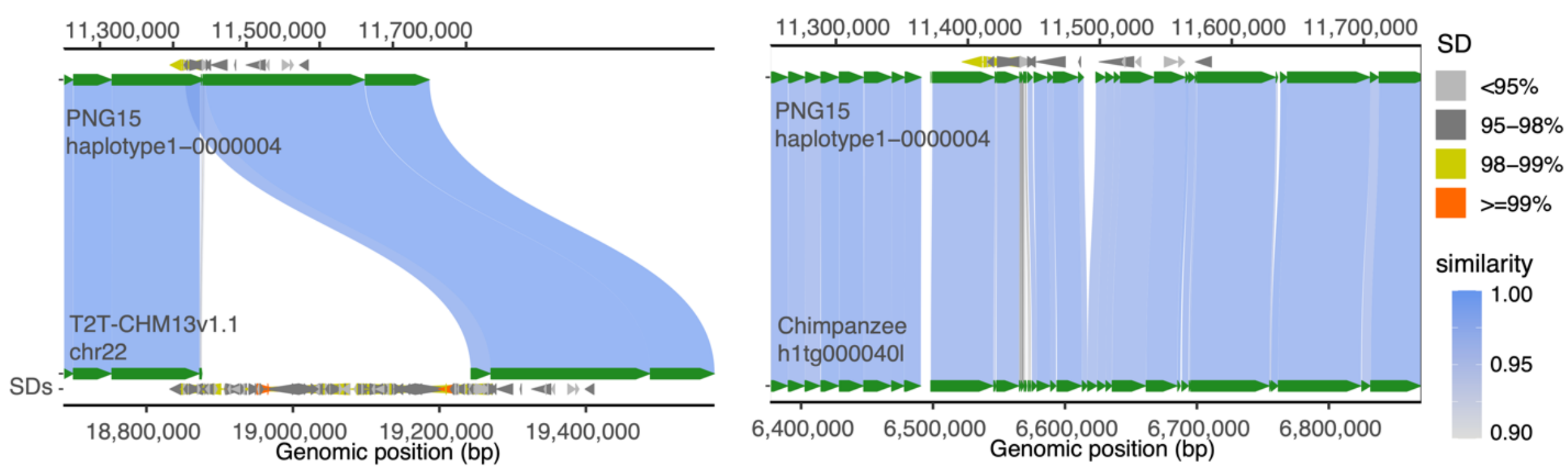

Fig. S21. Diverged SDs and SVs at the chr22q11.2 DiGeorge syndrome locus in the assembled PNG, T2T-CHM13v1.1, and chimpanzee haplotypes. (Left) The top represents the PNG15 deletion haplotype aligned to the bottom, T2T-CHM13v1.1 assembly. (Right) Alignment between the PNG15 deletion haplotype (top) and a high-quality chimpanzee assembled contig (bottom) shows similar sequence organization. SDs predicted by WGAC (Methods).

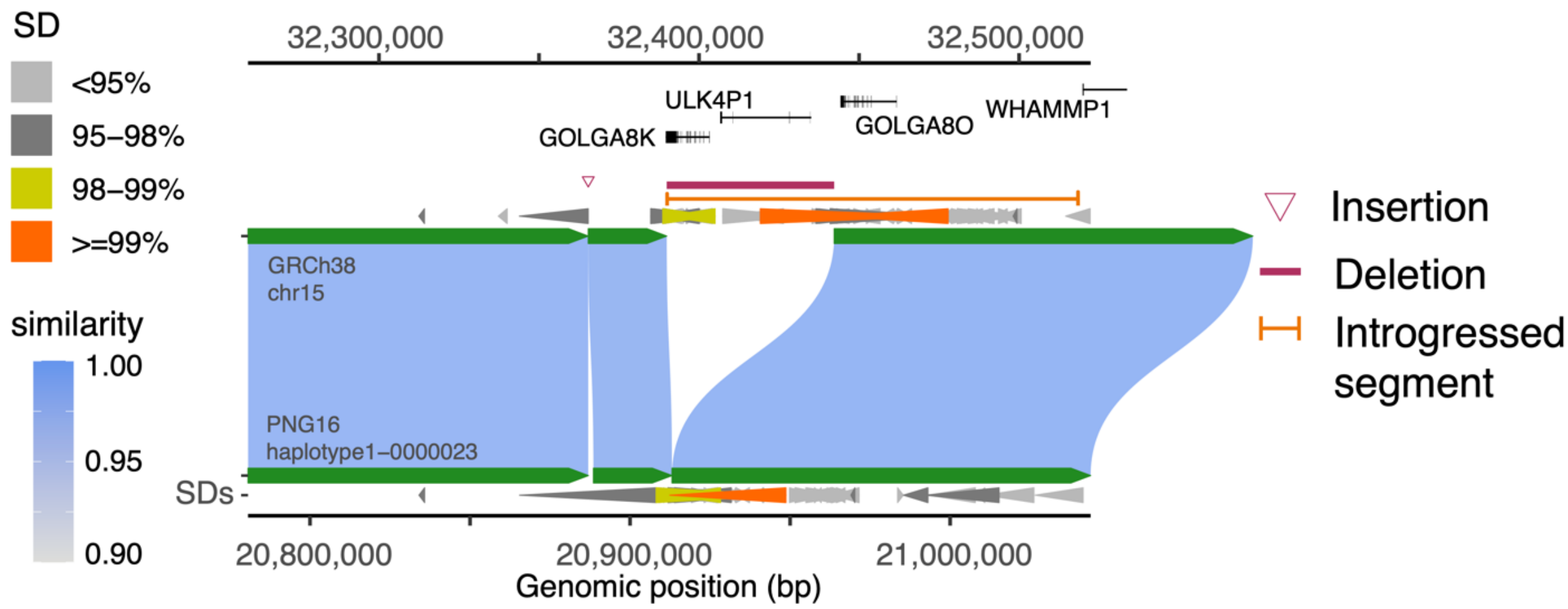

Fig. S22. Diverged SDs and SVs at the *GOLGA8K/ULK4P1* locus in the newly assembled PNG haplotypes. The top represents the GRCh38 reference genome aligned to the bottom, new genome assemblies. The gene annotation of the reference genome is shown at the top. The red rectangles indicate the 54-kbp PNG deletion locations in with respective to the GRCh38 coordinates.

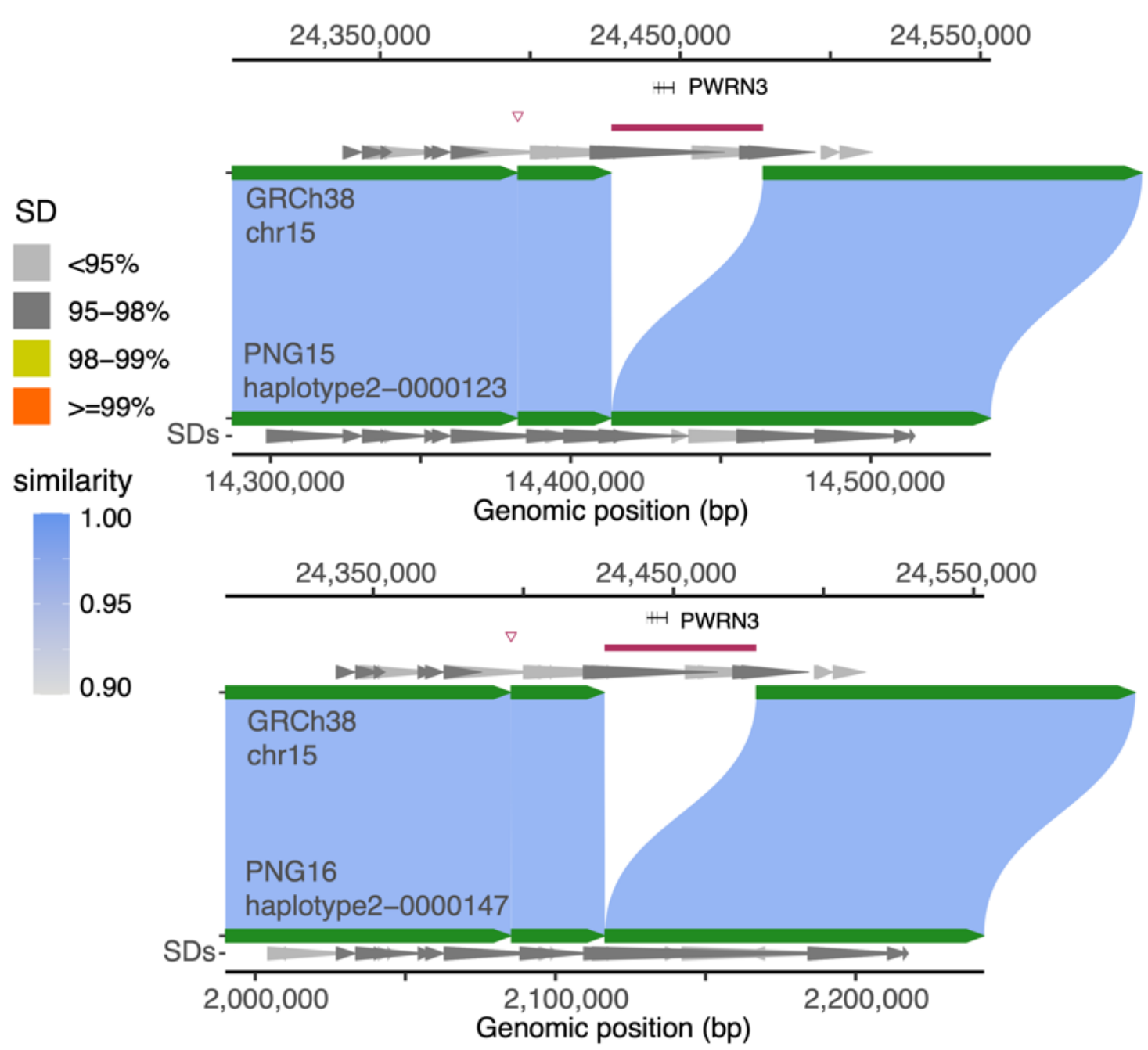

Fig. S23. Diverged SDs and SVs at the *PWRN3* locus in the newly assembled PNG haplotypes. The top represents the GRCh38 reference genome aligned to the bottom, new genome assemblies. The gene annotation of the reference genome is shown at the top. The red rectangles indicate the PNG deletion locations in with respective to the GRCh38 coordinates.

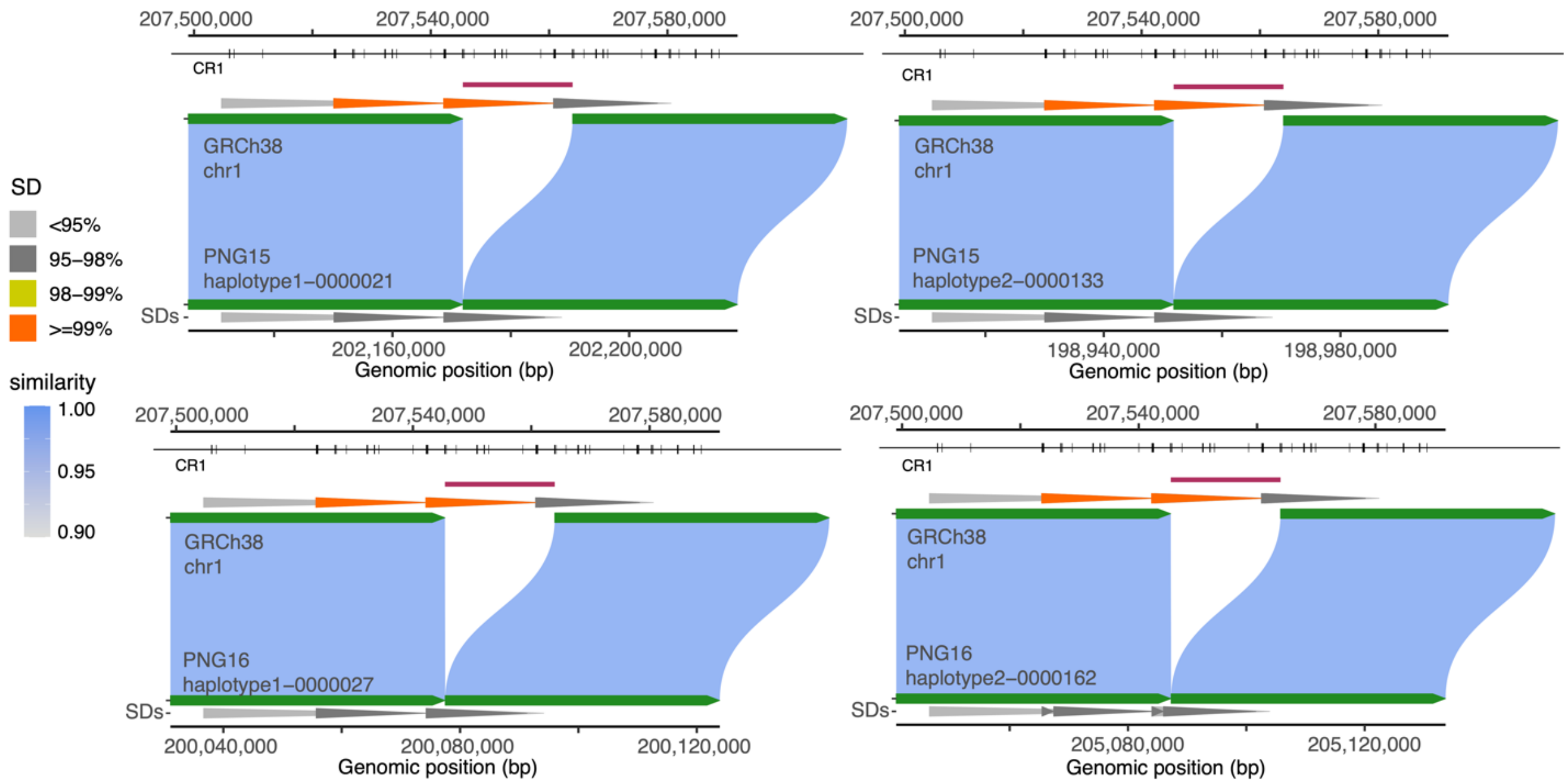

Fig. S24. Diverged SDs and SVs at the *CR1* locus in the newly assembled PNG haplotypes. The top represents the GRCh38 reference genome aligned to the bottom, new genome assemblies. The gene annotation of the reference genome is shown at the top. The red rectangles indicate the PNG deletion locations in with respective to the GRCh38 coordinates.

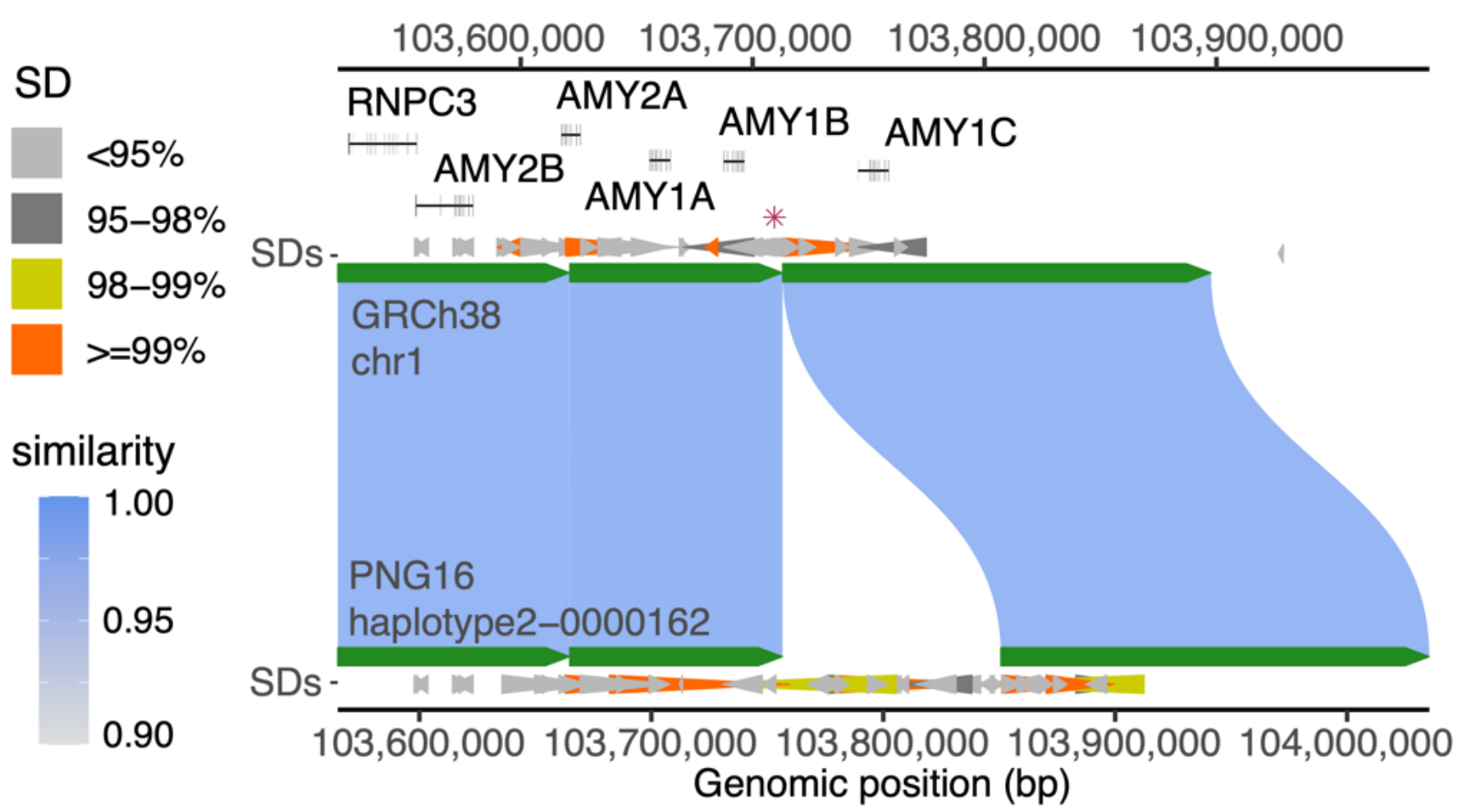

Fig. S25. Diverged SDs at the amylase locus in the newly assembled PNG haplotypes. The top represents the GRCh38 reference genome aligned to the bottom, new genome assemblies. The gene annotation of the reference genome is shown at the top. The red asterisk indicates an PNG insertion in the GRCh38 coordinates.

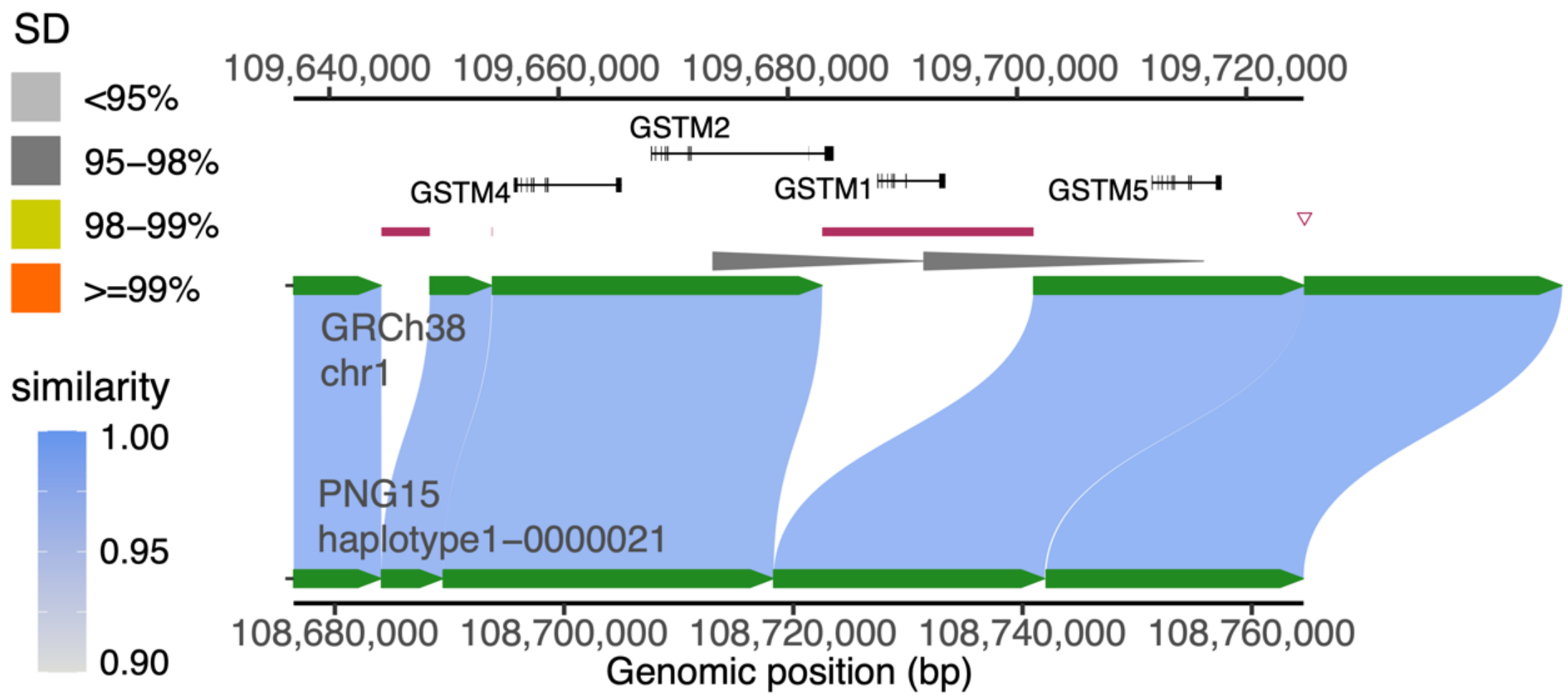

Fig. S26. Diverged SDs and SVs at the *GSTM1* locus in the newly assembled PNG haplotypes. The top represents the GRCh38 reference genome aligned to the bottom, new genome assemblies. The gene annotation of the reference genome is shown at the top. The red rectangles indicate the PNG deletion locations in with respective to the GRCh38 coordinates.

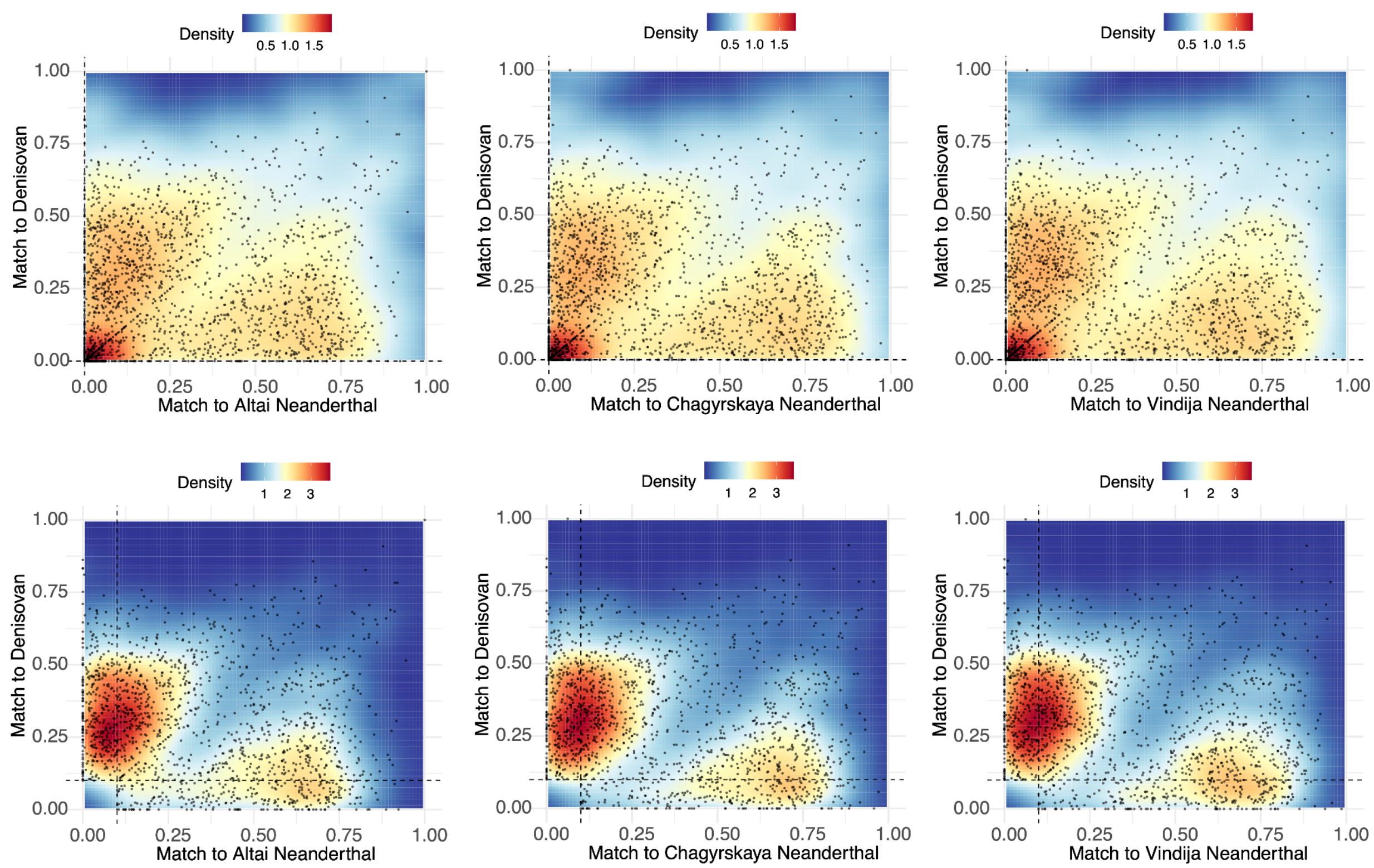

Fig. S27. Heatmap plots of matched proportion to a Neanderthal and a Denisovan genomes in PNG introgressed segments (dots). The proportion of putative archaic-specific alleles in each segment was computed for a given archaic genome. Top: all putative introgressed segments. Bottom: segments with less than 10% match rate on both axes were excluded due to low confidence.

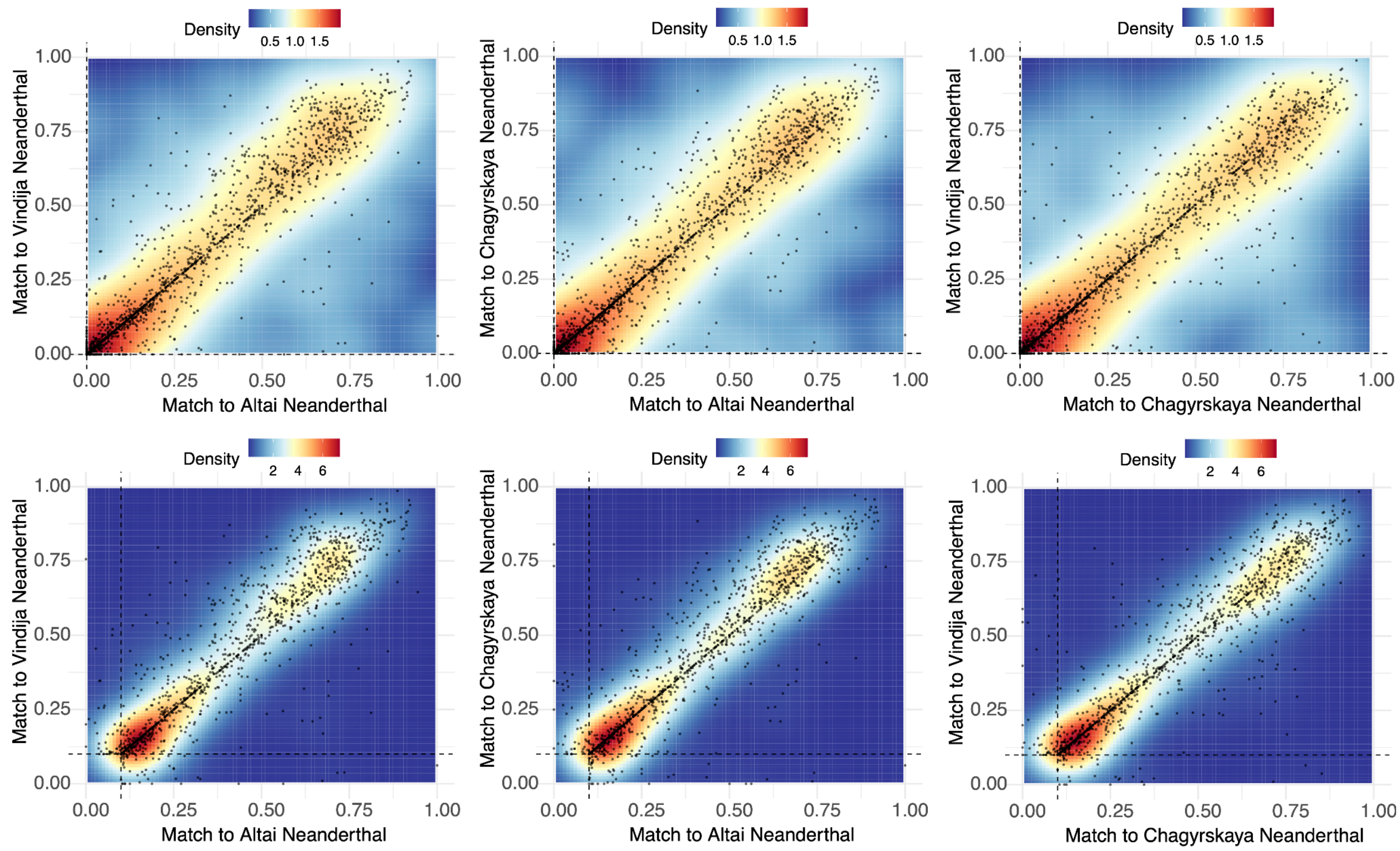

Fig. S28. Heatmap plots of matched proportion to pairs of Neanderthal genomes in PNG introgressed segments (dots). The proportion of putative archaic-specific alleles in each segment was computed for a given archaic genome. Top: all putative introgressed segments. Bottom: segments with less than 10% match rate on both axes were excluded due to low confidence.

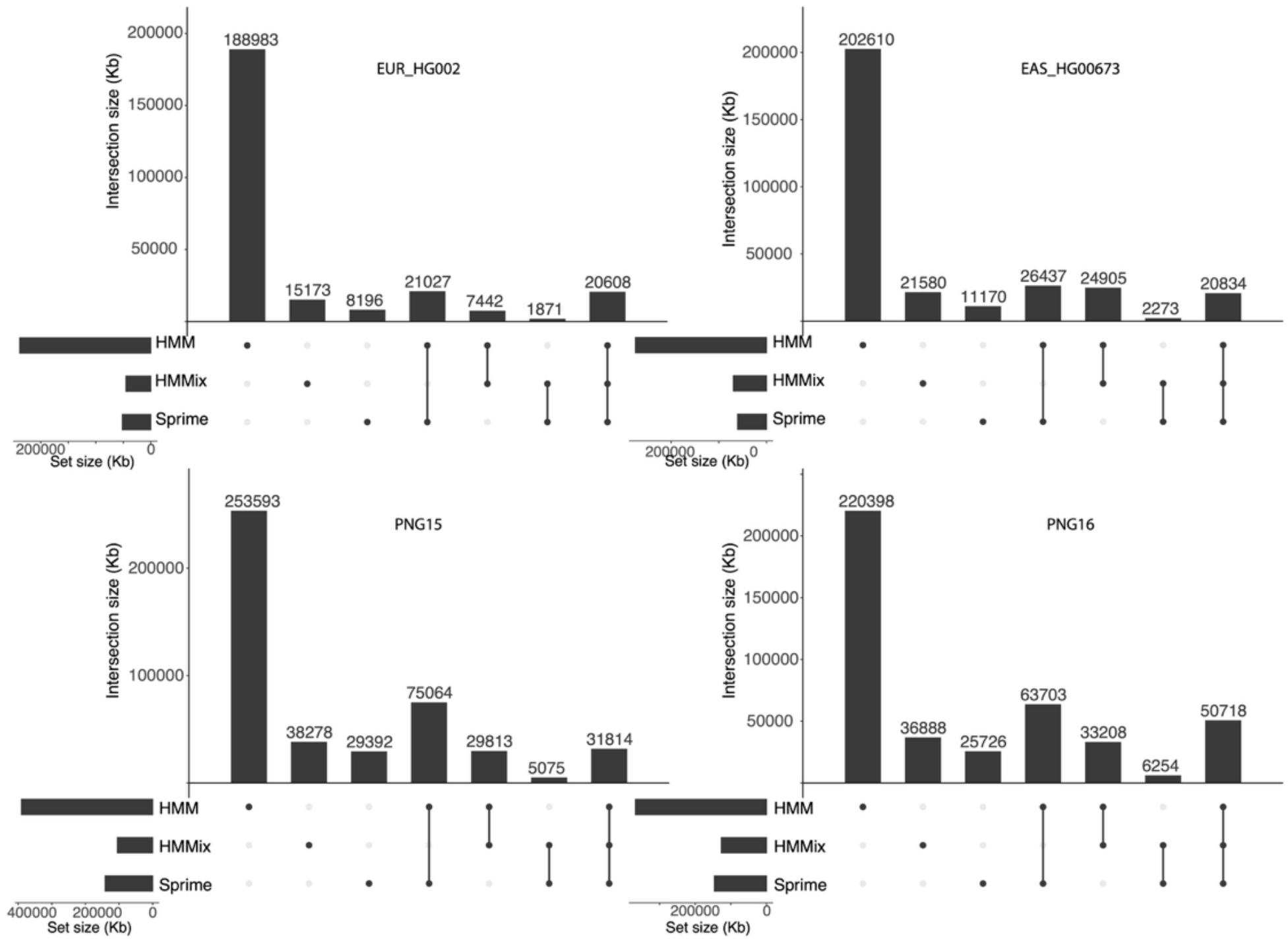

Fig. S29. Assessment of agreement among introgressed bases called by the three methods, including HMM (Seguin-Orlando et al., 2014), Sprime (Browning et al., 2018), and hmmix (Skov et al., 2018). Upset plots showing amounts of introgressed sequences identified by one, two, or three callers in the HG002 (European ancestry), HG00673 (Chinese Han South ancestry), PNG15, and PNG16 genomes.

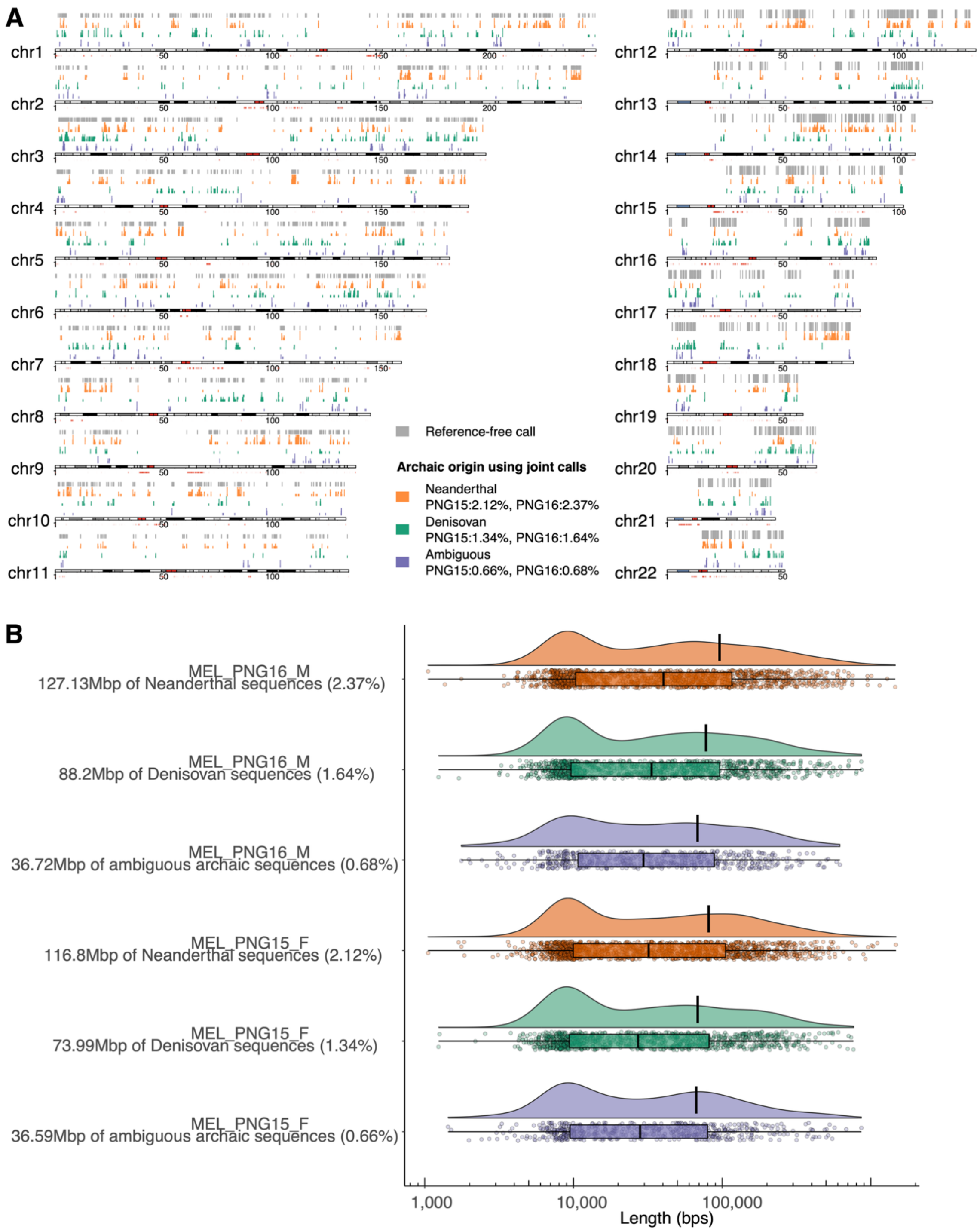

Fig. S30. Summary of archaic introgressed segments in PNG15 and PNG16. (A) The distribution of introgressed segments across the genome is illustrated, with segments identified by at least two of the three call sets (hmmix, Sprime and HMM-based methods) highlighted in color. Gray segments are from reference-free call sets (hmmix and Sprime). Red ticks below individual ideograms are known SDs in the reference genome GRCh38. (B) The length distributions of introgressed segments, categorized by their putative archaic origins. Each boxplot represents the median (the thick line in the box), the upper and lower quartiles (depicted by the box), and the 1.5× interquartile range (shown by the whiskers).

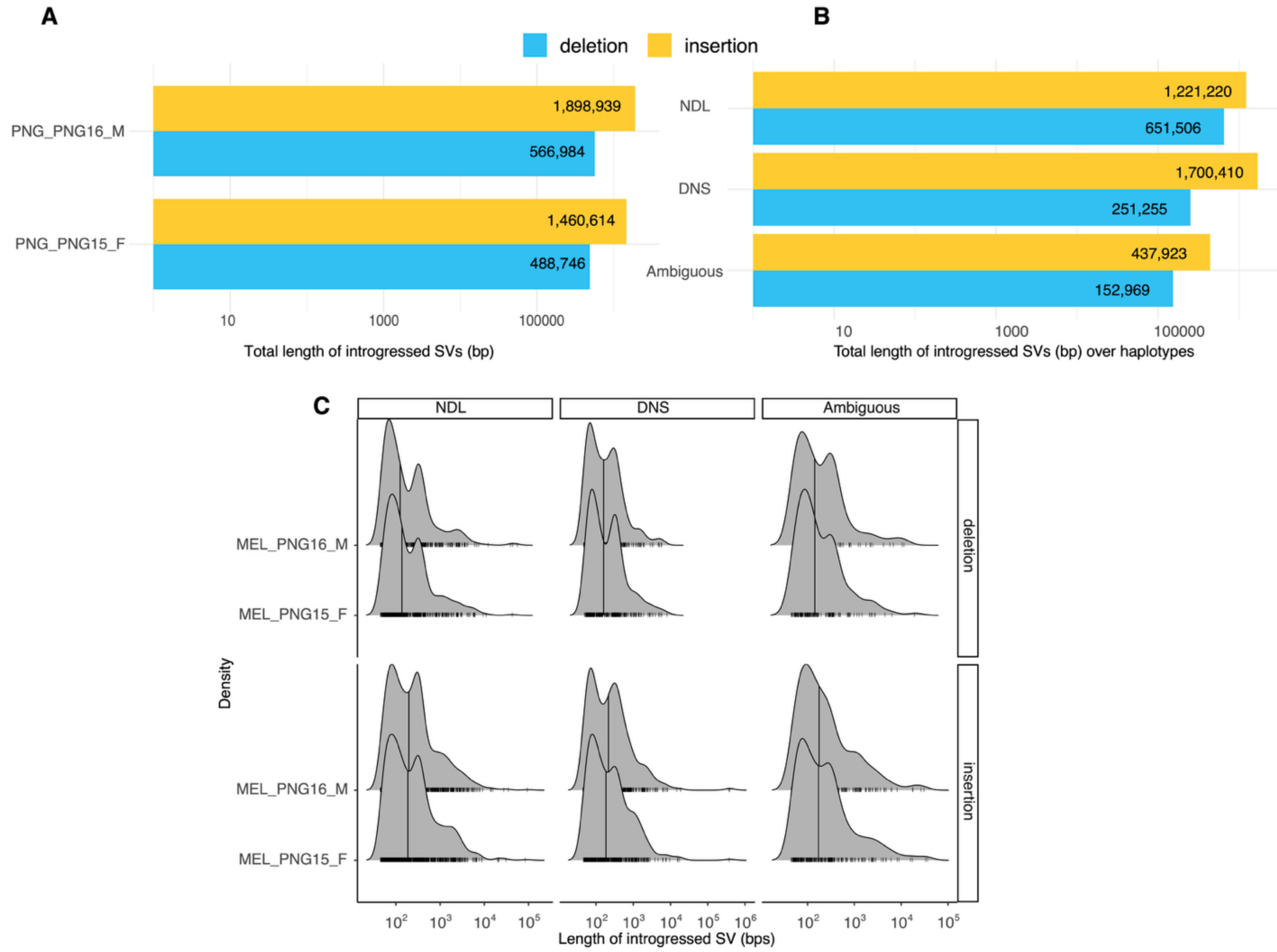

Fig. S31. Summary of introgressed SV alleles in PNG15 and PNG16. (A) Total lengths of introgressed deletion and insertion alleles in individual samples. (B) Total lengths of introgressed deletion and insertion alleles, categorized by the origins of introgression. (C) Comparison of length distributions of introgressed alleles across samples, stratified by the SV type and origin of introgression. All x-axes are on a base-10 logarithmic scale. NDL: Neanderthal. DNS: Denisovan.

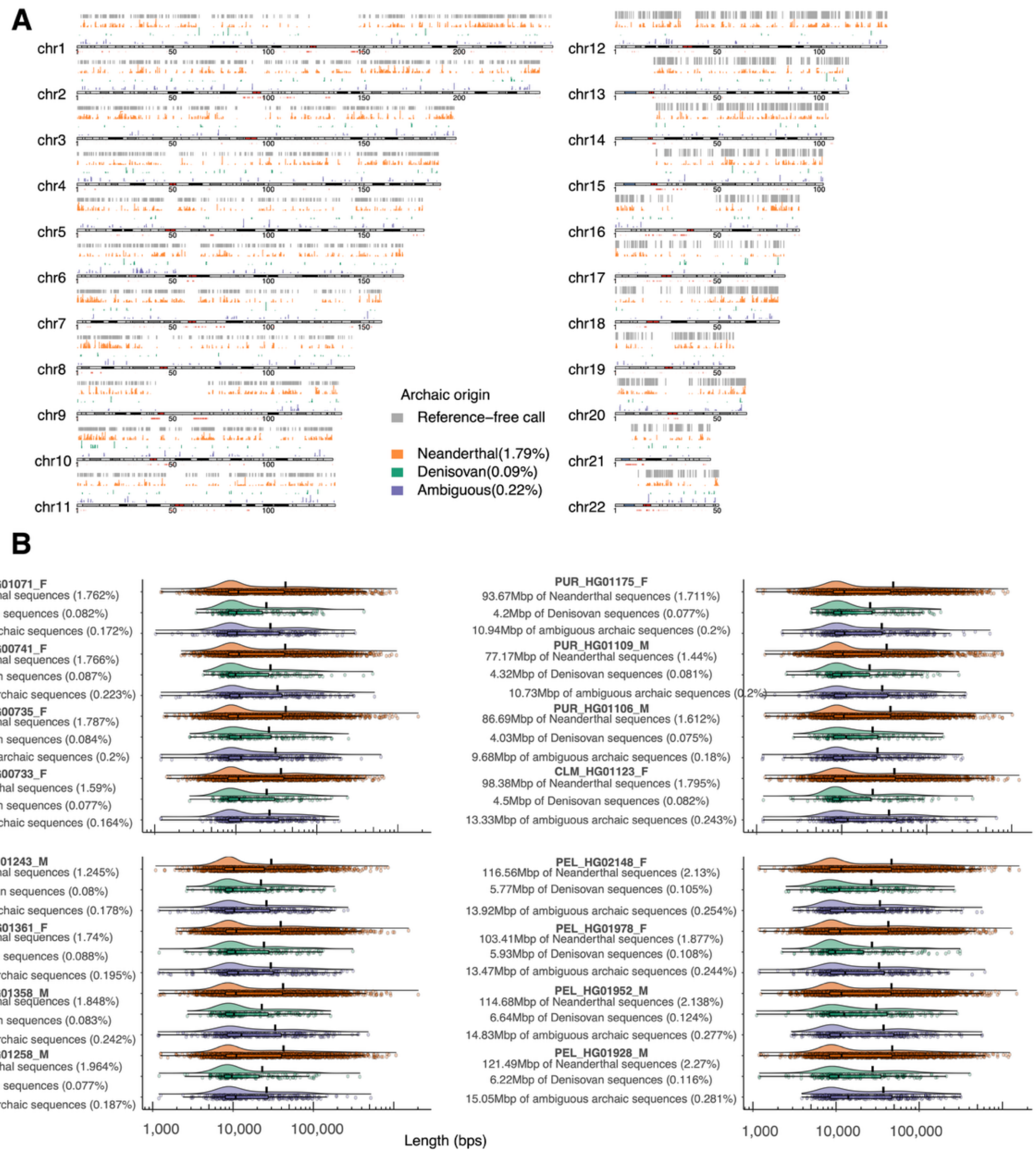

Fig. S32. Summary of introgressed segments in the admixed American (AMR) samples in HPRCr1. (A) The distribution of introgressed segments across the genome is illustrated, with segments identified by at least two of the three call sets (hmmix, Sprime and HMM-based methods) highlighted in color. Gray segments are from reference-free call sets (hmmix and Sprime). Red ticks below individual ideograms are known SDs in the reference genome GRCh38. (B) The length distributions of introgressed segments, categorized by their putative archaic origins. Each boxplot represents the median (the thick line in the box), the upper and lower quartiles (depicted by the box), and the 1.5× interquartile range (shown by the whiskers).

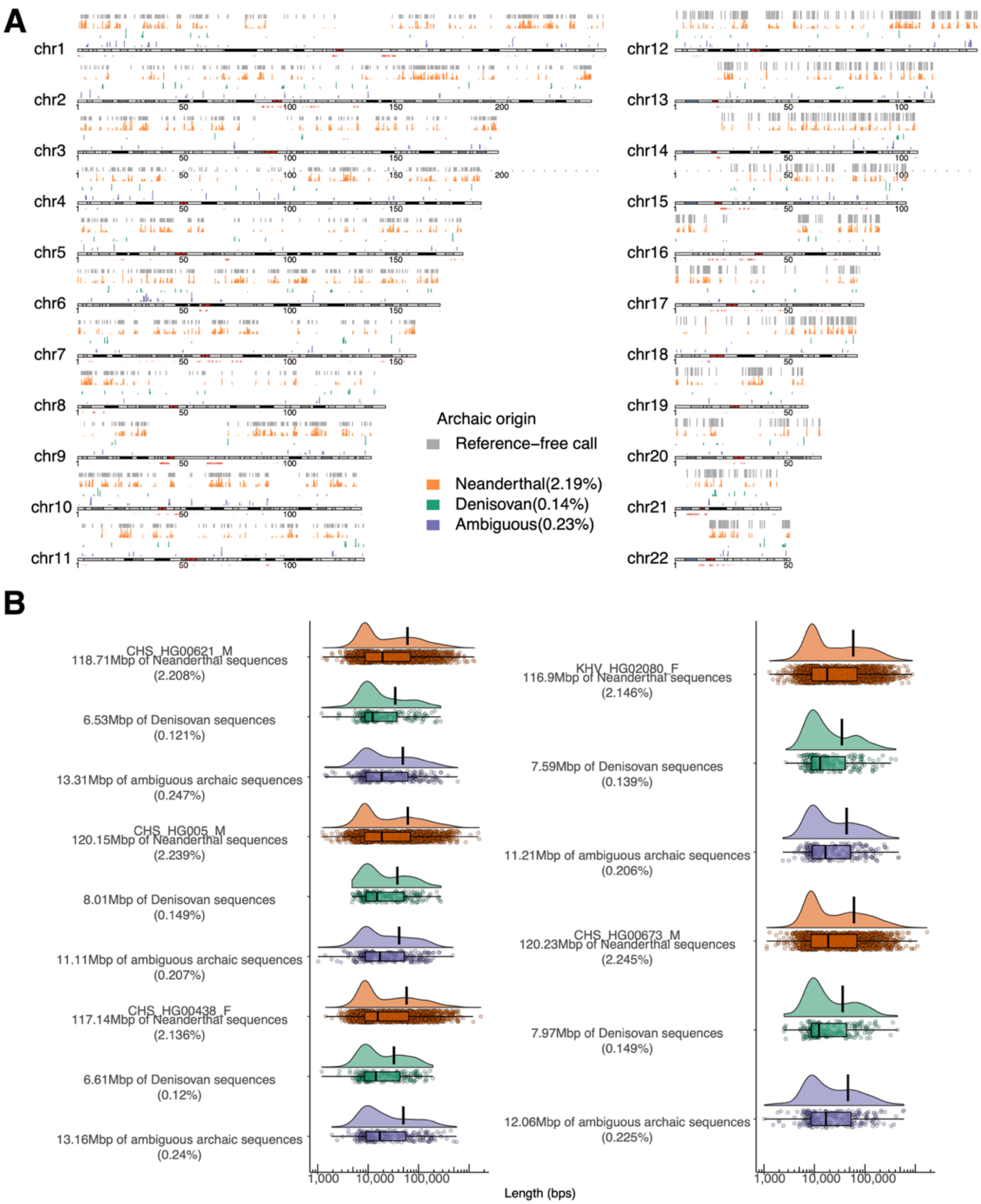

Fig. S33. Summary of introgressed segments in East Asian (EAS) samples in the HPRCr1 cohort. (A) The distribution of introgressed segments across the genome is illustrated, with segments identified by at least two of the three call sets (hmmix, Sprime and HMM-based methods) highlighted in color. Gray segments are from reference-free call sets (hmmix and Sprime). Red ticks below individual ideograms are known SDs in the reference genome GRCh38. (B) The length distributions of introgressed segments, categorized by their putative archaic origins. Each boxplot represents the median (the thick line in the box), the upper and lower quartiles (depicted by the box), and the 1.5× interquartile range (shown by the whiskers).

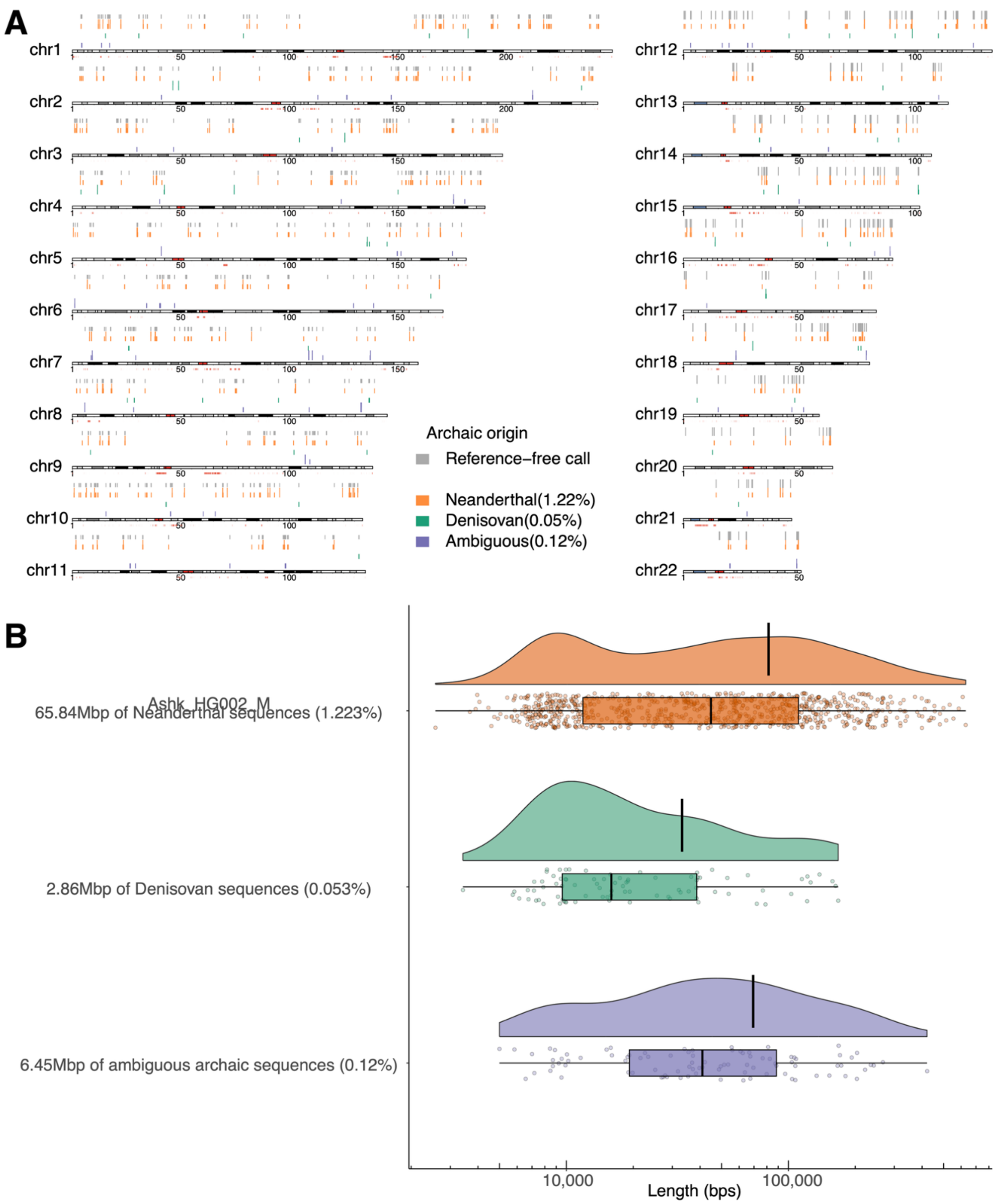

Fig. S34. Summary of introgressed segments in the European-descent/Ashkenazi sample in the HPRCr1 cohort. (A) The distribution of introgressed segments across the genome is illustrated, with segments identified by at least two of the three call sets (hmmix, Sprime and HMM-based methods) highlighted in color. Gray segments are from reference-free call sets (hmmix and Sprime). Red ticks below individual ideograms are known SDs in the reference genome GRCh38. (B) The length distributions of introgressed segments, categorized by their putative archaic origins. Each boxplot represents the median (the thick line in the box), the upper and lower quartiles (depicted by the box), and the 1.5× interquartile range (shown by the whiskers).

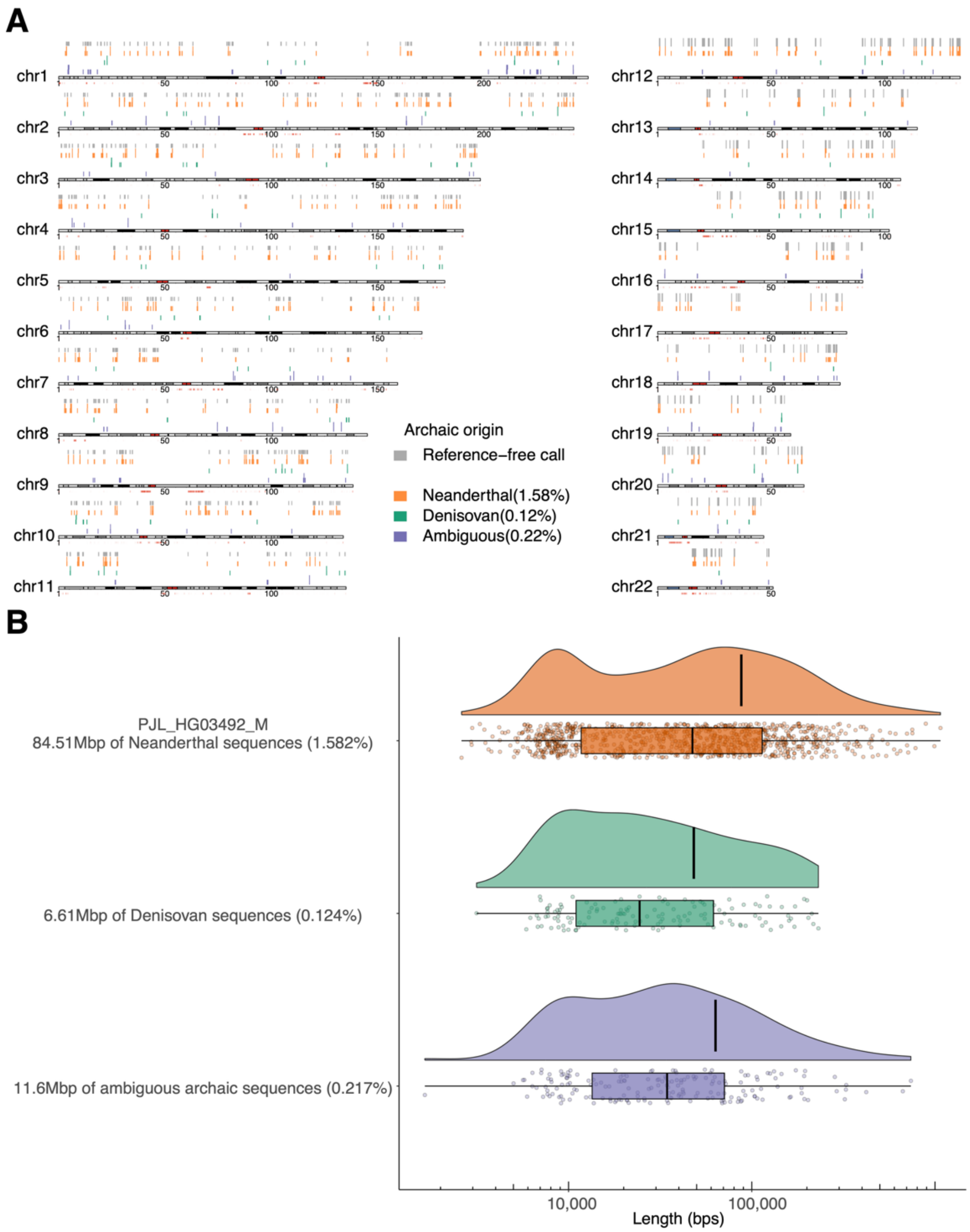

Fig. S35. Summary of introgressed segments in South Asian (SAS) sample in the HPRCr1 cohort. (A) The distribution of introgressed segments across the genome is illustrated, with segments identified by at least two of the three call sets (hmmix, Sprime and HMM-based methods) highlighted in color. Gray segments are from reference-free call sets (hmmix and Sprime). Red ticks below individual ideograms are known SDs in the reference genome GRCh38. (B) Length Distributions of Introgressed Segments by Putative Archaic Origin: This panel displays the length distributions of introgressed segments, categorized by their putative archaic origins. Each boxplot represents the median (the thick line in the box), the upper and lower quartiles (depicted by the box), and the 1.5× interquartile range (shown by the whiskers). Red ticks below individual ideograms are known SDs in the reference genome GRCh38.

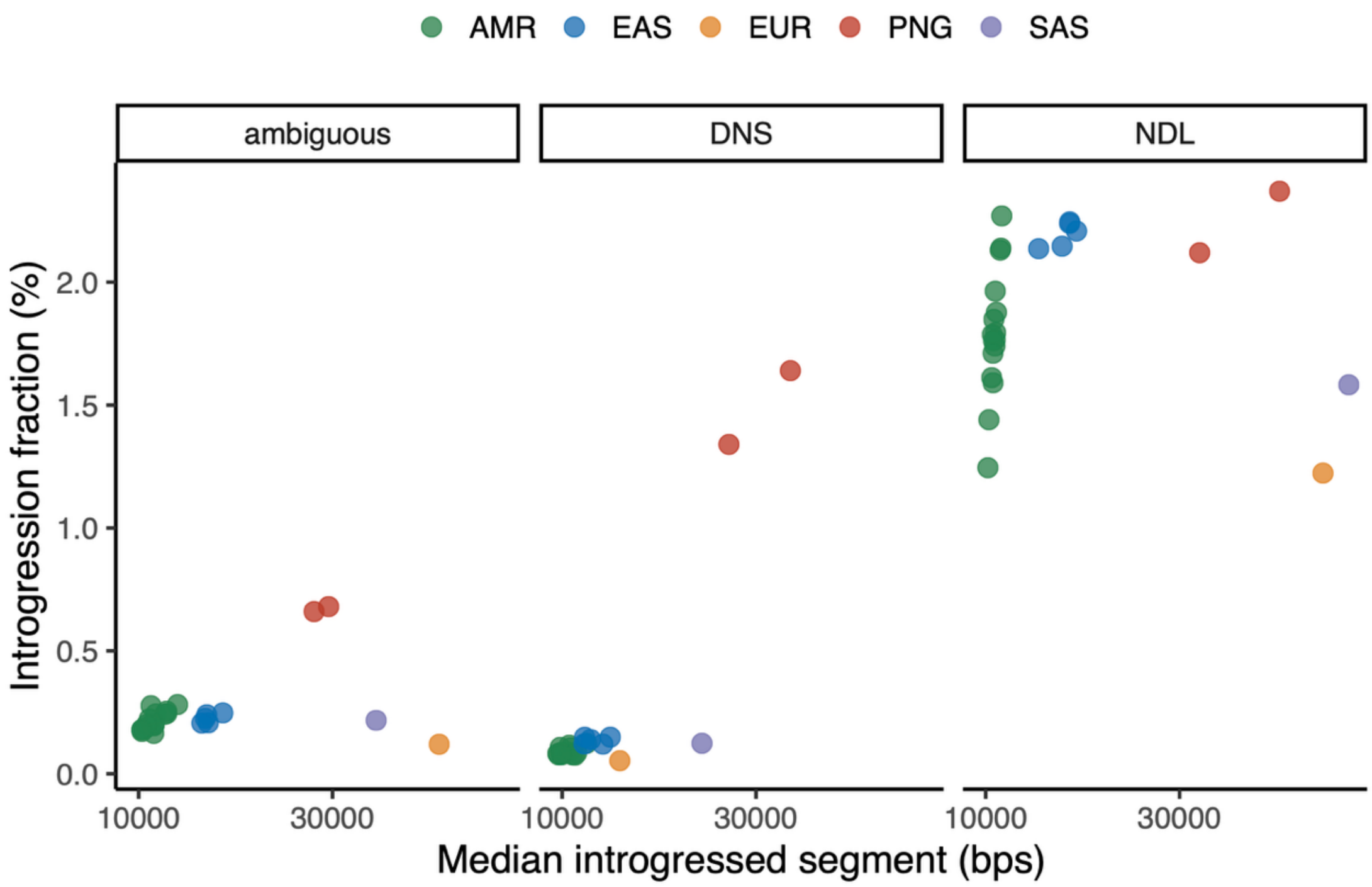

Fig. S36. Comparison of introgressed segments in length and proportion of introgressed DNA across PNG and HPRCr1 non-African genomes, stratified by the putative origin of introgressed segments. Each dot represents a diploid genome, with colors indicating the continental group.

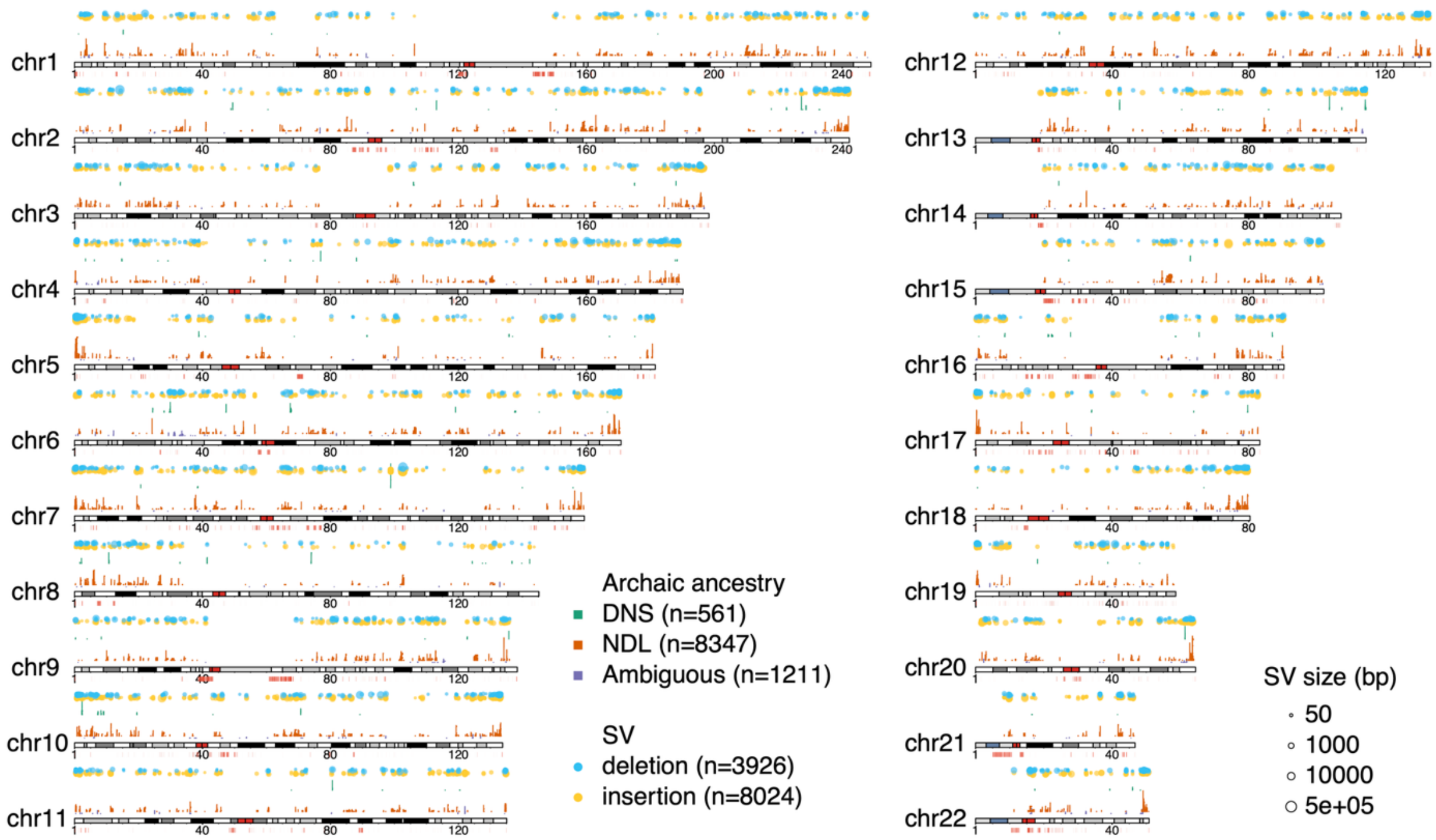

Fig. S37. Genome-wide distribution of introgressed SVs in the AMR samples. Introgressed deletion and insertion alleles are represented as circles, with circle size reflecting the length of each SV. Vertical bars indicate the corresponding introgressed segments, with colors denoting their origins of introgression. Red ticks below individual ideograms are known SDs in the reference genome GRCh38.

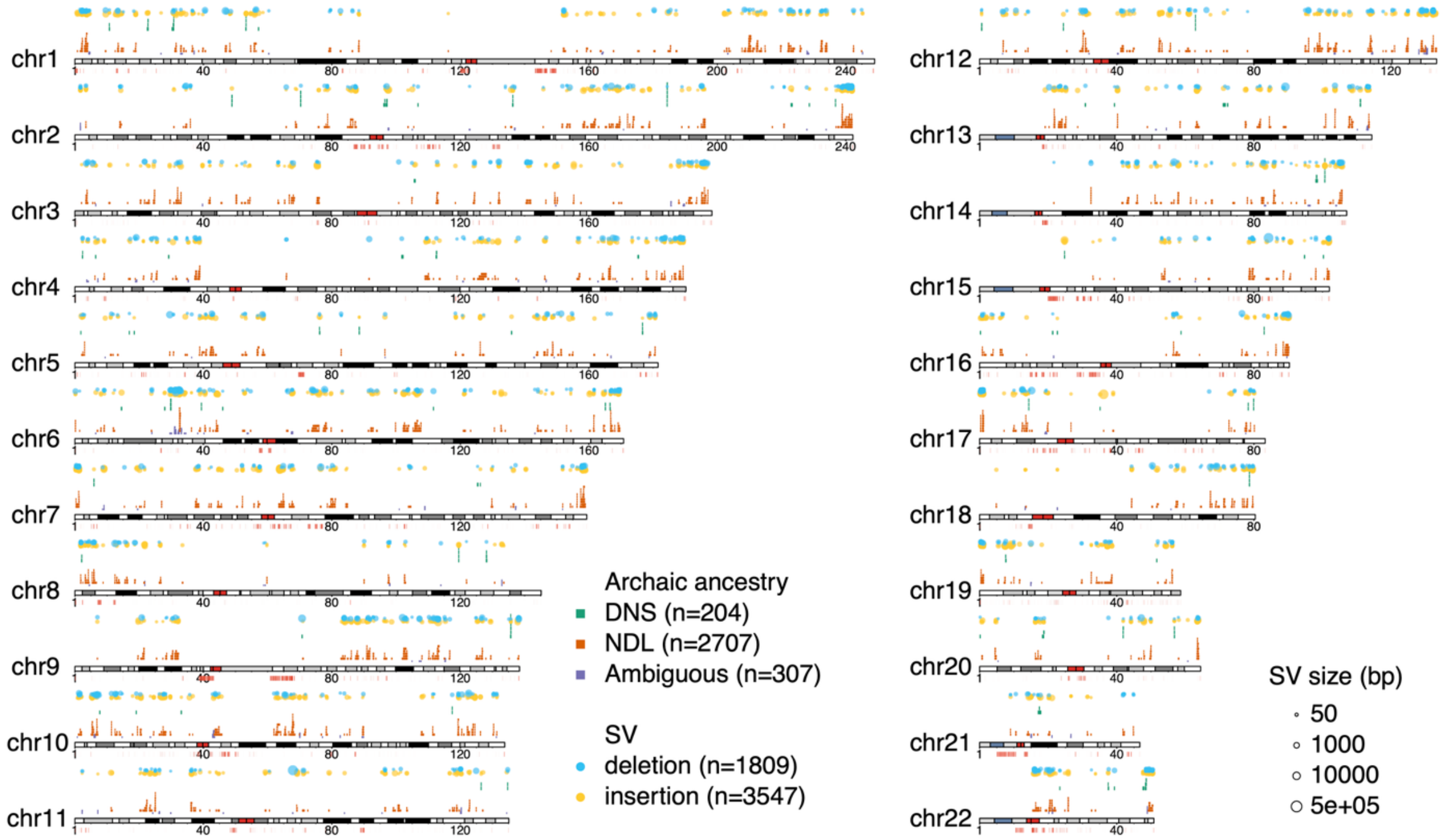

Fig. S38. Genome-wide distribution of introgressed SVs in the EAS samples. Introgressed deletion and insertion alleles are represented as circles, with circle size reflecting the length of each SV. Vertical bars indicate the corresponding introgressed segments, with colors denoting their origins of introgression. Red ticks below individual ideograms are known SDs in the reference genome GRCh38.

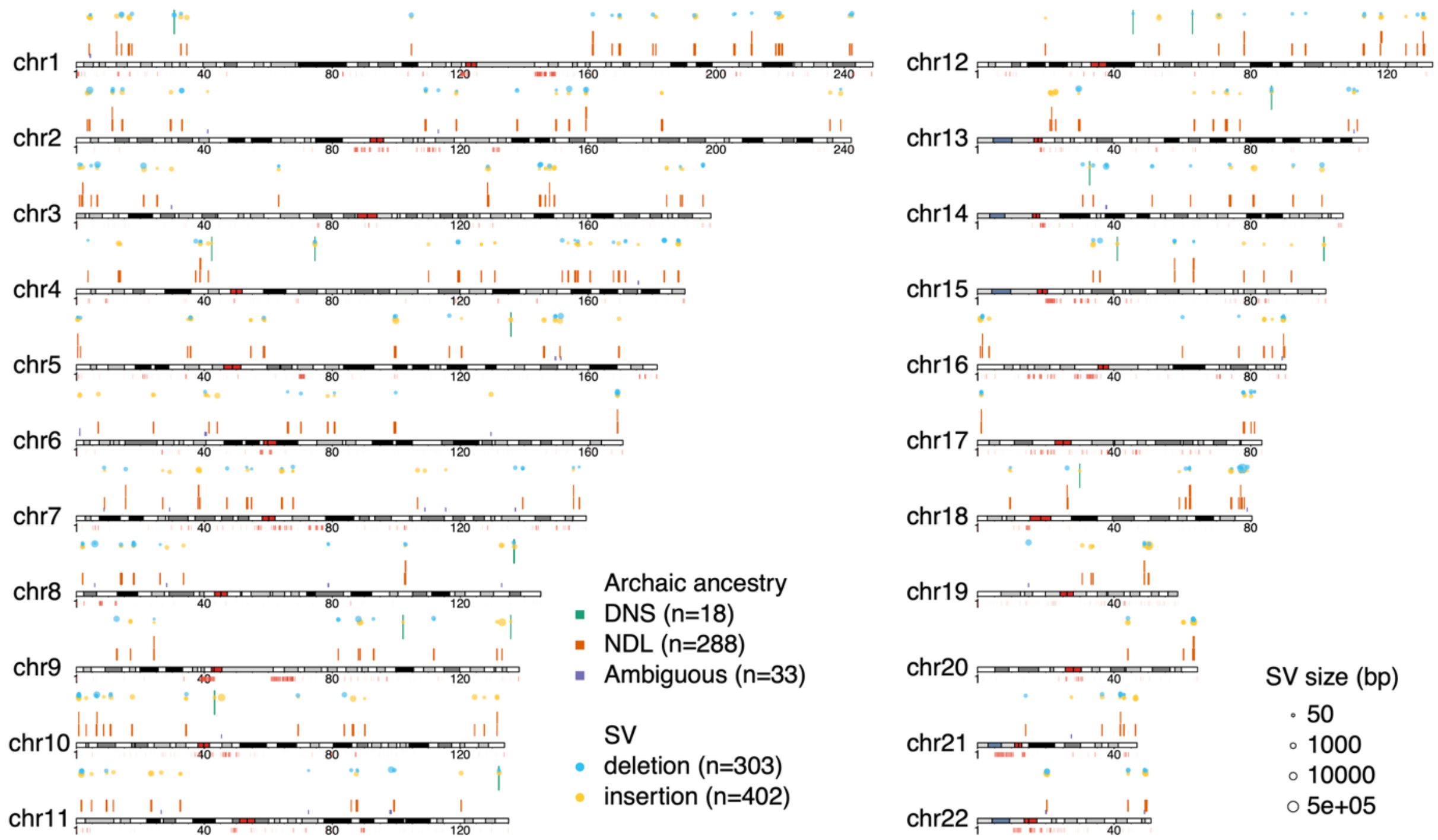

Fig. S39. Genome-wide distribution of introgressed SVs in the EUR sample. Introgressed deletion and insertion alleles are represented as circles, with circle size reflecting the length of each SV. Vertical bars indicate the corresponding introgressed segments, with colors denoting their origins of introgression. Red ticks below individual ideograms are known SDs in the reference genome GRCh38.

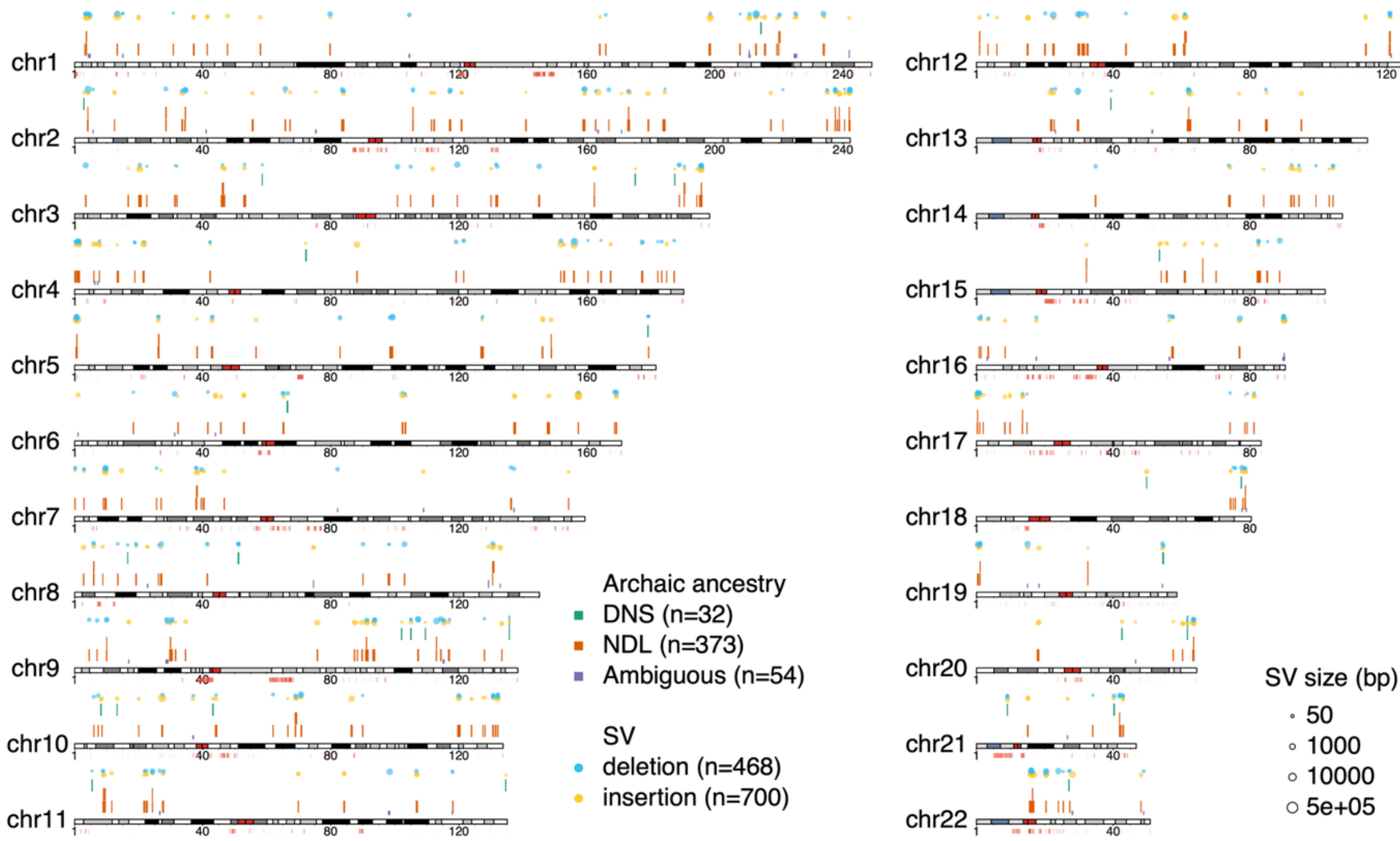

Fig. S40. Genome-wide distribution of introgressed SVs in the SAS sample. Introgressed deletion and insertion alleles are represented as circles, with circle size reflecting the length of each SV. Vertical bars indicate the corresponding introgressed segments, with colors denoting their origins of introgression. Red ticks below individual ideograms are known SDs in the reference genome GRCh38.

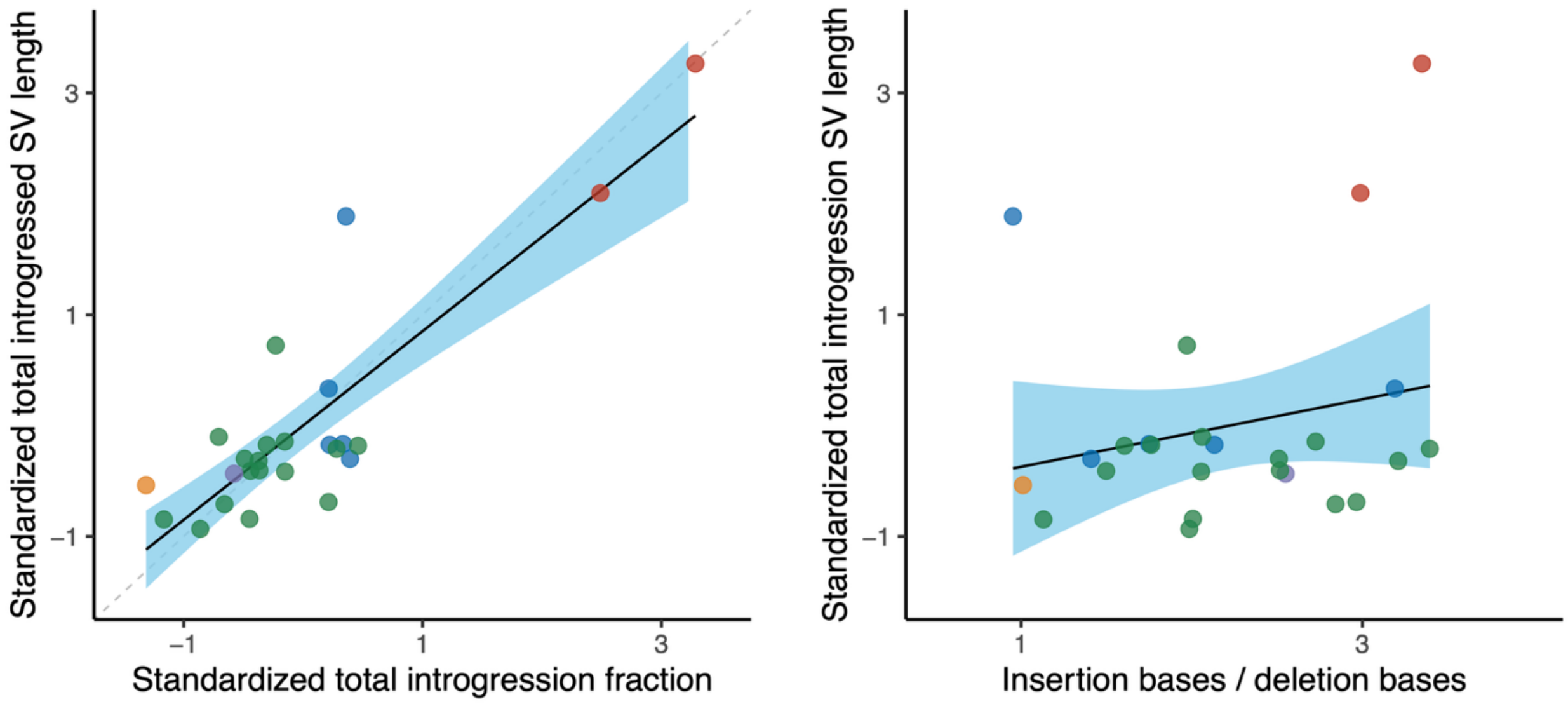

Fig. S41. Comparisons of the amount of introgressed SV bases and overall introgression fractions across PNG and HPRCr1 non-African samples. Each point is a diploid genome. (Left) A strong correlation is observed between the total base pairs of introgressed SV alleles and the introgression fraction across PNG and HPRCr1 non-African samples. The black line represents the linear regression, and the blue shaded area indicates the 95% confidence interval for the prediction. The gray dashed line denotes the diagonal. (Right) There is a higher number of introgressed insertion bases compared to introgressed deletion bases. Standardization of a measurement is computed by subtracting the mean and then dividing the difference by the standard deviation.

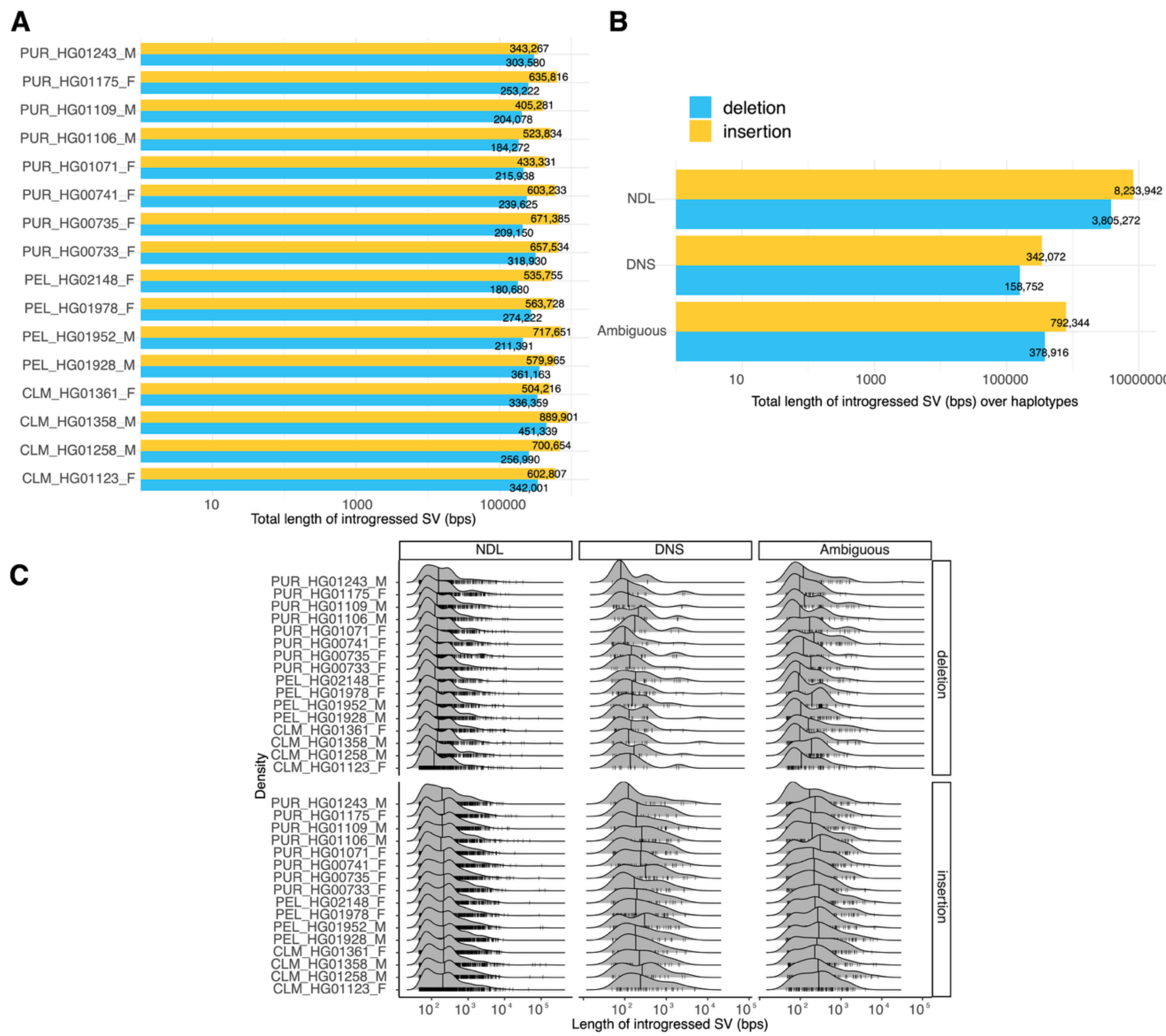

Fig. S42. Summary of introgressed SV alleles in AMR samples in the HPRCr1 cohort. (A) Total lengths of introgressed deletion and insertion alleles in individual samples. (B) Total lengths of introgressed deletion and insertion alleles, categorized by the origins of introgression. (C) Comparison of length distributions of introgressed alleles across samples, stratified by the SV type and origin of introgression. All x-axes are on a base-10 logarithmic scale.

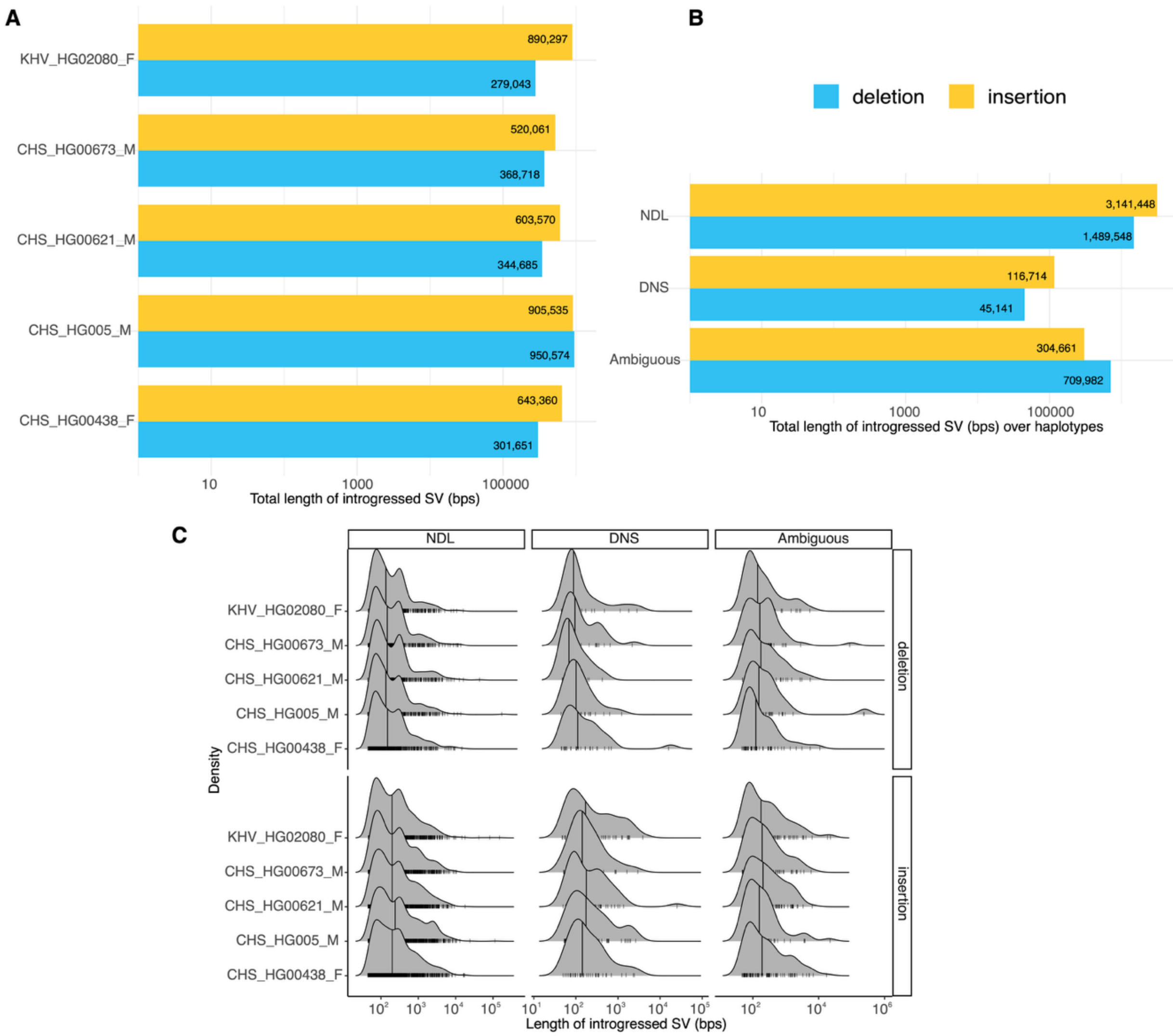

Fig. S43. Summary of introgressed SV alleles in the EAS samples in the HPRCr1 cohort. (A) Total lengths of introgressed deletion and insertion alleles in individual samples. (B) Total lengths of introgressed deletion and insertion alleles, categorized by the origins of introgression. (C) Comparison of length distributions of introgressed alleles across samples, stratified by the SV type and origin of introgression. All x-axes are on a base-10 logarithmic scale.

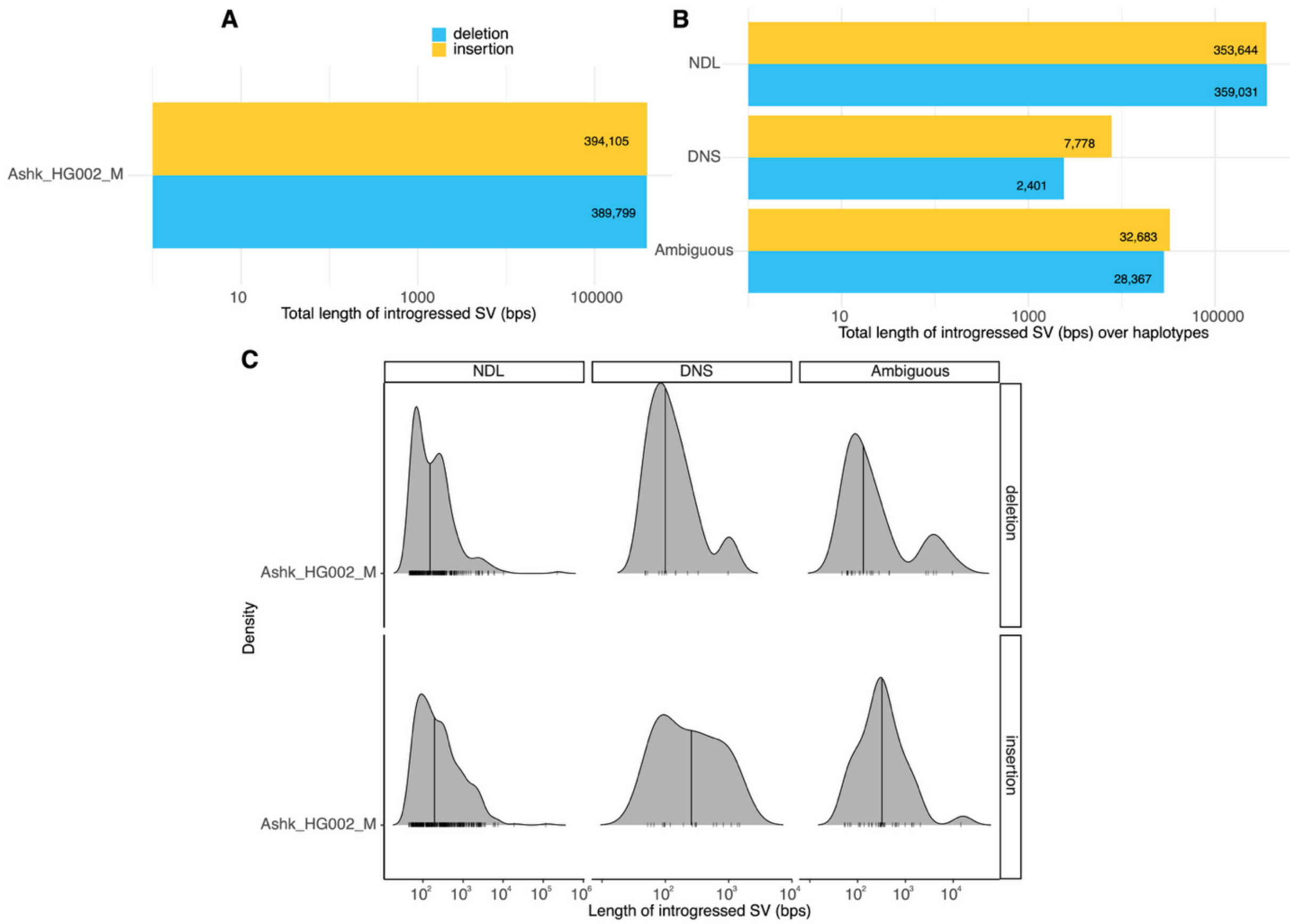

Fig. S44. Summary of introgressed SV alleles in the EUR sample in the HPRCr1 cohort. (A) Total lengths of introgressed deletion and insertion alleles in individual samples. (B) Total lengths of introgressed deletion and insertion alleles, categorized by the origins of introgression. (C) Comparison of length distributions of introgressed alleles across samples, stratified by the SV type and origin of introgression. All x-axes are on a base-10 logarithmic scale.

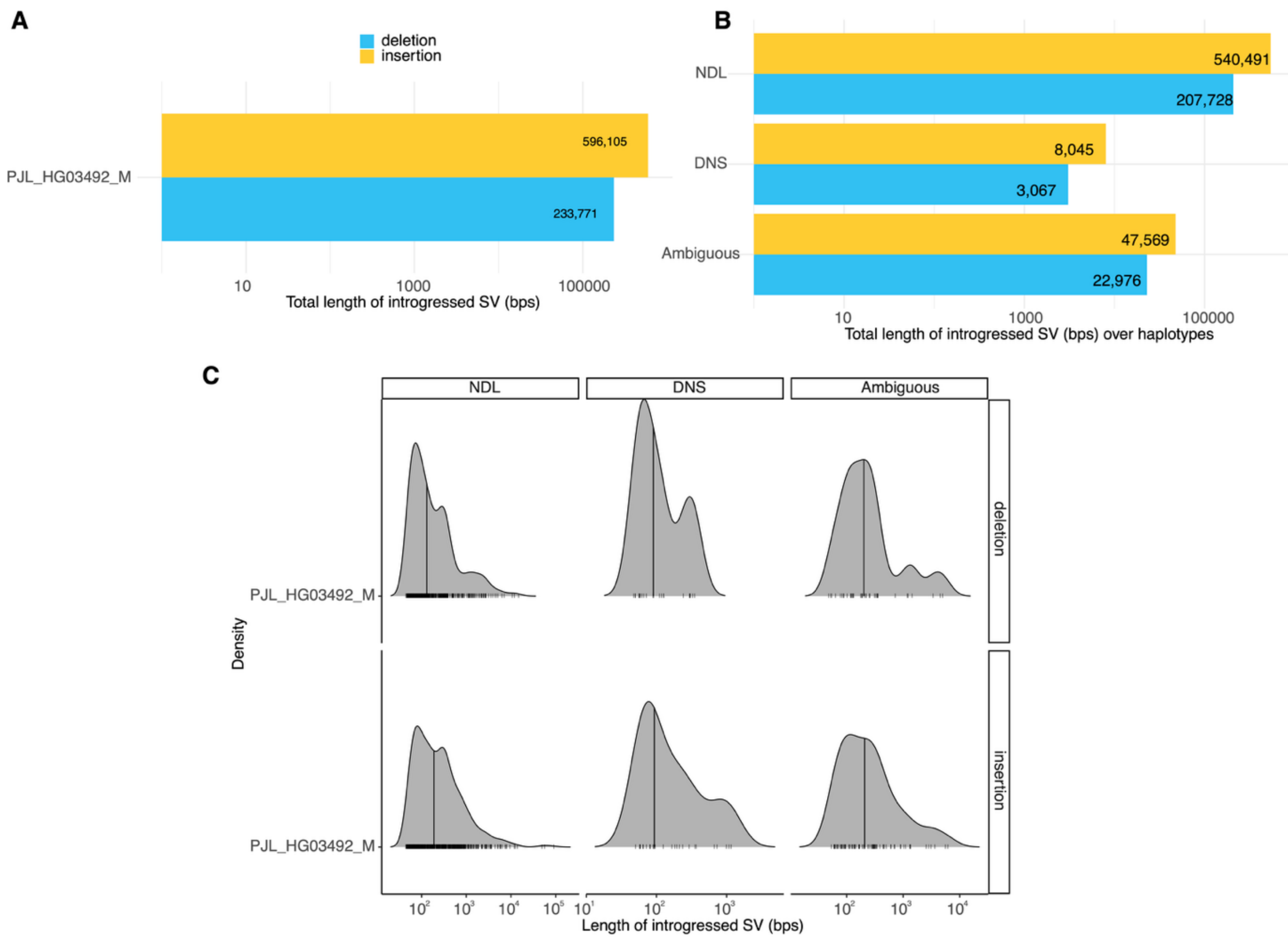

Fig. S45. Summary of introgressed SV alleles in the SAS sample in the HPRCr1 cohort. (A) Total lengths of introgressed deletion and insertion alleles in individual samples. (B) Total lengths of introgressed deletion and insertion alleles, categorized by the origins of introgression. (C) Comparison of length distributions of introgressed alleles across samples, stratified by the SV type and origin of introgression. All x-axes are on a base-10 logarithmic scale.

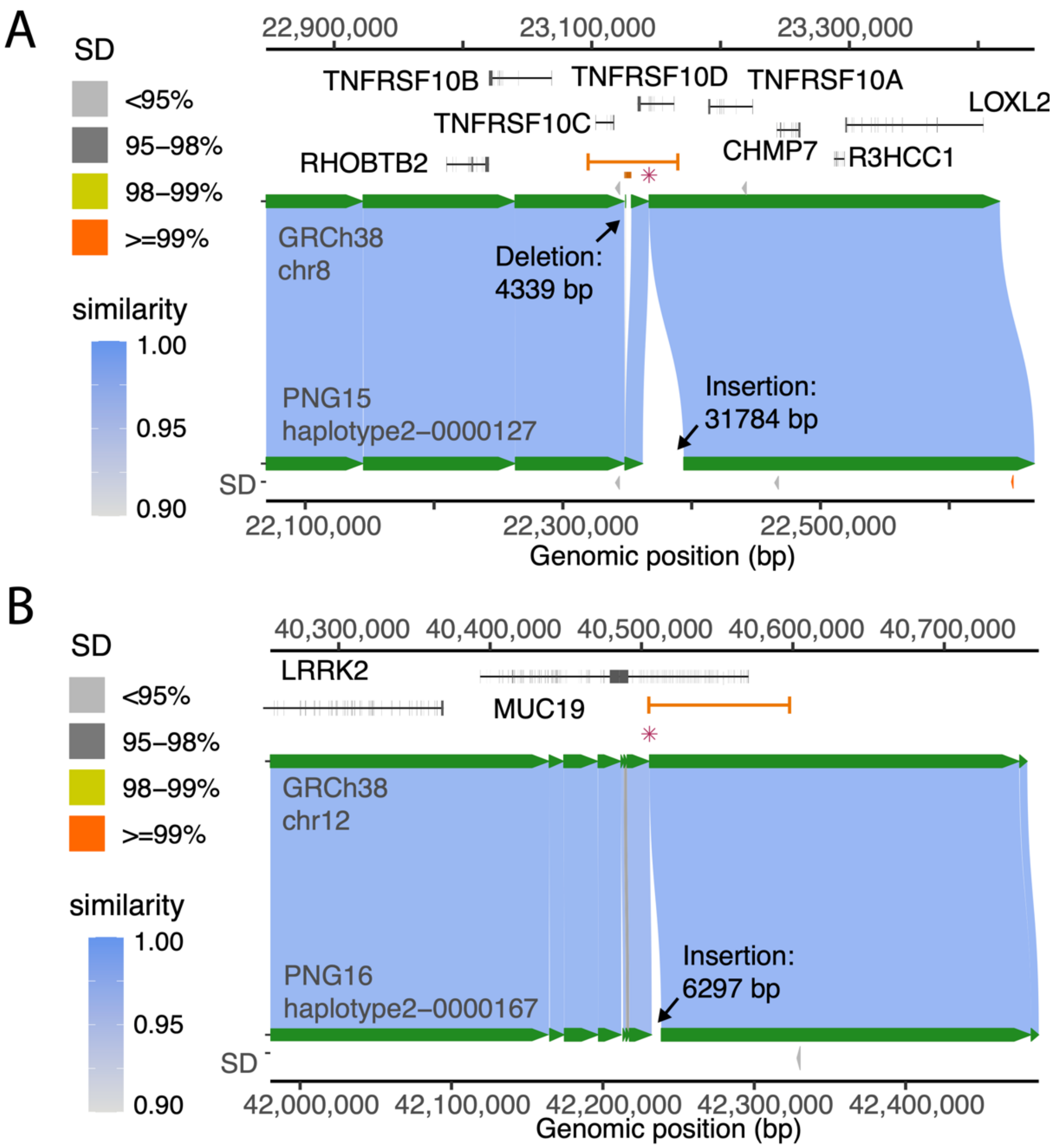

Fig. S46. Examples of previously reported archaic introgressed SV SDs in the newly assembled PNG haplotypes. Shown are the (A) *TNFRSF10D* locus and (B) *MUC19* locus. In each plot, the top represents the GRCh38 reference genome aligned to the bottom, new genome assemblies. The gene annotation of the reference genome is shown at the top, followed by SDs predicted by WGAC (Methods); the respective direction is indicated by blue arrowheads. On the plots, a red asterisk indicates an insertion locus, while a rectangle shows the range of a deletion. The orange bars indicate the putative archaic sequences.

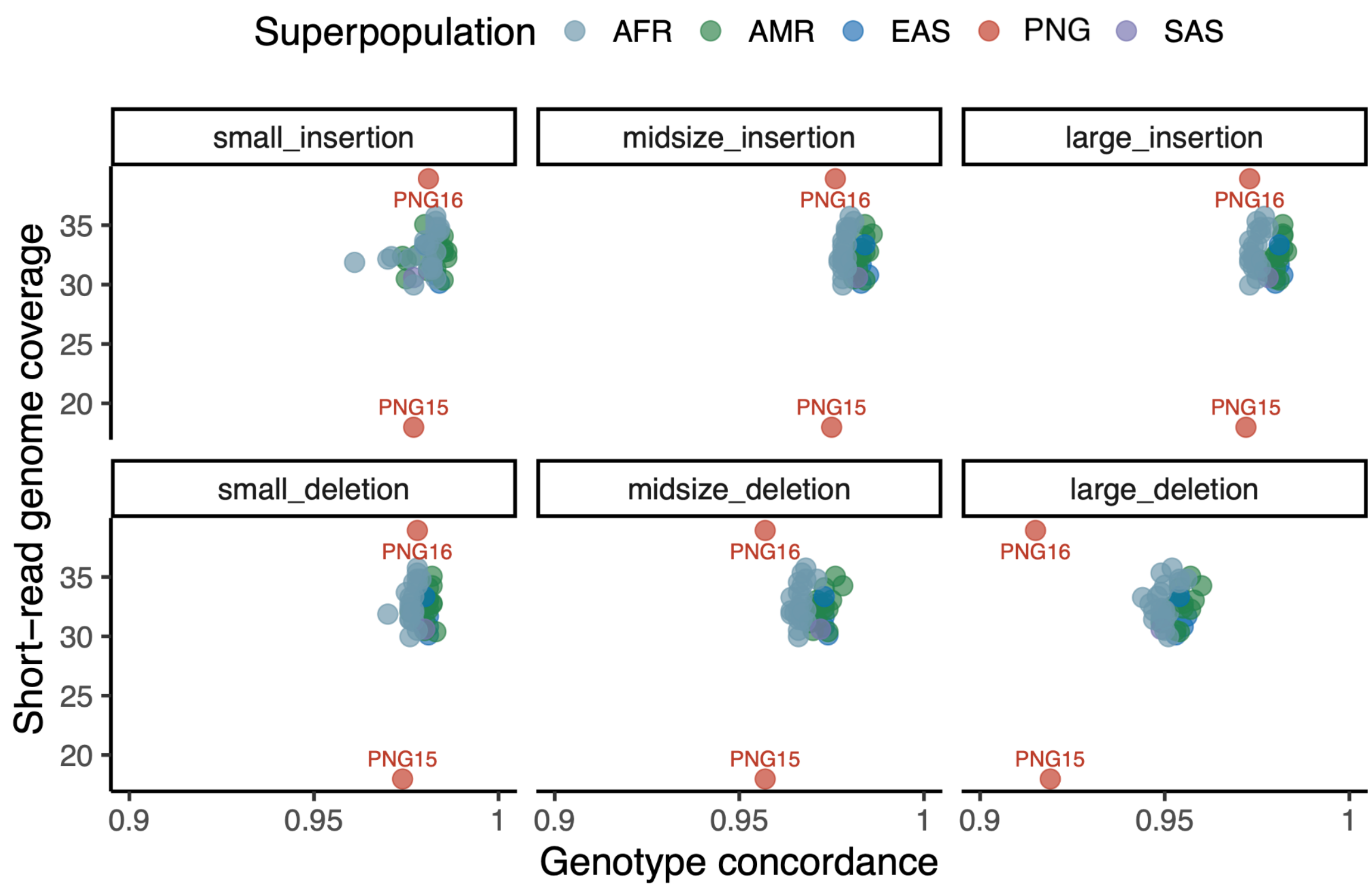

Fig. S47. Genotype concordance between PanGenie inferred and observed genotypes from the assemblies. Small variants: 0-24 bp, midsize variants: 25-49 bp, and large variants: ≥50 bp.

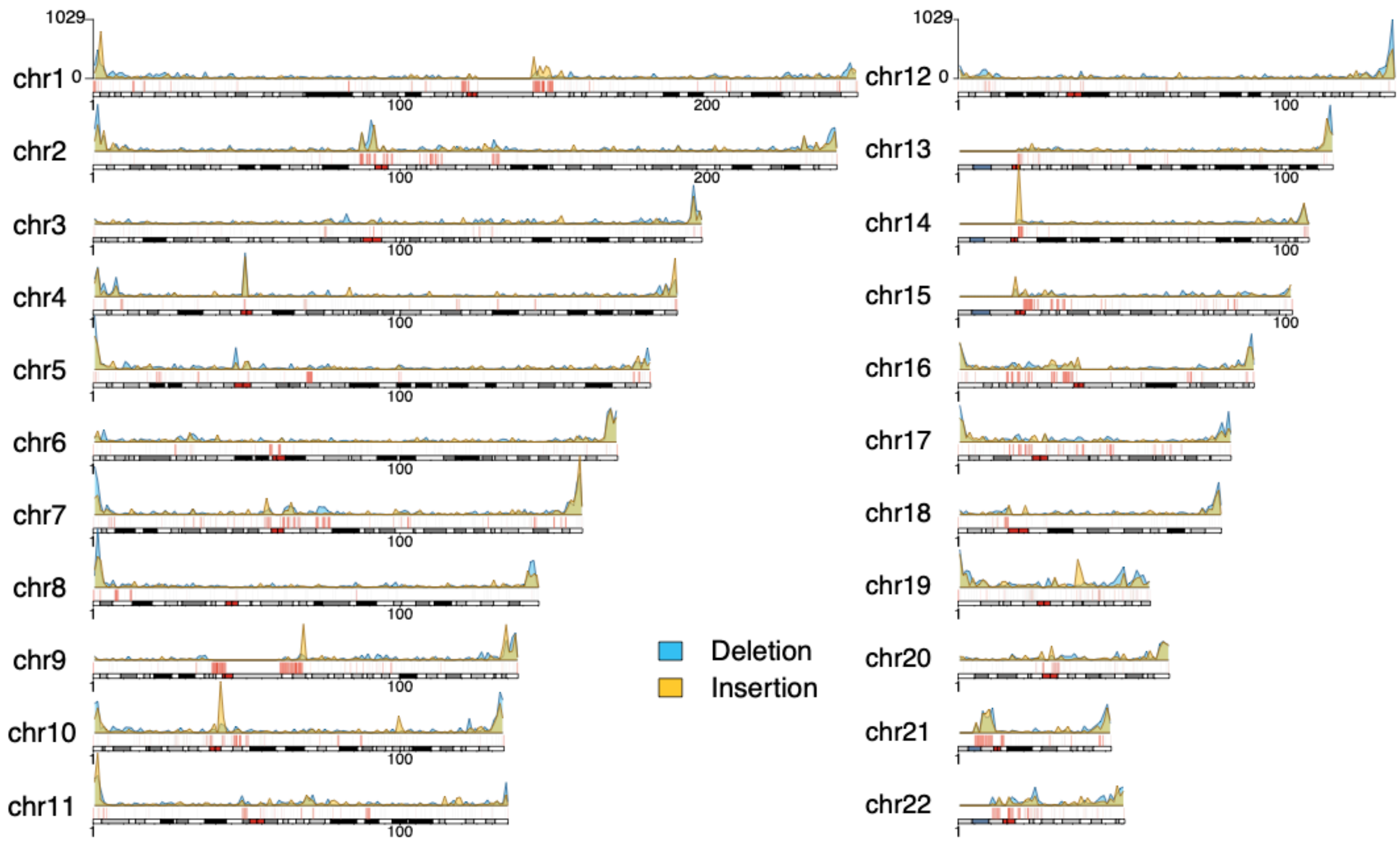

Fig. S48. Genome-wide discordant genotypes are correlated with complex genomic regions across HPRCr1 and PNG assemblies. Density plots show the locations of discordant genotypes. Red ticks above individual ideograms are the known SDs in the reference genome GRCh38.

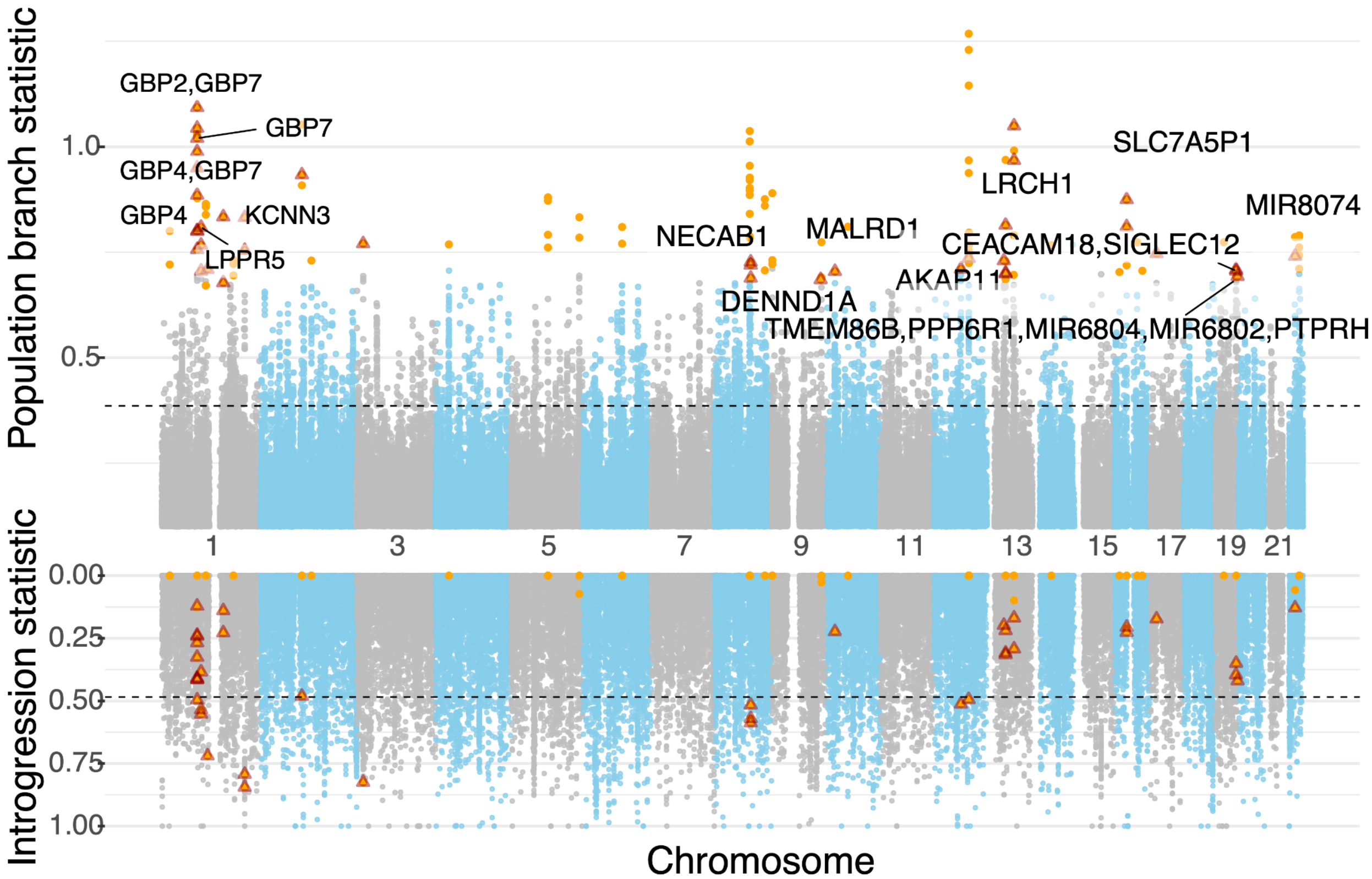

Fig. S49. Genome-wide Manhattan and Hudson plots for the adaptive introgression scans in the PNG (top: selection signals, bottom: introgression signals). The Manhattan plot illustrates the distribution of population branch statistics (*PBS*) from segments of 100 SNVs, while the Hudson plot depicts the f_D values for introgression signals. *PBS* values were computed using East Asian and African groups as the sister- and out-groups, respectively. Orange circles represent segments showing significant *PBS* values (p-value < 0.05, simulations based on 1,000 demographic models), while red triangles indicate evidence for introgression (p-value < 0.05, simulations based on 1,000 demographic models) using the Denisovan and Neanderthal as archaic reference genomes. Segments overlapping with genes are annotated with gene IDs.

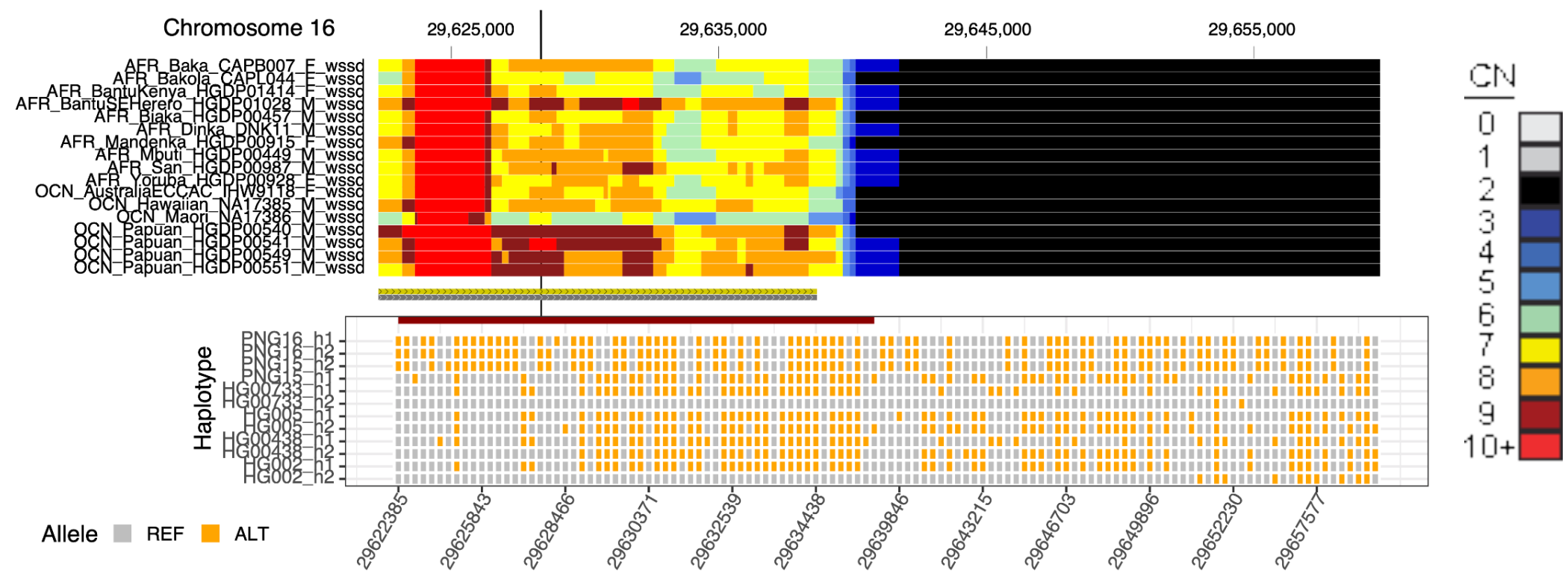

Fig. S50. Schematic representation of genomic variation at chr16p11.2 locus. Top panel: The heatmap shows the diploid copy number (CN) distribution for each individual (represented by a horizontal bar) at chromosome 16p11.2. The region with warm colors corresponds to highly identical SD sequences. Bottom panel: The distribution of allelic variation in the same region, with a red line indicating the corresponding SD region shown in the top panel. Note that the panels are not fully aligned or scaled.

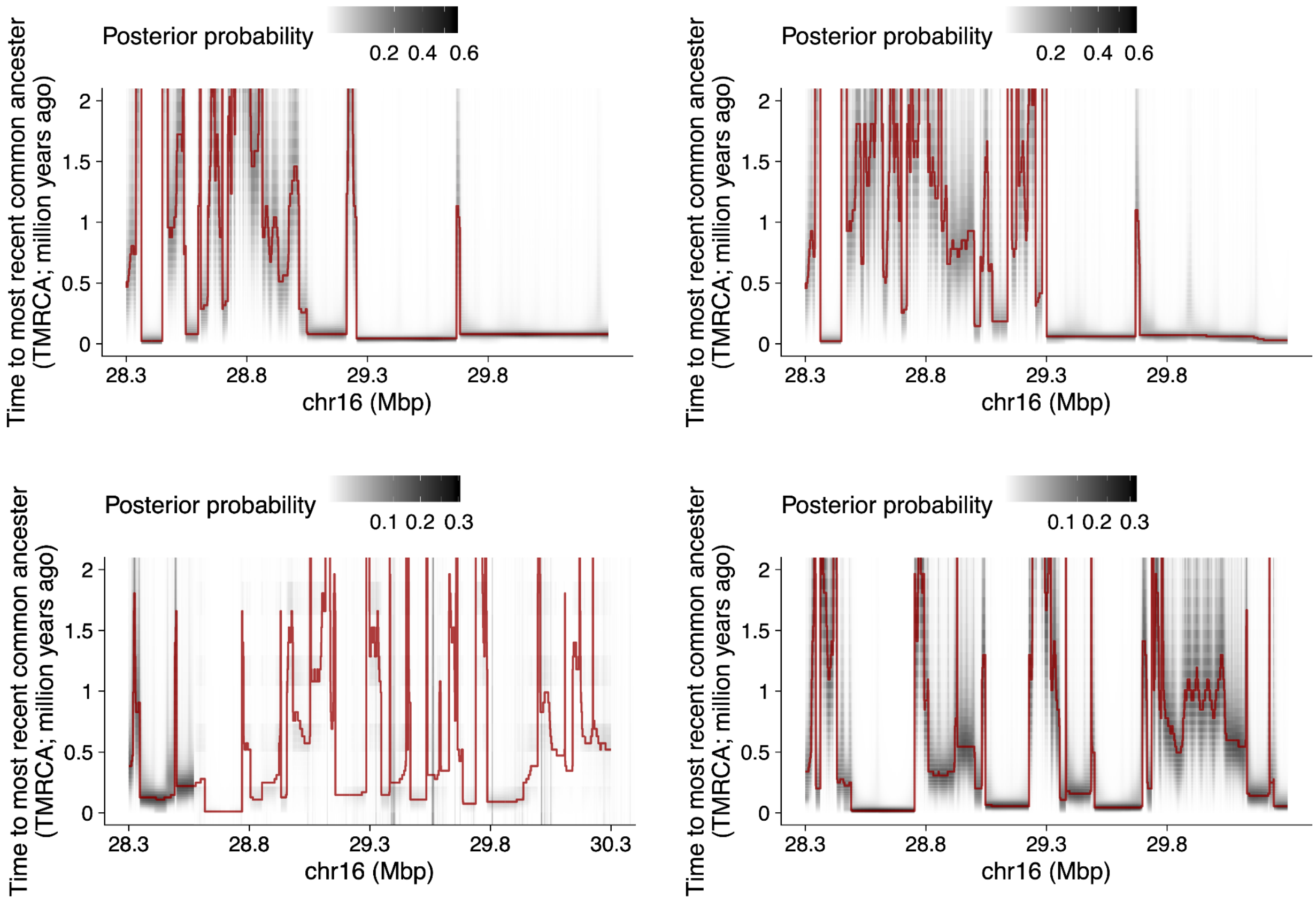

Fig. S51. Comparisons of pairwise coalescent decoding between homologous sequences at chr16p11.2. The heatmap shows the posterior probability distribution of piecewise time to most recent common ancestor (TMRCA). An enrichment for recent coalescence times in the distribution of TMRCAs along the sequence is consistent with positively selected loci. Top panels: PNG15_h2 vs. PNG16_h2 (left panel) and PNG16_h1 vs. PNG16_h2 (right panel). Bottom panels: HG02818_h1 vs. HG02818_h2 (Gambian in Western Division – Mandinka, Africa; left panel) and HG00621_h1 vs. HG00621_h2 (Han Chinese-South, East Asia; right panel).

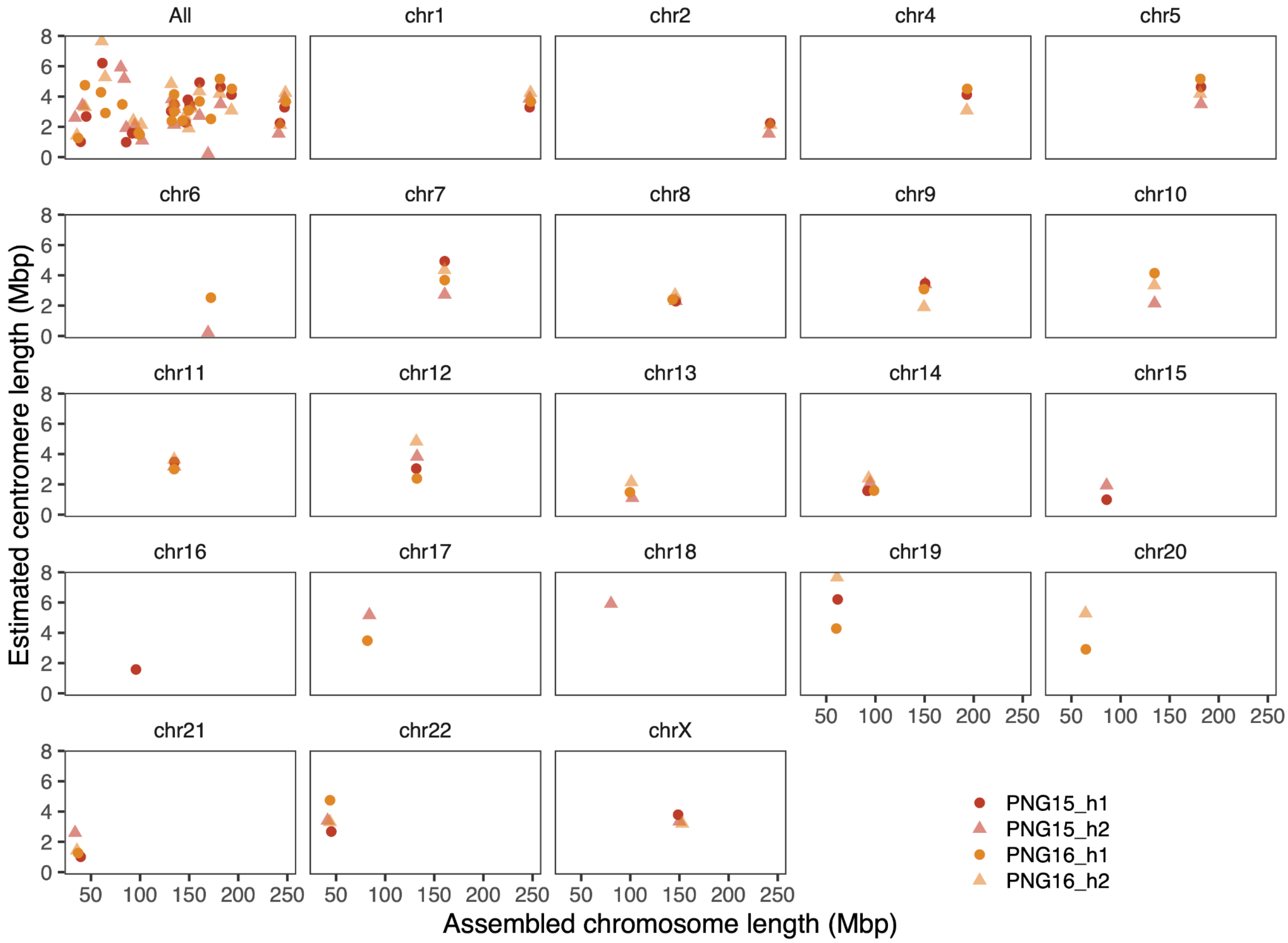

Fig. S52. No significant relationship between the length of an individual chromosome and its centromere size in the PNG samples. Only centromeres that are considered completely assembled are plotted.

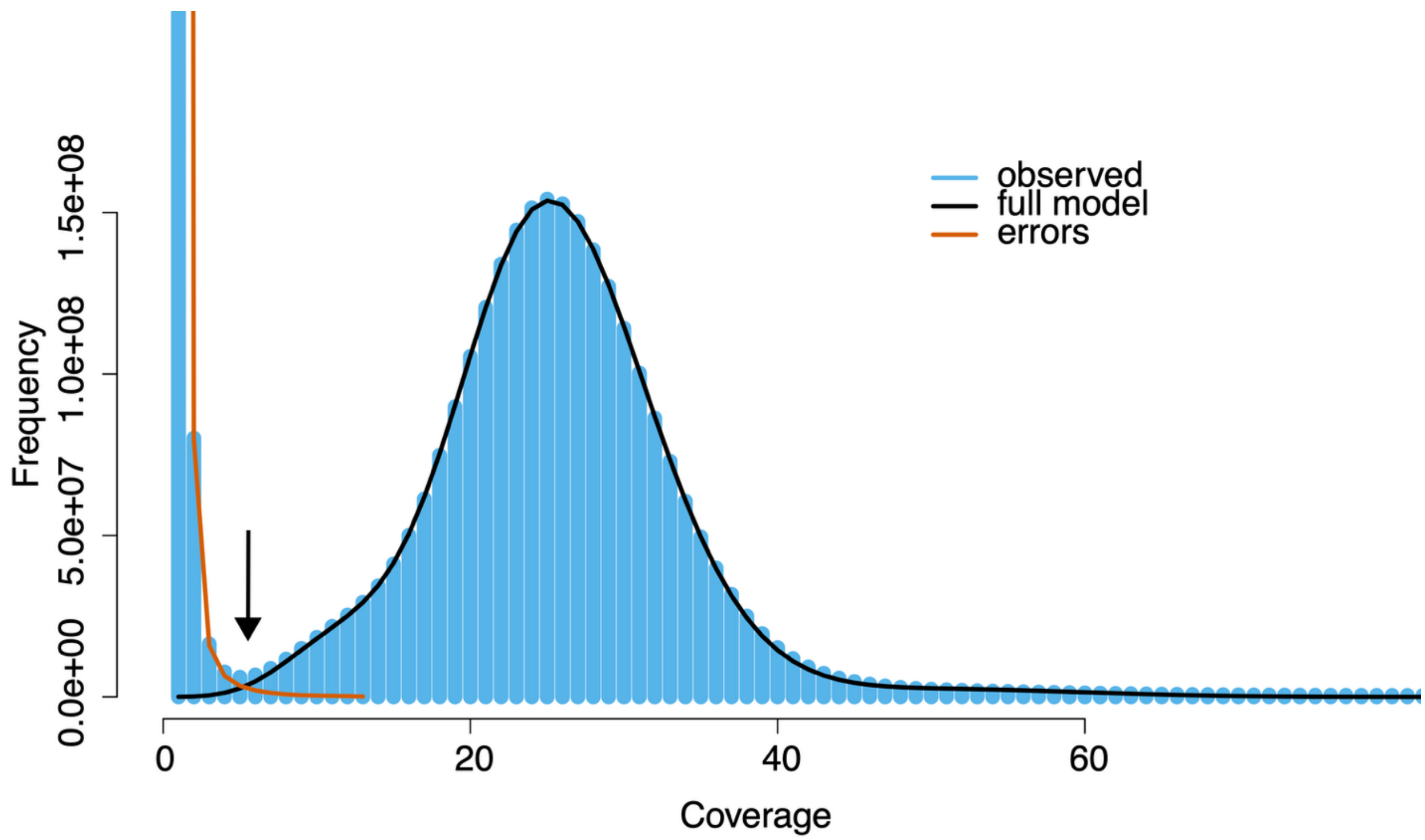

Fig. S53. Removal of potential erroneous k-mers from a short-read k-mer database. A k-mer database (blue histogram, using HG03516 as an example) was constructed using meryl (v) with k=21. The full model of expected k-mer distribution (black line) is fit using a mixture of negative binomial distributions accounting for differences in heterozygous, homozygous, unique and duplicated sequences (Vurture et al., 2017). To minimize the impact of erroneous k-mers in our analysis, we excluded low coverage k-mers by counting k-mers unexplained by the model through first peaks (orange). The vertical arrow indicates the cutoff used to filter potential error k-mers.

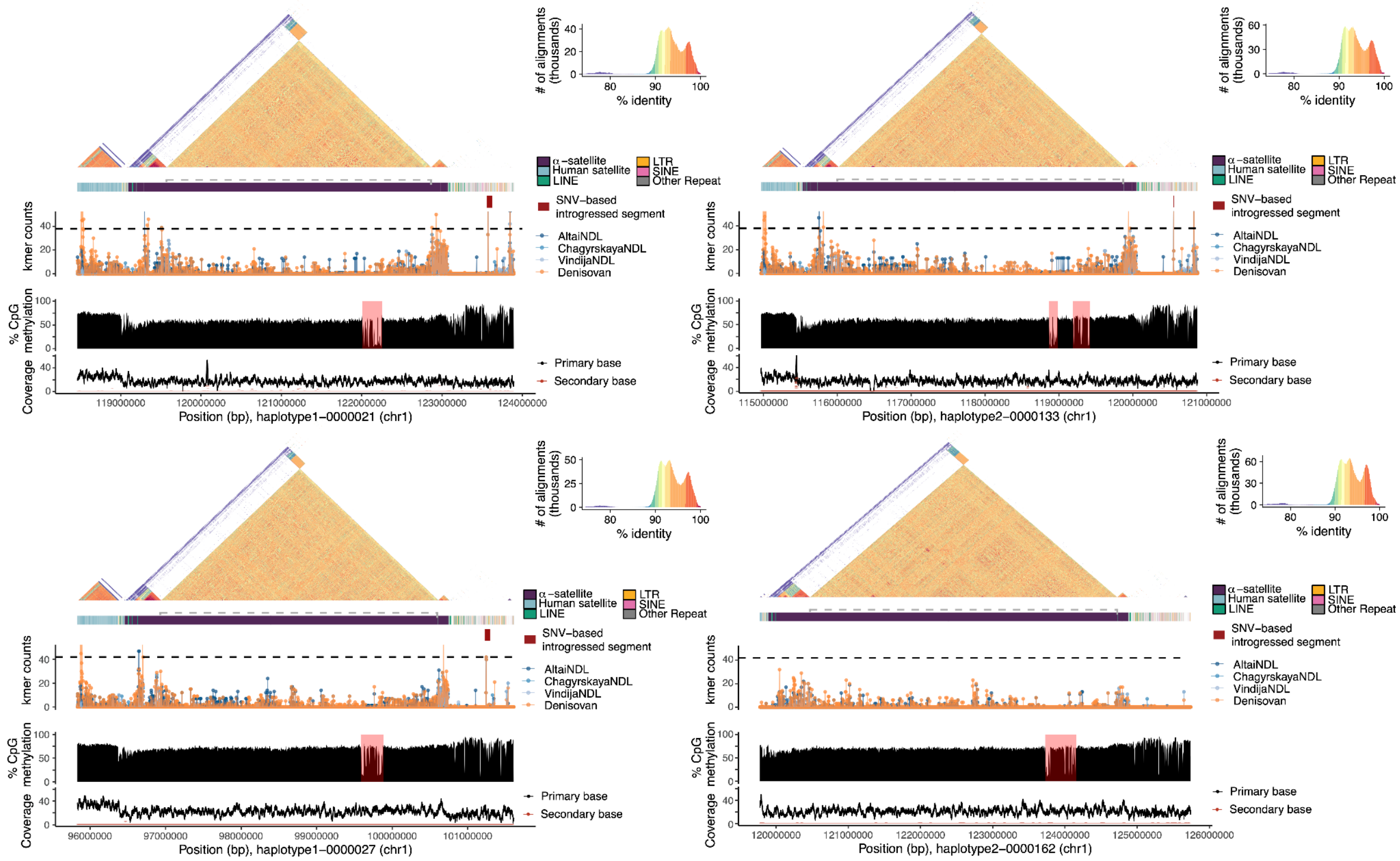

Fig. S54. Assembled chromosome 1 centromeres in PNG15_h1 (top left), PNG15_h2 (top right), PNG16_h1 (bottom left), and PNG16_h2 (bottom right). Top: a heatmap of pairwise sequence identity in 5 kbp windows (StainedGlass) across the centromere and flanking regions. Colors of the heatmap indicate sequence identity as depicted in the inset histogram. The colored vertical bars indicate underlying repeat contents across the centromere regions. The gray dashed line is the inferred centromere sequence based on annotated live α-satellite arrays. Red rectangles represent SNV-based introgression signals. First middle panel: Distribution of archaic-specific k-mers from four high-quality archaic short-read genomes in the centromere region. The black dashed lines indicate the genome-wide k-mer count cutoffs, 38 and 42, that correspond to the genome-wide archaic introgression fraction for PNG15 and PNG16, respectively. Second middle panel: the percentage of averaged methylated CpG frequency across 5-kbp bins across the region. The pink rectangles indicate the putative centromeric hypomethylated loci associated with kinetochore attachment. Bottom: The NucFreq plot for the assembled centromere. Black dots represent the count of the most abundant (primary) base at each position, while red dots indicate the count of the second most frequent (secondary) base. The low representation of secondary bases across the region suggests that the centromere is correctly assembled. **Table S10** provides detailed information about these centromere assemblies.

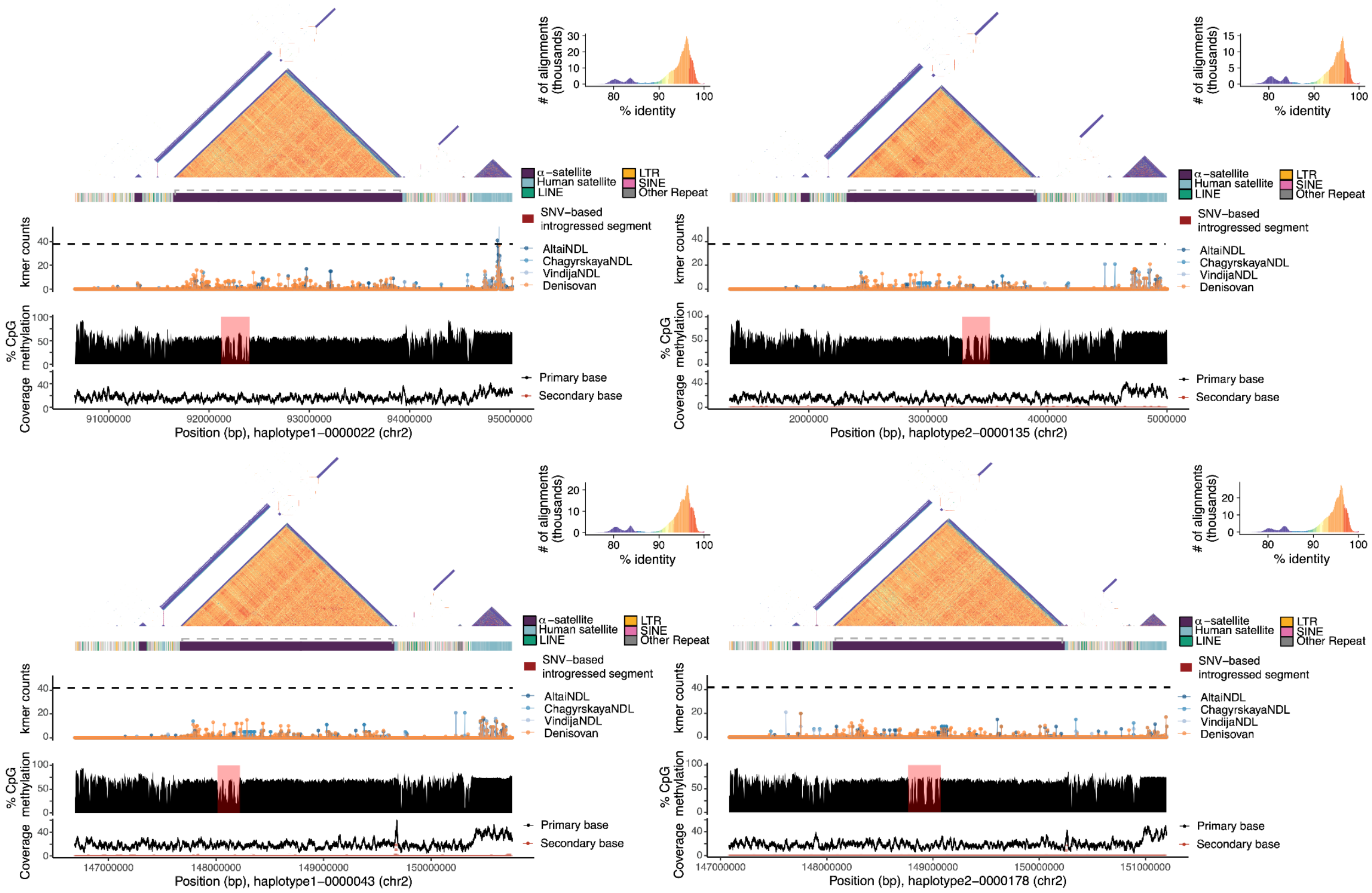

Fig. S55. Assembled chromosome 2 centromeres in PNG15_h1 (top left), PNG15_h2 (top right), PNG16_h1 (bottom left), and PNG16_h2 (bottom right). Top: a heatmap of pairwise sequence identity in 5 kbp windows (StainedGlass) across the centromere and flanking regions. Colors of the heatmap indicate sequence identity as depicted in the inset histogram. The colored vertical bars indicate underlying repeat contents across the centromere regions. The gray dashed line is the inferred centromere sequence based on annotated live α-satellite arrays. Red rectangles represent SNV-based introgression signals. First middle panel: Distribution of archaic-specific k-mers from four high-quality archaic short-read genomes in the centromere region. The black dashed lines indicate the genome-wide k-mer count cutoffs, 38 and 42, that correspond to the genome-wide archaic introgression fraction for PNG15 and PNG16, respectively. Second middle panel: the percentage of averaged methylated CpG frequency across 5-kbp bins across the region. The pink rectangles indicate the putative centromeric hypomethylated loci associated with kinetochore attachment. Bottom: The NucFreq plot for the assembled centromere. Black dots represent the count of the most abundant (primary) base at each position, while red dots indicate the count of the second most frequent (secondary) base. The low representation of secondary bases across the region suggests that the centromere is correctly assembled. **Table S10** provides detailed information about these centromere assemblies.

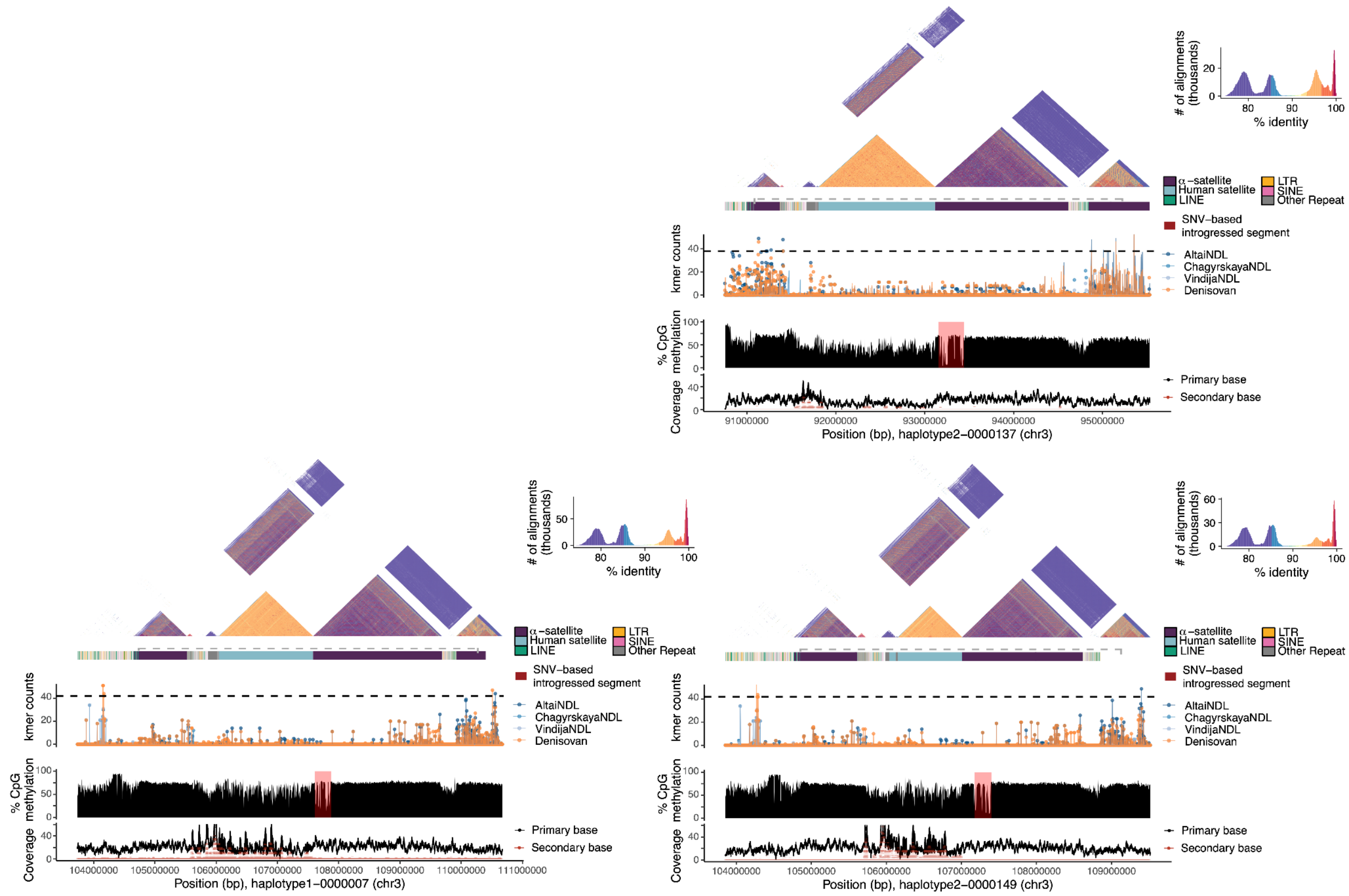

Fig. S56. Assembled chromosome 3 centromeres in PNG15_h2 (top right), PNG16_h1 (bottom left), and PNG16_h2 (bottom right). Top: a heatmap of pairwise sequence identity in 5 kbp windows (StainedGlass) across the centromere and flanking regions. Colors of the heatmap indicate sequence identity as depicted in the inset histogram. The colored vertical bars indicate underlying repeat contents across the centromere regions. The gray dashed line is the inferred centromere sequence based on annotated live α-satellite arrays. Red rectangles represent SNV-based introgression signals. First middle panel: Distribution of archaic-specific k-mers from four high-quality archaic short-read genomes in the centromere region. The black dashed lines indicate the genome-wide k-mer count cutoffs, 38 and 42, that correspond to the genome-wide archaic introgression fraction for PNG15 and PNG16, respectively. Second middle panel: the percentage of averaged methylated CpG frequency across 5-kbp bins across the region. The pink rectangles indicate the putative centromeric hypomethylated loci associated with kinetochore attachment. Bottom: The NucFreq plot for the assembled centromere. Black dots represent the count of the most abundant (primary) base at each position, while red dots indicate the count of the second most frequent (secondary) base. The low representation of secondary bases across the region suggests that the centromere is correctly assembled. **Table S10** provides detailed information about these centromere assemblies.

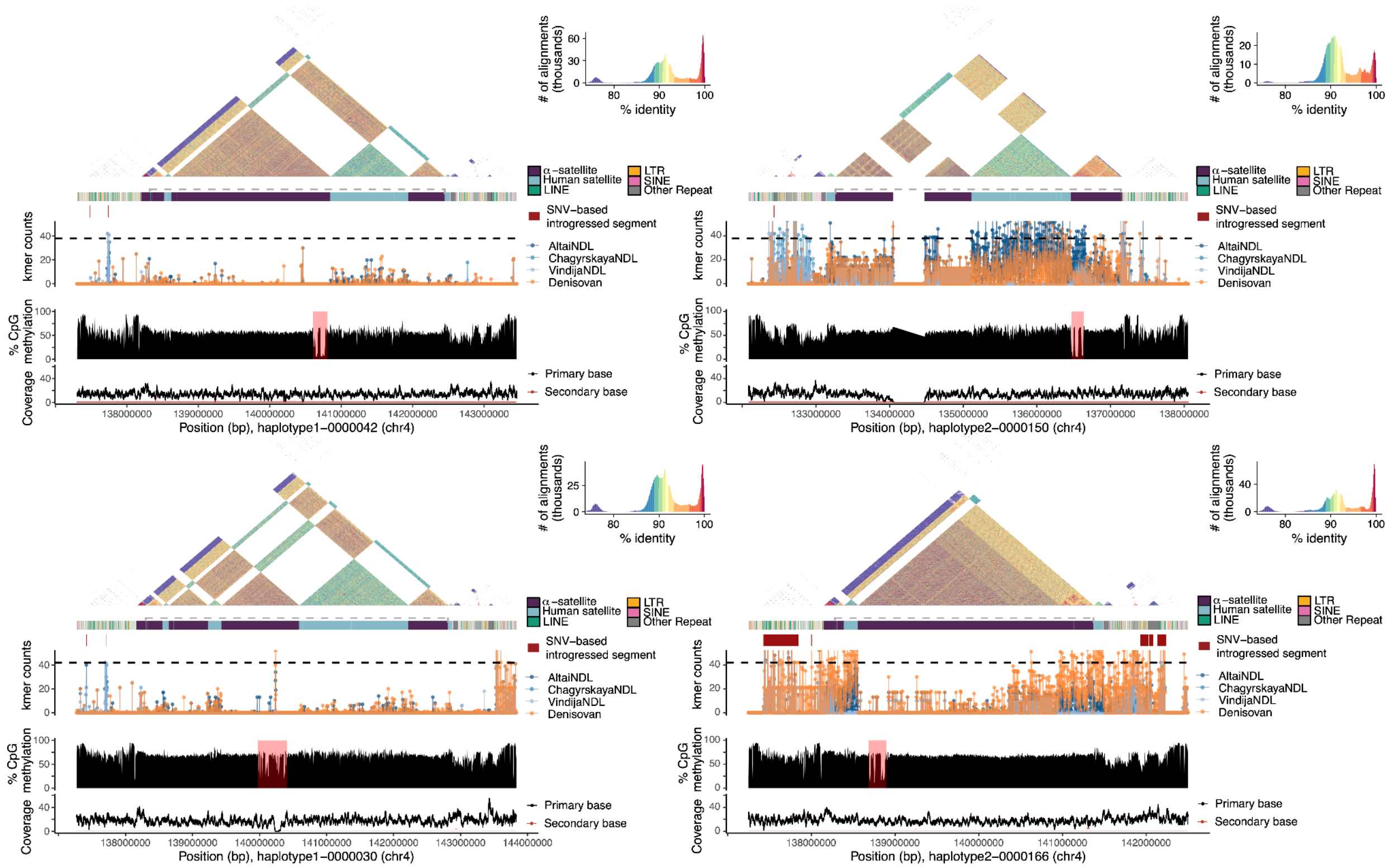

Fig. S57. Assembled chromosome 4 centromeres in PNG15_h1 (top left), PNG15_h2 (top right), PNG16_h1 (bottom left), and PNG16_h2 (bottom right). Top: a heatmap of pairwise sequence identity in 5 kbp windows (StainedGlass) across the centromere and flanking regions. Colors of the heatmap indicate sequence identity as depicted in the inset histogram. The colored vertical bars indicate underlying repeat contents across the centromere regions. The gray dashed line is the inferred centromere sequence based on annotated live α-satellite arrays. Red rectangles represent SNV-based introgression signals. First middle panel: Distribution of archaic-specific k-mers from four high-quality archaic short-read genomes in the centromere region. The black dashed lines indicate the genome-wide k-mer count cutoffs, 38 and 42, that correspond to the genome-wide archaic introgression fraction for PNG15 and PNG16, respectively. Second middle panel: the percentage of averaged methylated CpG frequency across 5-kbp bins across the region. The pink rectangles indicate the putative centromeric hypomethylated loci associated with kinetochore attachment. Bottom: The NucFreq plot for the assembled centromere. Black dots represent the count of the most abundant (primary) base at each position, while red dots indicate the count of the second most frequent (secondary) base. The low representation of secondary bases across the region suggests that the centromere is correctly assembled. **Table S10** provides detailed information about these centromere assemblies.

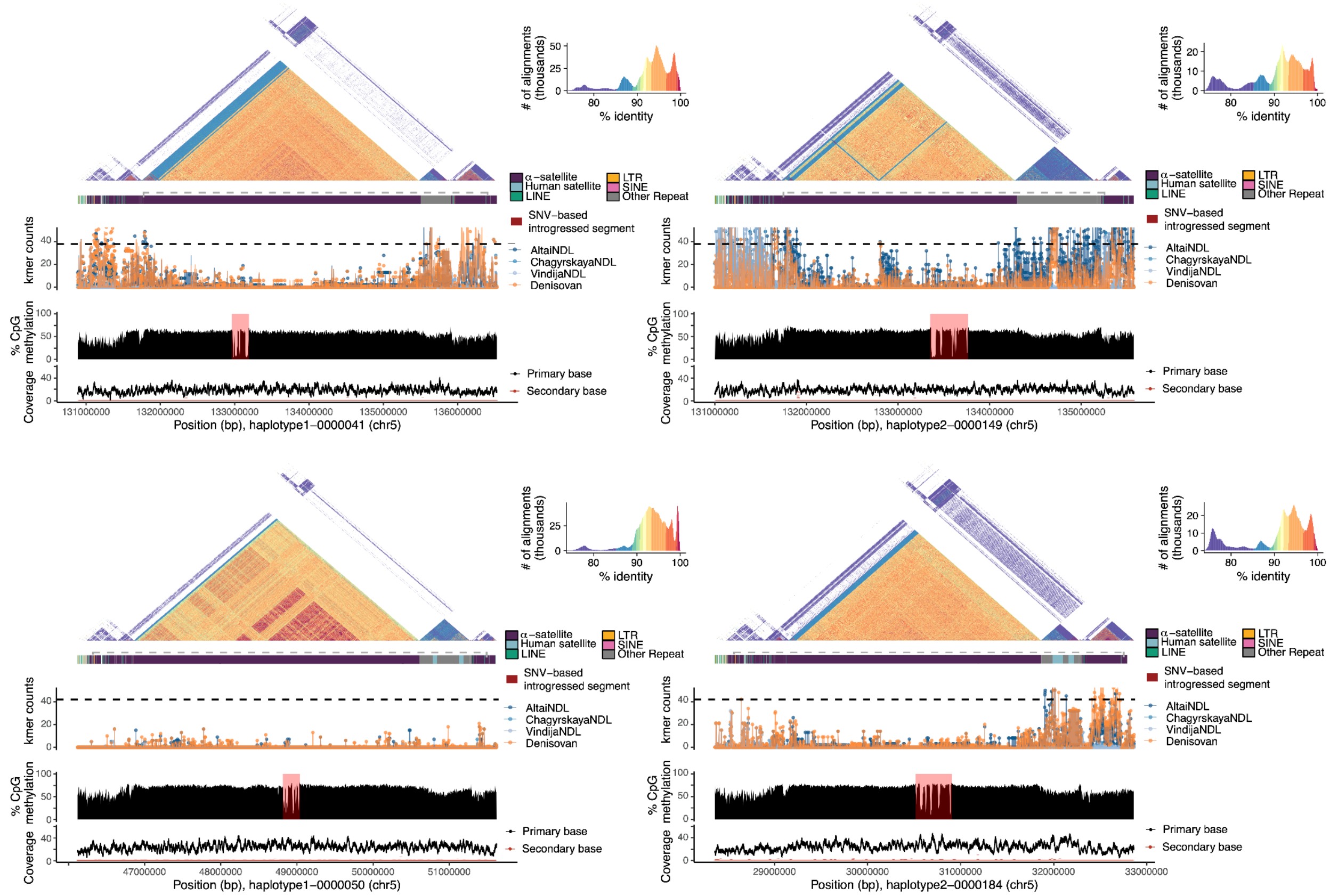

Fig. S58. Assembled chromosome 5 centromeres in PNG15_h1 (top left), PNG15_h2 (top right), PNG16_h1 (bottom left), and PNG16_h2 (bottom right). Top: a heatmap of pairwise sequence identity in 5 kbp windows (StainedGlass) across the centromere and flanking regions. Colors of the heatmap indicate sequence identity as depicted in the inset histogram. The colored vertical bars indicate underlying repeat contents across the centromere regions. The gray dashed line is the inferred centromere sequence based on annotated live α-satellite arrays. Red rectangles represent SNV-based introgression signals. First middle panel: Distribution of archaic-specific k-mers from four high-quality archaic short-read genomes in the centromere region. The black dashed lines indicate the genome-wide k-mer count cutoffs, 38 and 42, that correspond to the genome-wide archaic introgression fraction for PNG15 and PNG16, respectively. Second middle panel: the percentage of averaged methylated CpG frequency across 5-kbp bins across the region. The pink rectangles indicate the putative centromeric hypomethylated loci associated with kinetochore attachment. Bottom: The NucFreq plot for the assembled centromere. Black dots represent the count of the most abundant (primary) base at each position, while red dots indicate the count of the second most frequent (secondary) base. The low representation of secondary bases across the region suggests that the centromere is correctly assembled. **Table S10** provides detailed information about these centromere assemblies.

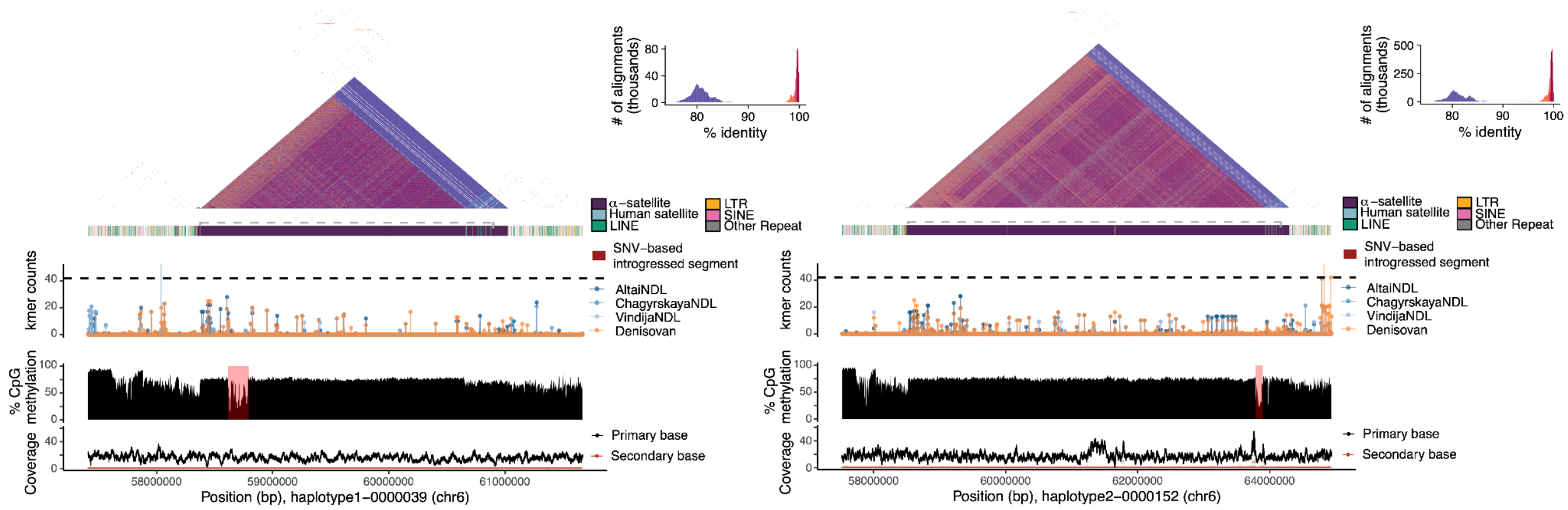

Fig. S59. Assembled chromosome 6 centromeres in PNG16_h1 (left) and PNG16_h2 (right). Top: a heatmap of pairwise sequence identity in 5 kbp windows (StainedGlass) across the centromere and flanking regions. Colors of the heatmap indicate sequence identity as depicted in the inset histogram. The colored vertical bars indicate underlying repeat contents across the centromere regions. The gray dashed line is the inferred centromere sequence based on annotated live α-satellite arrays. Red rectangles represent SNV-based introgression signals. First middle panel: Distribution of archaic-specific k-mers from four high-quality archaic short-read genomes in the centromere region. The black dashed lines indicate the genome-wide k-mer count cutoffs, 38 and 42, that correspond to the genome-wide archaic introgression fraction for PNG15 and PNG16, respectively. Second middle panel: the percentage of averaged methylated CpG frequency across 5-kbp bins across the region. The pink rectangles indicate the putative centromeric hypomethylated loci associated with kinetochore attachment. Bottom: The NucFreq plot for the assembled centromere. Black dots represent the count of the most abundant (primary) base at each position, while red dots indicate the count of the second most frequent (secondary) base. The low representation of secondary bases across the region suggests that the centromere is correctly assembled. **Table S10** provides detailed information about these centromere assemblies.

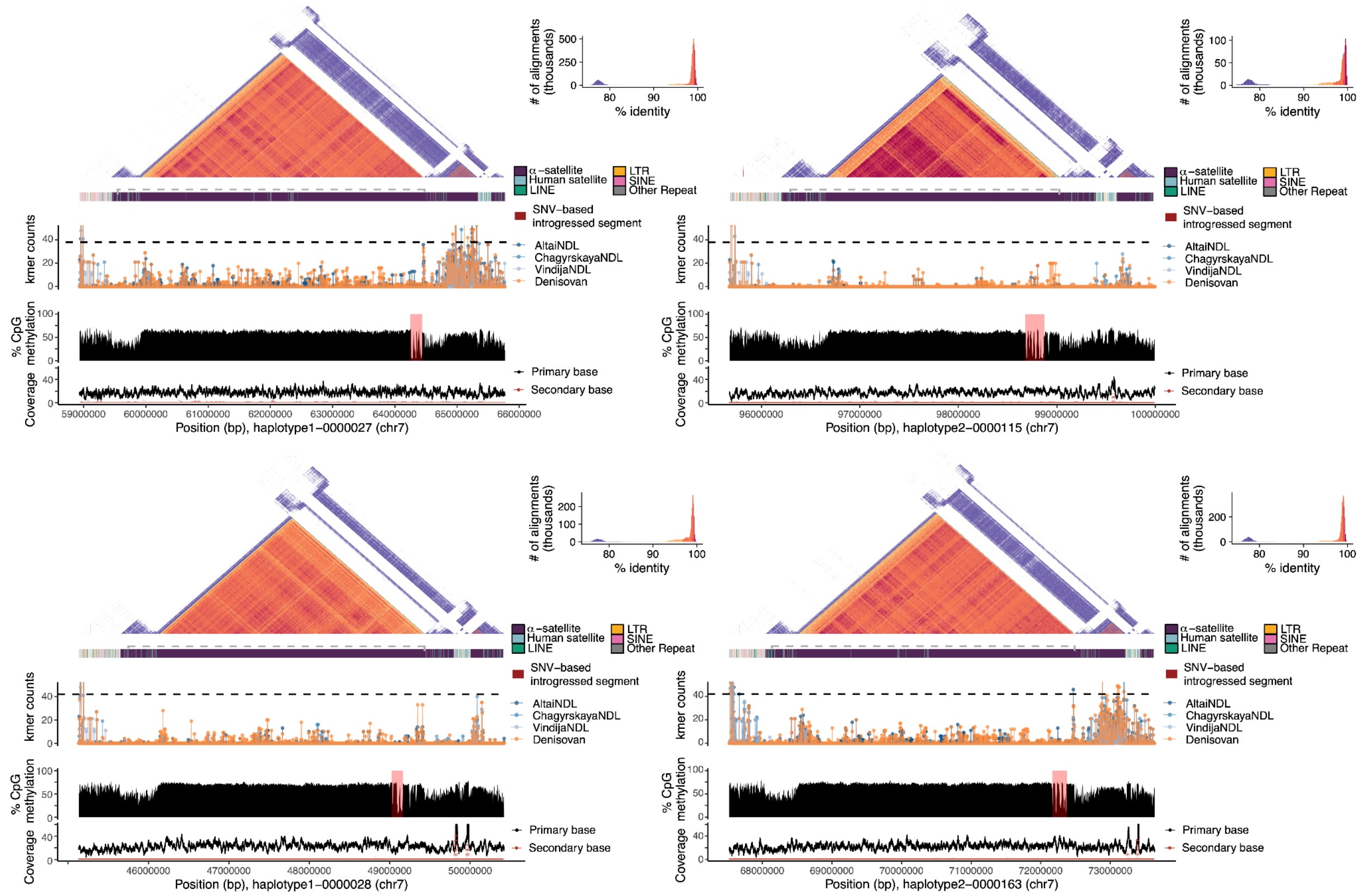

Fig. S60. Assembled chromosome 7 centromeres in PNG15_h1 (top left), PNG15_h2 (top right), PNG16_h1 (bottom left), and PNG16_h2 (bottom right). Top: a heatmap of pairwise sequence identity in 5 kbp windows (StainedGlass) across the centromere and flanking regions. Colors of the heatmap indicate sequence identity as depicted in the inset histogram. The colored vertical bars indicate underlying repeat contents across the centromere regions. The gray dashed line is the inferred centromere sequence based on annotated live α-satellite arrays. Red rectangles represent SNV-based introgression signals. First middle panel: Distribution of archaic-specific k-mers from four high-quality archaic short-read genomes in the centromere region. The black dashed lines indicate the genome-wide k-mer count cutoffs, 38 and 42, that correspond to the genome-wide archaic introgression fraction for PNG15 and PNG16, respectively. Second middle panel: the percentage of averaged methylated CpG frequency across 5-kbp bins across the region. The pink rectangles indicate the putative centromeric hypomethylated loci associated with kinetochore attachment. Bottom: The NucFreq plot for the assembled centromere. Black dots represent the count of the most abundant (primary) base at each position, while red dots indicate the count of the second most frequent (secondary) base. The low representation of secondary bases across the region suggests that the centromere is correctly assembled. **Table S10** provides detailed information about these centromere assemblies.

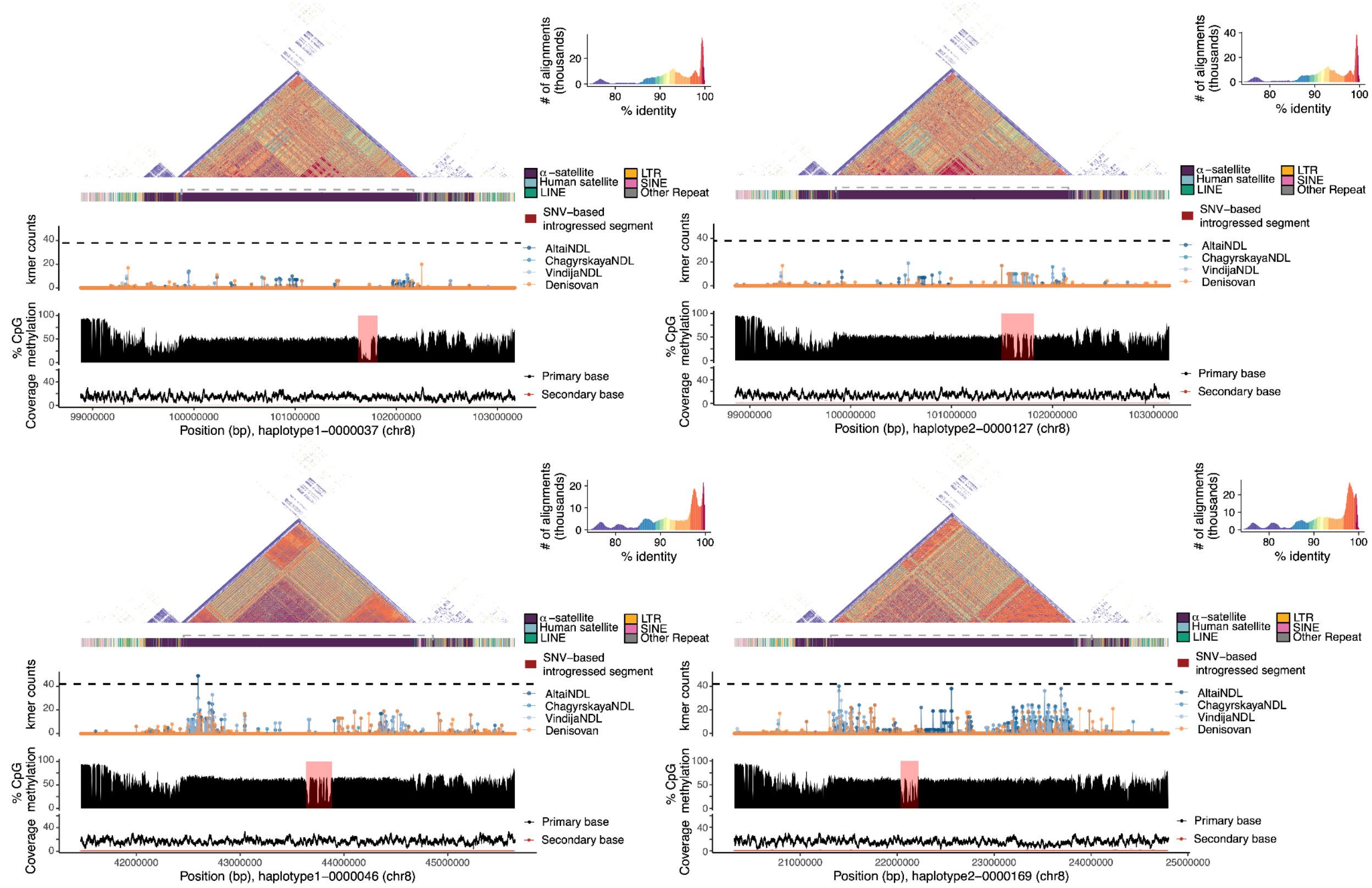

Fig. S61. Assembled chromosome 8 centromeres in PNG15_h1 (top left), PNG15_h2 (top right), PNG16_h1 (bottom left), and PNG16_h2 (bottom right). Top: a heatmap of pairwise sequence identity in 5 kbp windows (StainedGlass) across the centromere and flanking regions. Colors of the heatmap indicate sequence identity as depicted in the inset histogram. The colored vertical bars indicate underlying repeat contents across the centromere regions. The gray dashed line is the inferred centromere sequence based on annotated live α-satellite arrays. Red rectangles represent SNV-based introgression signals. First middle panel: Distribution of archaic-specific k-mers from four high-quality archaic short-read genomes in the centromere region. The black dashed lines indicate the genome-wide k-mer count cutoffs, 38 and 42, that correspond to the genome-wide archaic introgression fraction for PNG15 and PNG16, respectively. Second middle panel: the percentage of averaged methylated CpG frequency across 5-kbp bins across the region. The pink rectangles indicate the putative centromeric hypomethylated loci associated with kinetochore attachment. Bottom: The NucFreq plot for the assembled centromere. Black dots represent the count of the most abundant (primary) base at each position, while red dots indicate the count of the second most frequent (secondary) base. The low representation of secondary bases across the region suggests that the centromere is correctly assembled. **Table S10** provides detailed information about these centromere assemblies.

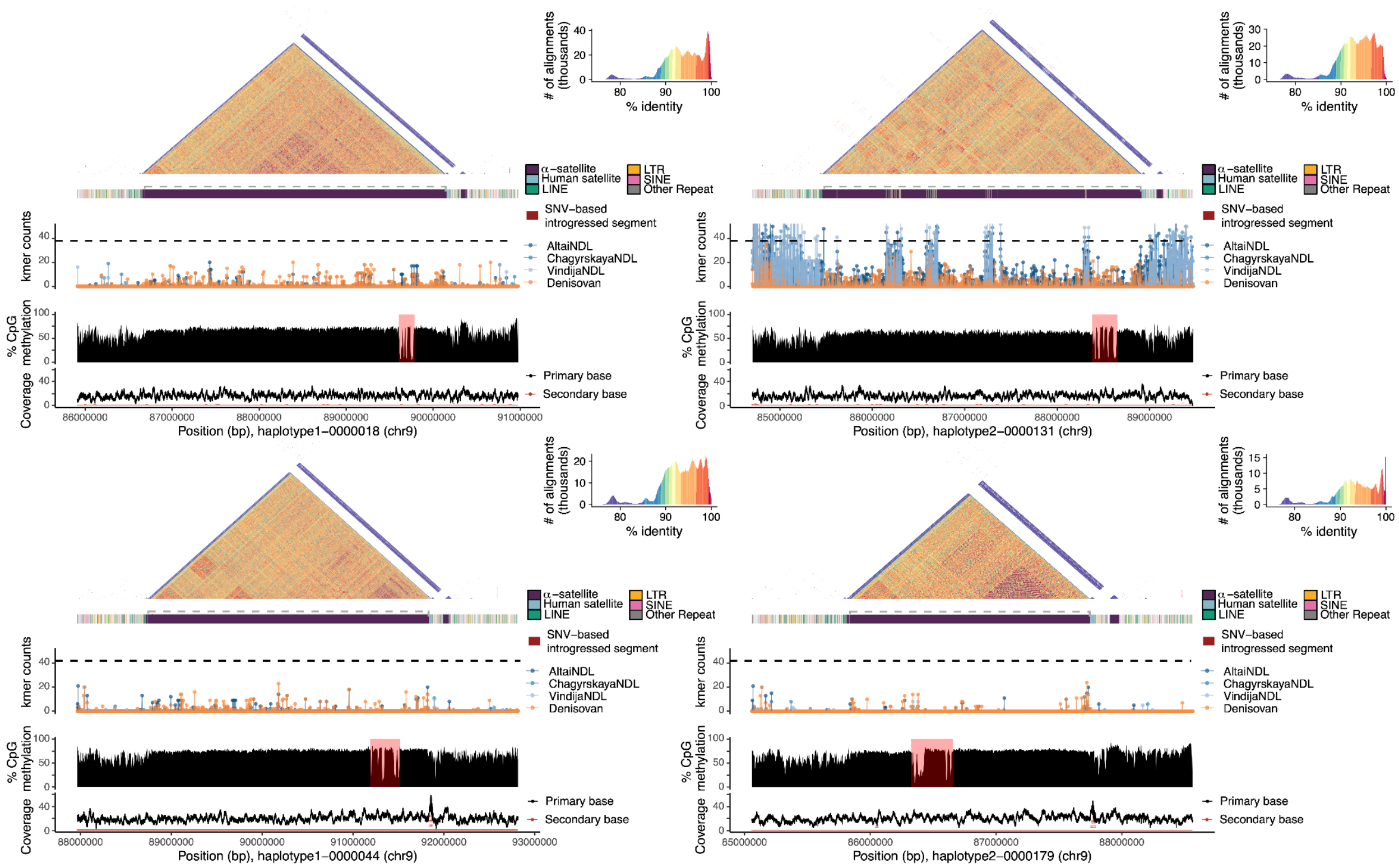

Fig. S62. Assembled chromosome 9 centromeres in PNG15_h1 (top left), PNG15_h2 (top right), PNG16_h1 (bottom left), and PNG16_h2 (bottom right). Top: a heatmap of pairwise sequence identity in 5 kbp windows (StainedGlass) across the centromere and flanking regions. Colors of the heatmap indicate sequence identity as depicted in the inset histogram. The colored vertical bars indicate underlying repeat contents across the centromere regions. The gray dashed line is the inferred centromere sequence based on annotated live α-satellite arrays. Red rectangles represent SNV-based introgression signals. First middle panel: Distribution of archaic-specific k-mers from four high-quality archaic short-read genomes in the centromere region. The black dashed lines indicate the genome-wide k-mer count cutoffs, 38 and 42, that correspond to the genome-wide archaic introgression fraction for PNG15 and PNG16, respectively. Second middle panel: the percentage of averaged methylated CpG frequency across 5-kbp bins across the region. The pink rectangles indicate the putative centromeric hypomethylated loci associated with kinetochore attachment. Bottom: The NucFreq plot for the assembled centromere. Black dots represent the count of the most abundant (primary) base at each position, while red dots indicate the count of the second most frequent (secondary) base. The low representation of secondary bases across the region suggests that the centromere is correctly assembled. **Table S10** provides detailed information about these centromere assemblies.

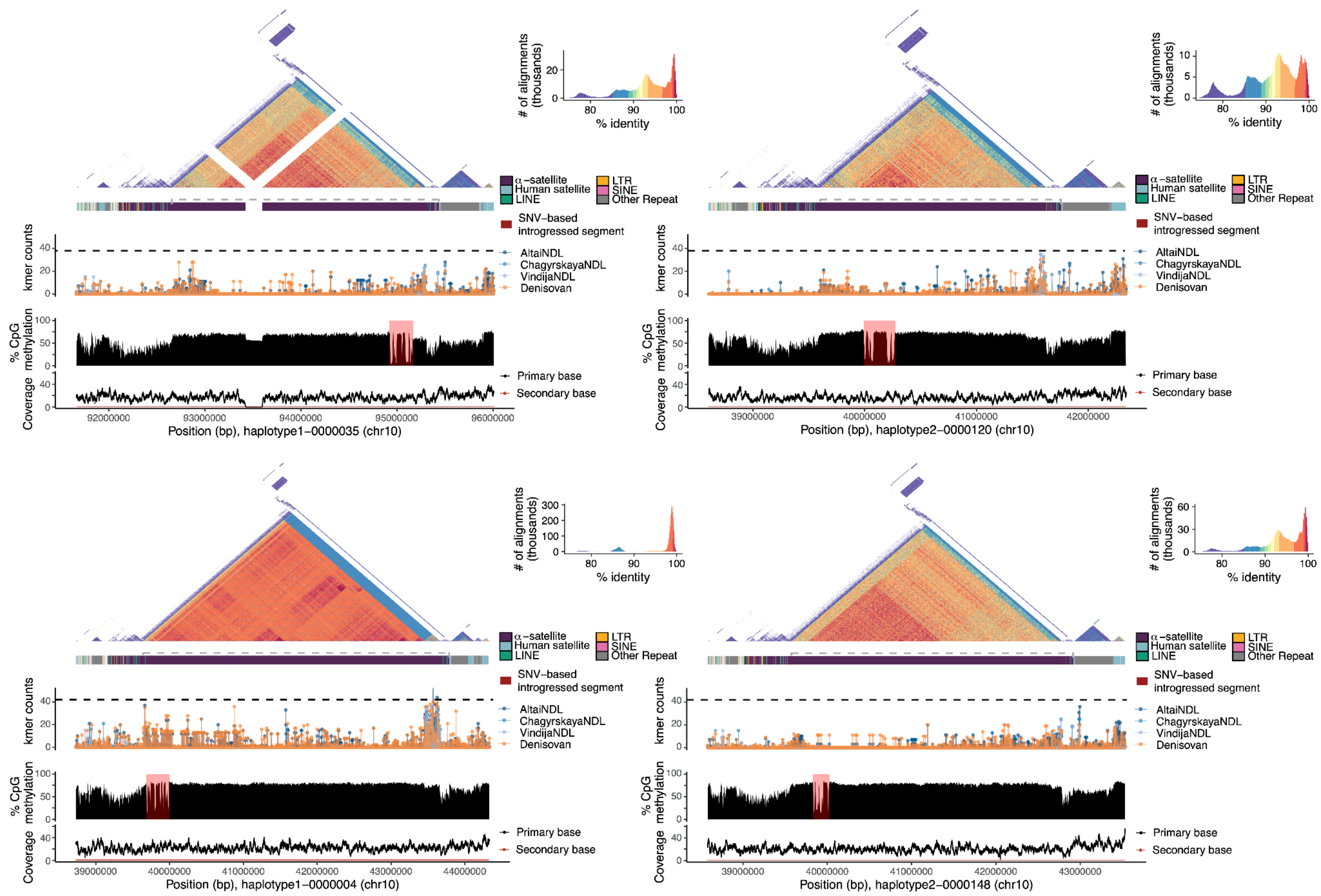

Fig. S63. Assembled chromosome 10 centromeres in PNG15_h1 (top left), PNG15_h2 (top right), PNG16_h1 (bottom left), and PNG16_h2 (bottom right). Top: a heatmap of pairwise sequence identity in 5 kbp windows (StainedGlass) across the centromere and flanking regions. Colors of the heatmap indicate sequence identity as depicted in the inset histogram. The colored vertical bars indicate underlying repeat contents across the centromere regions. The gray dashed line is the inferred centromere sequence based on annotated live α-satellite arrays. Red rectangles represent SNV-based introgression signals. First middle panel: Distribution of archaic-specific k-mers from four high-quality archaic short-read genomes in the centromere region. The black dashed lines indicate the genome-wide k-mer count cutoffs, 38 and 42, that correspond to the genome-wide archaic introgression fraction for PNG15 and PNG16, respectively. Second middle panel: the percentage of averaged methylated CpG frequency across 5-kbp bins across the region. The pink rectangles indicate the putative centromeric hypomethylated loci associated with kinetochore attachment. Bottom: The NucFreq plot for the assembled centromere. Black dots represent the count of the most abundant (primary) base at each position, while red dots indicate the count of the second most frequent (secondary) base. The low representation of secondary bases across the region suggests that the centromere is correctly assembled. **Table S10** provides detailed information about these centromere assemblies.

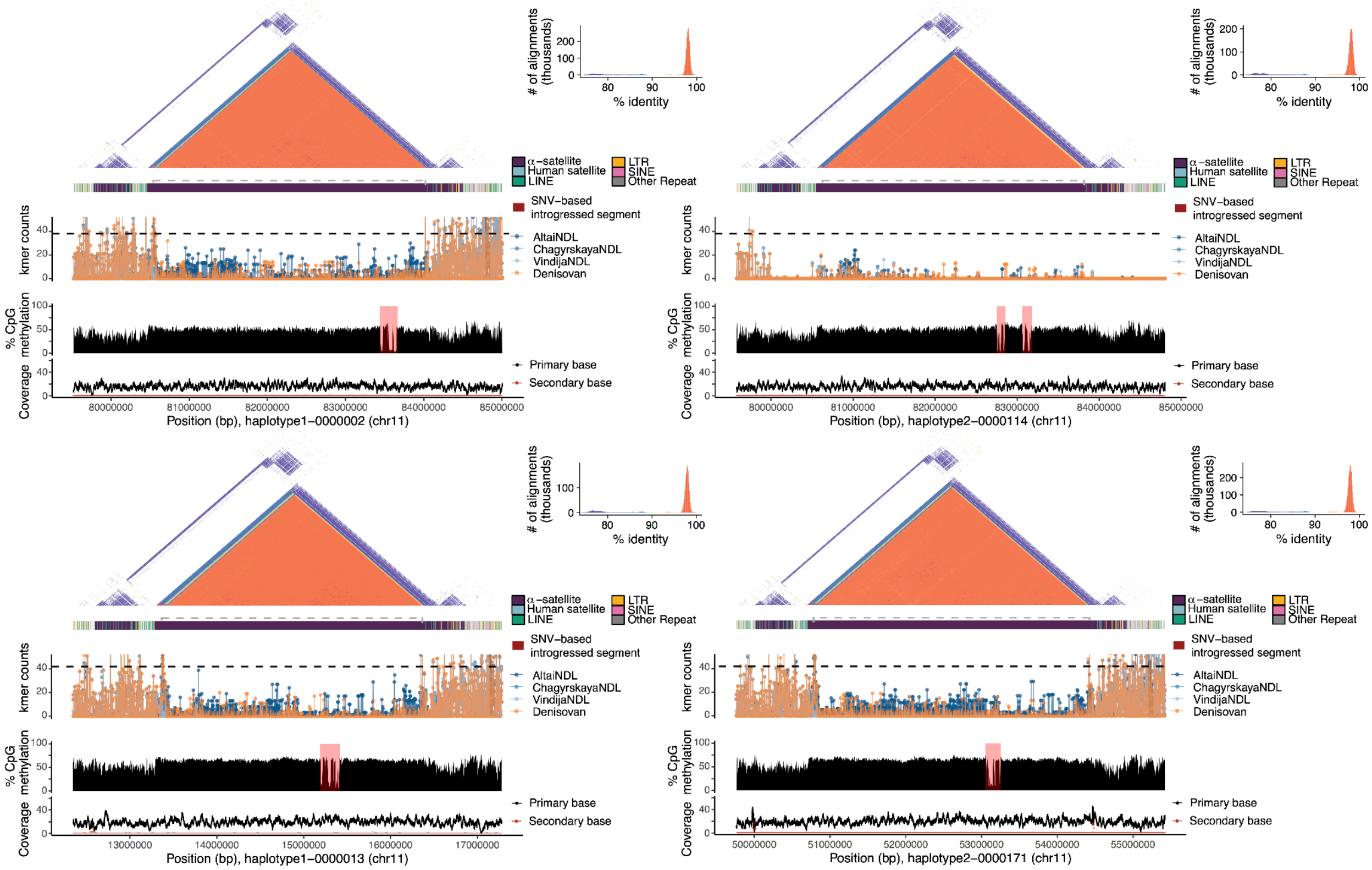

Fig. S64. Assembled chromosome 11 centromeres in PNG15_h1 (top left), PNG15_h2 (top right), PNG16_h1 (bottom left), and PNG16_h2 (bottom right). Top: a heatmap of pairwise sequence identity in 5 kbp windows (StainedGlass) across the centromere and flanking regions. Colors of the heatmap indicate sequence identity as depicted in the inset histogram. The colored vertical bars indicate underlying repeat contents across the centromere regions. The gray dashed line is the inferred centromere sequence based on annotated live α-satellite arrays. Red rectangles represent SNV-based introgression signals. First middle panel: Distribution of archaic-specific k-mers from four high-quality archaic short-read genomes in the centromere region. The black dashed lines indicate the genome-wide k-mer count cutoffs, 38 and 42, that correspond to the genome-wide archaic introgression fraction for PNG15 and PNG16, respectively. Second middle panel: the percentage of averaged methylated CpG frequency across 5-kbp bins across the region. The pink rectangles indicate the putative centromeric hypomethylated loci associated with kinetochore attachment. Bottom: The NucFreq plot for the assembled centromere. Black dots represent the count of the most abundant (primary) base at each position, while red dots indicate the count of the second most frequent (secondary) base. The low representation of secondary bases across the region suggests that the centromere is correctly assembled. **Table S10** provides detailed information about these centromere assemblies.

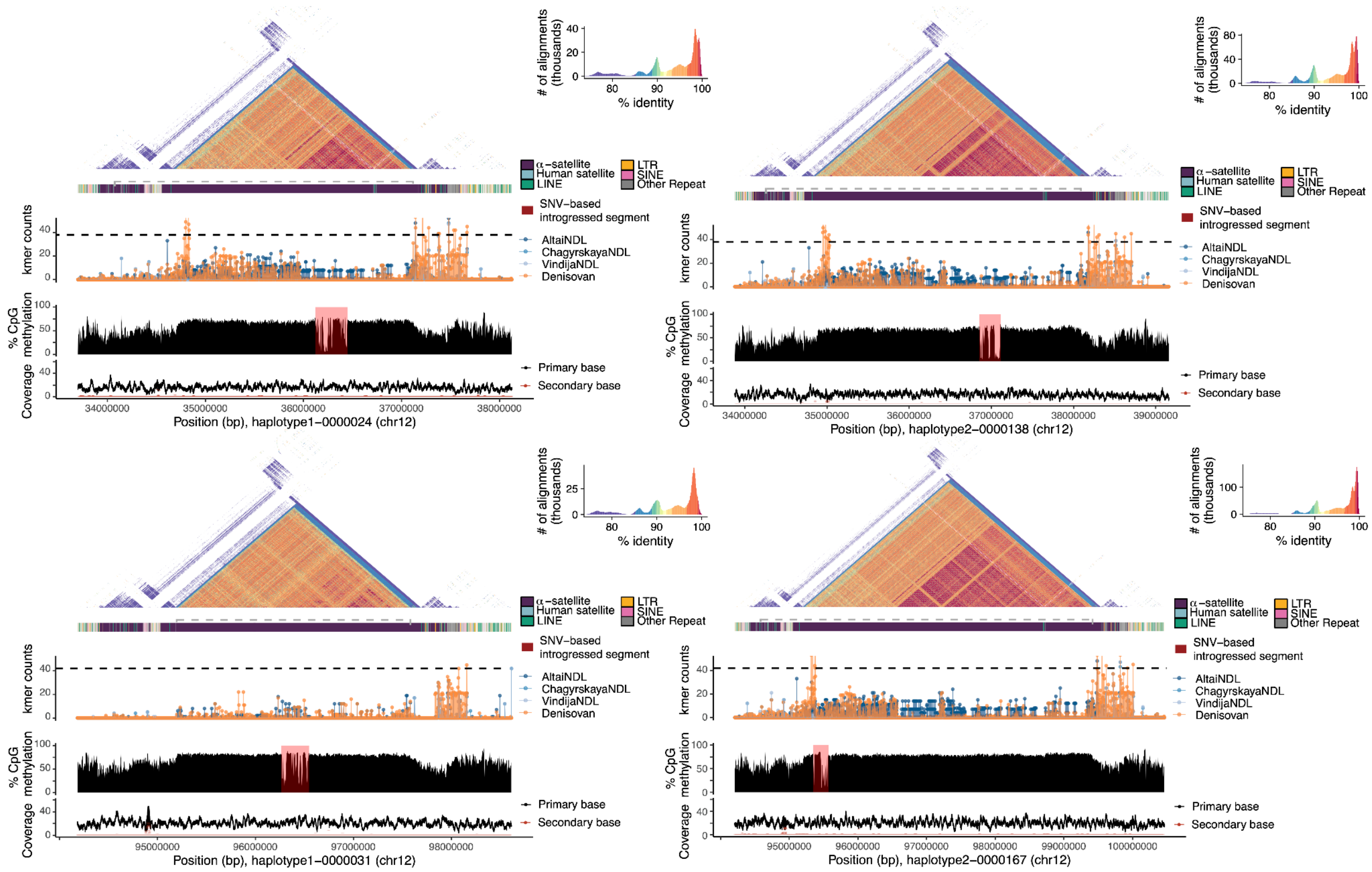

Fig. S65. Assembled chromosome 12 centromeres in PNG15_h1 (top left), PNG15_h2 (top right), PNG16_h1 (bottom left), and PNG16_h2 (bottom right). Top: a heatmap of pairwise sequence identity in 5 kbp windows (StainedGlass) across the centromere and flanking regions. Colors of the heatmap indicate sequence identity as depicted in the inset histogram. The colored vertical bars indicate underlying repeat contents across the centromere regions. The gray dashed line is the inferred centromere sequence based on annotated live α-satellite arrays. Red rectangles represent SNV-based introgression signals. First middle panel: Distribution of archaic-specific k-mers from four high-quality archaic short-read genomes in the centromere region. The black dashed lines indicate the genome-wide k-mer count cutoffs, 38 and 42, that correspond to the genome-wide archaic introgression fraction for PNG15 and PNG16, respectively. Second middle panel: the percentage of averaged methylated CpG frequency across 5-kbp bins across the region. The pink rectangles indicate the putative centromeric hypomethylated loci associated with kinetochore attachment. Bottom: The NucFreq plot for the assembled centromere. Black dots represent the count of the most abundant (primary) base at each position, while red dots indicate the count of the second most frequent (secondary) base. The low representation of secondary bases across the region suggests that the centromere is correctly assembled. **Table S10** provides detailed information about these centromere assemblies.

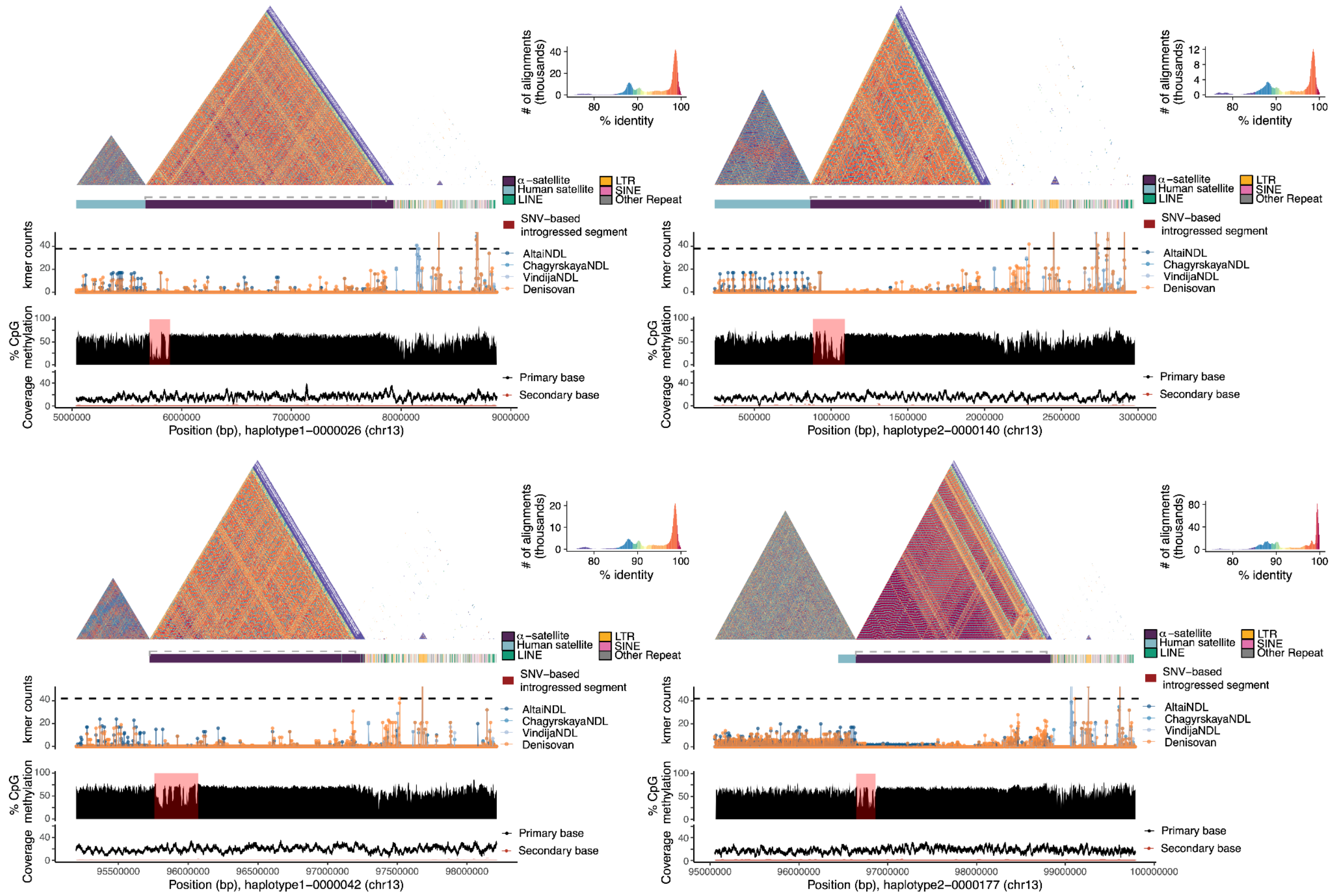

Fig. S66. Assembled chromosome 13 centromeres in PNG15_h1 (top left), PNG15_h2 (top right), PNG16_h1 (bottom left), and PNG16_h2 (bottom right). Top: a heatmap of pairwise sequence identity in 5 kbp windows (StainedGlass) across the centromere and flanking regions. Colors of the heatmap indicate sequence identity as depicted in the inset histogram. The colored vertical bars indicate underlying repeat contents across the centromere regions. The gray dashed line is the inferred centromere sequence based on annotated live α-satellite arrays. Red rectangles represent SNV-based introgression signals. First middle panel: Distribution of archaic-specific k-mers from four high-quality archaic short-read genomes in the centromere region. The black dashed lines indicate the genome-wide k-mer count cutoffs, 38 and 42, that correspond to the genome-wide archaic introgression fraction for PNG15 and PNG16, respectively. Second middle panel: the percentage of averaged methylated CpG frequency across 5-kbp bins across the region. The pink rectangles indicate the putative centromeric hypomethylated loci associated with kinetochore attachment. Bottom: The NucFreq plot for the assembled centromere. Black dots represent the count of the most abundant (primary) base at each position, while red dots indicate the count of the second most frequent (secondary) base. The low representation of secondary bases across the region suggests that the centromere is correctly assembled. **Table S10** provides detailed information about these centromere assemblies.

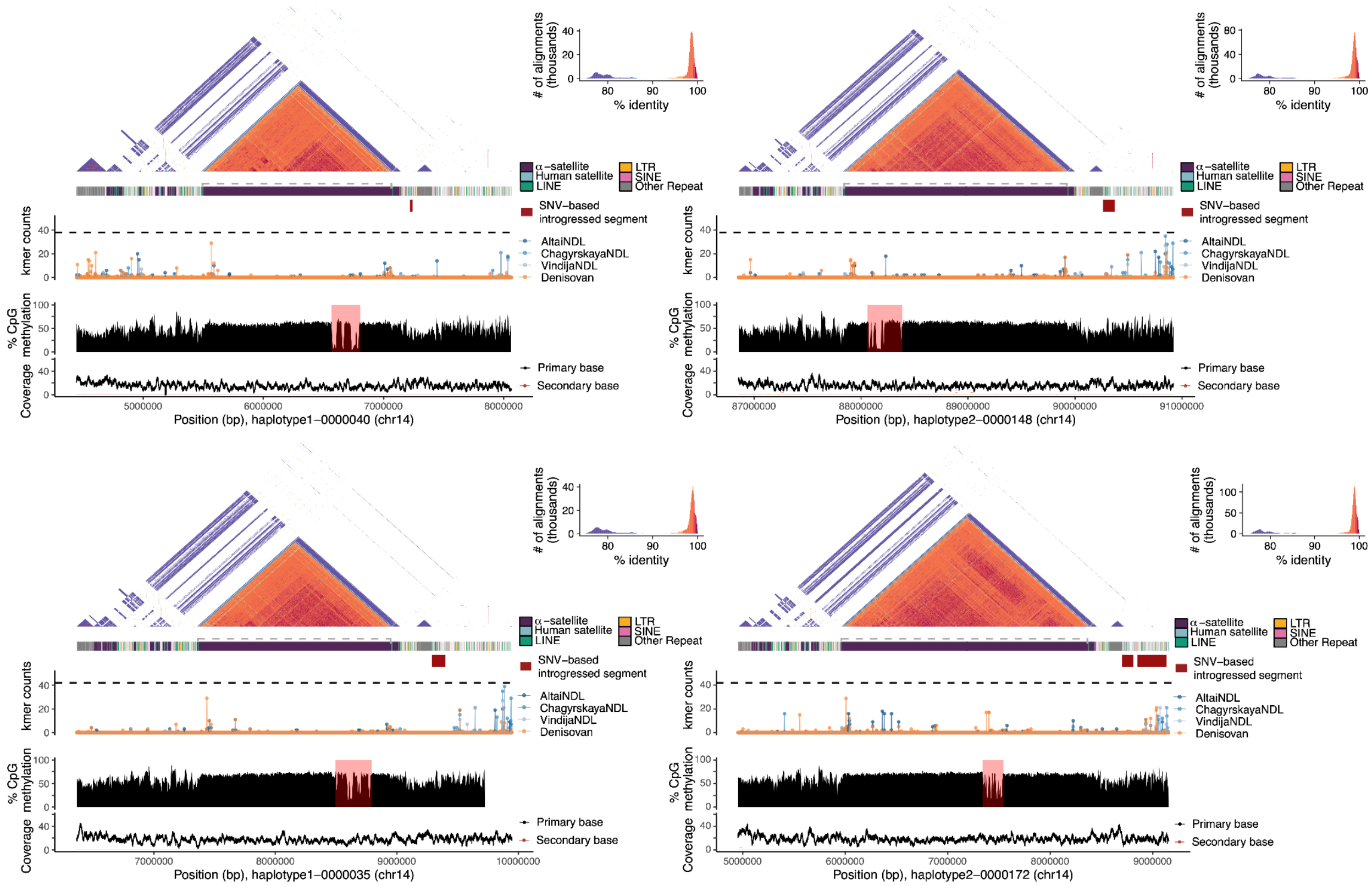

Fig. S67. Assembled chromosome 14 centromeres in PNG15_h1 (top left), PNG15_h2 (top right), PNG16_h1 (bottom left), and PNG16_h2 (bottom right). Top: a heatmap of pairwise sequence identity in 5 kbp windows (StainedGlass) across the centromere and flanking regions. Colors of the heatmap indicate sequence identity as depicted in the inset histogram. The colored vertical bars indicate underlying repeat contents across the centromere regions. The gray dashed line is the inferred centromere sequence based on annotated live α-satellite arrays. Red rectangles represent SNV-based introgression signals. First middle panel: Distribution of archaic-specific k-mers from four high-quality archaic short-read genomes in the centromere region. The black dashed lines indicate the genome-wide k-mer count cutoffs, 38 and 42, that correspond to the genome-wide archaic introgression fraction for PNG15 and PNG16, respectively. Second middle panel: the percentage of averaged methylated CpG frequency across 5-kbp bins across the region. The pink rectangles indicate the putative centromeric hypomethylated loci associated with kinetochore attachment. Bottom: The NucFreq plot for the assembled centromere. Black dots represent the count of the most abundant (primary) base at each position, while red dots indicate the count of the second most frequent (secondary) base. The low representation of secondary bases across the region suggests that the centromere is correctly assembled. **Table S10** provides detailed information about these centromere assemblies.

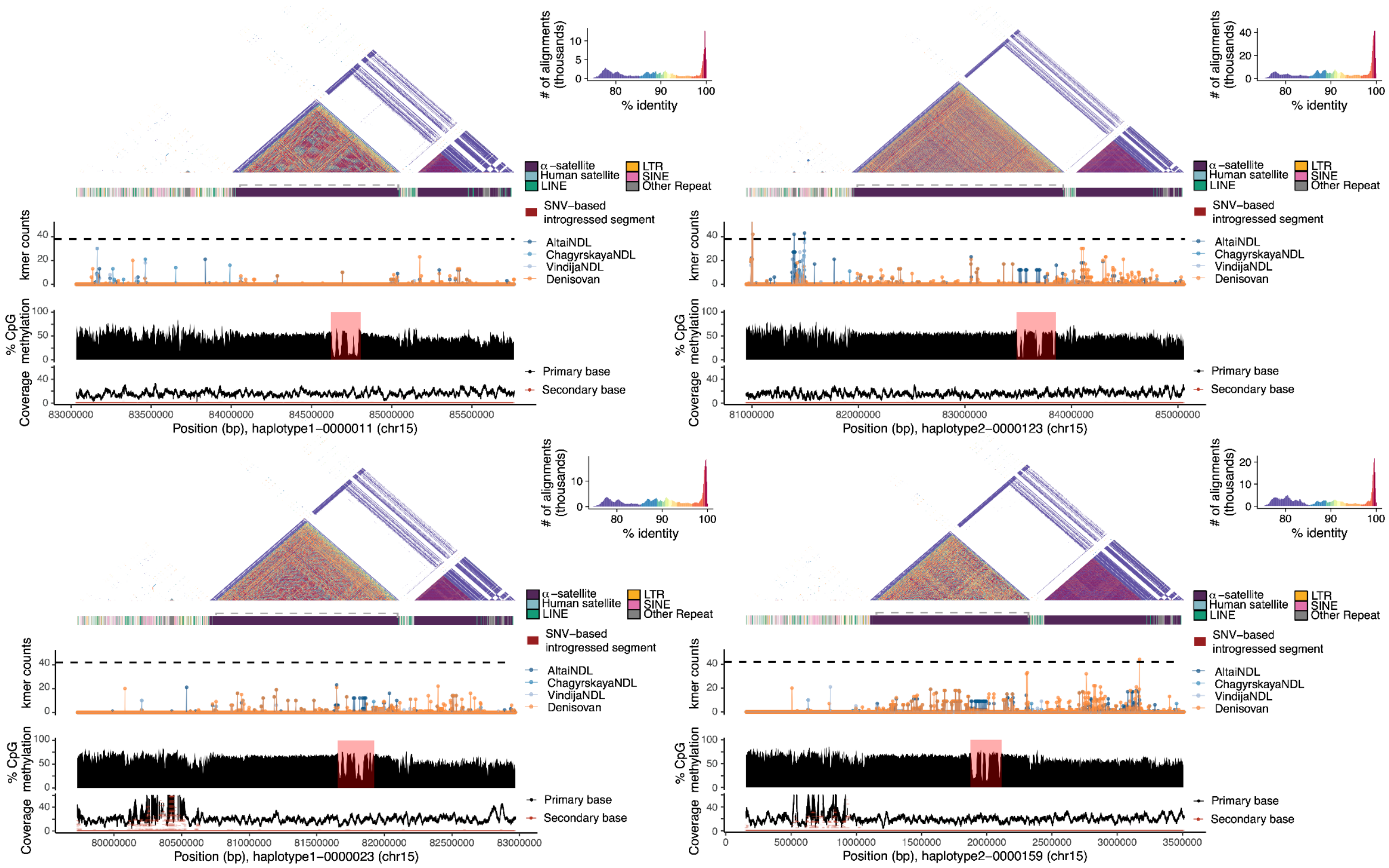

Fig. S68. Assembled chromosome 15 centromeres in PNG15_h1 (top left), PNG15_h2 (top right), PNG16_h1 (bottom left), and PNG16_h2 (bottom right). Top: a heatmap of pairwise sequence identity in 5 kbp windows (StainedGlass) across the centromere and flanking regions. Colors of the heatmap indicate sequence identity as depicted in the inset histogram. The colored vertical bars indicate underlying repeat contents across the centromere regions. The gray dashed line is the inferred centromere sequence based on annotated live α-satellite arrays. Red rectangles represent SNV-based introgression signals. First middle panel: Distribution of archaic-specific k-mers from four high-quality archaic short-read genomes in the centromere region. The black dashed lines indicate the genome-wide k-mer count cutoffs, 38 and 42, that correspond to the genome-wide archaic introgression fraction for PNG15 and PNG16, respectively. Second middle panel: the percentage of averaged methylated CpG frequency across 5-kbp bins across the region. The pink rectangles indicate the putative centromeric hypomethylated loci associated with kinetochore attachment. Bottom: The NucFreq plot for the assembled centromere. Black dots represent the count of the most abundant (primary) base at each position, while red dots indicate the count of the second most frequent (secondary) base. The low representation of secondary bases across the region suggests that the centromere is correctly assembled. **Table S10** provides detailed information about these centromere assemblies.

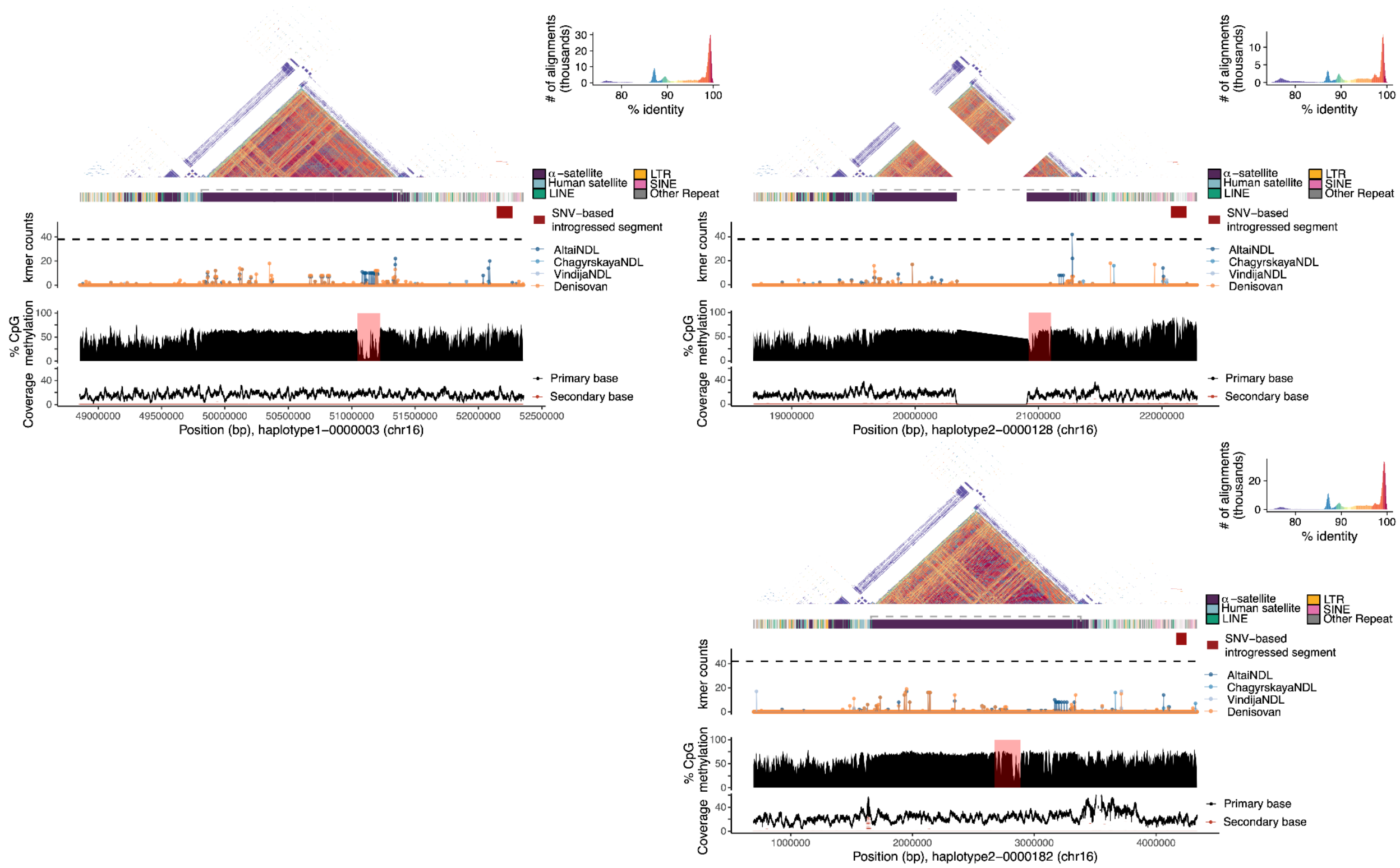

Fig. S69. Assembled chromosome 16 centromeres in PNG15_h1 (top left), PNG15_h2 (top right), PNG16_h1 (bottom left), and PNG16_h2 (bottom right). Top: a heatmap of pairwise sequence identity in 5 kbp windows (StainedGlass) across the centromere and flanking regions. Colors of the heatmap indicate sequence identity as depicted in the inset histogram. The colored vertical bars indicate underlying repeat contents across the centromere regions. The gray dashed line is the inferred centromere sequence based on annotated live α-satellite arrays. Red rectangles represent SNV-based introgression signals. First middle panel: Distribution of archaic-specific k-mers from four high-quality archaic short-read genomes in the centromere region. The black dashed lines indicate the genome-wide k-mer count cutoffs, 38 and 42, that correspond to the genome-wide archaic introgression fraction for PNG15 and PNG16, respectively. Second middle panel: the percentage of averaged methylated CpG frequency across 5-kbp bins across the region. The pink rectangles indicate the putative centromeric hypomethylated loci associated with kinetochore attachment. Bottom: The NucFreq plot for the assembled centromere. Black dots represent the count of the most abundant (primary) base at each position, while red dots indicate the count of the second most frequent (secondary) base. The low representation of secondary bases across the region suggests that the centromere is correctly assembled. **Table S10** provides detailed information about these centromere assemblies.

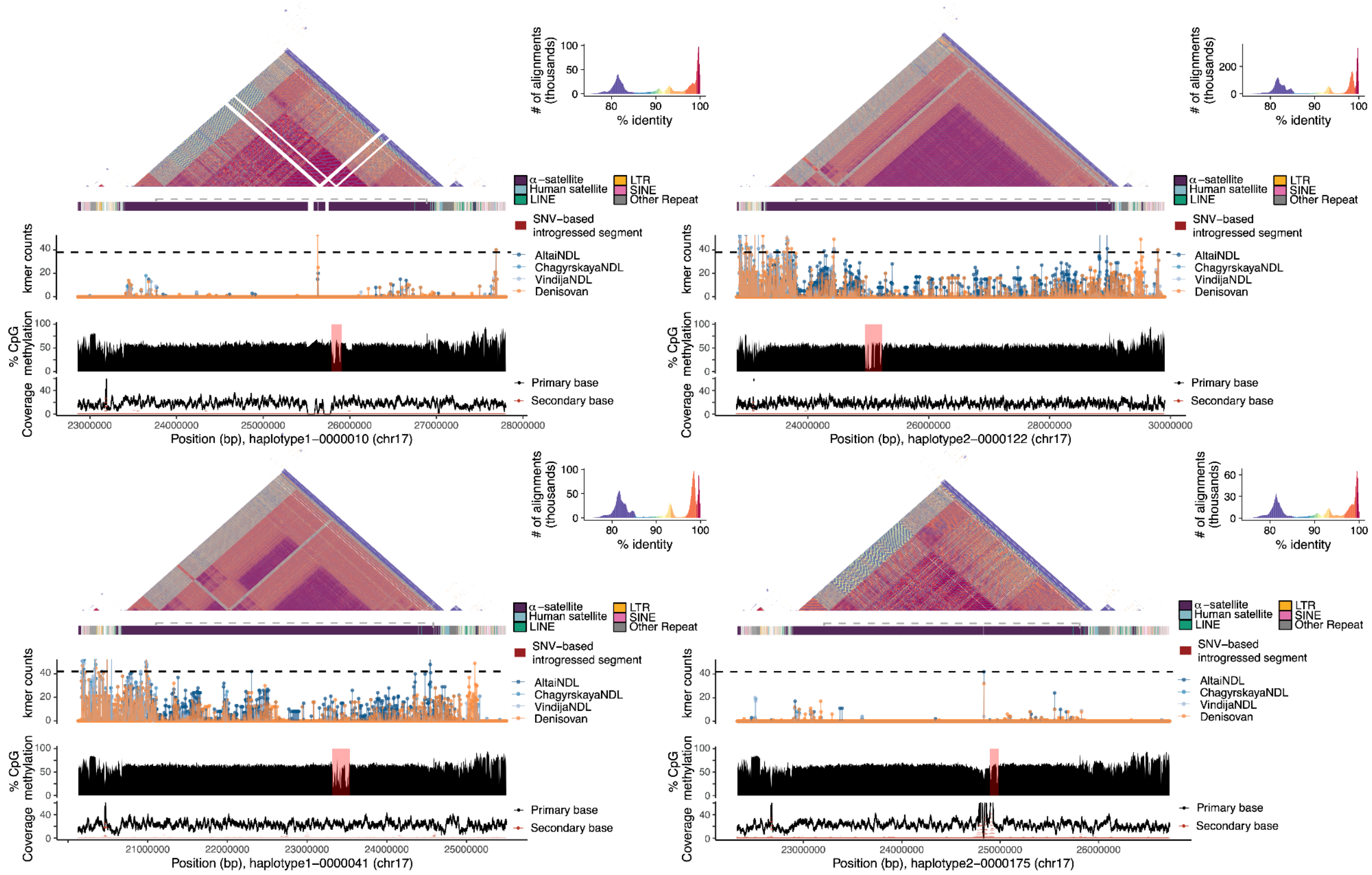

Fig. S70. Assembled chromosome 17 centromeres in PNG15_h1 (top left), PNG15_h2 (top right), PNG16_h1 (bottom left), and PNG16_h2 (bottom right). Top: a heatmap of pairwise sequence identity in 5 kbp windows (StainedGlass) across the centromere and flanking regions. Colors of the heatmap indicate sequence identity as depicted in the inset histogram. The colored vertical bars indicate underlying repeat contents across the centromere regions. The gray dashed line is the inferred centromere sequence based on annotated live α-satellite arrays. Red rectangles represent SNV-based introgression signals. First middle panel: Distribution of archaic-specific k-mers from four high-quality archaic short-read genomes in the centromere region. The black dashed lines indicate the genome-wide k-mer count cutoffs, 38 and 42, that correspond to the genome-wide archaic introgression fraction for PNG15 and PNG16, respectively. Second middle panel: the percentage of averaged methylated CpG frequency across 5-kbp bins across the region. The pink rectangles indicate the putative centromeric hypomethylated loci associated with kinetochore attachment. Bottom: The NucFreq plot for the assembled centromere. Black dots represent the count of the most abundant (primary) base at each position, while red dots indicate the count of the second most frequent (secondary) base. The low representation of secondary bases across the region suggests that the centromere is correctly assembled. **Table S10** provides detailed information about these centromere assemblies.

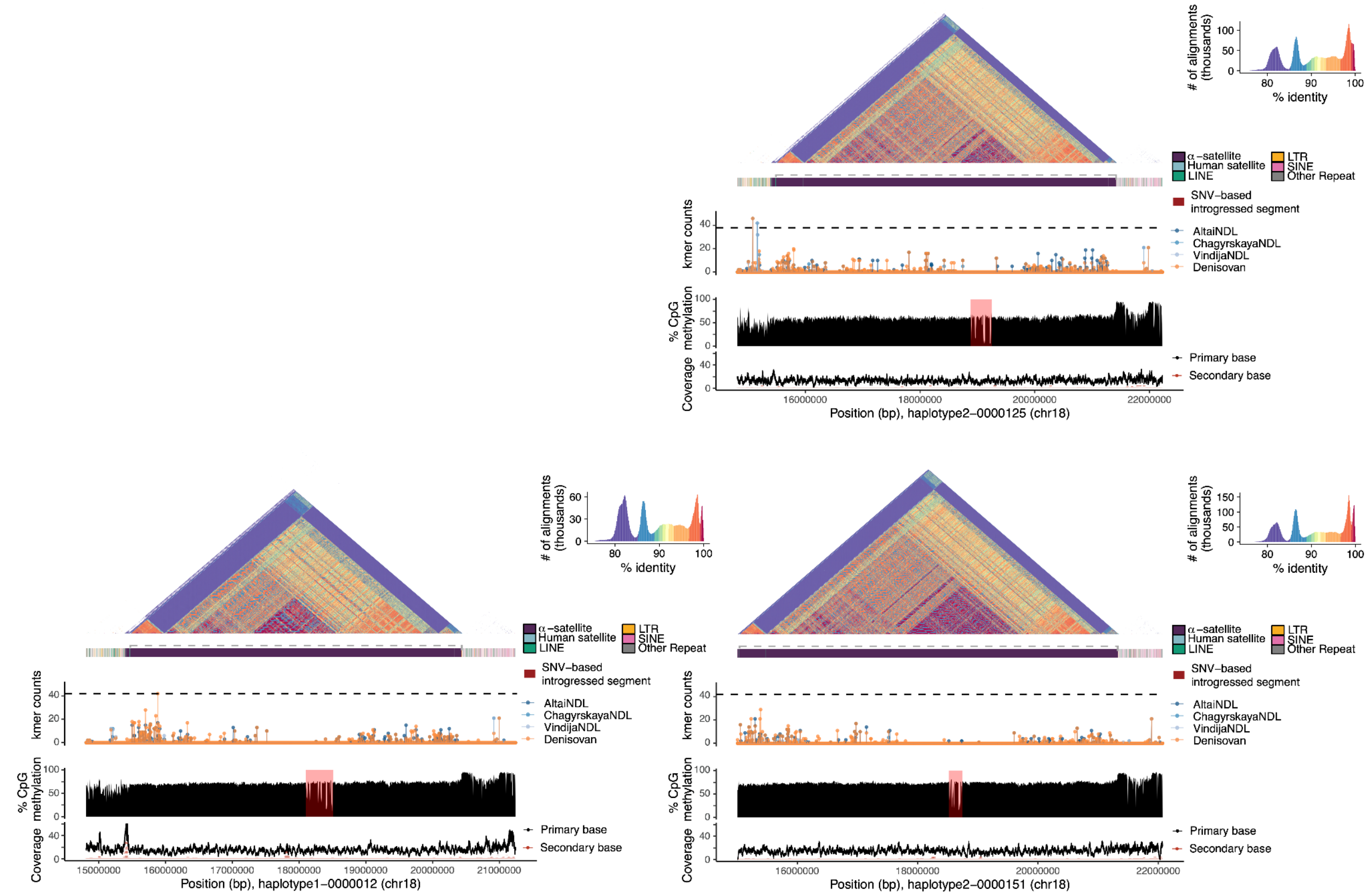

Fig. S71. Assembled chromosome 18 centromeres in PNG15_h2 (top right), PNG16_h1 (bottom left), and PNG16_h2 (bottom right). Top: a heatmap of pairwise sequence identity in 5 kbp windows (StainedGlass) across the centromere and flanking regions. Colors of the heatmap indicate sequence identity as depicted in the inset histogram. The colored vertical bars indicate underlying repeat contents across the centromere regions. The gray dashed line is the inferred centromere sequence based on annotated live α-satellite arrays. Red rectangles represent SNV-based introgression signals. First middle panel: Distribution of archaic-specific k-mers from four high-quality archaic short-read genomes in the centromere region. The black dashed lines indicate the genome-wide k-mer count cutoffs, 38 and 42, that correspond to the genome-wide archaic introgression fraction for PNG15 and PNG16, respectively. Second middle panel: the percentage of averaged methylated CpG frequency across 5-kbp bins across the region. The pink rectangles indicate the putative centromeric hypomethylated loci associated with kinetochore attachment. Bottom: The NucFreq plot for the assembled centromere. Black dots represent the count of the most abundant (primary) base at each position, while red dots indicate the count of the second most frequent (secondary) base. The low representation of secondary bases across the region suggests that the centromere is correctly assembled. **Table S10** provides detailed information about these centromere assemblies.

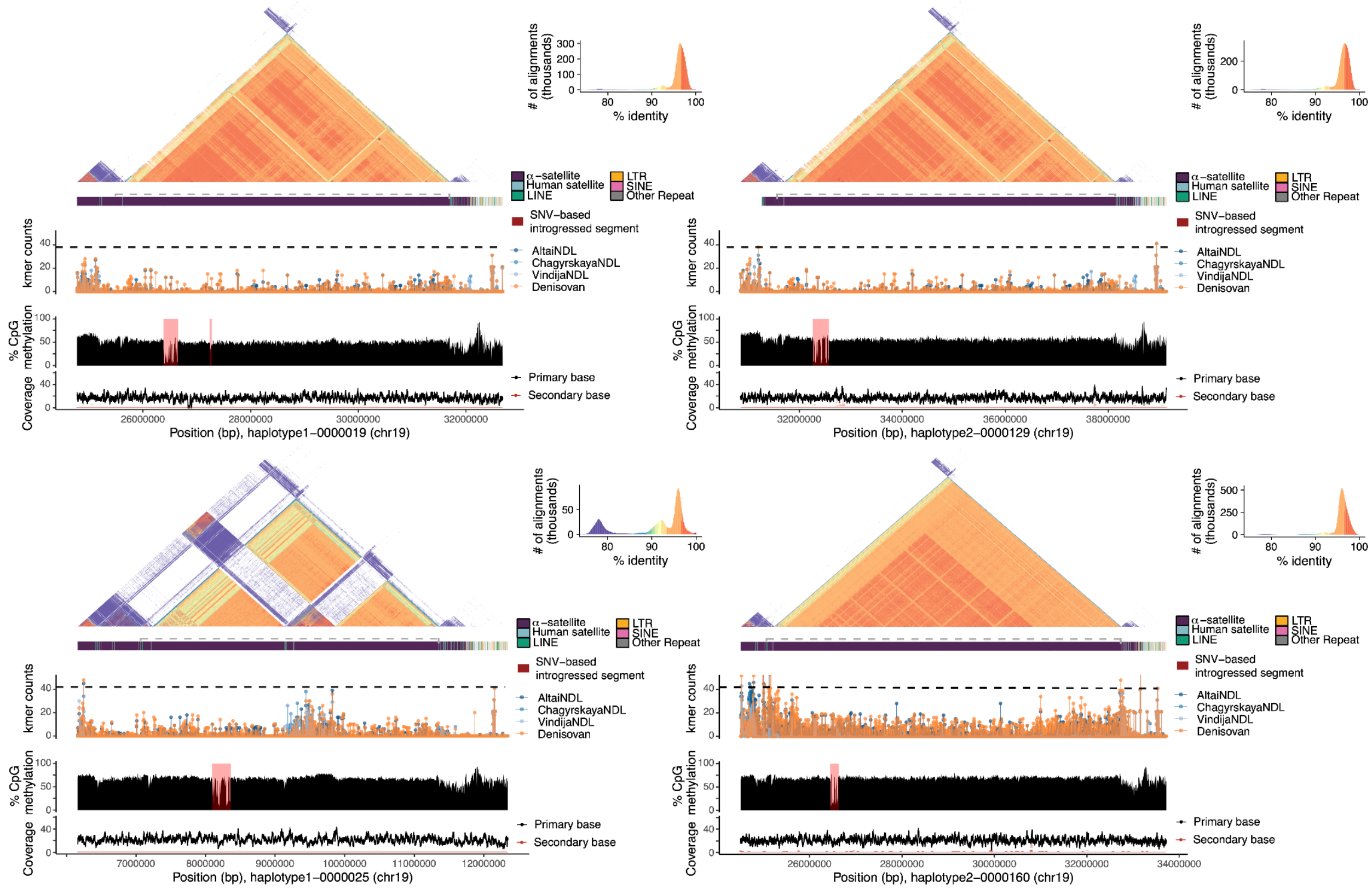

Fig. S72. Assembled chromosome 19 centromeres in PNG15_h1 (top left), PNG15_h2 (top right), PNG16_h1 (bottom left), and PNG16_h2 (bottom right). Top: a heatmap of pairwise sequence identity in 5 kbp windows (StainedGlass) across the centromere and flanking regions. Colors of the heatmap indicate sequence identity as depicted in the inset histogram. The colored vertical bars indicate underlying repeat contents across the centromere regions. The gray dashed line is the inferred centromere sequence based on annotated live α-satellite arrays. Red rectangles represent SNV-based introgression signals. First middle panel: Distribution of archaic-specific k-mers from four high-quality archaic short-read genomes in the centromere region. The black dashed lines indicate the genome-wide k-mer count cutoffs, 38 and 42, that correspond to the genome-wide archaic introgression fraction for PNG15 and PNG16, respectively. Second middle panel: the percentage of averaged methylated CpG frequency across 5-kbp bins across the region. The pink rectangles indicate the putative centromeric hypomethylated loci associated with kinetochore attachment. Bottom: The NucFreq plot for the assembled centromere. Black dots represent the count of the most abundant (primary) base at each position, while red dots indicate the count of the second most frequent (secondary) base. The low representation of secondary bases across the region suggests that the centromere is correctly assembled. **Table S10** provides detailed information about these centromere assemblies.

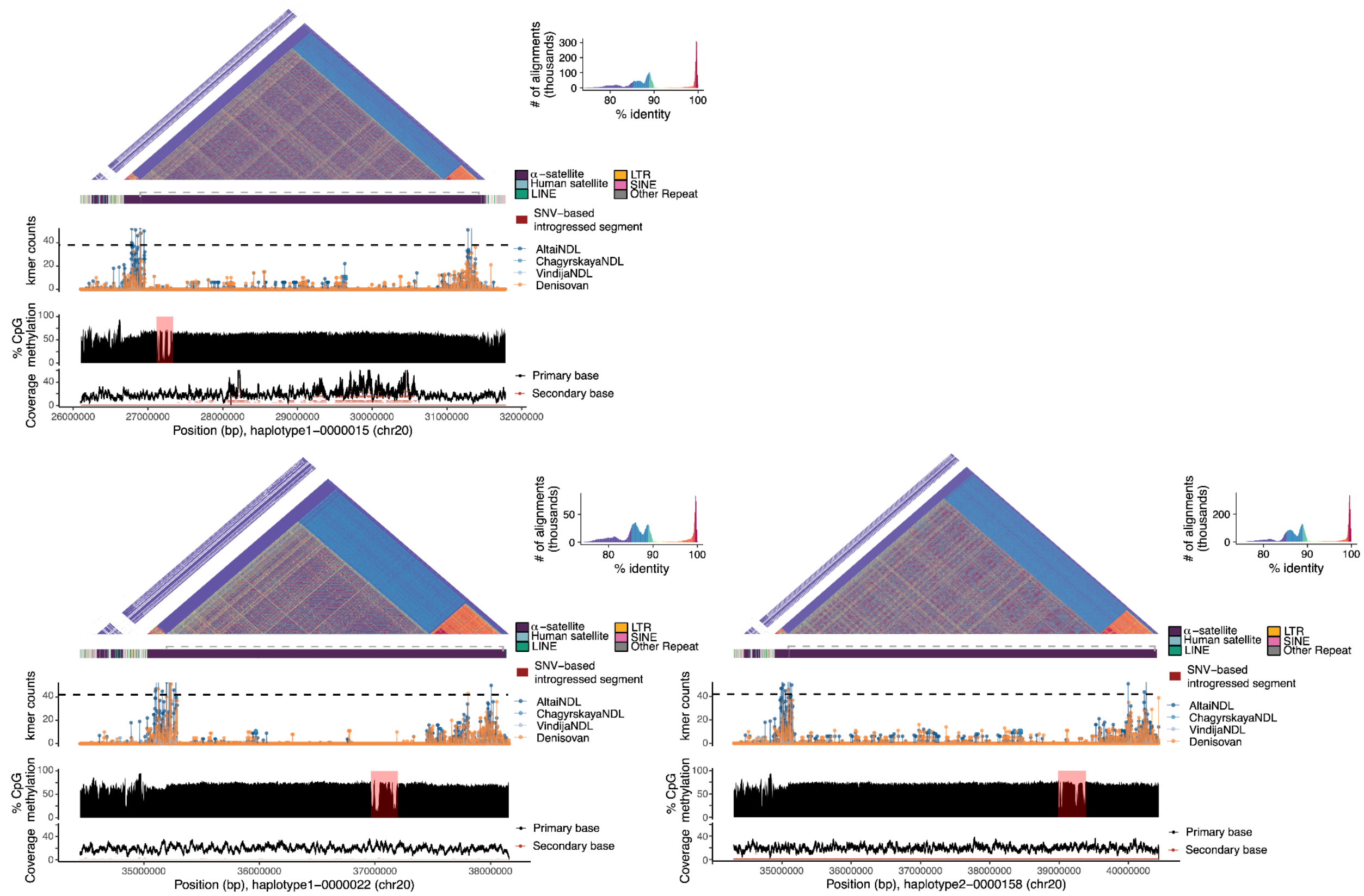

Fig. S73. Assembled chromosome 20 centromeres in PNG15_h1 (top left), PNG16_h1 (bottom left), and PNG16_h2 (bottom right). Top: a heatmap of pairwise sequence identity in 5 kbp windows (StainedGlass) across the centromere and flanking regions. Colors of the heatmap indicate sequence identity as depicted in the inset histogram. The colored vertical bars indicate underlying repeat contents across the centromere regions. The gray dashed line is the inferred centromere sequence based on annotated live α-satellite arrays. Red rectangles represent SNV-based introgression signals. First middle panel: Distribution of archaic-specific k-mers from four high-quality archaic short-read genomes in the centromere region. The black dashed lines indicate the genome-wide k-mer count cutoffs, 38 and 42, that correspond to the genome-wide archaic introgression fraction for PNG15 and PNG16, respectively. Second middle panel: the percentage of averaged methylated CpG frequency across 5-kbp bins across the region. The pink rectangles indicate the putative centromeric hypomethylated loci associated with kinetochore attachment. Bottom: The NucFreq plot for the assembled centromere. Black dots represent the count of the most abundant (primary) base at each position, while red dots indicate the count of the second most frequent (secondary) base. The low representation of secondary bases across the region suggests that the centromere is correctly assembled. **Table S10** provides detailed information about these centromere assemblies.

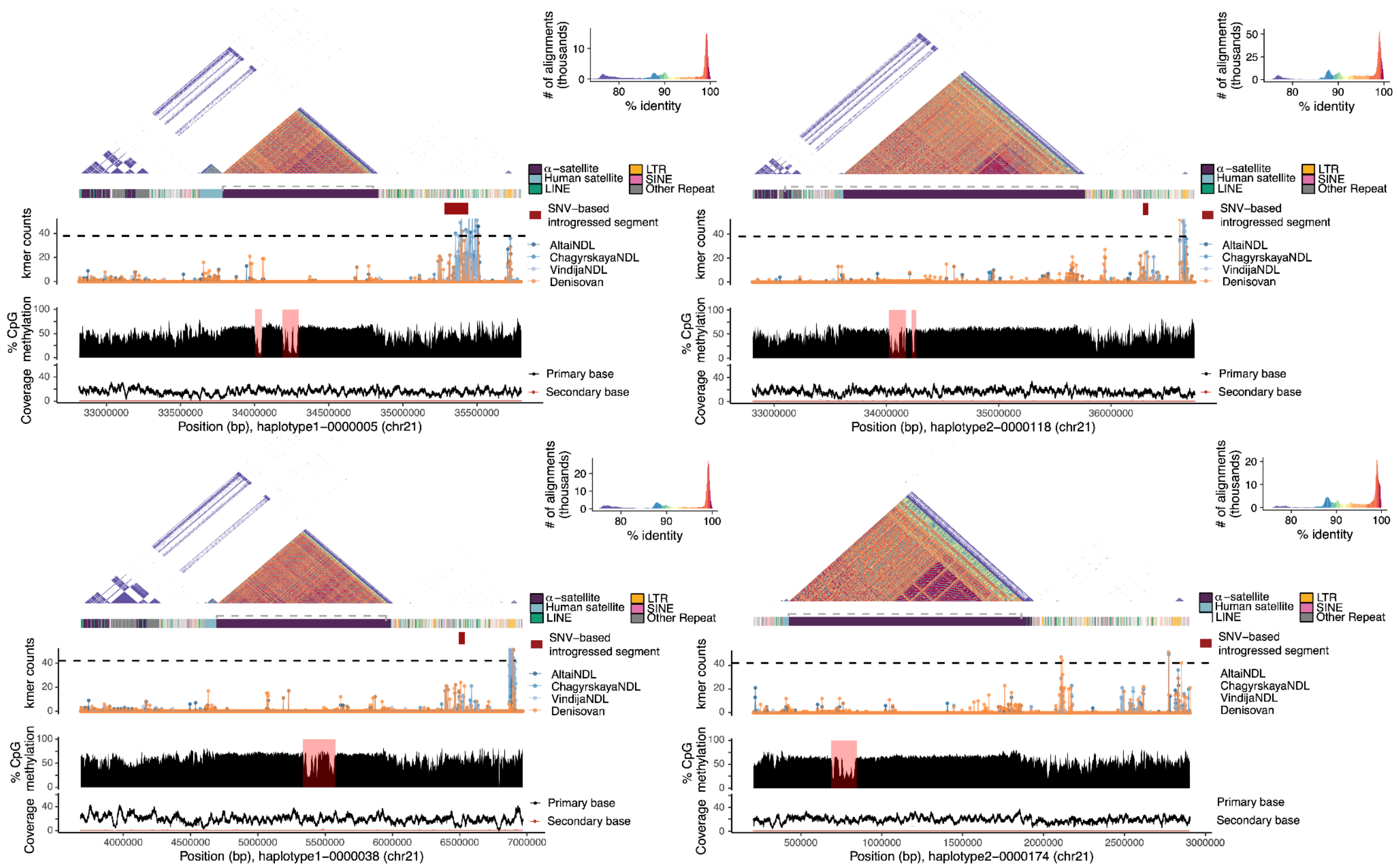

Fig. S74. Assembled chromosome 21 centromeres in PNG15_h1 (top left), PNG15_h2 (top right), PNG16_h1 (bottom left), and PNG16_h2 (bottom right). Top: a heatmap of pairwise sequence identity in 5 kbp windows (StainedGlass) across the centromere and flanking regions. Colors of the heatmap indicate sequence identity as depicted in the inset histogram. The colored vertical bars indicate underlying repeat contents across the centromere regions. The gray dashed line is the inferred centromere sequence based on annotated live α-satellite arrays. Red rectangles represent SNV-based introgression signals. First middle panel: Distribution of archaic-specific k-mers from four high-quality archaic short-read genomes in the centromere region. The black dashed lines indicate the genome-wide k-mer count cutoffs, 38 and 42, that correspond to the genome-wide archaic introgression fraction for PNG15 and PNG16, respectively. Second middle panel: the percentage of averaged methylated CpG frequency across 5-kbp bins across the region. The pink rectangles indicate the putative centromeric hypomethylated loci associated with kinetochore attachment. Bottom: The NucFreq plot for the assembled centromere. Black dots represent the count of the most abundant (primary) base at each position, while red dots indicate the count of the second most frequent (secondary) base. The low representation of secondary bases across the region suggests that the centromere is correctly assembled. **Table S10** provides detailed information about these centromere assemblies.

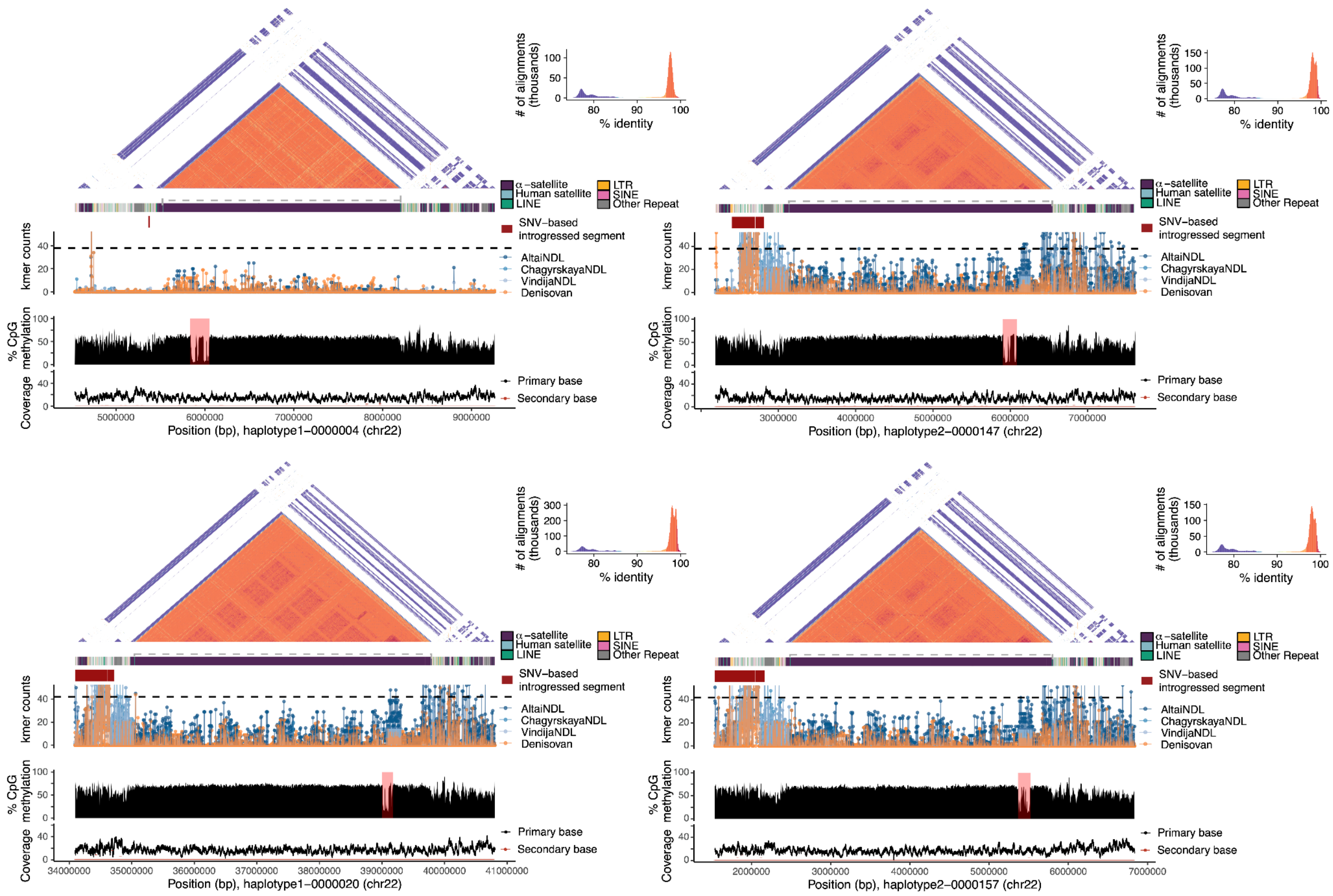

Fig. S75. Assembled chromosome 22 centromeres in PNG15_h1 (top left), PNG15_h2 (top right), PNG16_h1 (bottom left), and PNG16_h2 (bottom right). Top: a heatmap of pairwise sequence identity in 5 kbp windows (StainedGlass) across the centromere and flanking regions. Colors of the heatmap indicate sequence identity as depicted in the inset histogram. The colored vertical bars indicate underlying repeat contents across the centromere regions. The gray dashed line is the inferred centromere sequence based on annotated live α-satellite arrays. Red rectangles represent SNV-based introgression signals. First middle panel: Distribution of archaic-specific k-mers from four high-quality archaic short-read genomes in the centromere region. The black dashed lines indicate the genome-wide k-mer count cutoffs, 38 and 42, that correspond to the genome-wide archaic introgression fraction for PNG15 and PNG16, respectively. Second middle panel: the percentage of averaged methylated CpG frequency across 5-kbp bins across the region. The pink rectangles indicate the putative centromeric hypomethylated loci associated with kinetochore attachment. Bottom: The NucFreq plot for the assembled centromere. Black dots represent the count of the most abundant (primary) base at each position, while red dots indicate the count of the second most frequent (secondary) base. The low representation of secondary bases across the region suggests that the centromere is correctly assembled. **Table S10** provides detailed information about these centromere assemblies.

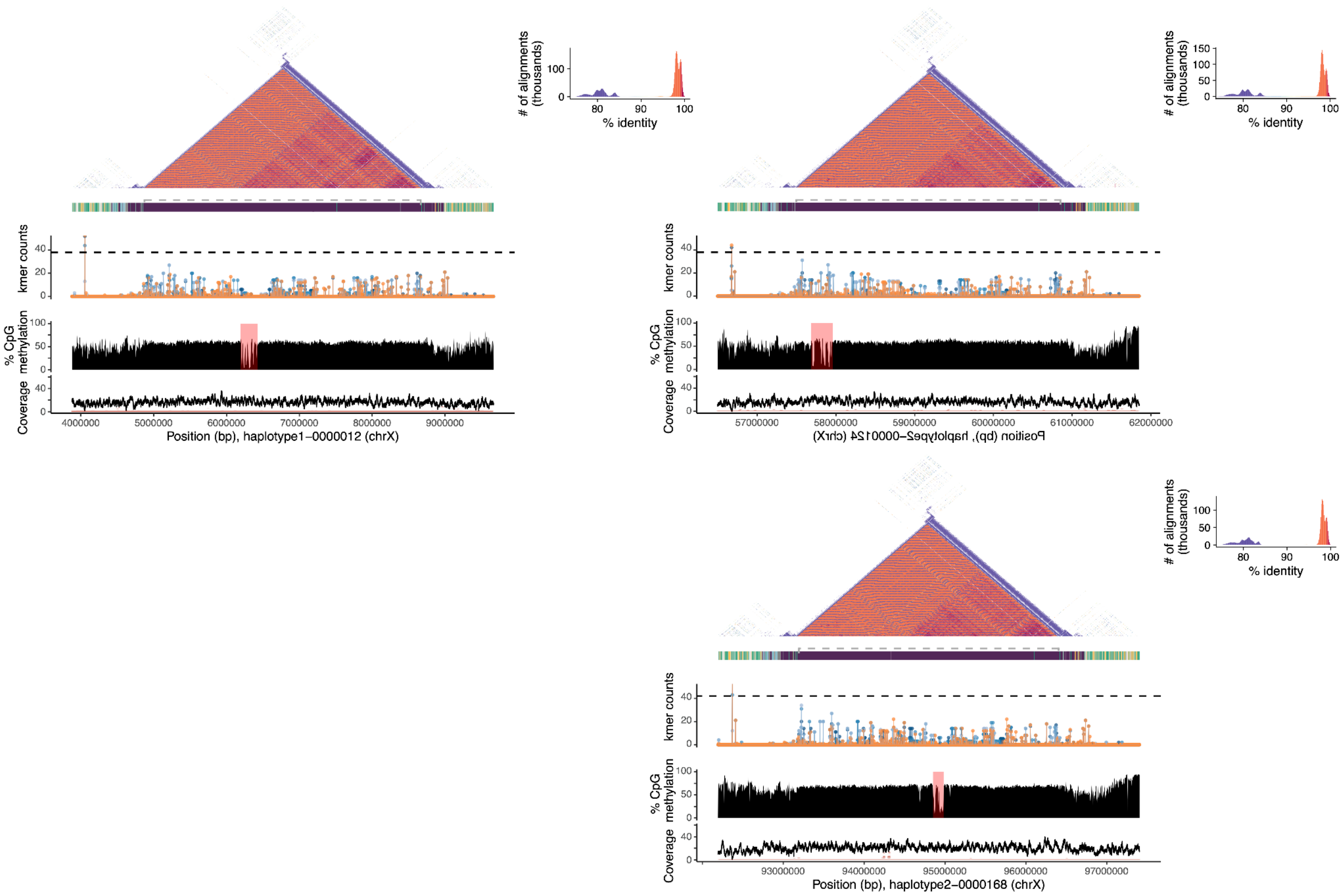

Fig. S76. Assembled chromosome X centromeres in PNG15_h1 (top left), PNG15_h2 (top right), and PNG16_h2 (bottom right). Top: a heatmap of pairwise sequence identity in 5 kbp windows (StainedGlass) across the centromere and flanking regions. Colors of the heatmap indicate sequence identity as depicted in the inset histogram. The colored vertical bars indicate underlying repeat contents across the centromere regions. The gray dashed line is the inferred centromere sequence based on annotated live α-satellite arrays. Red rectangles represent SNV-based introgression signals. First middle panel: Distribution of archaic-specific k-mers from four high-quality archaic short-read genomes in the centromere region. The black dashed lines indicate the genome-wide k-mer count cutoffs, 38 and 42, that correspond to the genome-wide archaic introgression fraction for PNG15 and PNG16, respectively. Second middle panel: the percentage of averaged methylated CpG frequency across 5-kbp bins across the region. The pink rectangles indicate the putative centromeric hypomethylated loci associated with kinetochore attachment. Bottom: The NucFreq plot for the assembled centromere. Black dots represent the count of the most abundant (primary) base at each position, while red dots indicate the count of the second most frequent (secondary) base. The low representation of secondary bases across the region suggests that the centromere is correctly assembled. **Table S10** provides detailed information about these centromere assemblies.

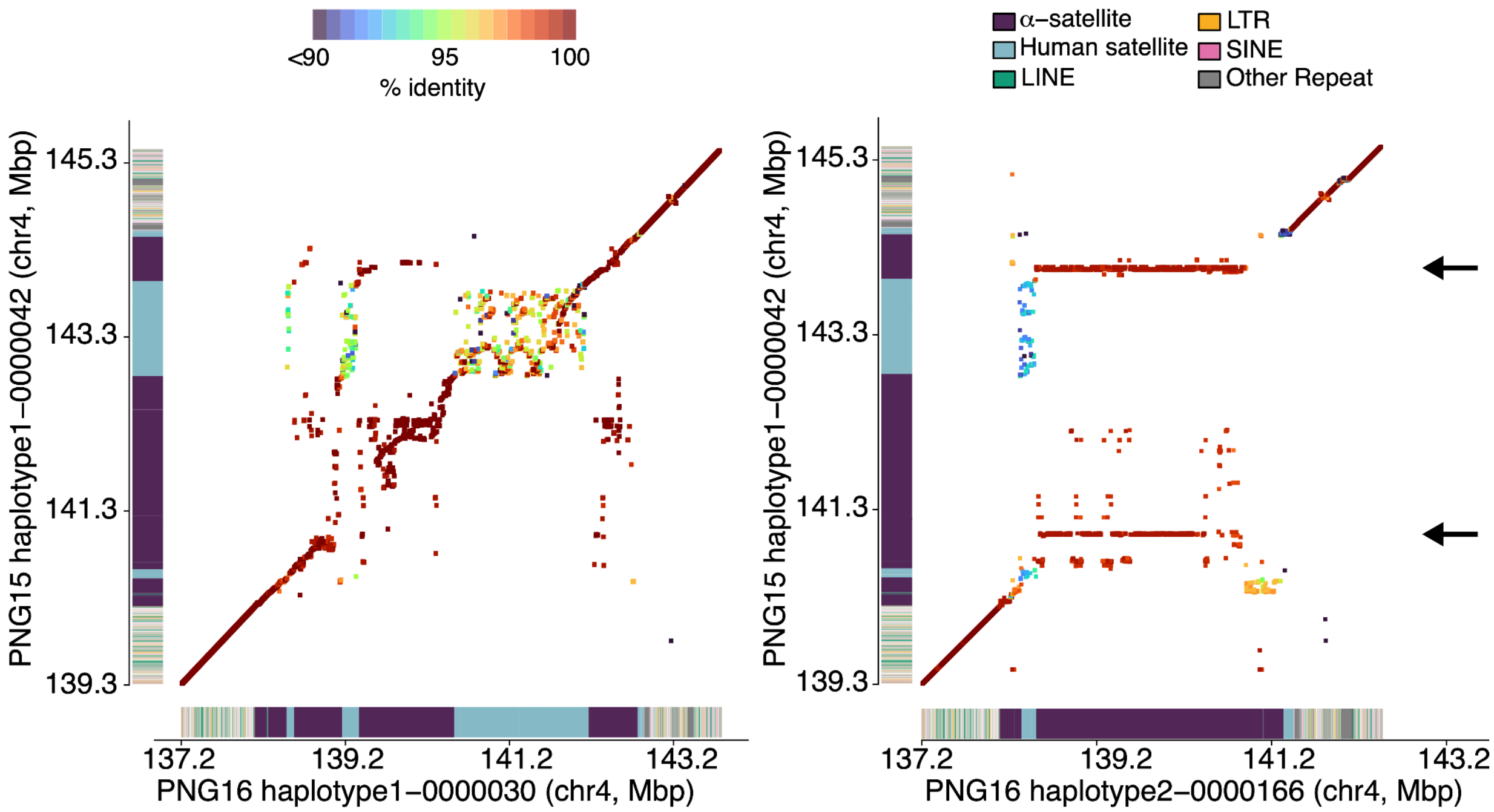

Fig. S77. Allelic variation between putative introgressed and modern human chromosome 4 centromeric haplotypes. (Left) comparison of modern human centromeres and (Right) comparison of putative introgressed centromere (PNG16 haplotype 2-0000166) vs. a non-introgressed centromere. The 20 kbp windows with a 1 kbp sliding step from the query sequence (horizontal axis) were aligned to the target sequence (vertical axis). Only the best alignments, based on sequence identity, were retained. Colors of dots indicate percent sequence identity. The α-satellite and other human satellite array structure is shown on the axes as those in **Fig. 6**. The black arrows on the right panel indicate the modern human α-satellite HORs expanded in the putative archaic centromeric sequences.

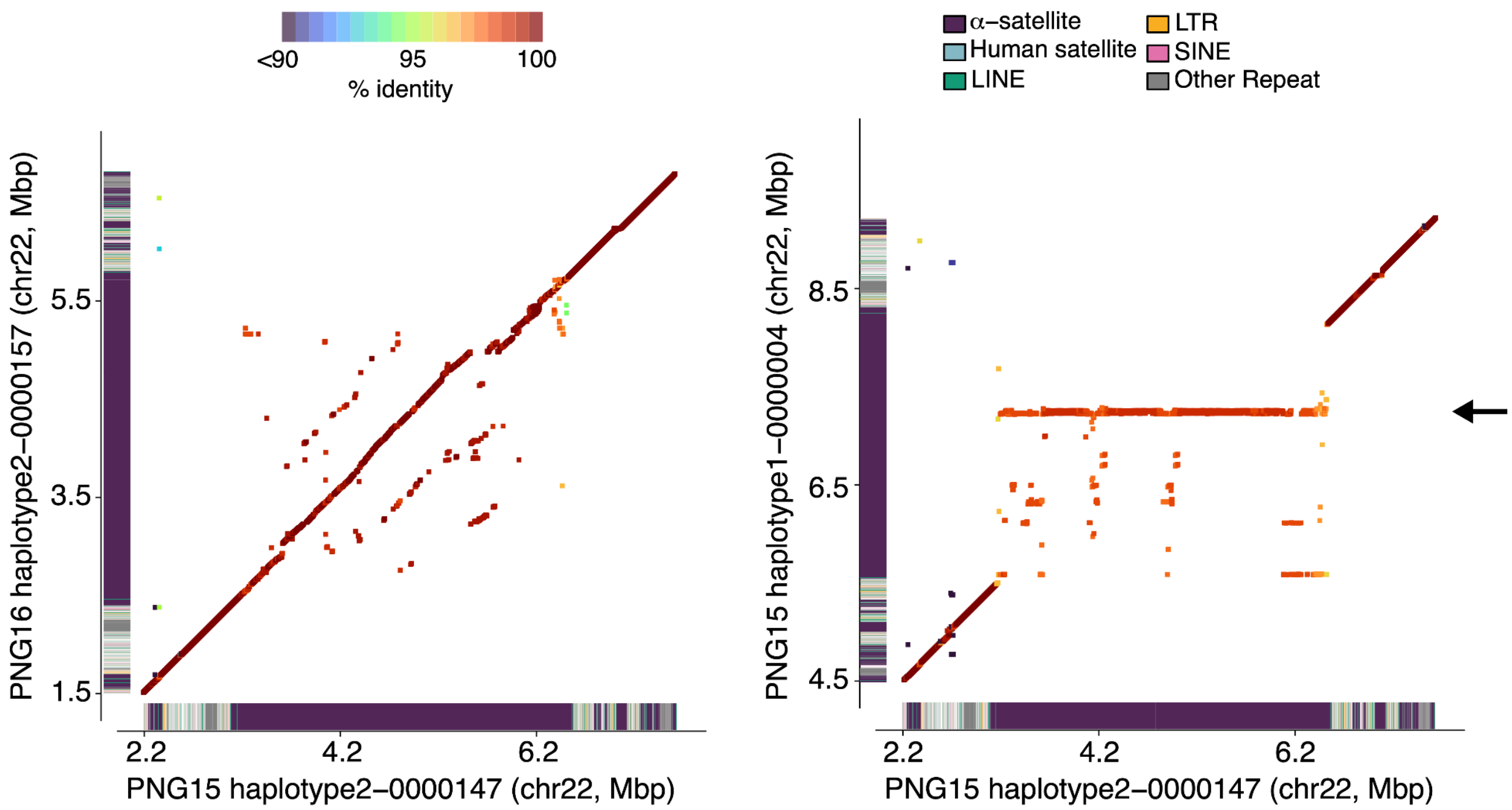

Fig. S78. Allelic variation between putative introgressed and modern human chromosome 22 centromeric haplotypes. (Left) comparison of two putative archaic human centromeres and (Right) comparison between an archaic and a modern human centromeric haplotypes (archaic centromeric sequence: PNG15 haplotype 2-0000147). The 20 kbp windows with a 1 kbp sliding step from the query sequence (horizontal axis) were aligned to the target sequence (vertical axis). Only the best alignments, based on sequence identity, were retained. Colors of dots indicate percent sequence identity. The α-satellite and other human satellite array structure are shown on the axes as those in **Fig. 6**. The black arrow on the right panel indicates the modern human α-satellite HORs expanded in the putative archaic centromeric sequences.

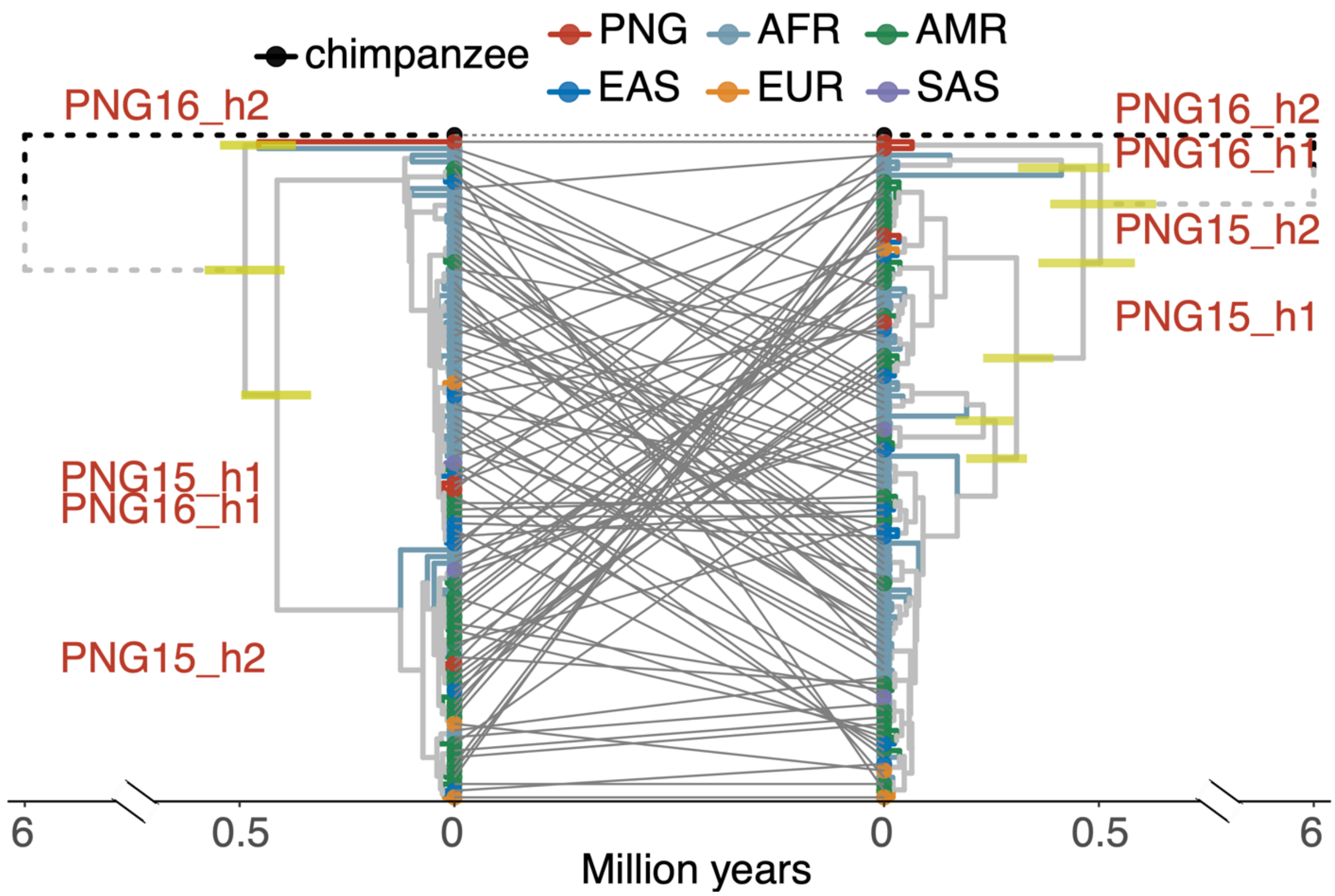

Fig. S79. Phylogenies using the putative introgressed sequences from the left and right flanking sequences of chromosome 4 centromere in PNG16_h2 and its orthologous sequences from PNG and HPRCr1 haplotypes. The ancestral branches leading to the chimpanzee and ancestral lineages of humans are truncated (dashed lines) for illustrative purposes. 95% high posterior density intervals for node ages between 0.2 and 1.0 million years are displayed as horizontal yellow lines. Gray lines connect the same haplotype assemblies.

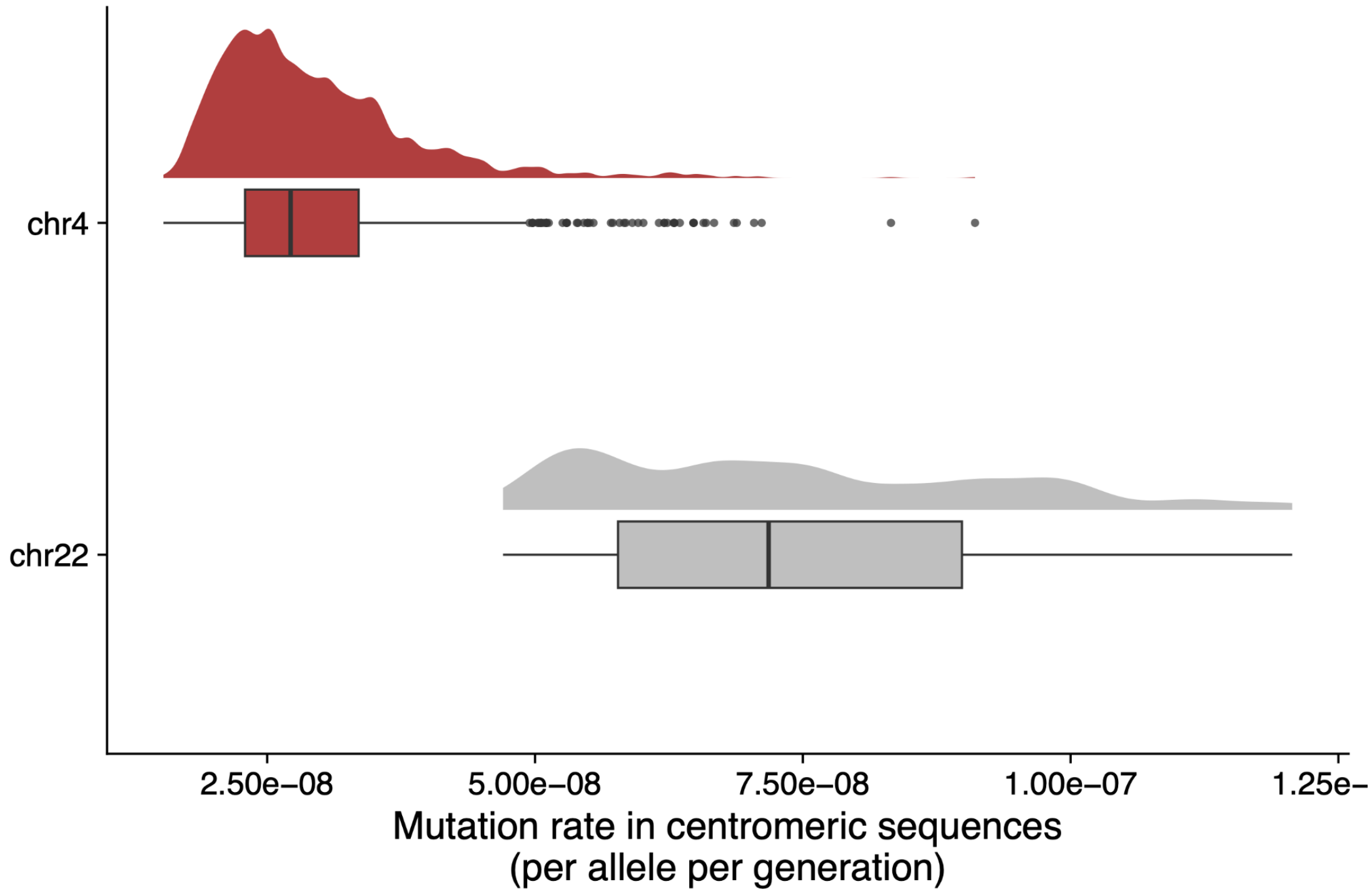

Fig. S80. Estimated mutation rates of α-satellite HORs in the centromeric sequences of chromosomes 4 and 22. Mutation rates are estimated using an evolutionary model assuming a human-archaic divergence of 500-800 thousand years ago (**Methods**). Allelic differences are computed using alignments between the putative archaic and modern α-satellite HOR sequences focusing on loci associated with the expansion of human α-satellite HORs in the putative archaic centromeres (black arrows in **Figs. S77-S78**). Boxplots display the median (thick line), upper and lower quartiles (box), and 1.5× interquartile range (whiskers).

Supplementary Tables

Table S1. Summary of the high-quality haplotype assemblies for two PNG individuals using PacBio HiFi and Oxford Nanopore Technologies (ONT) sequencing. The complete human reference T2T-CHM13 (v1.1) is listed for comparison. The X and Y superscripts indicate the inclusion of sex chromosomes in individual haplotype assemblies.

Table S2. Summary for Flagger-detected assembly errors.

Table S3. Summary of NucFreq-detected assembly errors.

Table S4. Assembly completeness based on the assessment of single-copy orthologs (n=13,780) across primate taxa using compleasm.

Table S5. Length comparisons of PAV-specific vs. PAV with secondary support from at least one additional caller.

Table S6. PNG assembly-specific structural variants (SVs). Raw SV calls found exclusively in the PNG assemblies compared against the HPRC cohort. SV frequency is computed based on PanGenie-inferred genotypes from the PNG and 1000 Genomes Project (1KG) short-read genomes. Gene IDs, where SVs overlap with their gene body (within 1,000 base pairs on their flanking) and/or exons, are reported. Archaic origin: DNS (Denisovan), NDL (Neanderthal), unresolved. NA: not available. For insertions, the fraction of SVs overlapped with segmental duplications (SDs) is either 0 and 1, referring to the insertion site is either not or in SDs, respectively.

Table S7. Inferred introgressed segments in PNG and HPRCr1 non-African haplotypes. NDL: Neanderthal. DNS: Denisovan.

Table S8. Gene ontology analysis using Panther Overrepresentation Test (released 20240807) for shared introgressed SVs among PNG and HPRC non-African genomes. Analysis was done using GO Ontology database (DOI: 10.5281/zenodo.12173881 Released 2024-06-17) and Fisher's exact test with Bonferroni's correction for p-values.

Table S9. Candidates of genome-wide selection and introgression scans. Data used in genome-wide scans were based on biallelic SNVs inferred from 71 PNG, 703 African, and 585 East Asian samples from the high-coverage 1000 Genomes Project dataset using PanGenie. PBS: population branch statistic. fD: introgression index. P-values were computed using coalescent simulations under realistic neutral demographic models. Gene IDs, where candidates overlap with their exonic sequences, are reported.
Archaic origin: DNS (Denisovan), NDL (Neanderthal), unresolved. NA: not available.

Table S10. Candidates of introgressed and/or adaptive SVs in the PNG cohort.

Table S11. Summary of assembled centromeres in PNG samples. Evidence for archaic introgressed centromeres is included.

Table S12. Summary of incomplete lineage sorting (ILS) analysis for putative introgressed centromeres.
